# Supplementary material for: The Basic Reproduction Number as a Predictor for Epidemic Outbreaks in Temporal Networks
Source: PLoS One. 2015 Mar 20;10(3):e0120567. doi: 10.1371/journal.pone.0120567 (PMC4368036; doi:10.1371/journal.pone.0120567)
Supplement: S1 Fig — This file contains plots corresponding to Fig. 1 for all 69 days of data for the Gallery data set. (PDF) [file pone.0120567.s001.pdf]

The basic reproduction number as a predictor for epidemic outbreaks in temporal networks

Supporting Information 1, R_0 vs. Ω plots for the *Gallery* data

Petter Holme, Naoki Masuda

In chronological order.

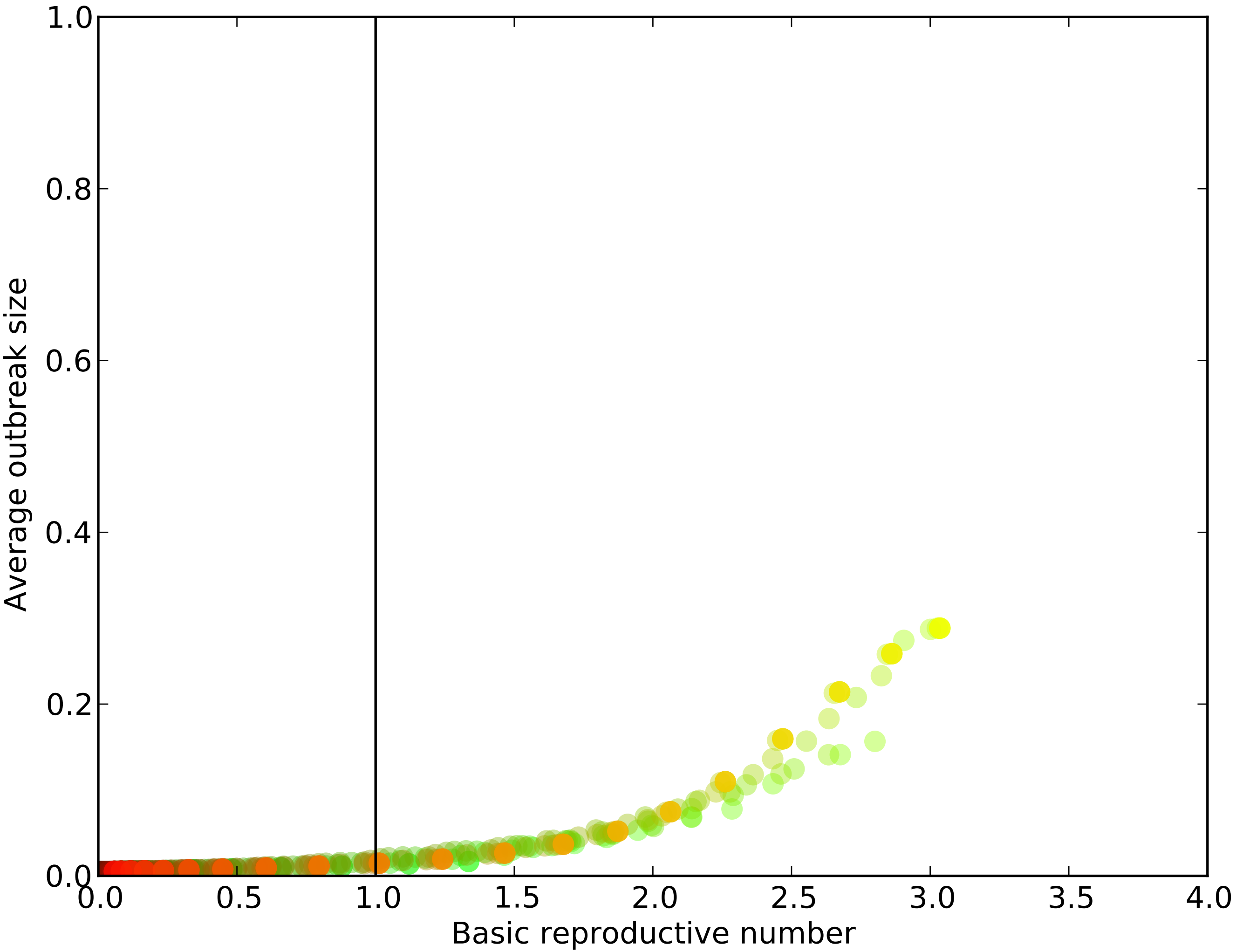
1.0
0.8
0.6
0.4
0.2
0.0
Average outbreak size
0.0
0.5
1.0
1.5
2.0
2.5
3.0
3.5
4.0
Basic reproductive number

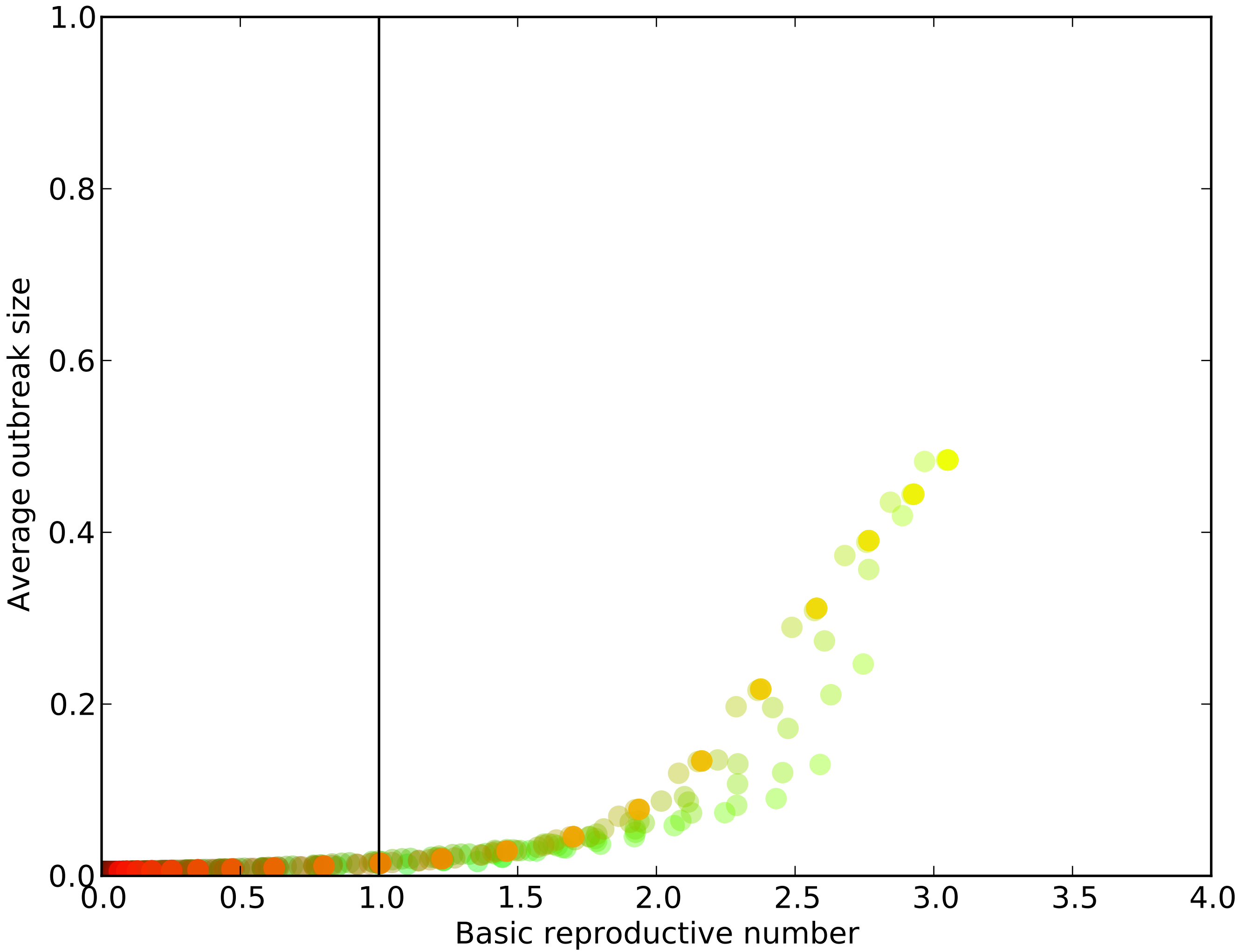
1.0
0.8
0.6
0.4
0.2
0.0
Average outbreak size
0.0
0.5
1.0
1.5
2.0
2.5
3.0
3.5
4.0
Basic reproductive number

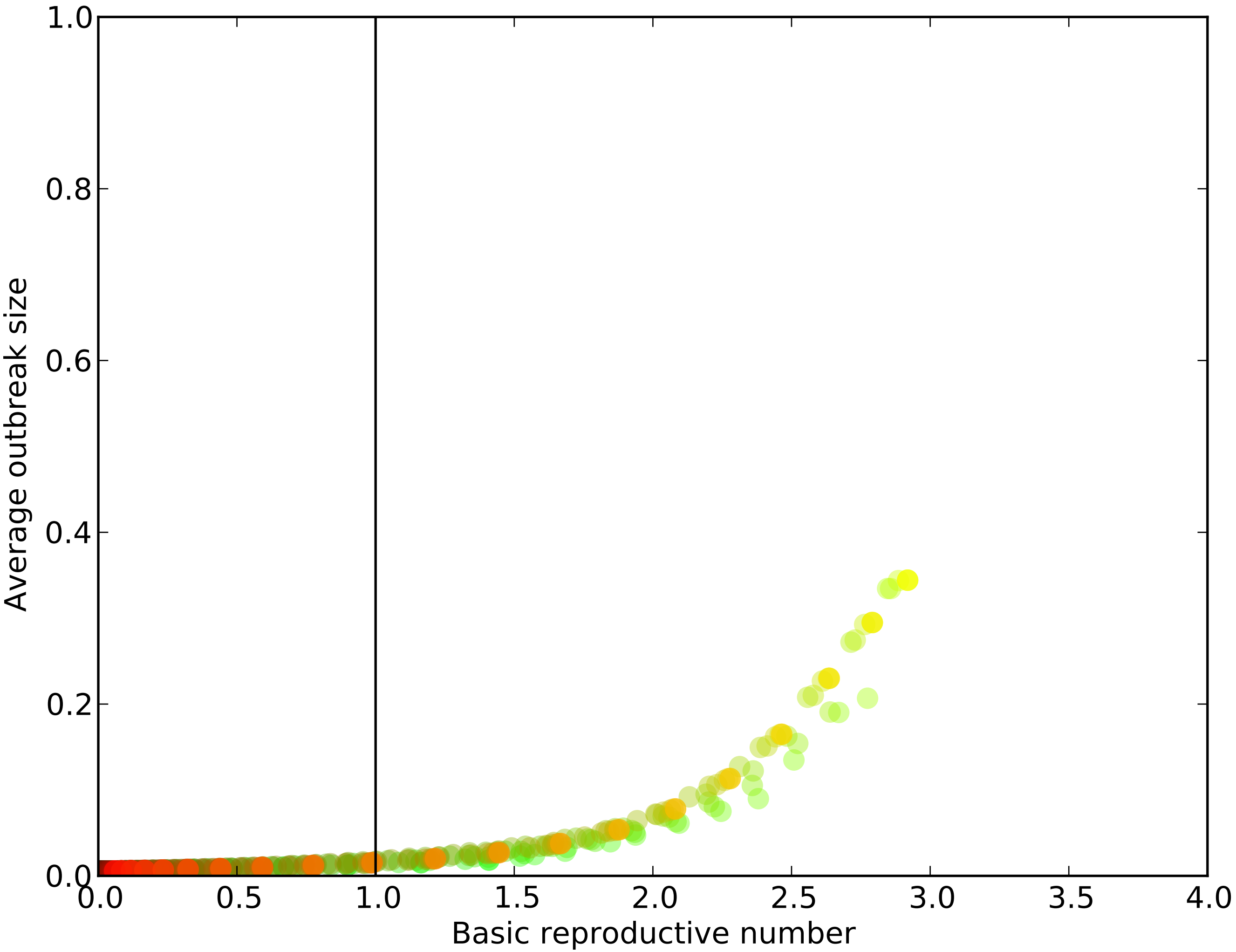

1.0
0.8
0.6
0.4
0.2
0.0
Average outbreak size
0.0
0.5
1.0
1.5
2.0
2.5
3.0
3.5
4.0
Basic reproductive number

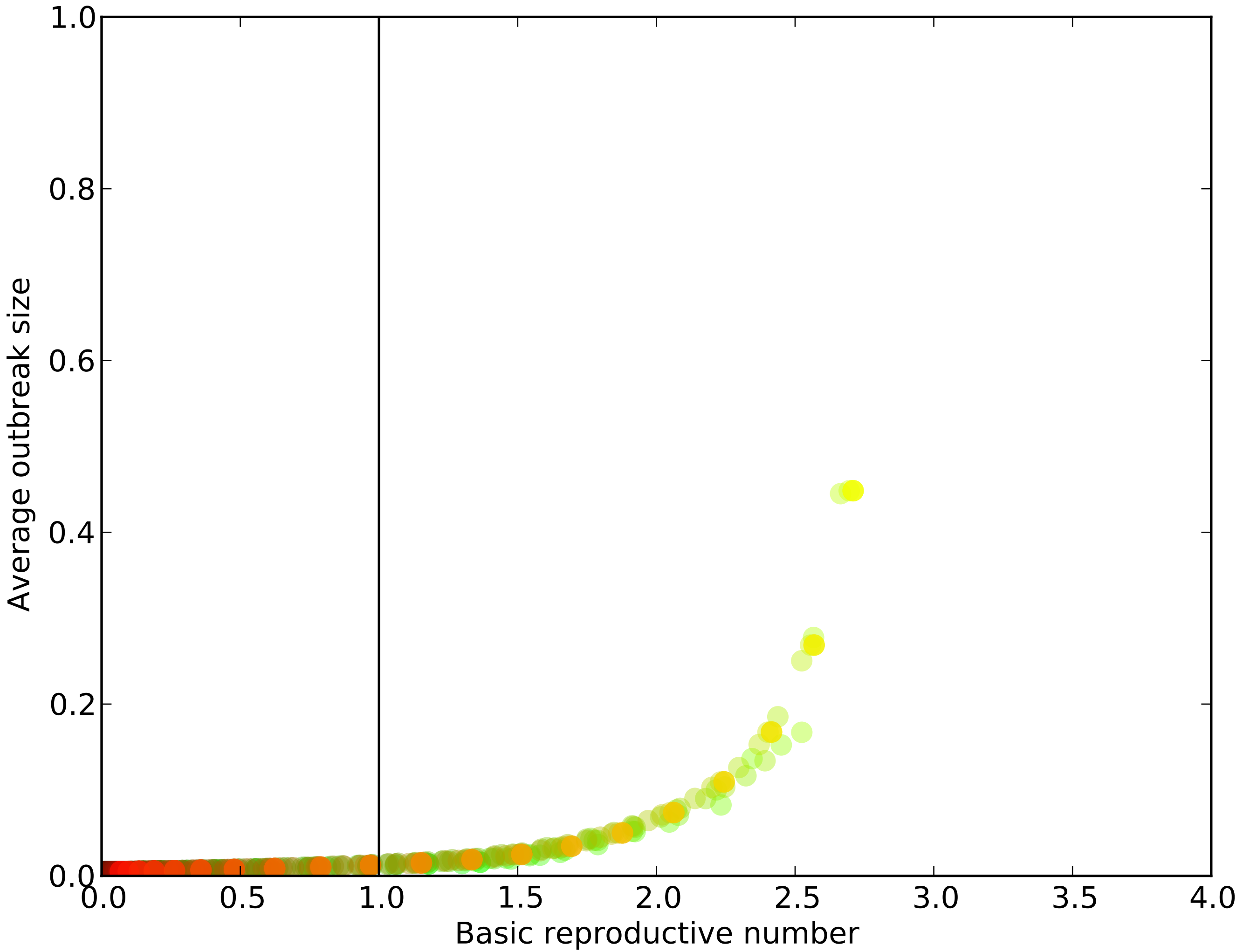

1.0
0.8
0.6
0.4
0.2
0.0
0.0
0.5
1.0
1.5
2.0
2.5
3.0
3.5
4.0
Average outbreak size
Basic reproductive number

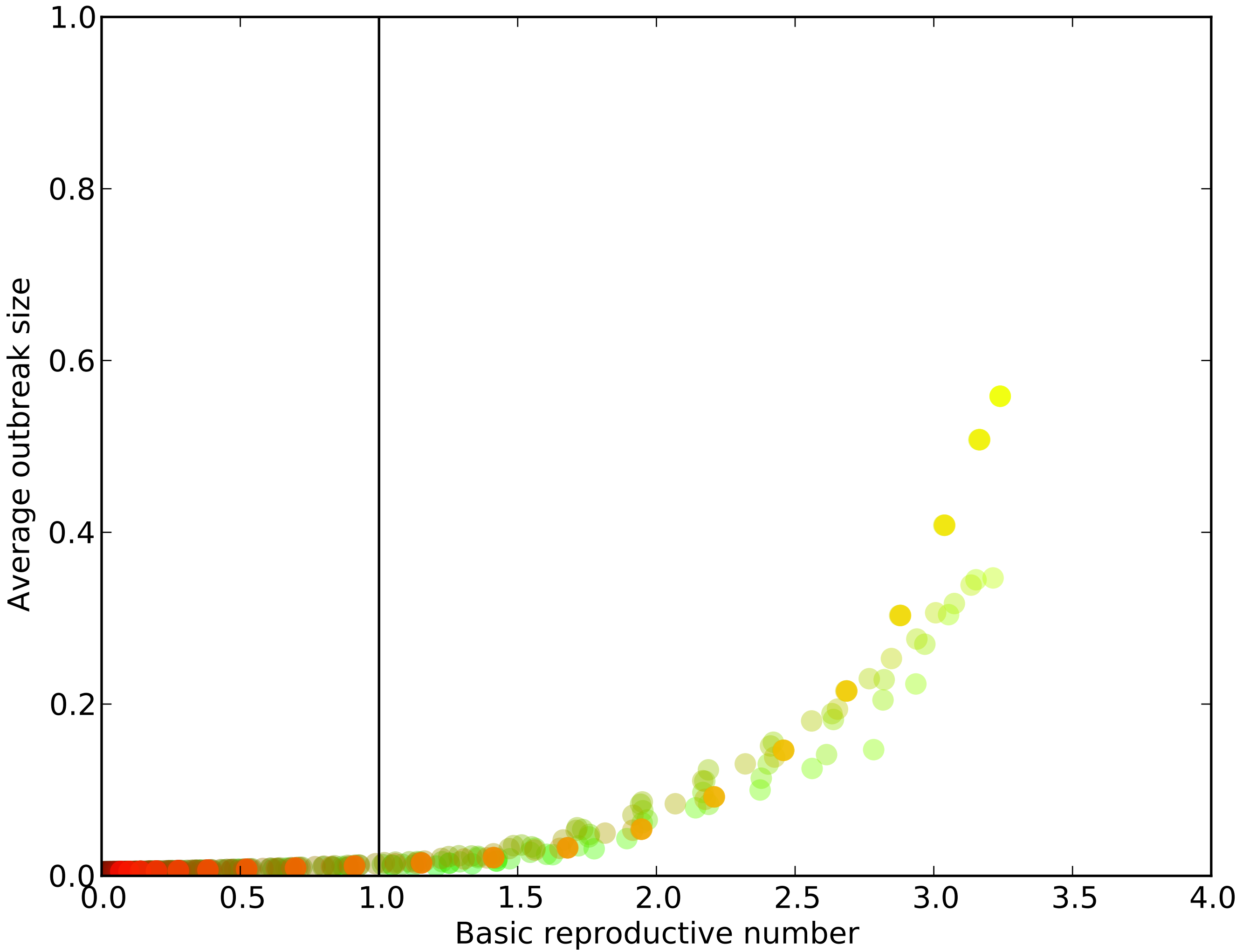

1.0
0.8
0.6
0.4
0.2
0.0
Average outbreak size
0.0
0.5
1.0
1.5
2.0
2.5
3.0
3.5
4.0
Basic reproductive number

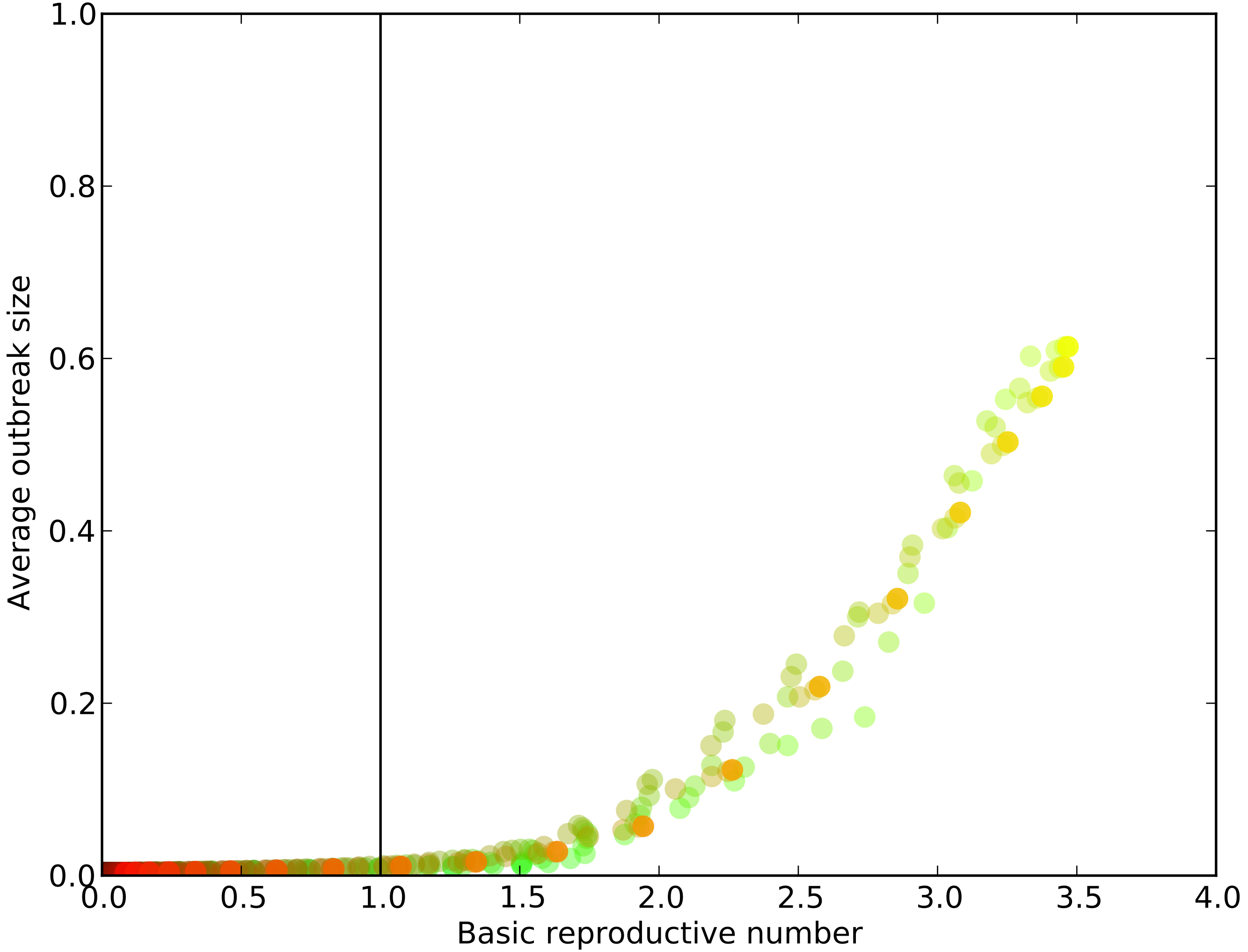

1.0
0.8
0.6
0.4
0.2
0.0
0.0
0.5
1.0
1.5
2.0
2.5
3.0
3.5
4.0
Average outbreak size
Basic reproductive number

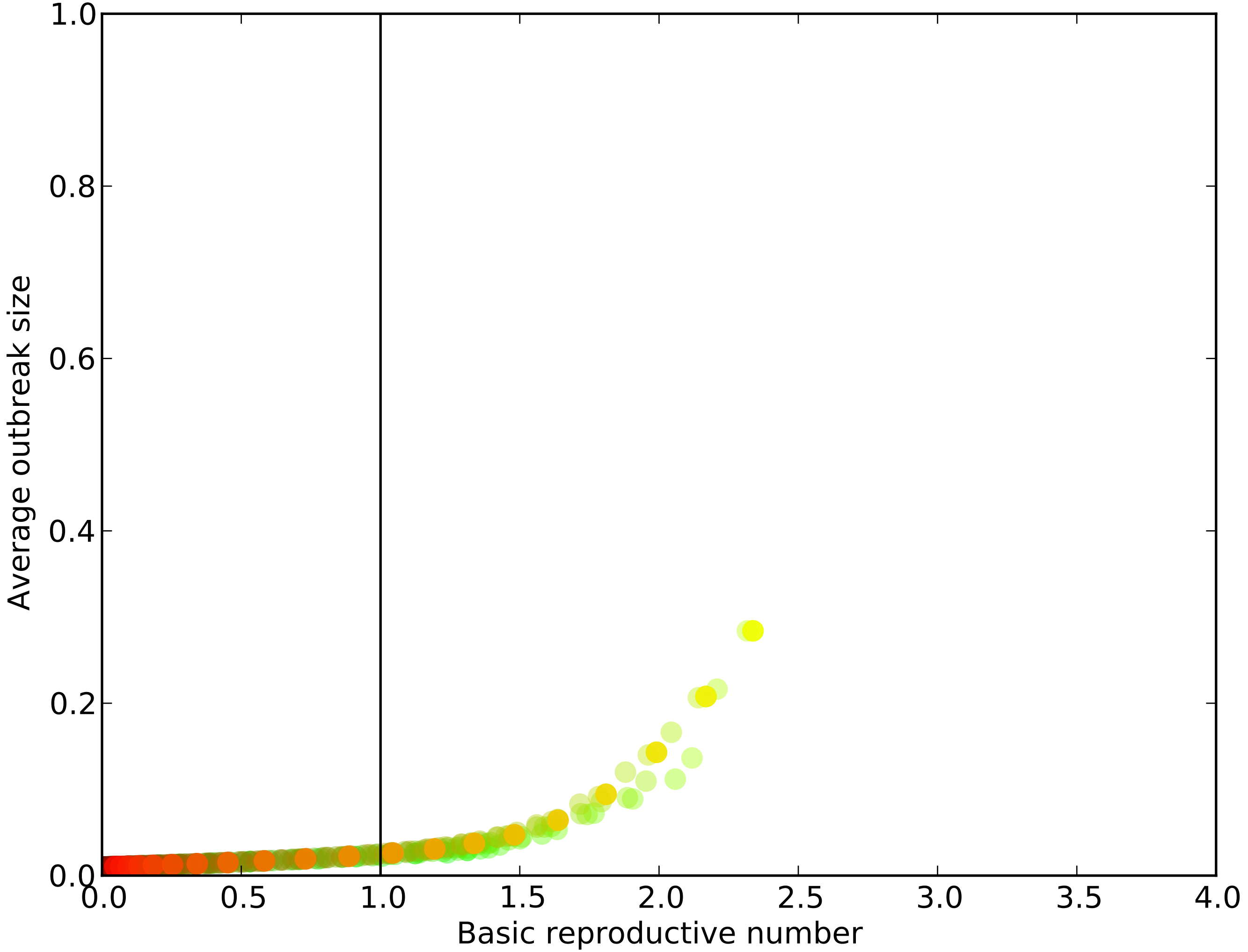

Average outbreak size
Basic reproductive number
1.0
0.8
0.6
0.4
0.2
0.0
0.0
0.5
1.0
1.5
2.0
2.5
3.0
3.5
4.0

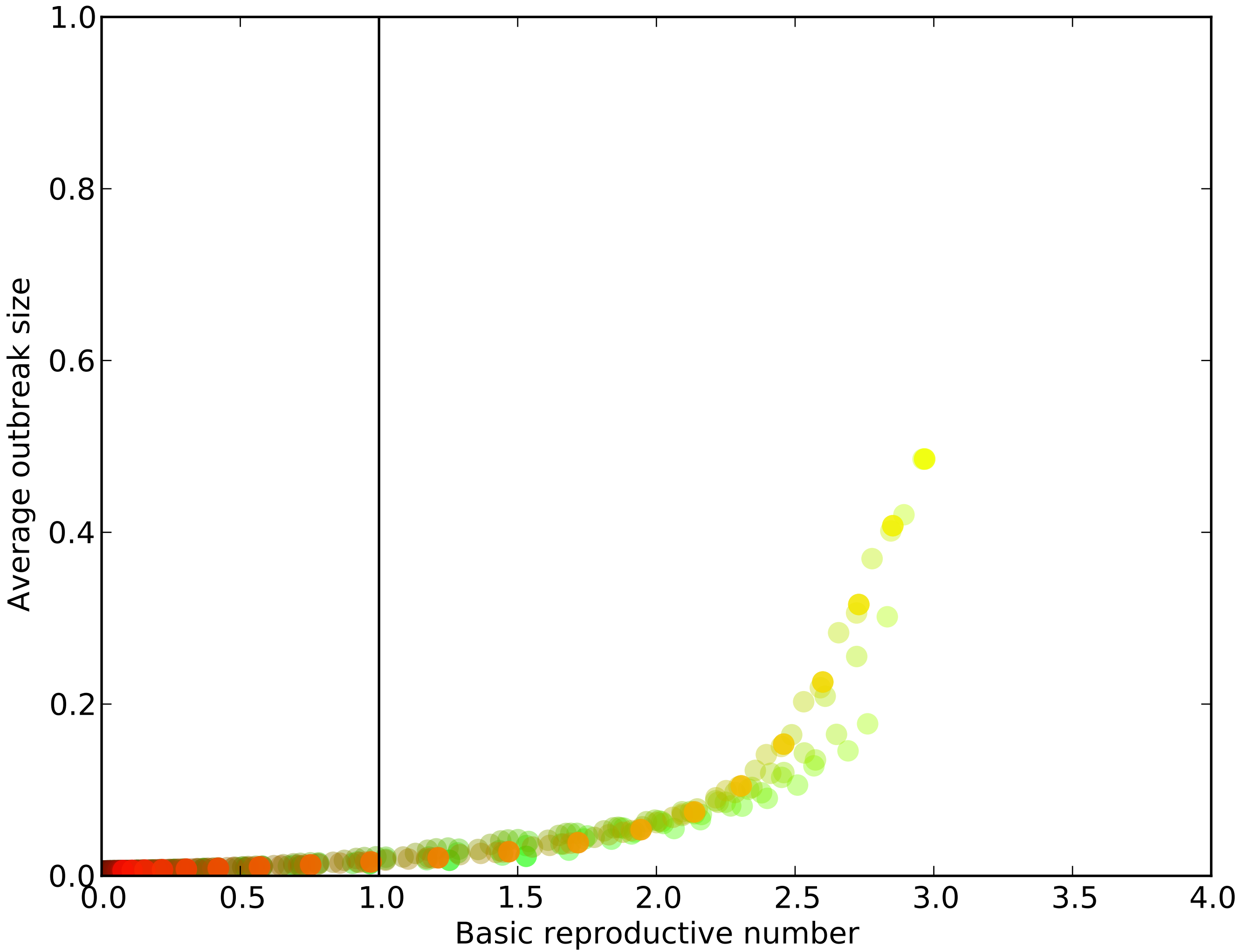

1.0
0.8
0.6
0.4
0.2
0.0
Average outbreak size
0.0
0.5
1.0
1.5
2.0
2.5
3.0
3.5
4.0
Basic reproductive number

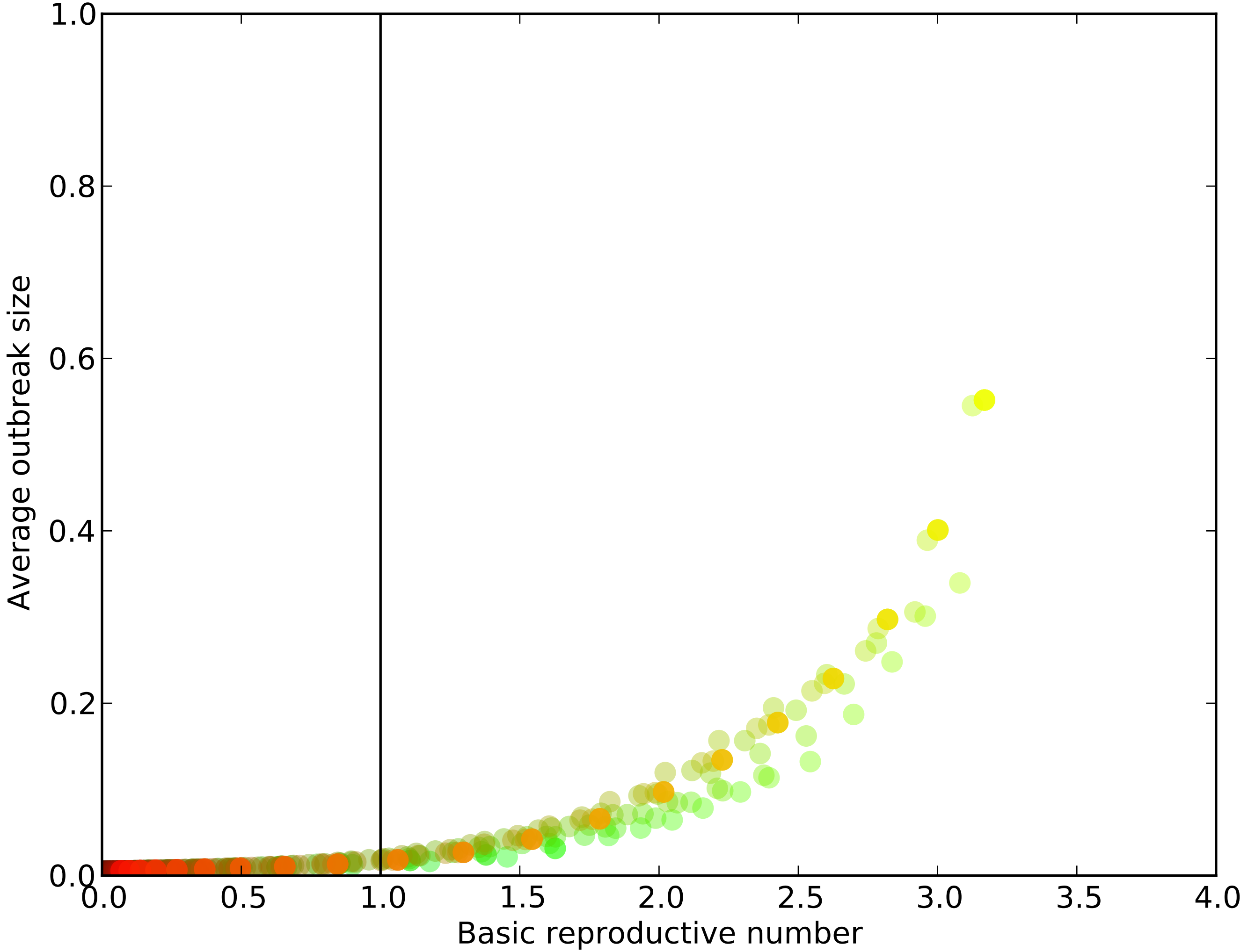

1.0
0.8
0.6
0.4
0.2
0.0
Average outbreak size
0.0
0.5
1.0
1.5
2.0
2.5
3.0
3.5
4.0
Basic reproductive number

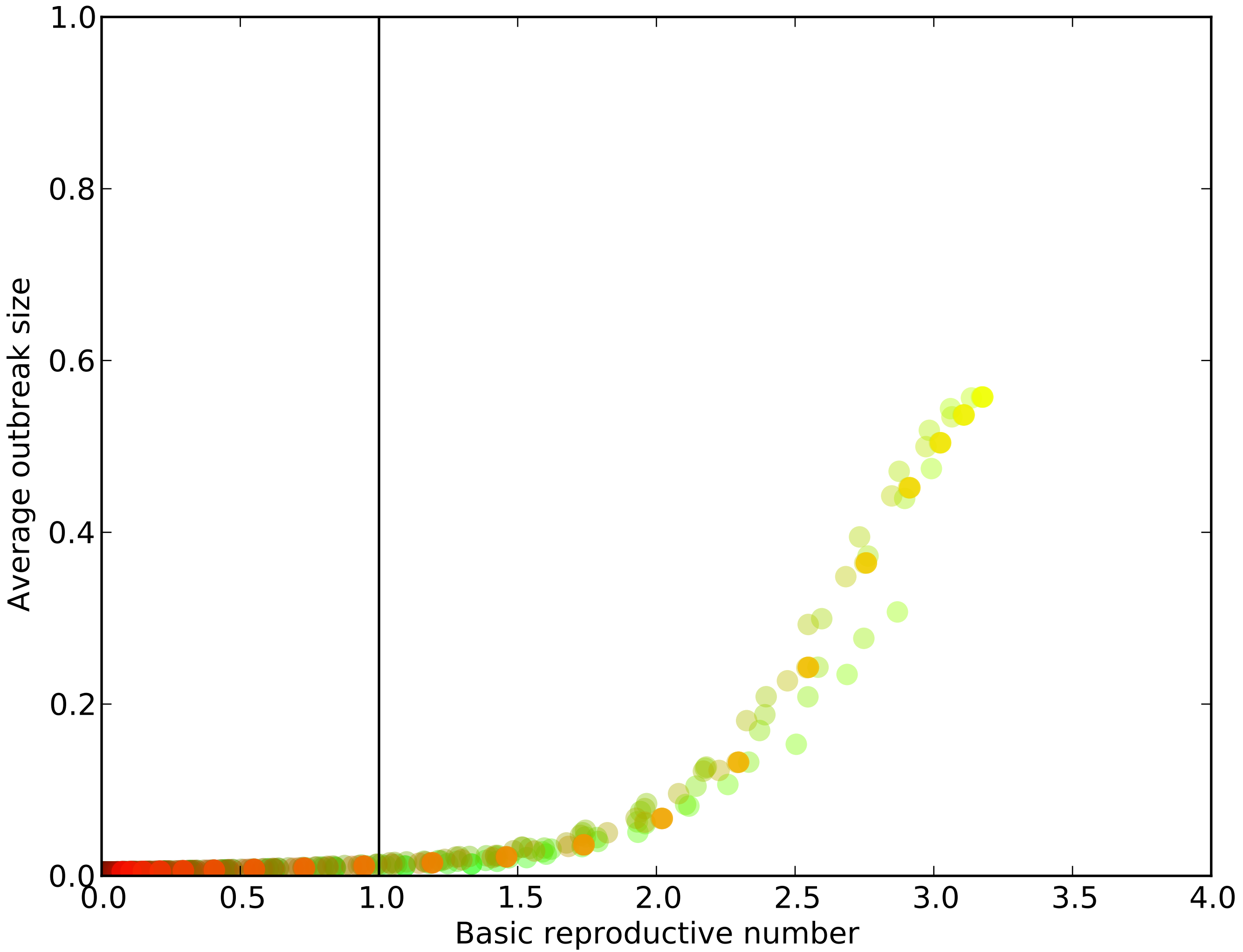

1.0
0.8
0.6
0.4
0.2
0.0
Average outbreak size
0.0
0.5
1.0
1.5
2.0
2.5
3.0
3.5
4.0
Basic reproductive number

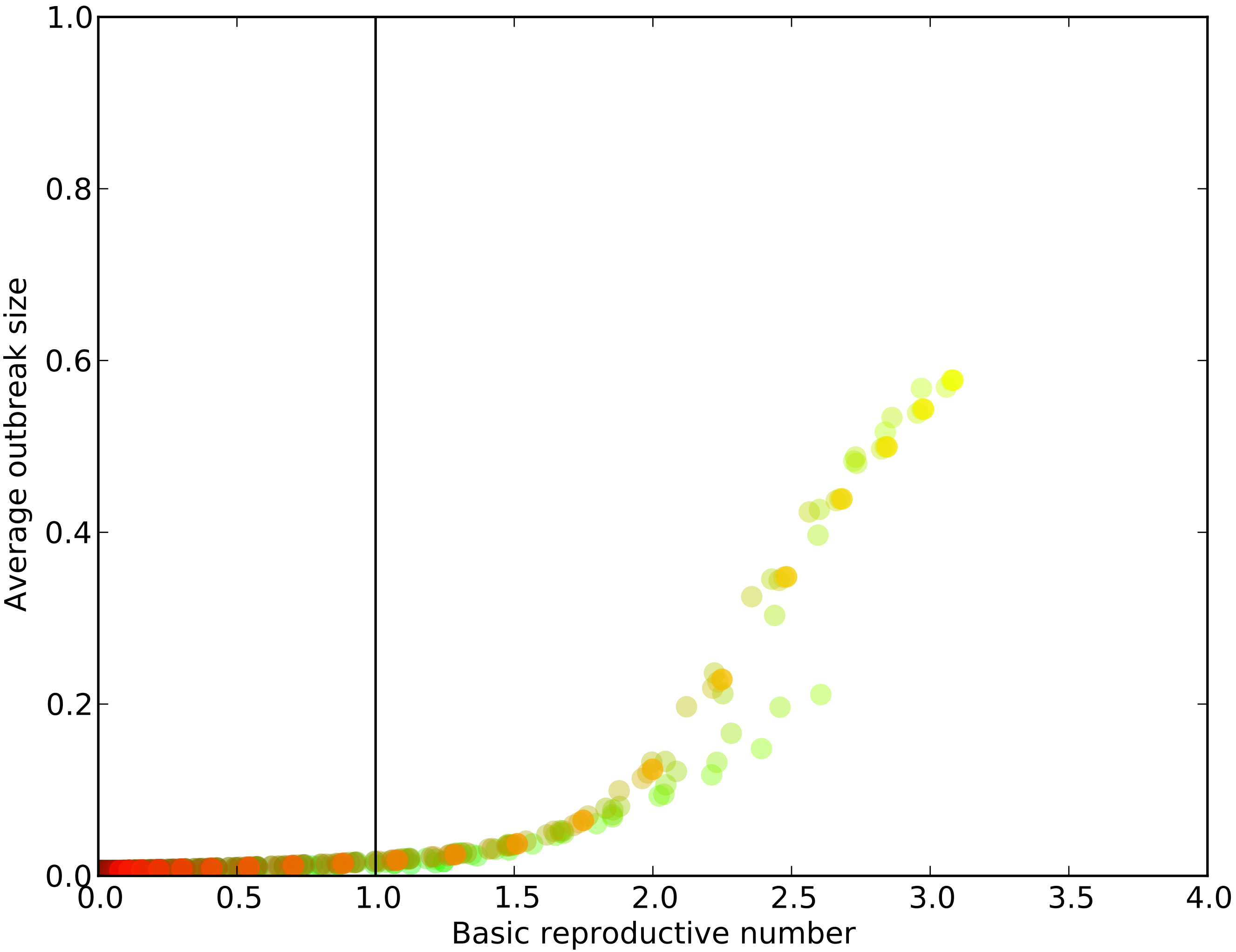

1.0
0.8
0.6
0.4
0.2
0.0
Average outbreak size
0.0
0.5
1.0
1.5
2.0
2.5
3.0
3.5
4.0
Basic reproductive number

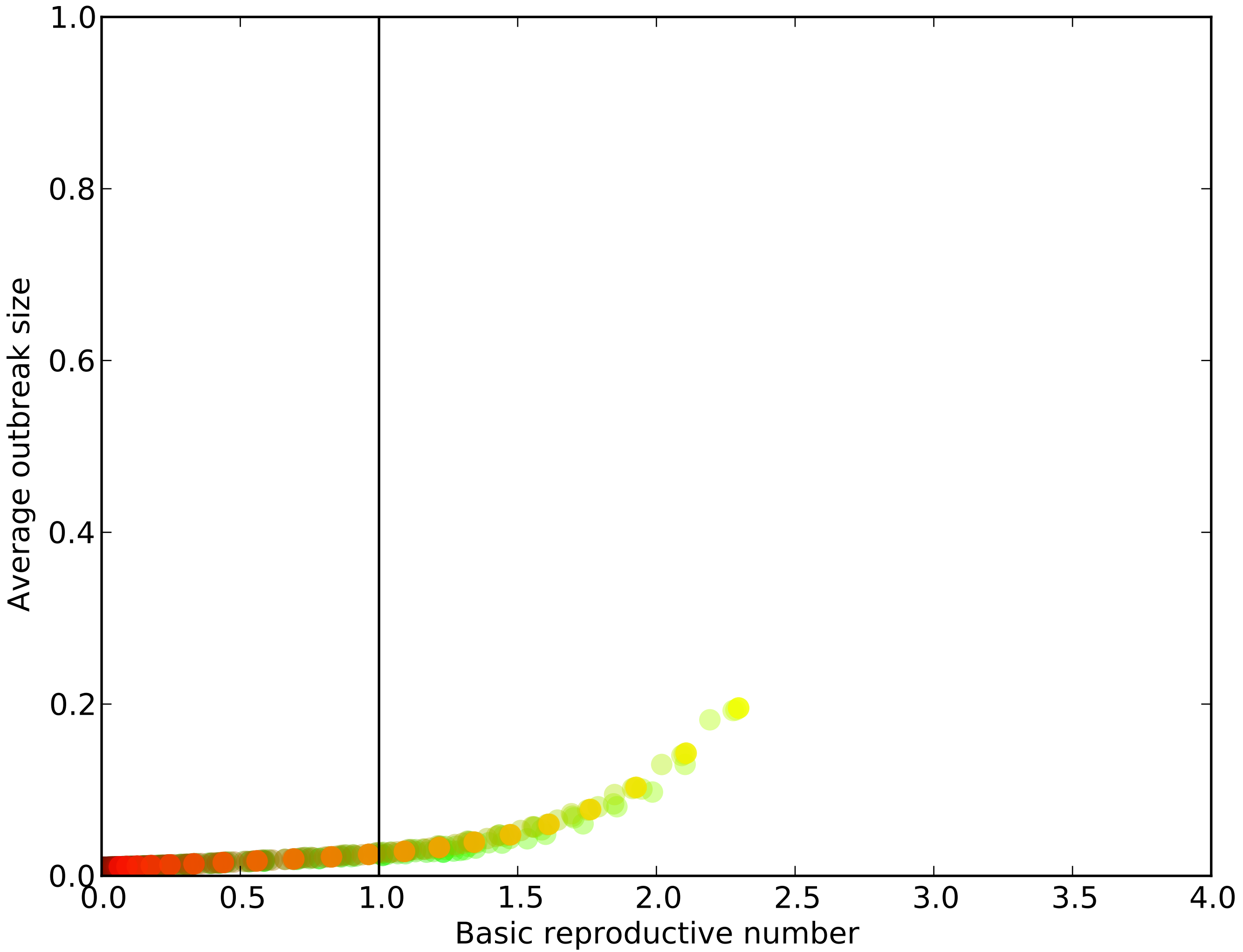

1.0
0.8
0.6
0.4
0.2
0.0
Average outbreak size
0.0
0.5
1.0
1.5
2.0
2.5
3.0
3.5
4.0
Basic reproductive number

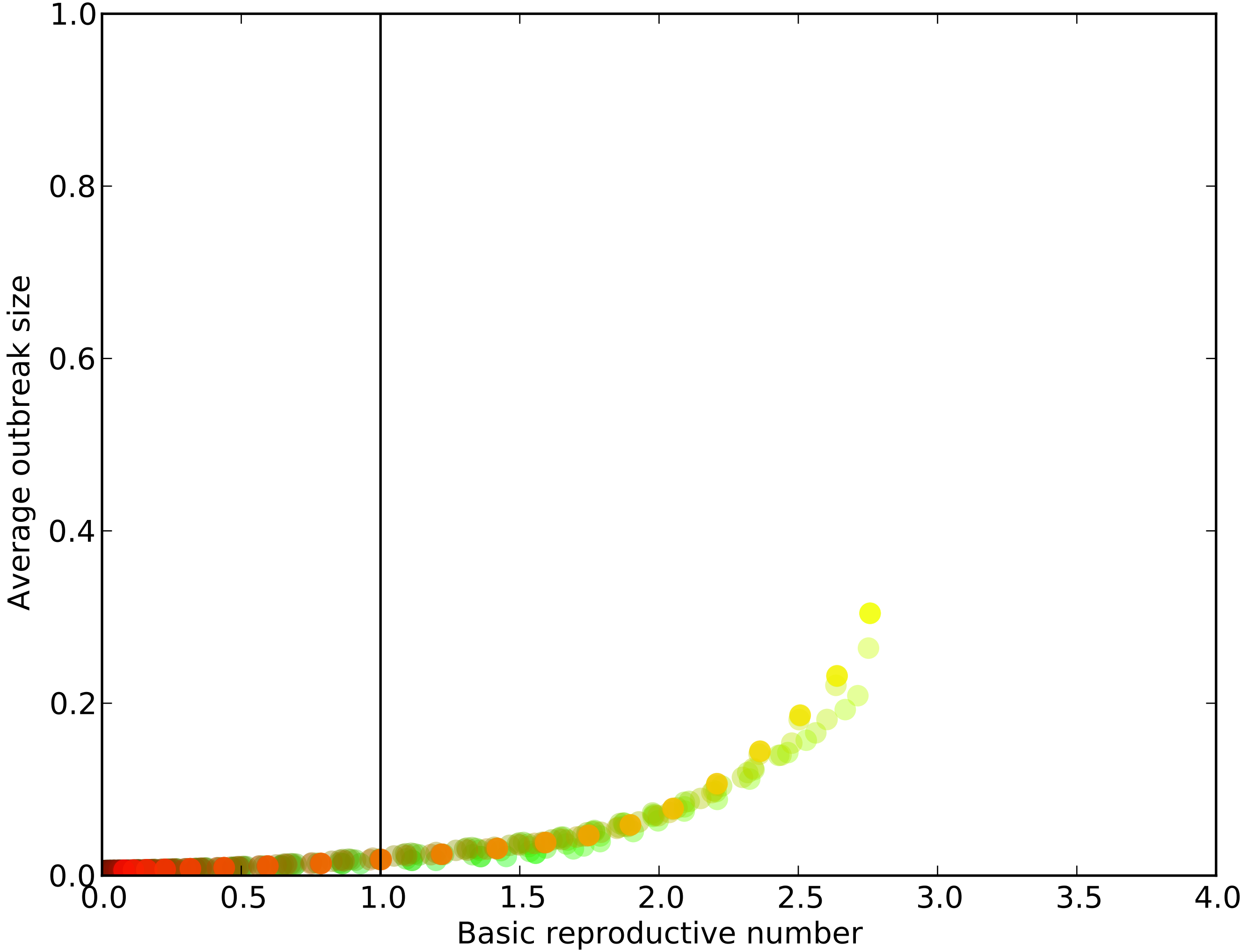

Average outbreak size
Basic reproductive number
0.0
0.2
0.4
0.6
0.8
1.0
0.5
1.5
2.0
2.5
3.0
3.5
4.0

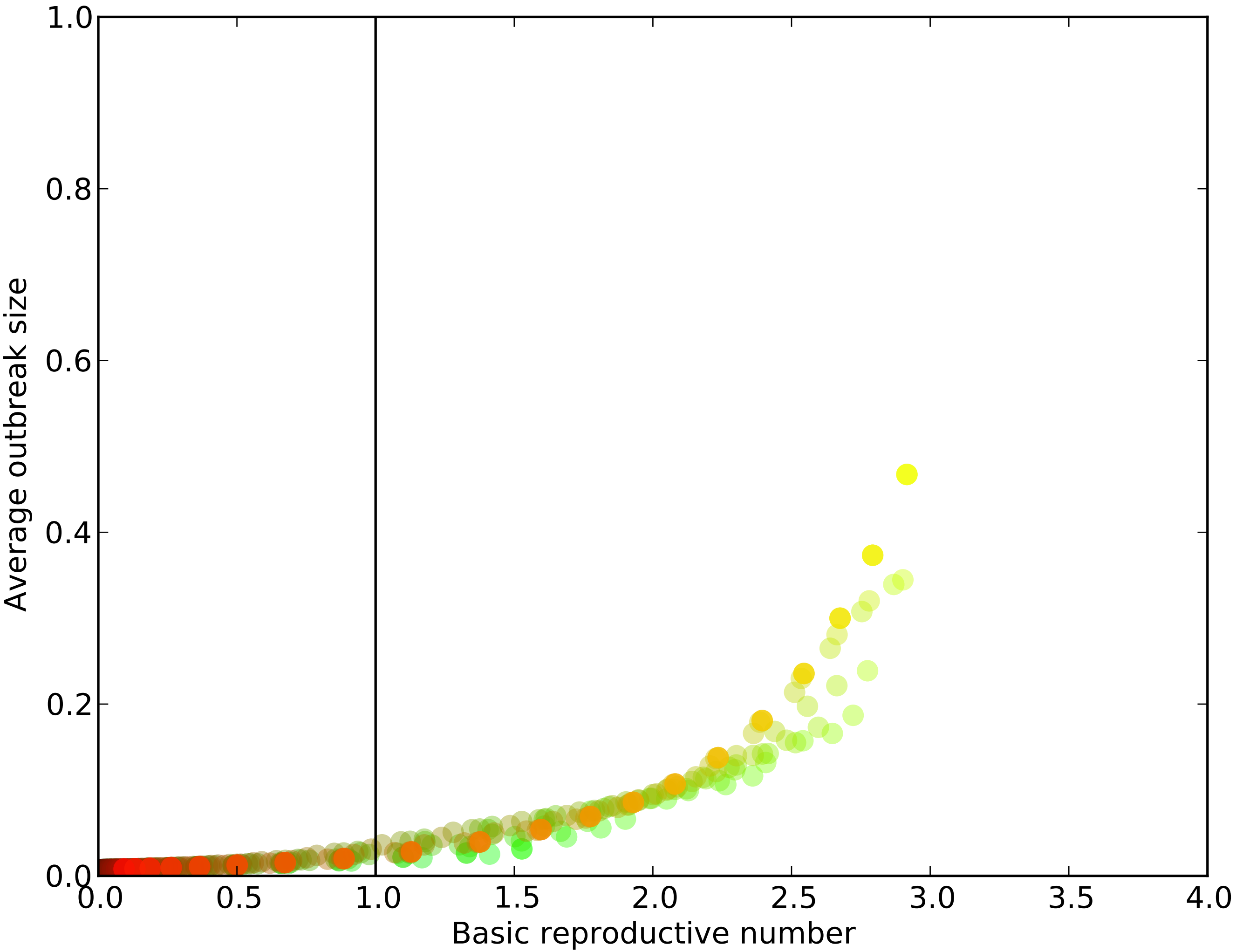

Average outbreak size
Basic reproductive number
1.0
0.8
0.6
0.4
0.2
0.0
0.0
0.5
1.0
1.5
2.0
2.5
3.0
3.5
4.0

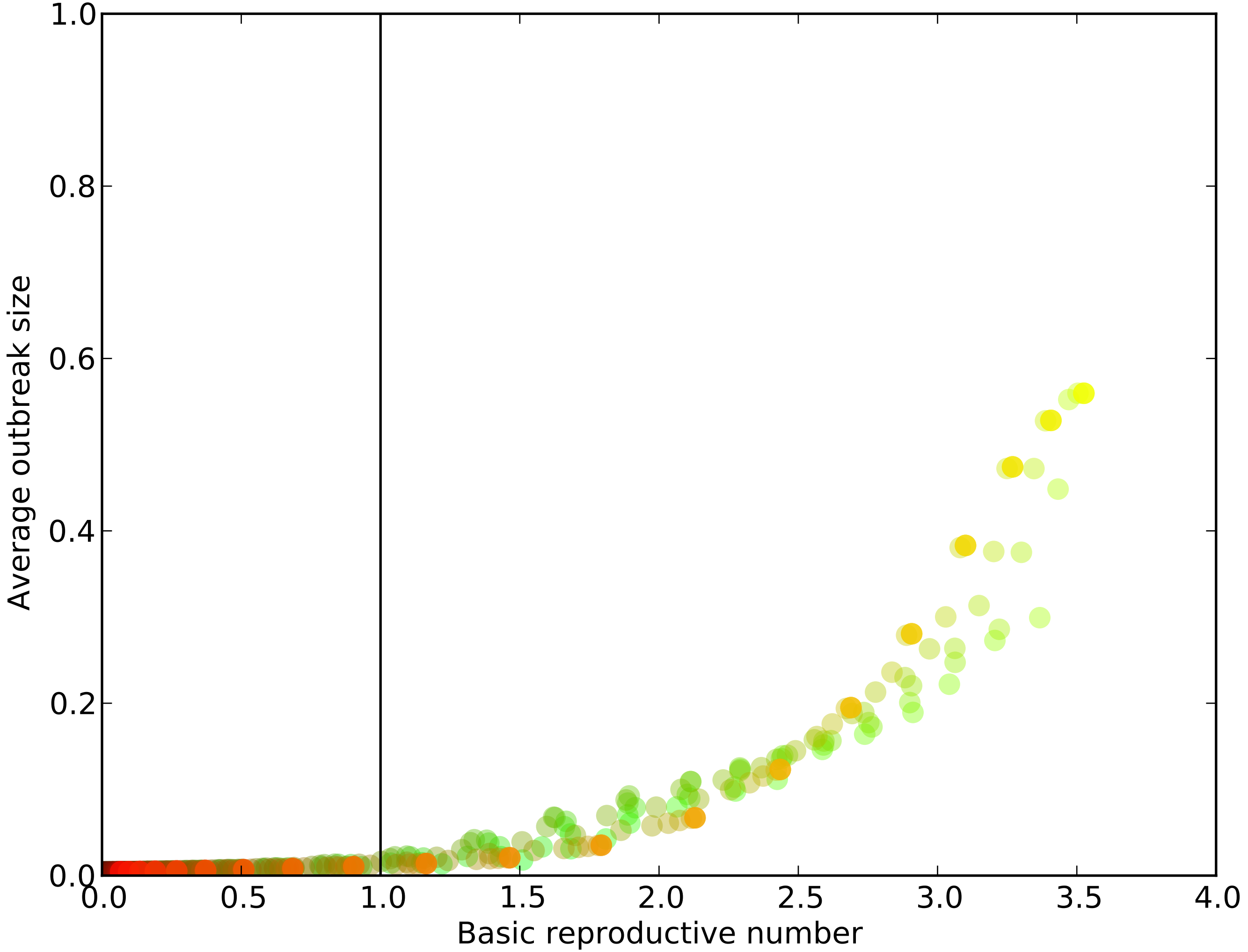

1.0
0.8
0.6
0.4
0.2
0.0
Average outbreak size
0.0
0.5
1.0
1.5
2.0
2.5
3.0
3.5
4.0
Basic reproductive number

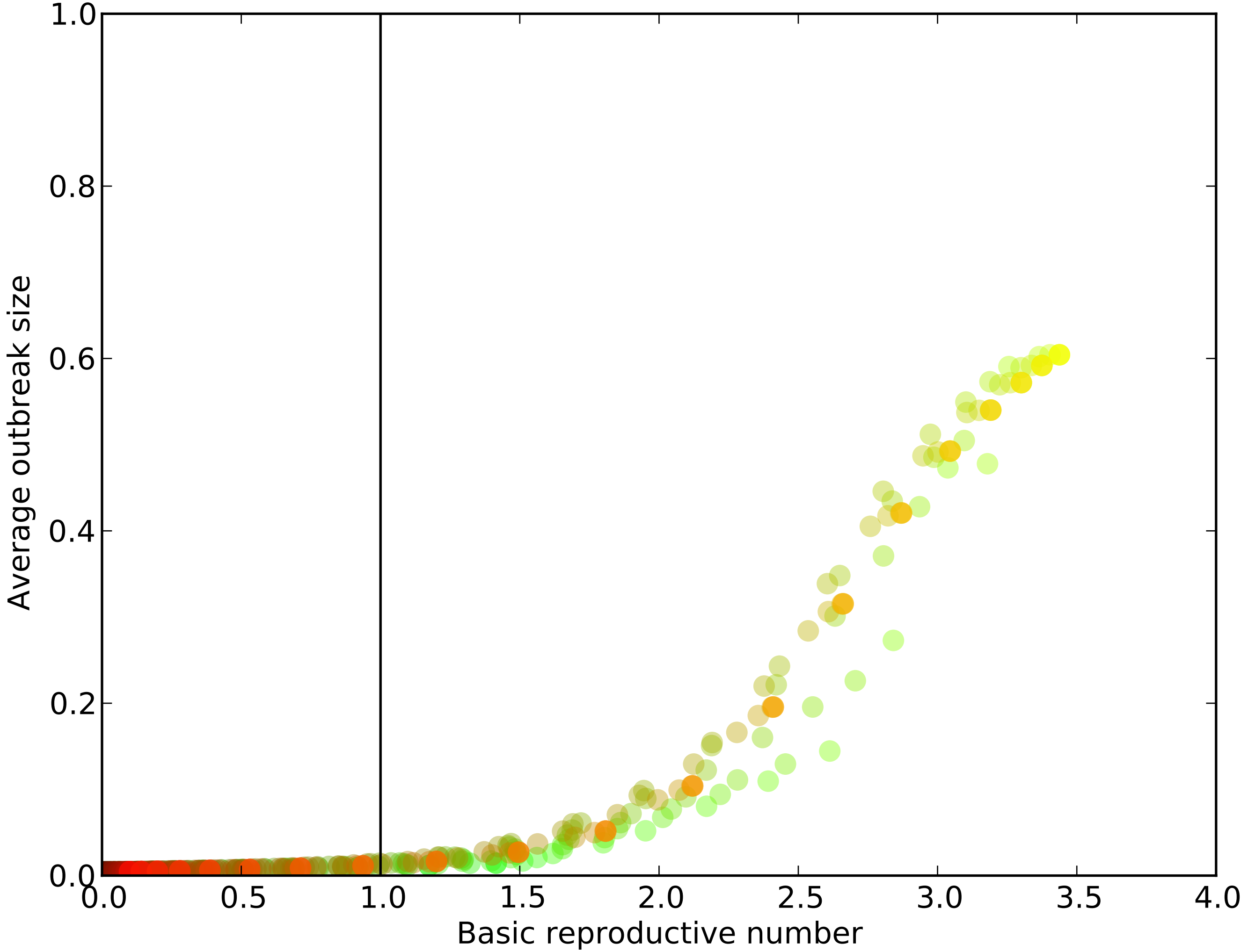

1.0
0.8
0.6
0.4
0.2
0.0
Average outbreak size
0.0
0.5
1.0
1.5
2.0
2.5
3.0
3.5
4.0
Basic reproductive number

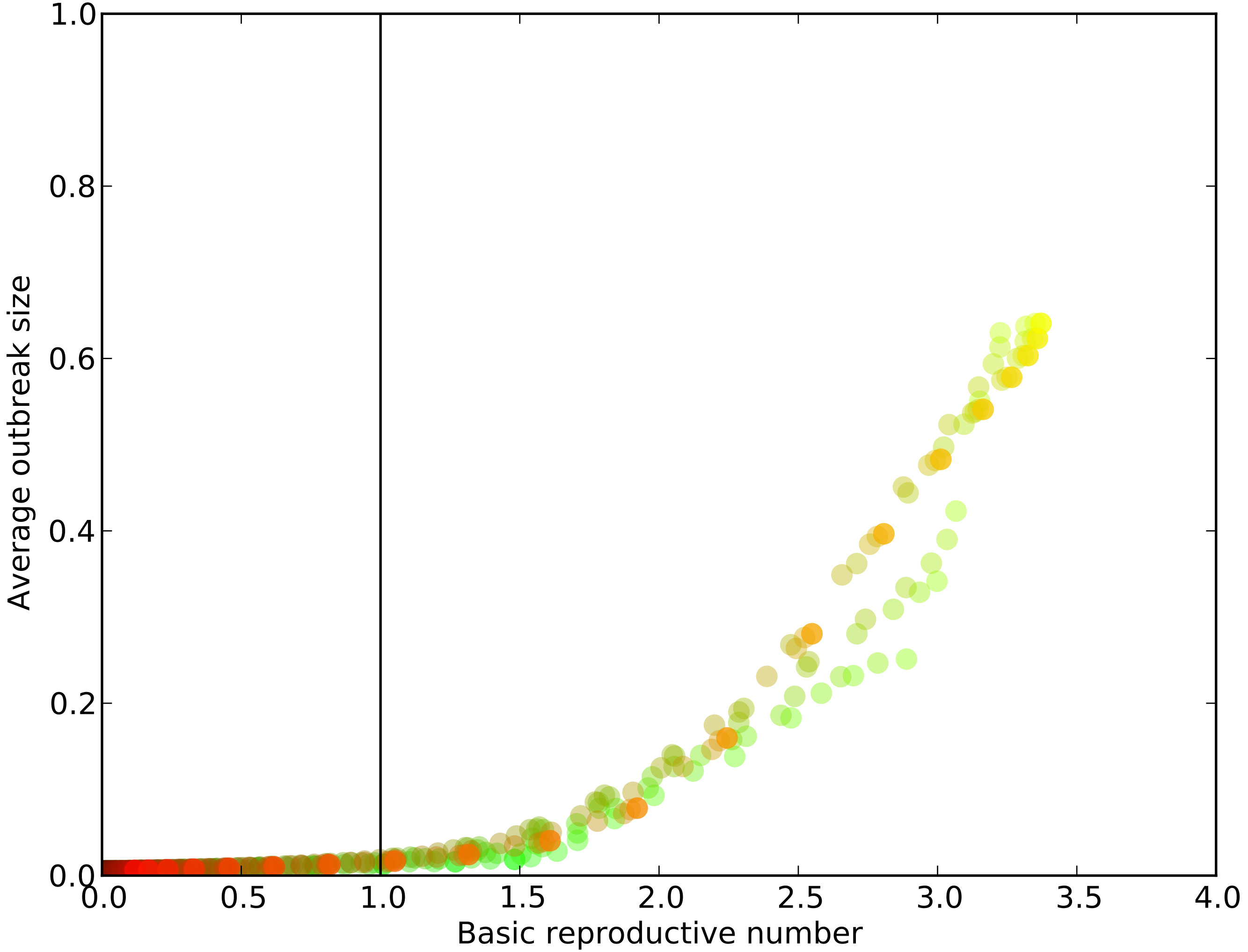
Average outbreak size
Basic reproductive number
0.0
0.2
0.4
0.6
0.8
1.0
0.0
0.5
1.0
1.5
2.0
2.5
3.0
3.5
4.0

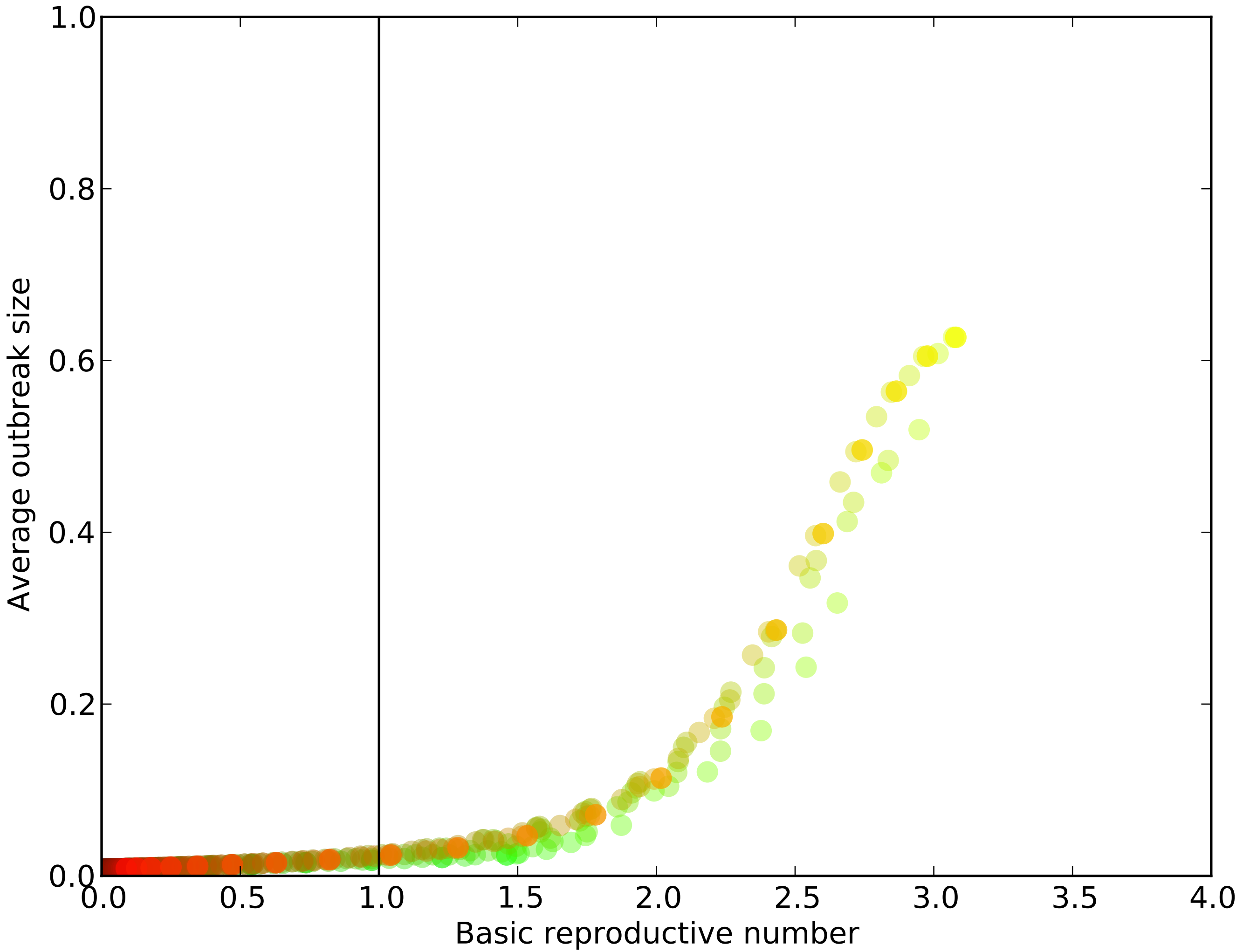

1.0
0.8
0.6
0.4
0.2
0.0
Average outbreak size
0.0
0.5
1.0
1.5
2.0
2.5
3.0
3.5
4.0
Basic reproductive number

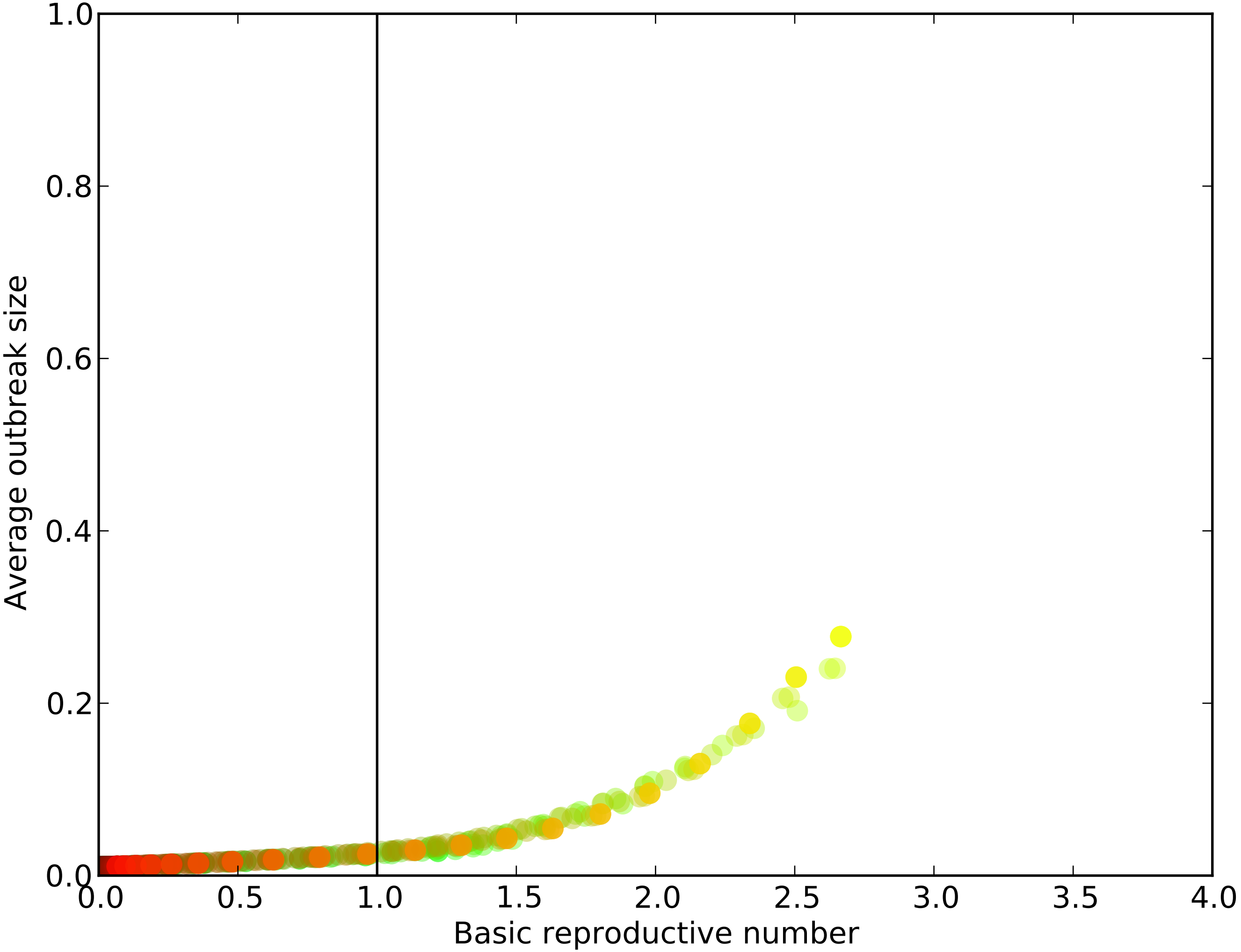

Average outbreak size
Basic reproductive number
1.0
0.8
0.6
0.4
0.2
0.0
0.0
0.5
1.0
1.5
2.0
2.5
3.0
3.5
4.0

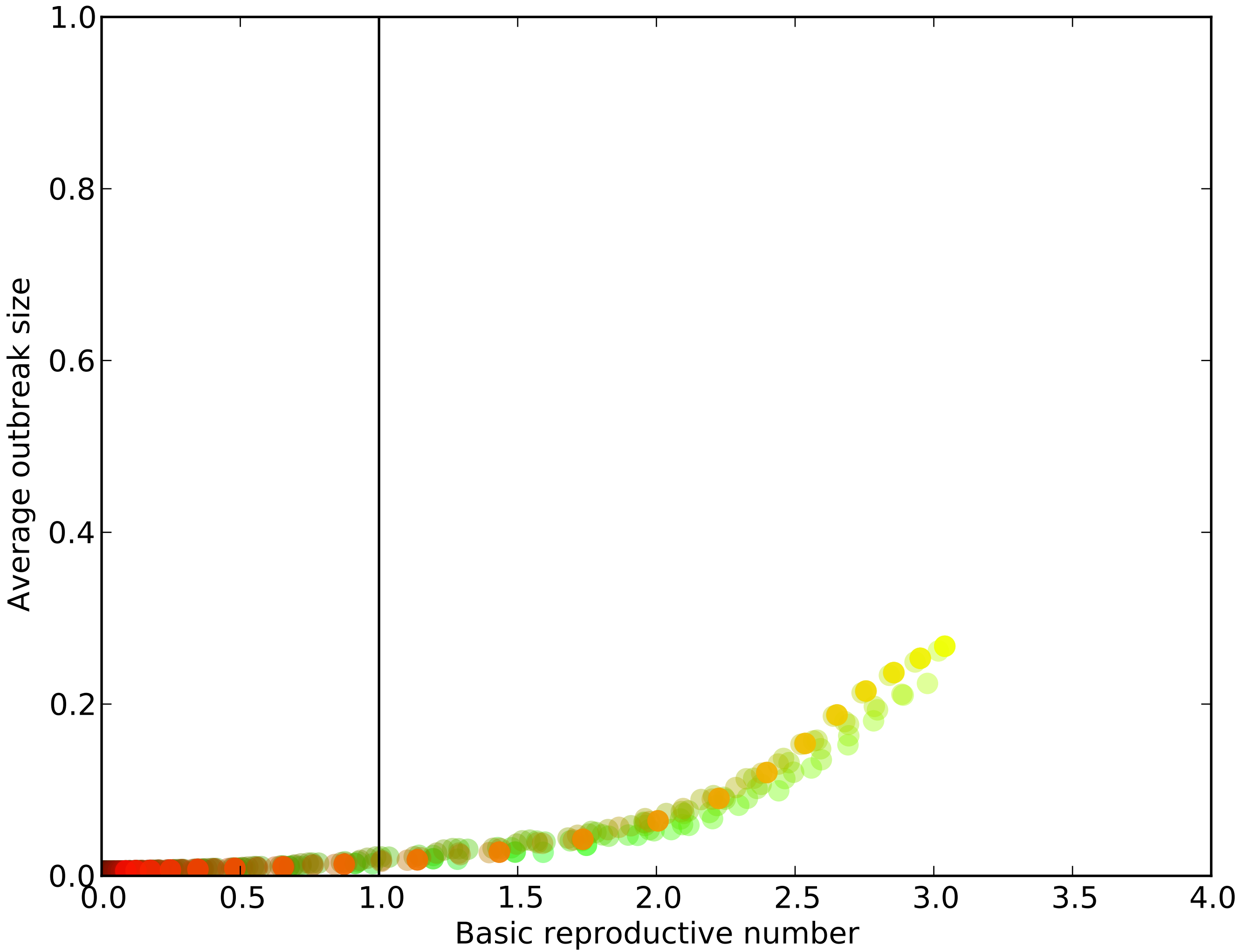
Average outbreak size
Basic reproductive number
0.0
0.2
0.4
0.6
0.8
1.0
0.5
1.0
1.5
2.0
2.5
3.0
3.5
4.0

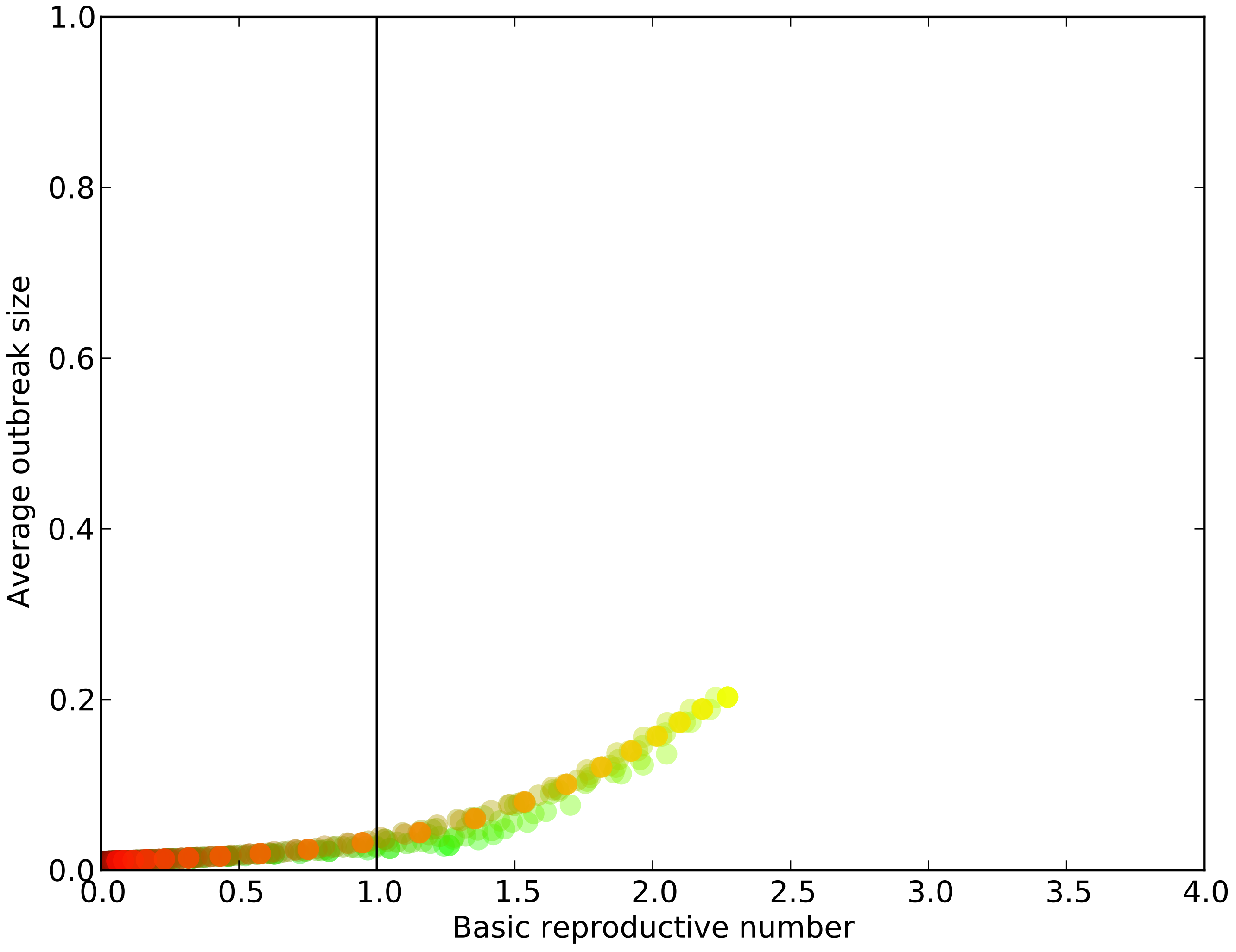

1.0
0.8
0.6
0.4
0.2
0.0
Average outbreak size
0.0
0.5
1.0
1.5
2.0
2.5
3.0
3.5
4.0
Basic reproductive number

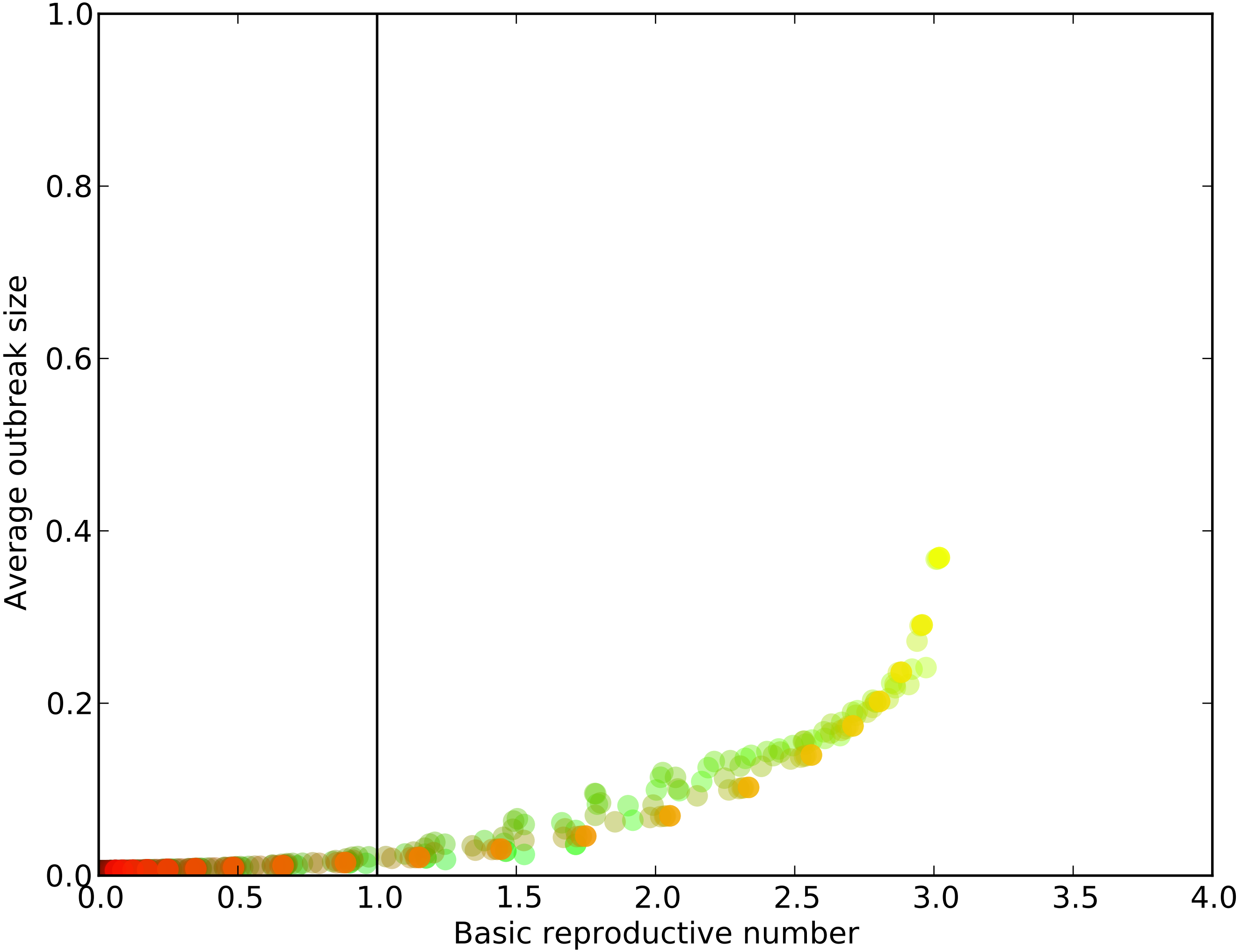

1.0
0.8
0.6
0.4
0.2
0.0
Average outbreak size
0.0
0.5
1.0
1.5
2.0
2.5
3.0
3.5
4.0
Basic reproductive number

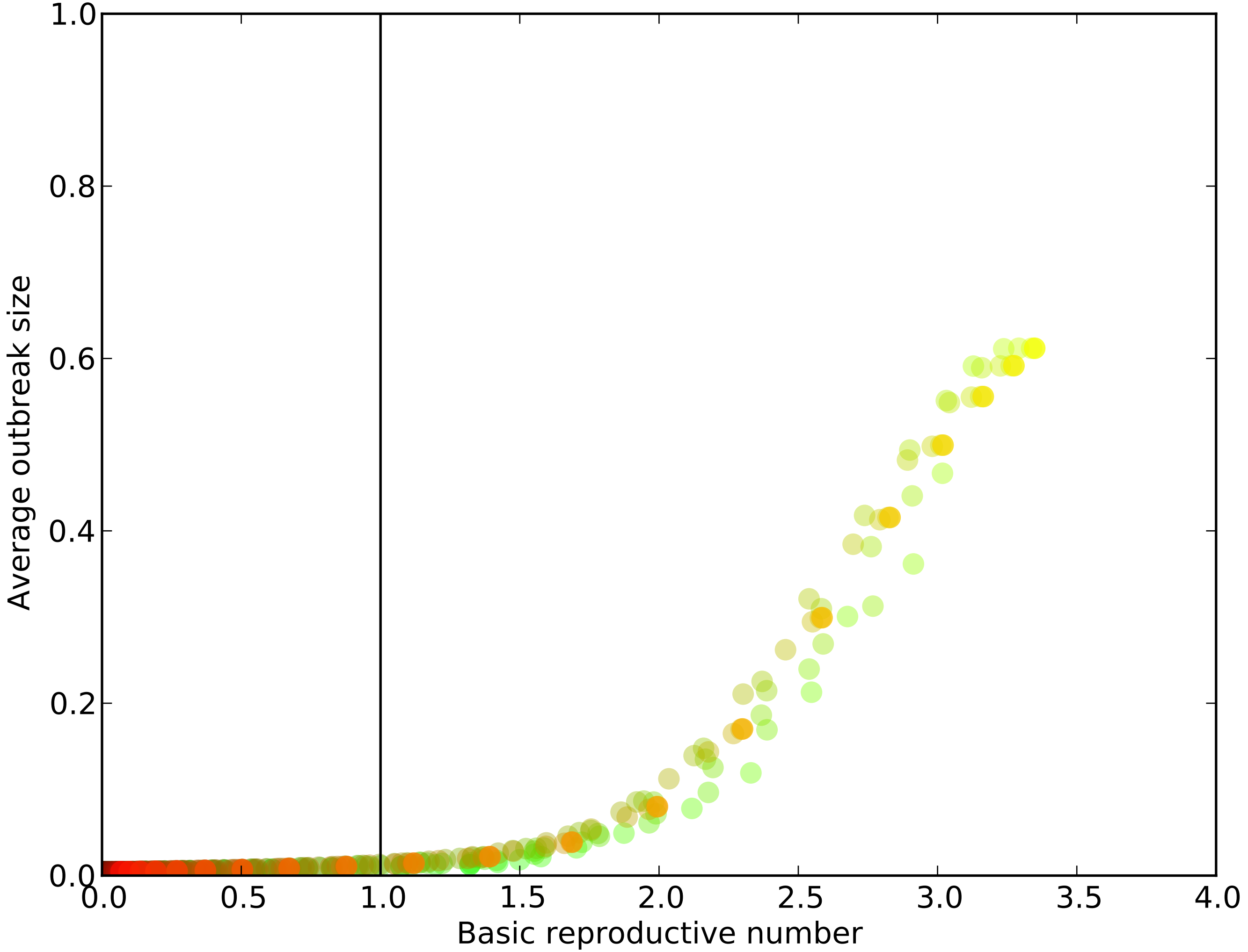
Average outbreak size
Basic reproductive number
0.0
0.2
0.4
0.6
0.8
1.0
0.0
0.5
1.0
1.5
2.0
2.5
3.0
3.5
4.0

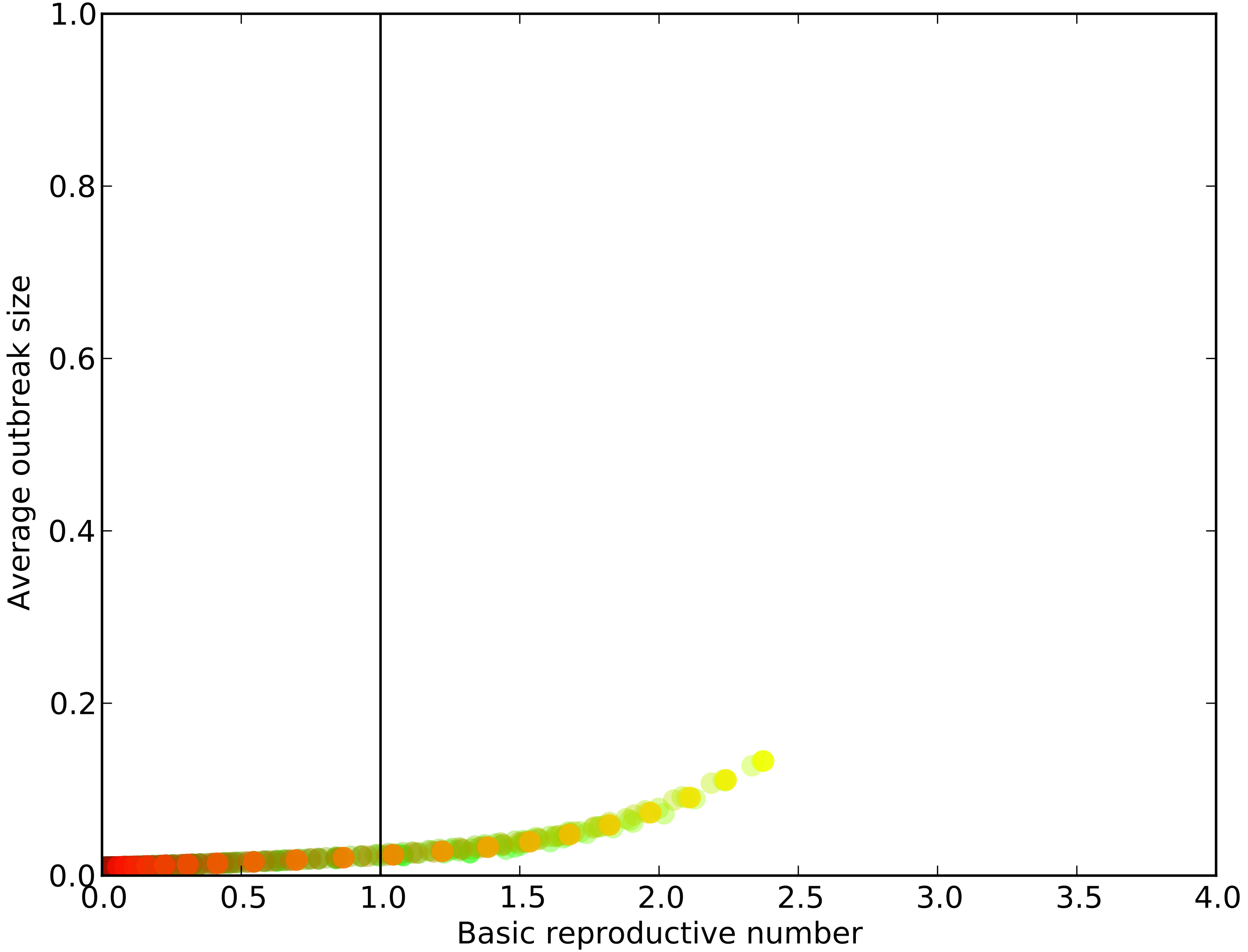

Average outbreak size
1.0
0.8
0.6
0.4
0.2
0.0
0.0
0.5
1.0
1.5
2.0
2.5
3.0
3.5
4.0
Basic reproductive number

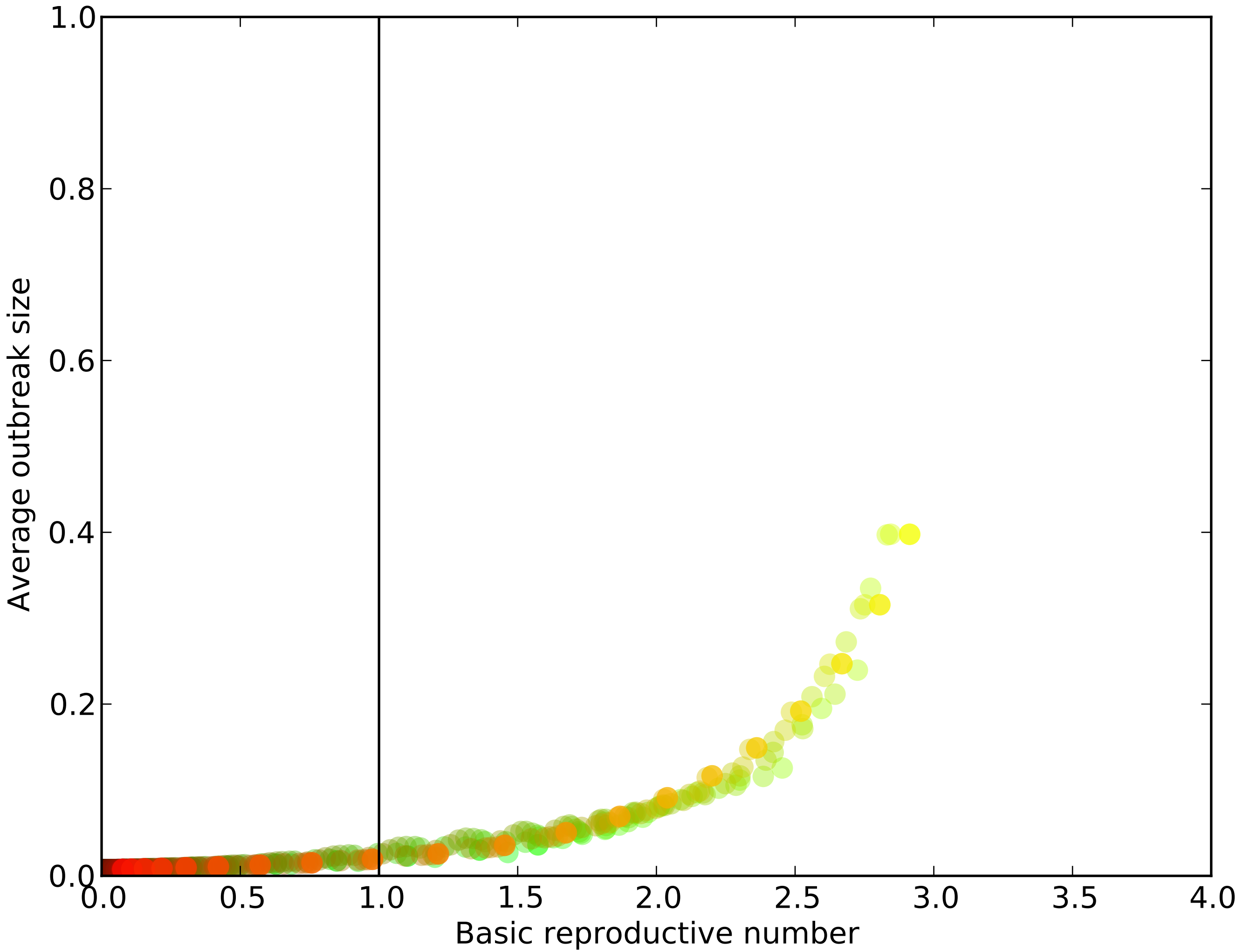

1.0
0.8
0.6
0.4
0.2
0.0
0.0
0.5
1.0
1.5
2.0
2.5
3.0
3.5
4.0
Average outbreak size
Basic reproductive number

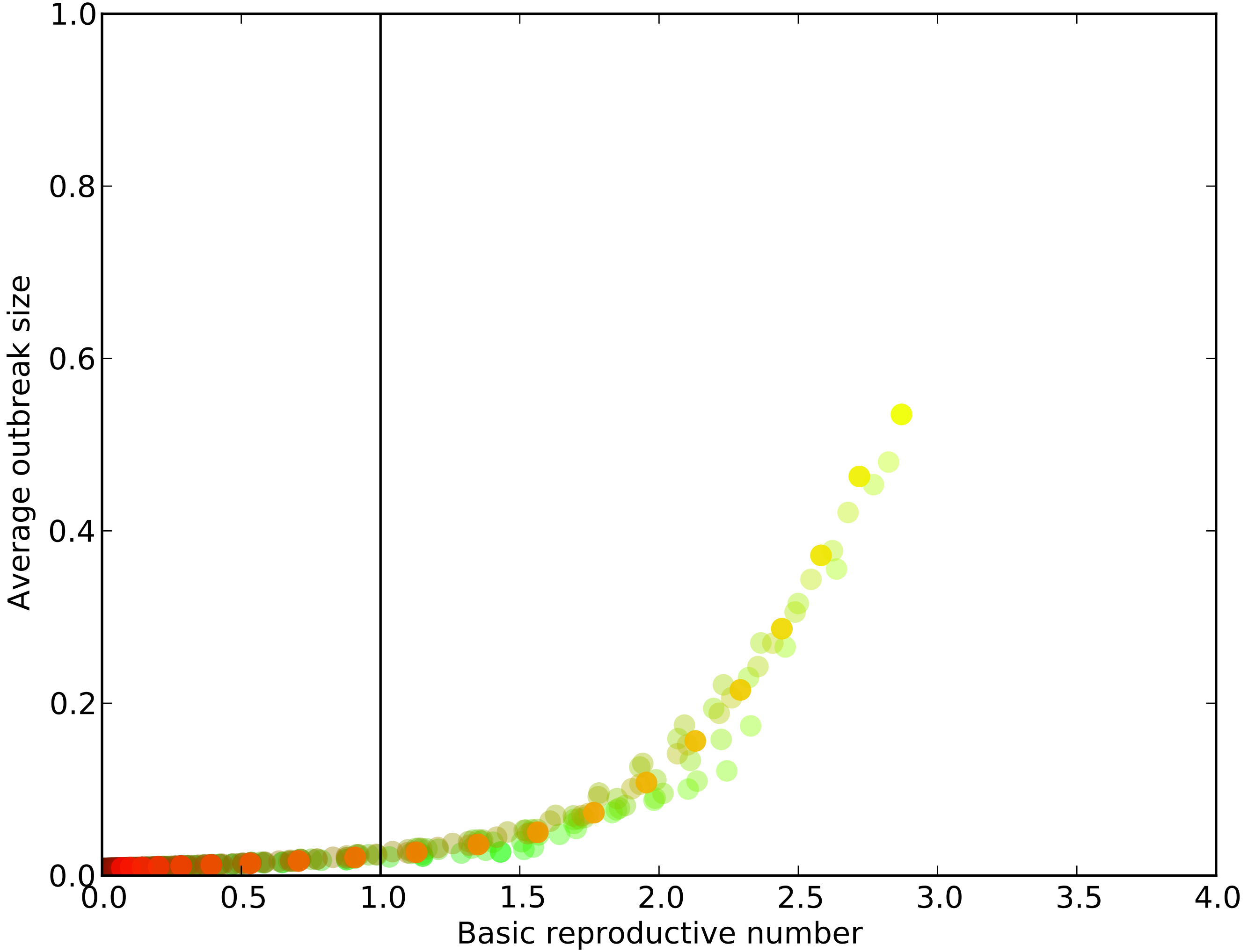

1.0
0.8
0.6
0.4
0.2
0.0
Average outbreak size
0.0
0.5
1.0
1.5
2.0
2.5
3.0
3.5
4.0
Basic reproductive number

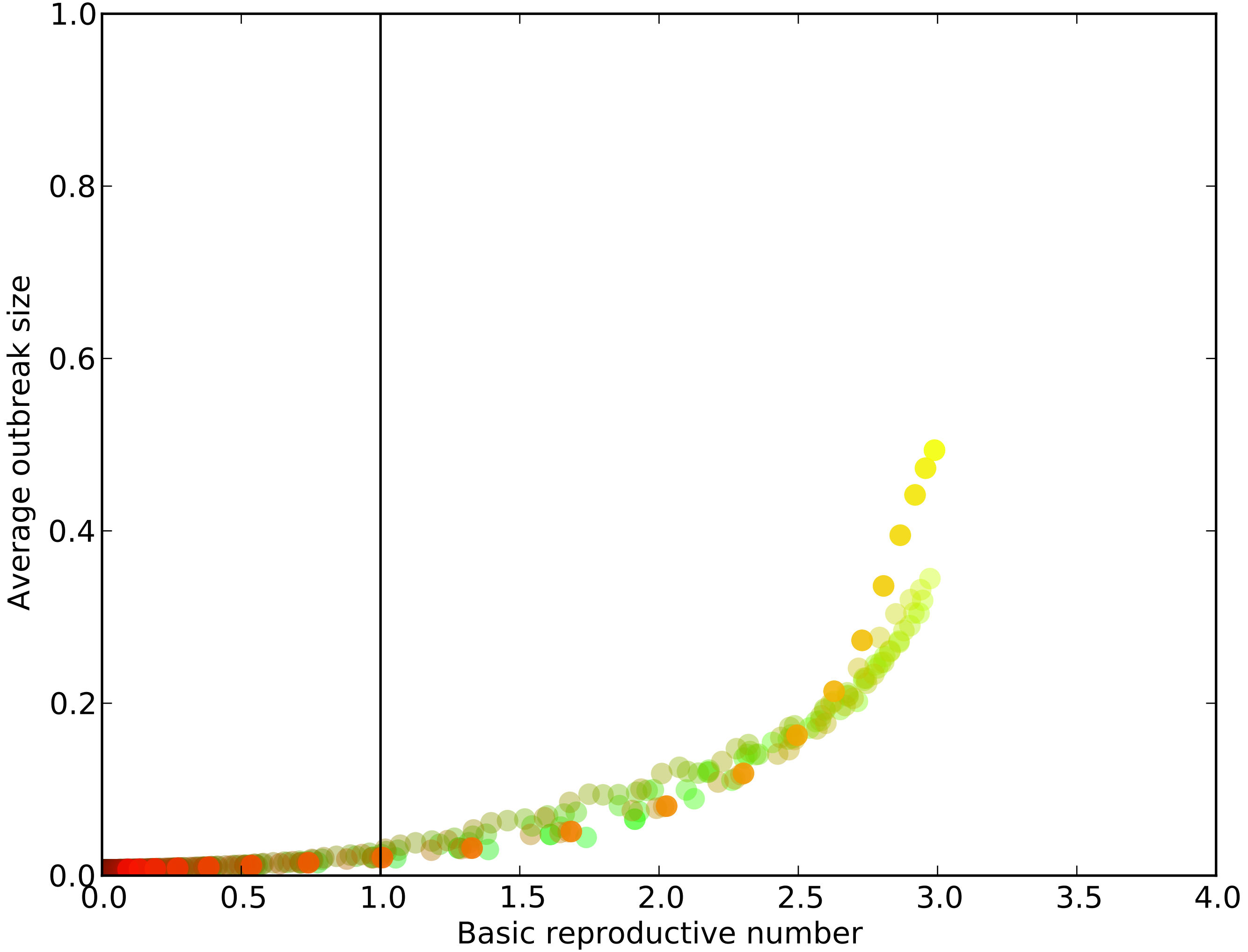

1.0
0.8
0.6
0.4
0.2
0.0
0.0
0.5
1.0
1.5
2.0
2.5
3.0
3.5
4.0
Basic reproductive number
Average outbreak size

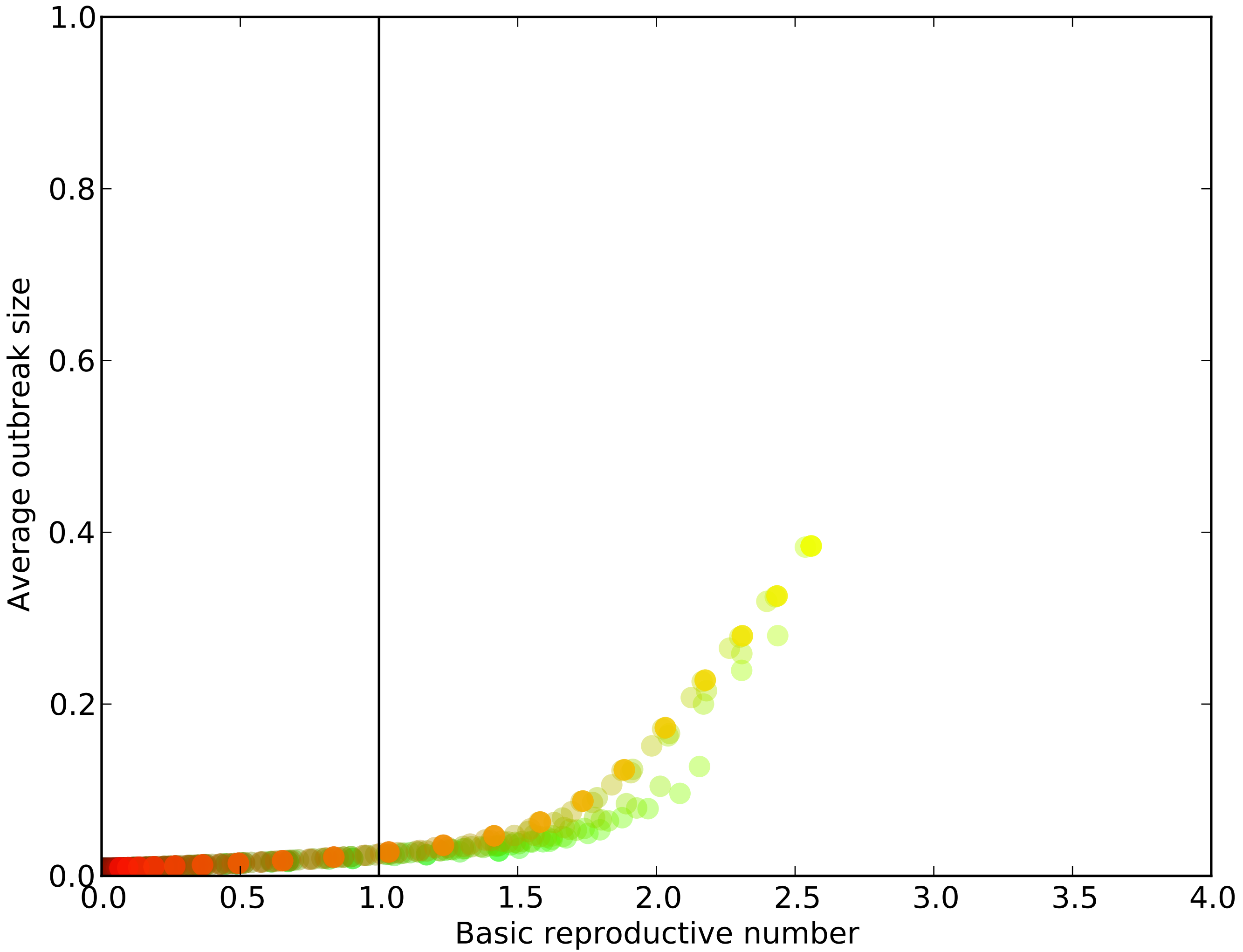

Average outbreak size
1.0
0.8
0.6
0.4
0.2
0.0
0.0
0.5
1.0
1.5
2.0
2.5
3.0
3.5
4.0
Basic reproductive number

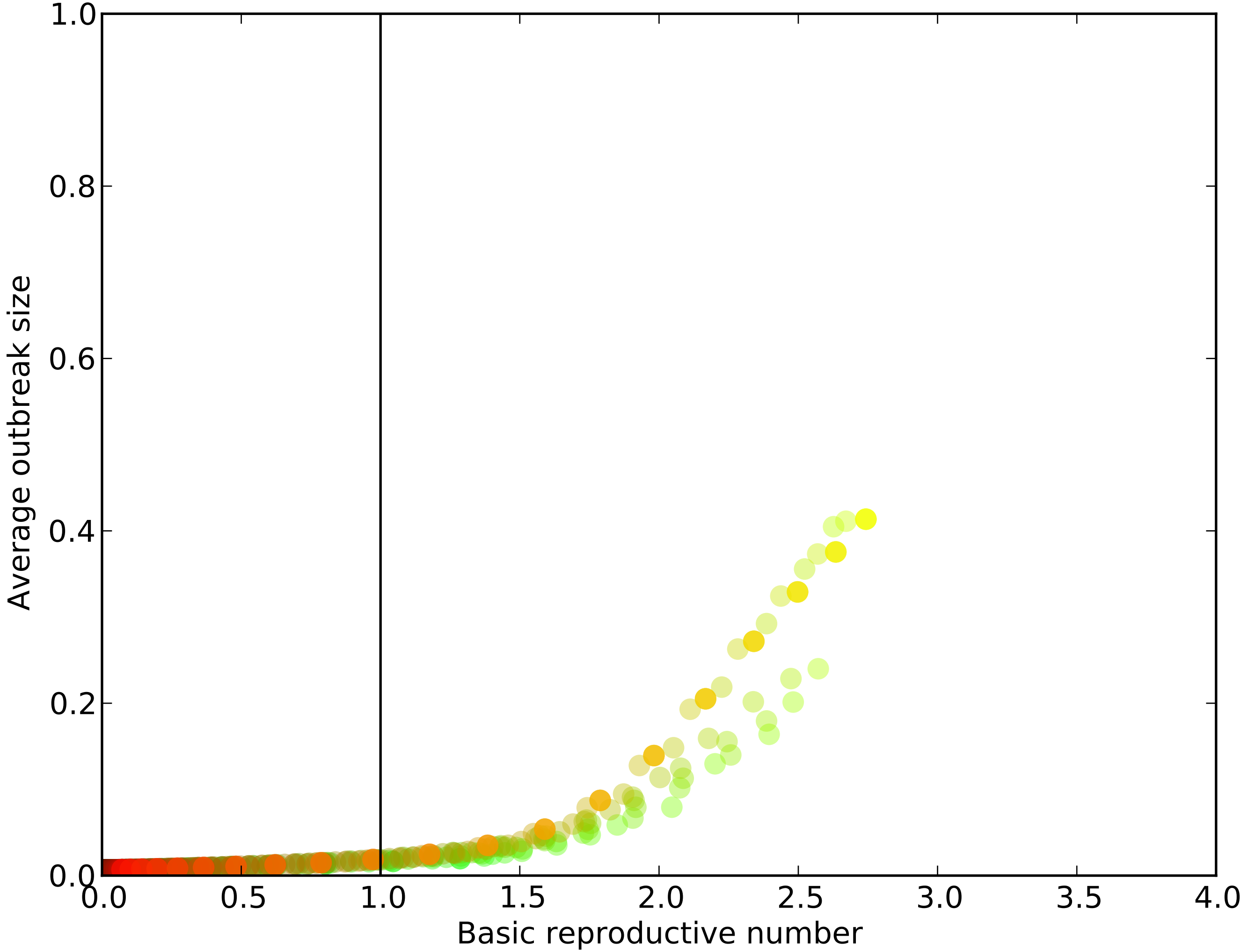

1.0
0.8
0.6
0.4
0.2
0.0
Average outbreak size
0.0
0.5
1.0
1.5
2.0
2.5
3.0
3.5
4.0
Basic reproductive number

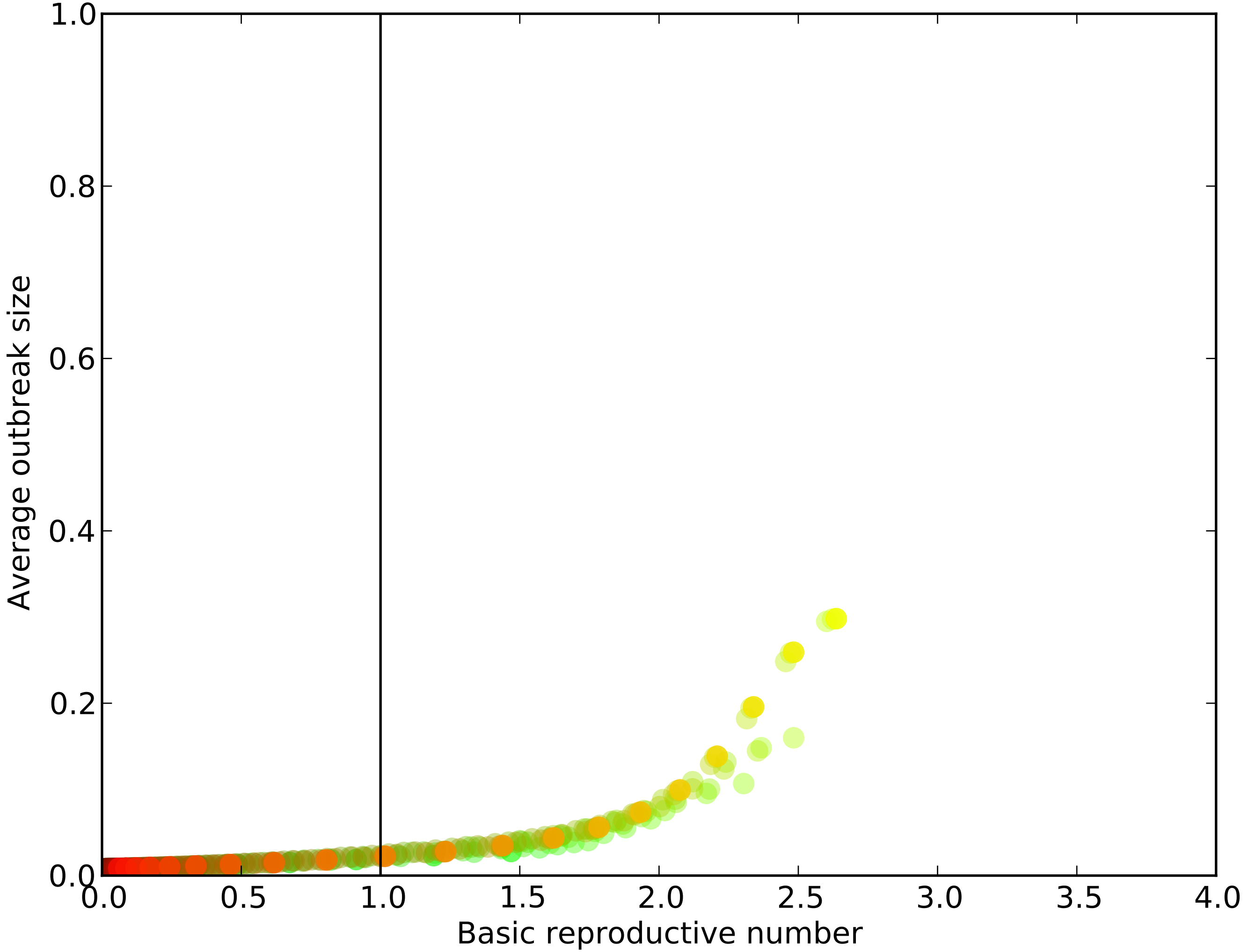

1.0
0.8
0.6
0.4
0.2
0.0
Average outbreak size
0.0
0.5
1.0
1.5
2.0
2.5
3.0
3.5
4.0
Basic reproductive number

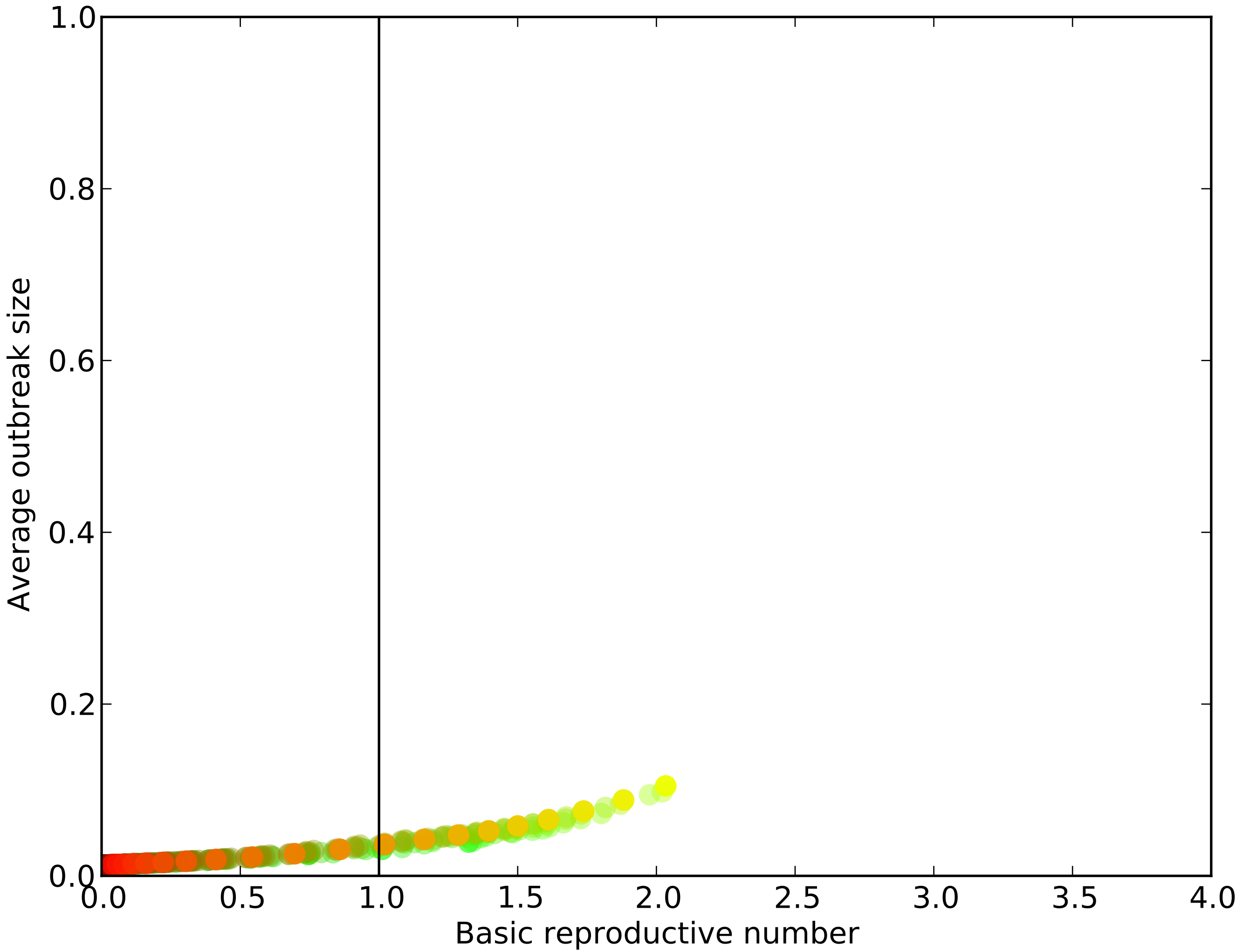
1.0
0.8
0.6
0.4
0.2
0.0
Average outbreak size
0.0
0.5
1.0
1.5
2.0
2.5
3.0
3.5
4.0
Basic reproductive number

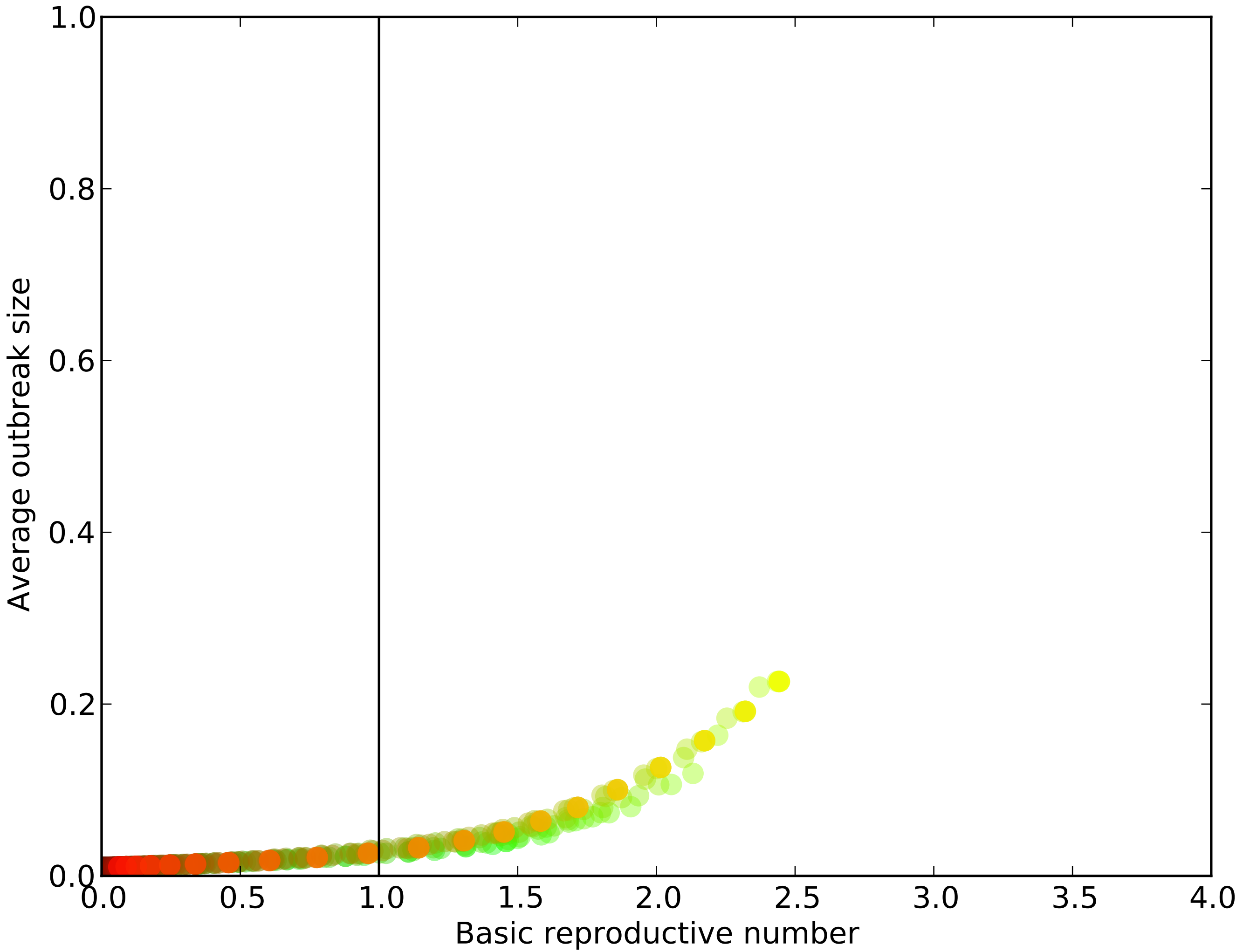

1.0
0.8
0.6
0.4
0.2
0.0
0.0
0.5
1.0
1.5
2.0
2.5
3.0
3.5
4.0
Average outbreak size
Basic reproductive number

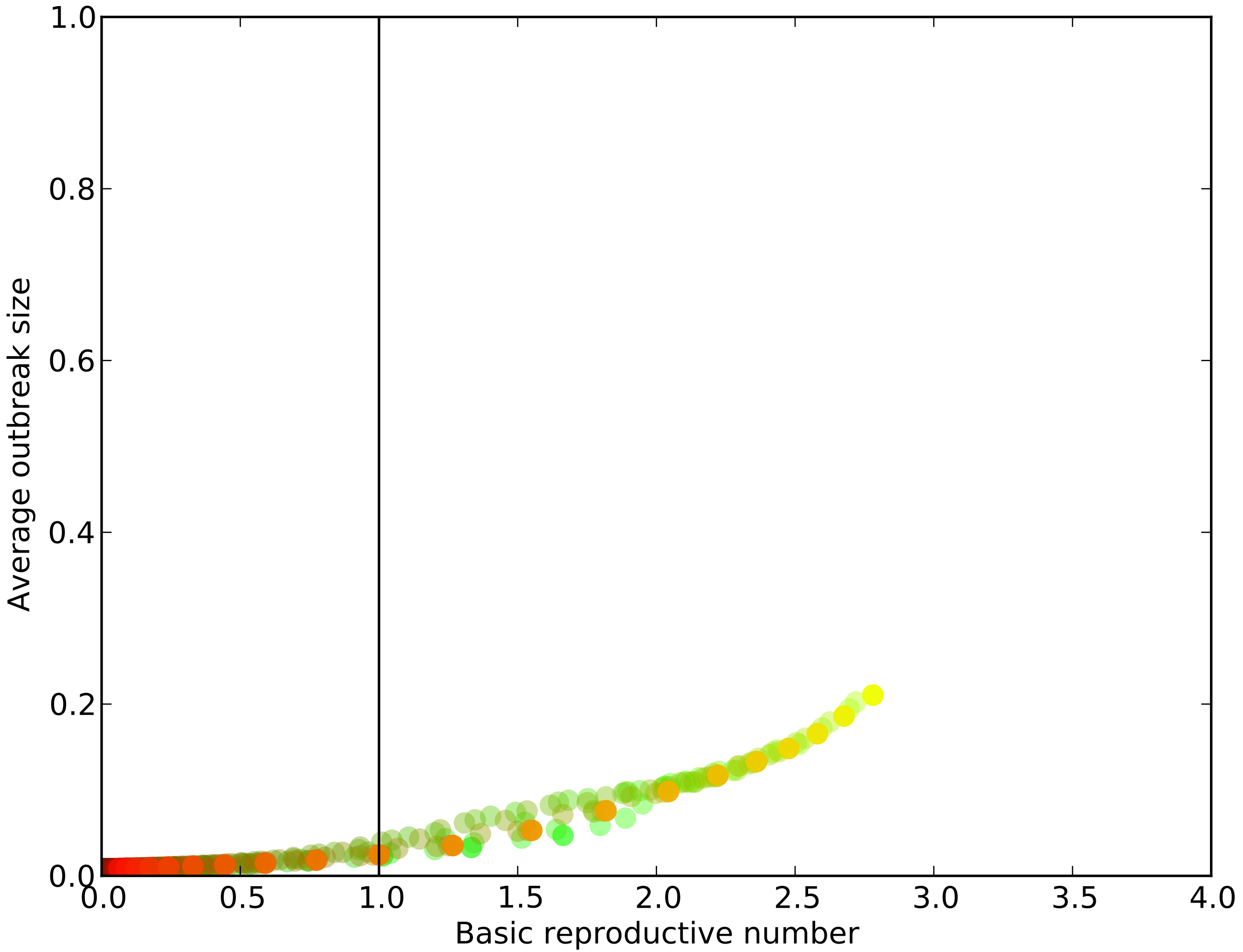

1.0
0.8
0.6
0.4
0.2
0.0
Average outbreak size
0.0
0.5
1.0
1.5
2.0
2.5
3.0
3.5
4.0
Basic reproductive number

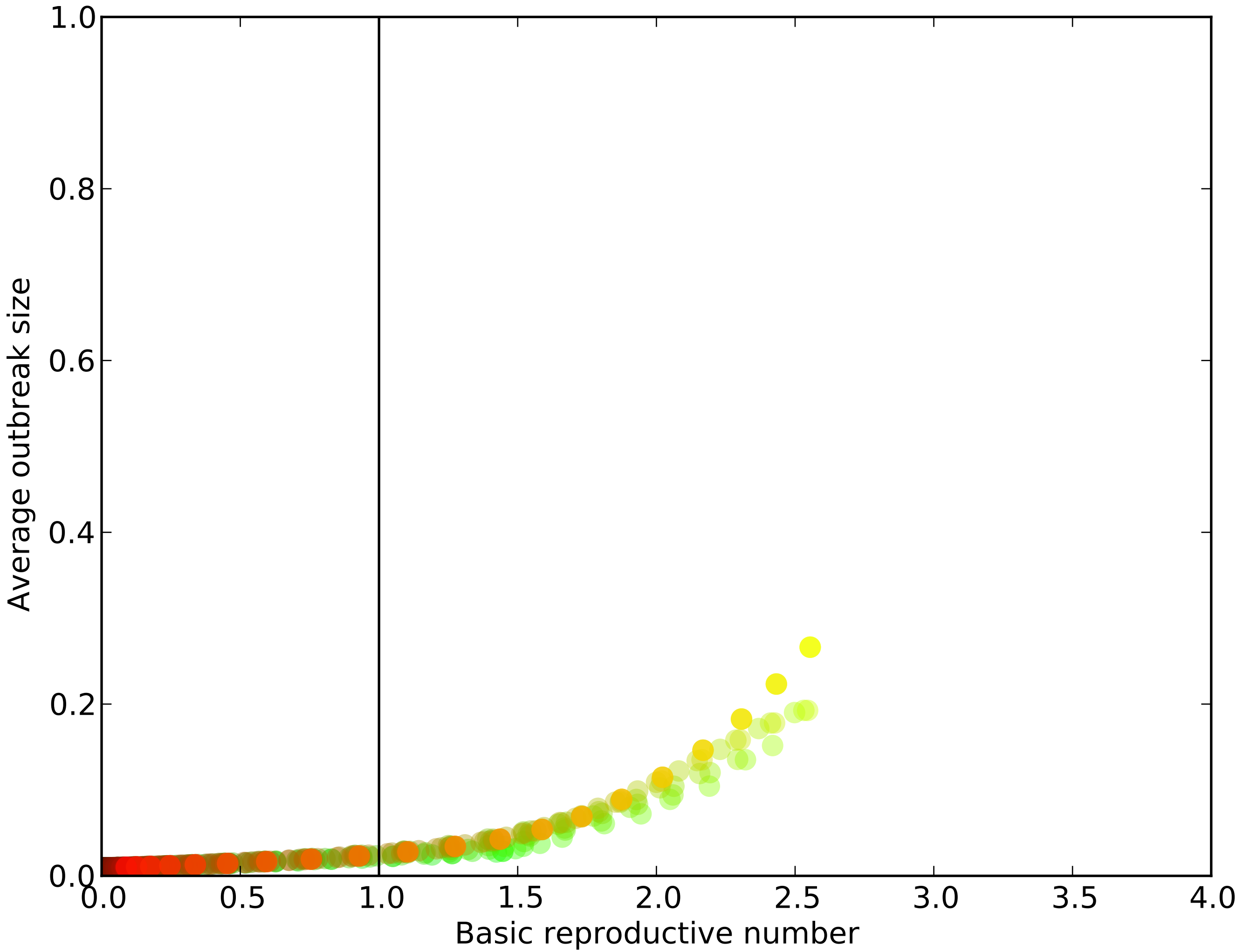
Average outbreak size
Basic reproductive number
0.0
0.2
0.4
0.6
0.8
1.0
0.0
0.5
1.0
1.5
2.0
2.5
3.0
3.5
4.0

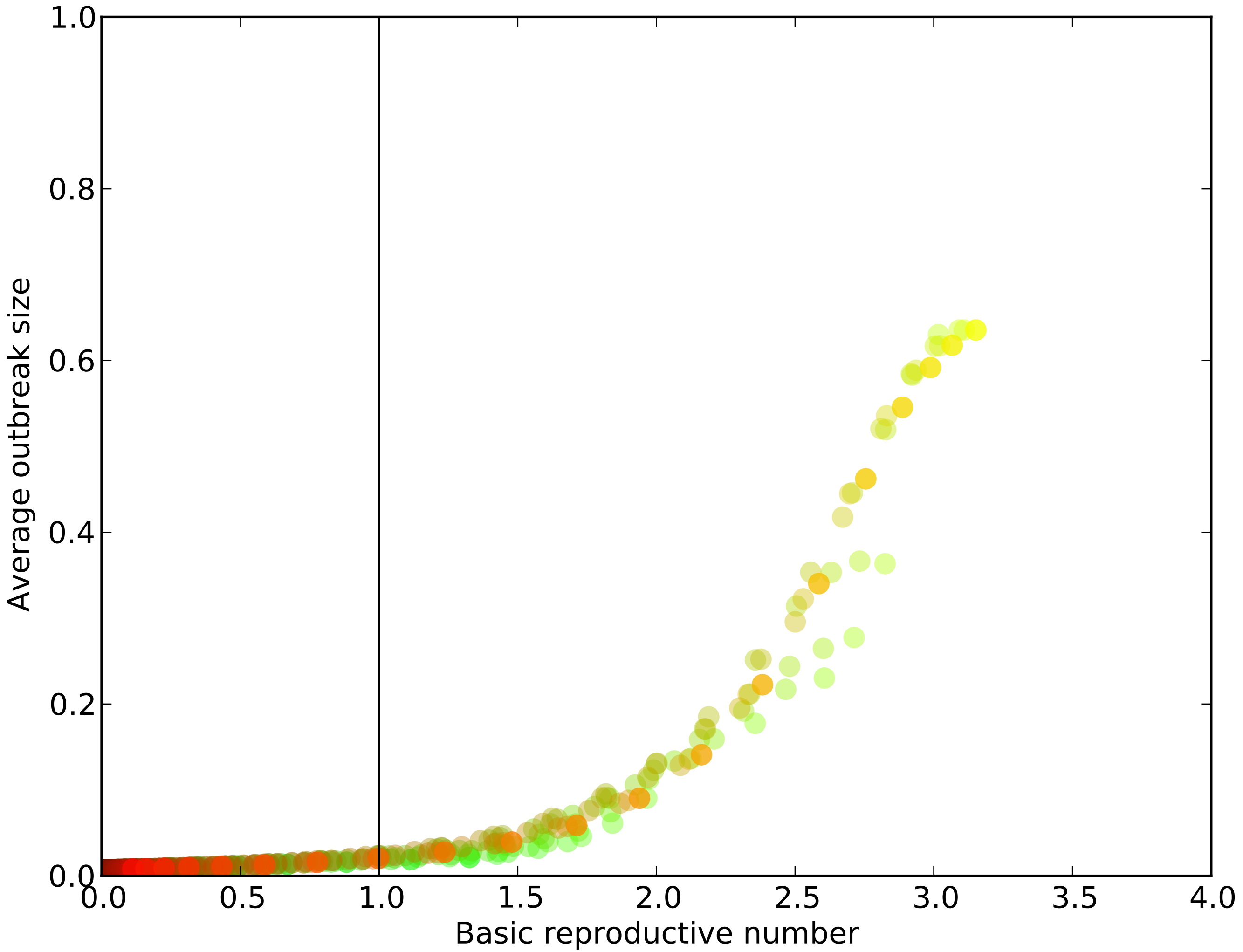

Average outbreak size
Basic reproductive number
0.0
0.2
0.4
0.6
0.8
1.0
0.0
0.5
1.0
1.5
2.0
2.5
3.0
3.5
4.0

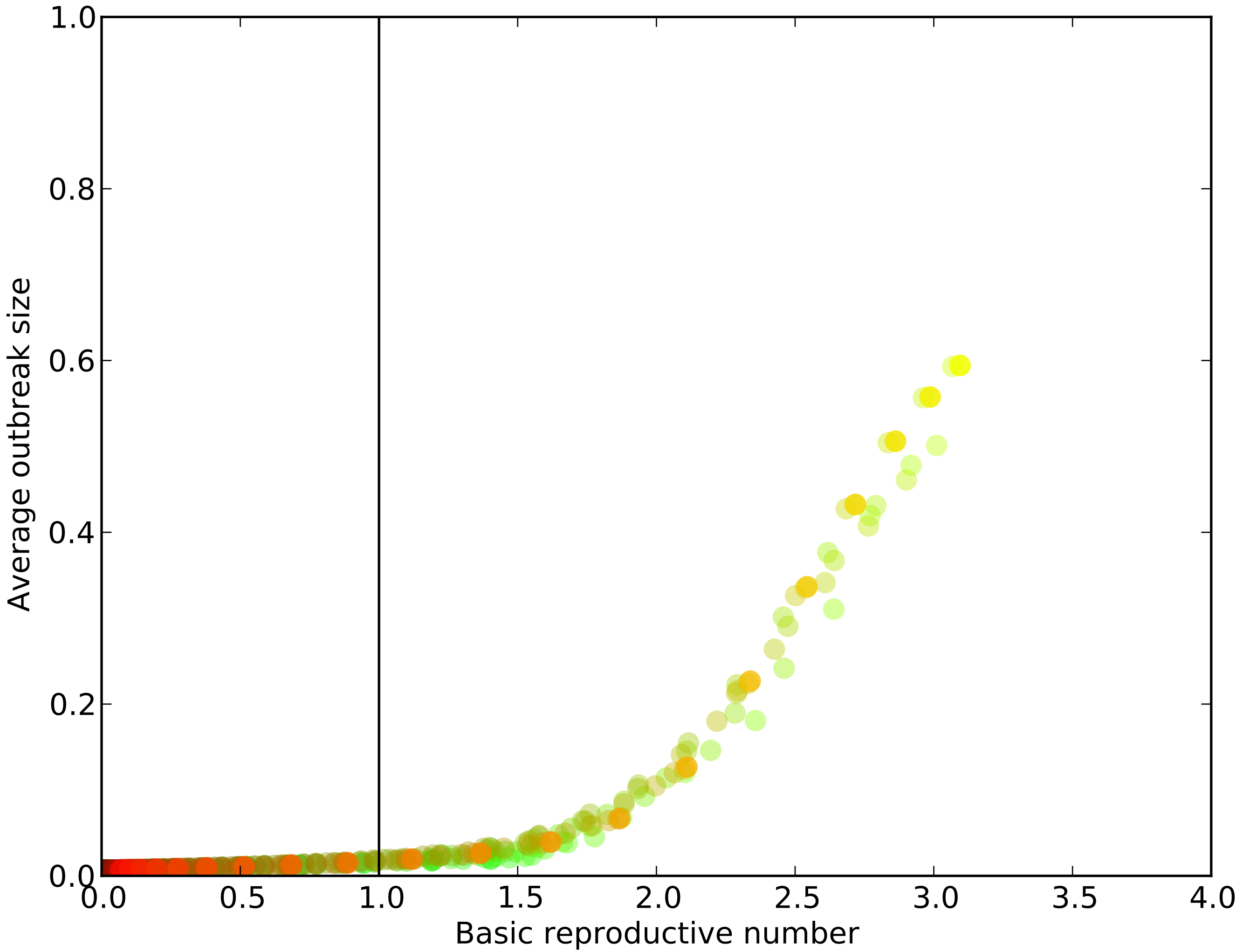

1.0
0.8
0.6
0.4
0.2
0.0
0.0
0.5
1.0
1.5
2.0
2.5
3.0
3.5
4.0
Average outbreak size
Basic reproductive number

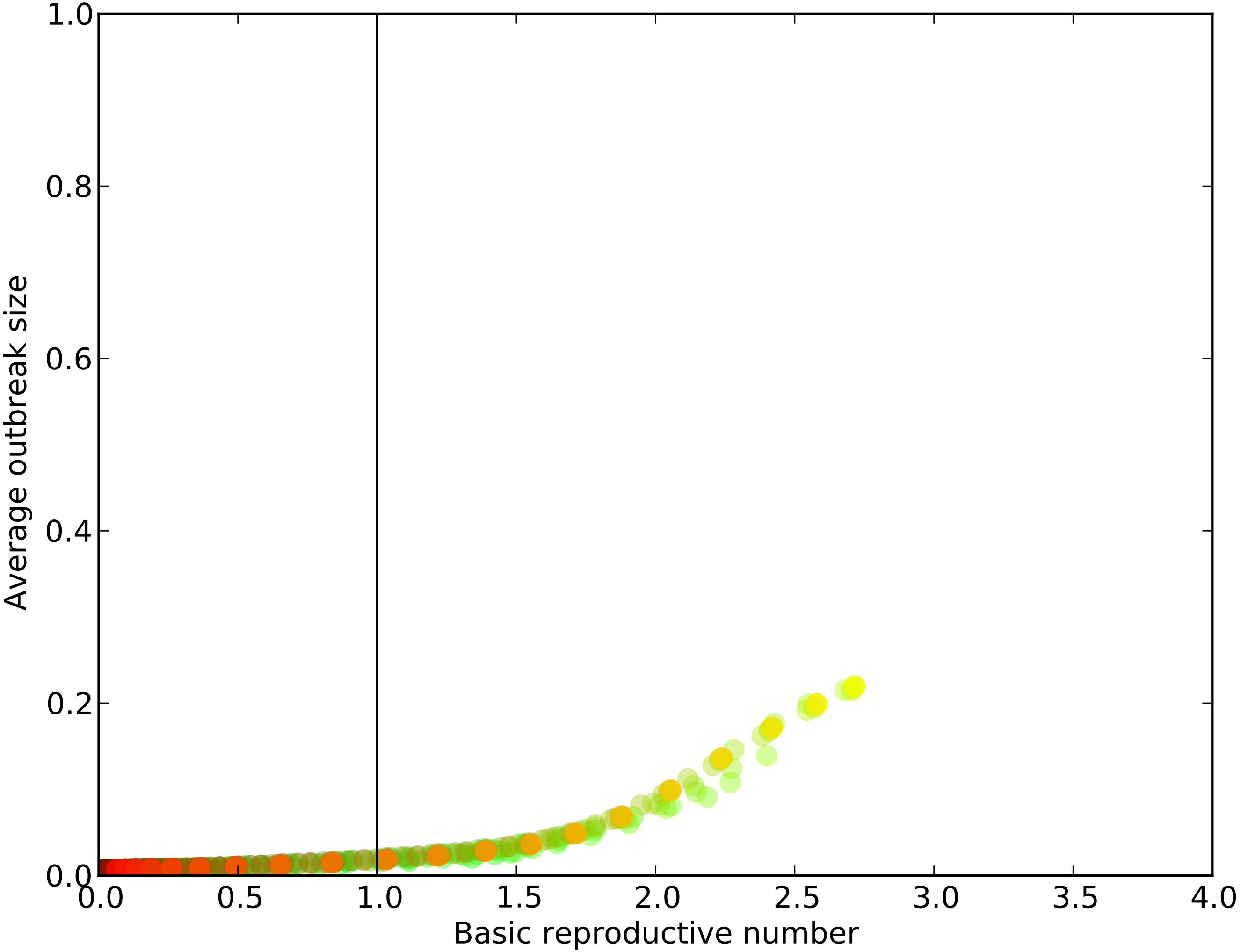
Average outbreak size
Basic reproductive number
0.0
0.2
0.4
0.6
0.8
1.0
0.0
0.5
1.0
1.5
2.0
2.5
3.0
3.5
4.0

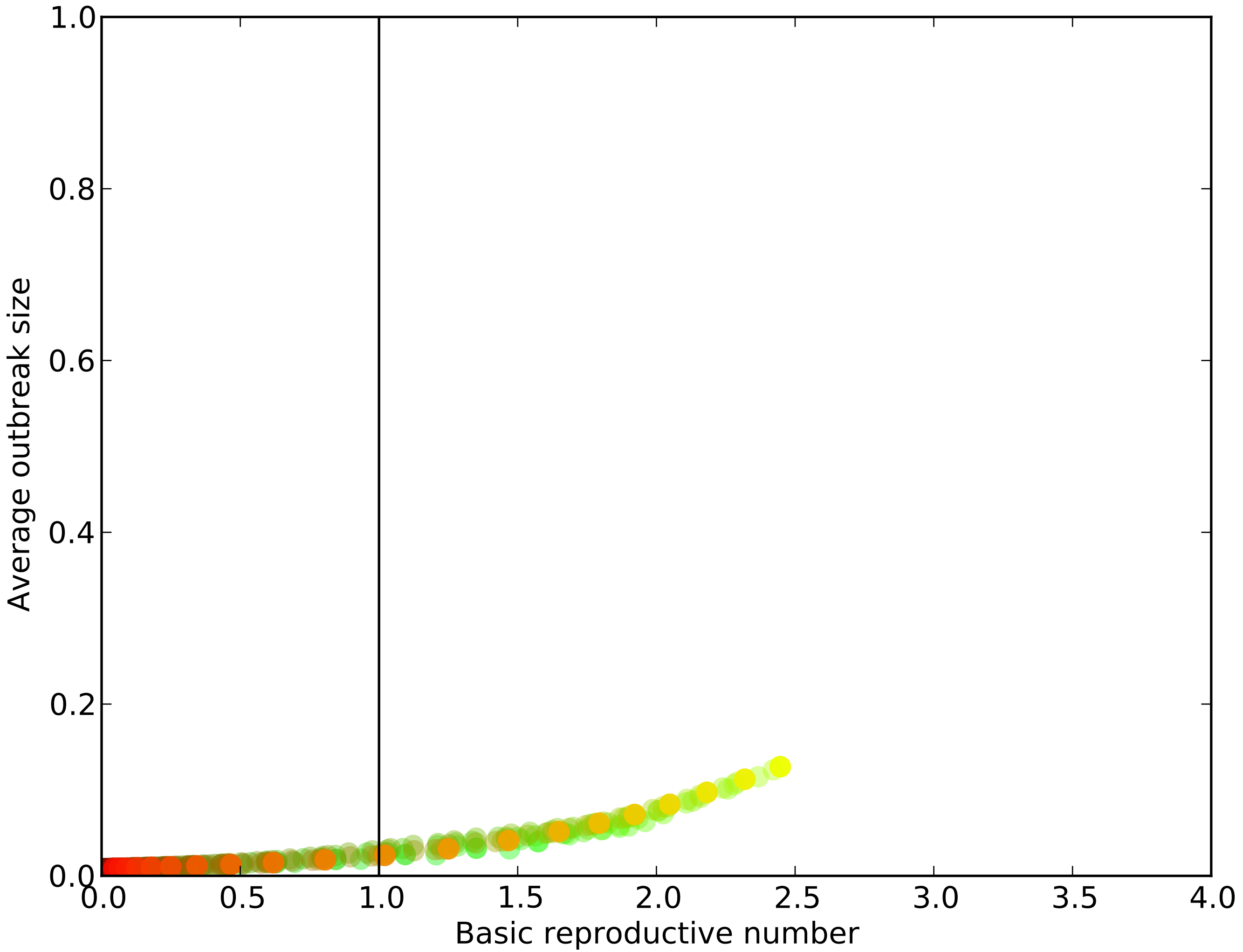

Average outbreak size
1.0
0.8
0.6
0.4
0.2
0.0
0.0
0.5
1.0
1.5
2.0
2.5
3.0
3.5
4.0
Basic reproductive number

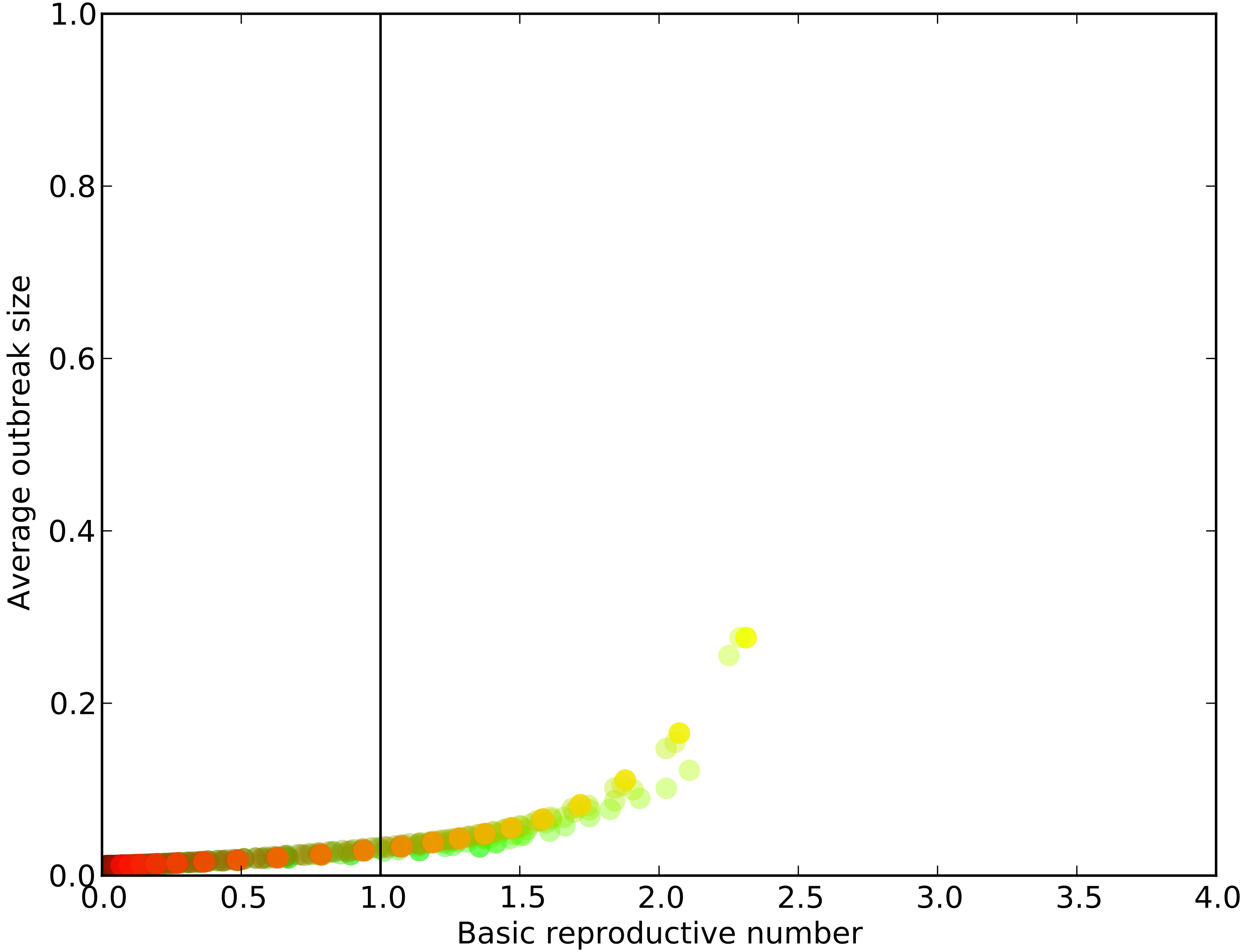
1.0
0.8
0.6
0.4
0.2
0.0
Average outbreak size
0.0
0.5
1.0
1.5
2.0
2.5
3.0
3.5
4.0
Basic reproductive number

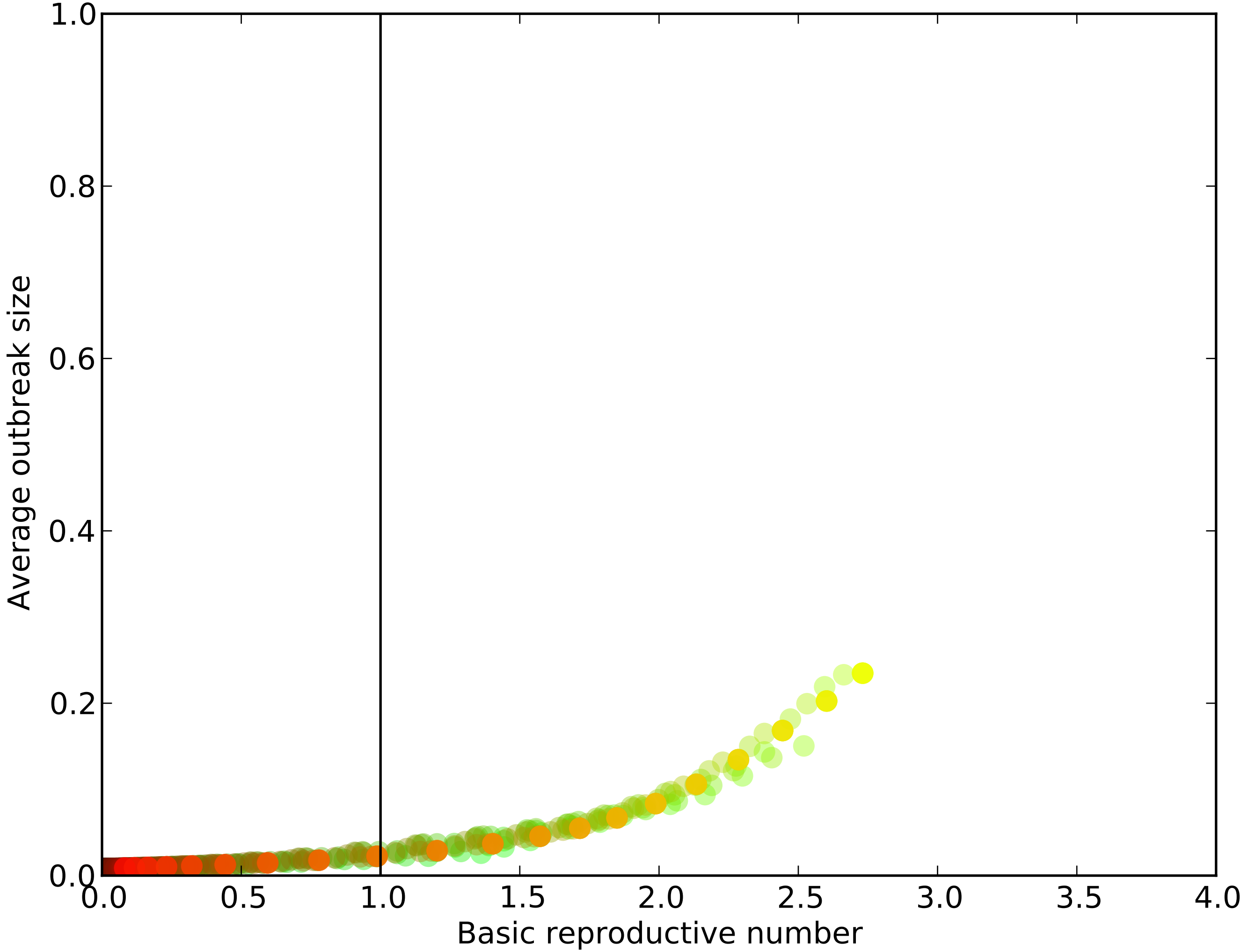
1.0
0.8
0.6
0.4
0.2
0.0
Average outbreak size
0.0
0.5
1.0
1.5
2.0
2.5
3.0
3.5
4.0
Basic reproductive number

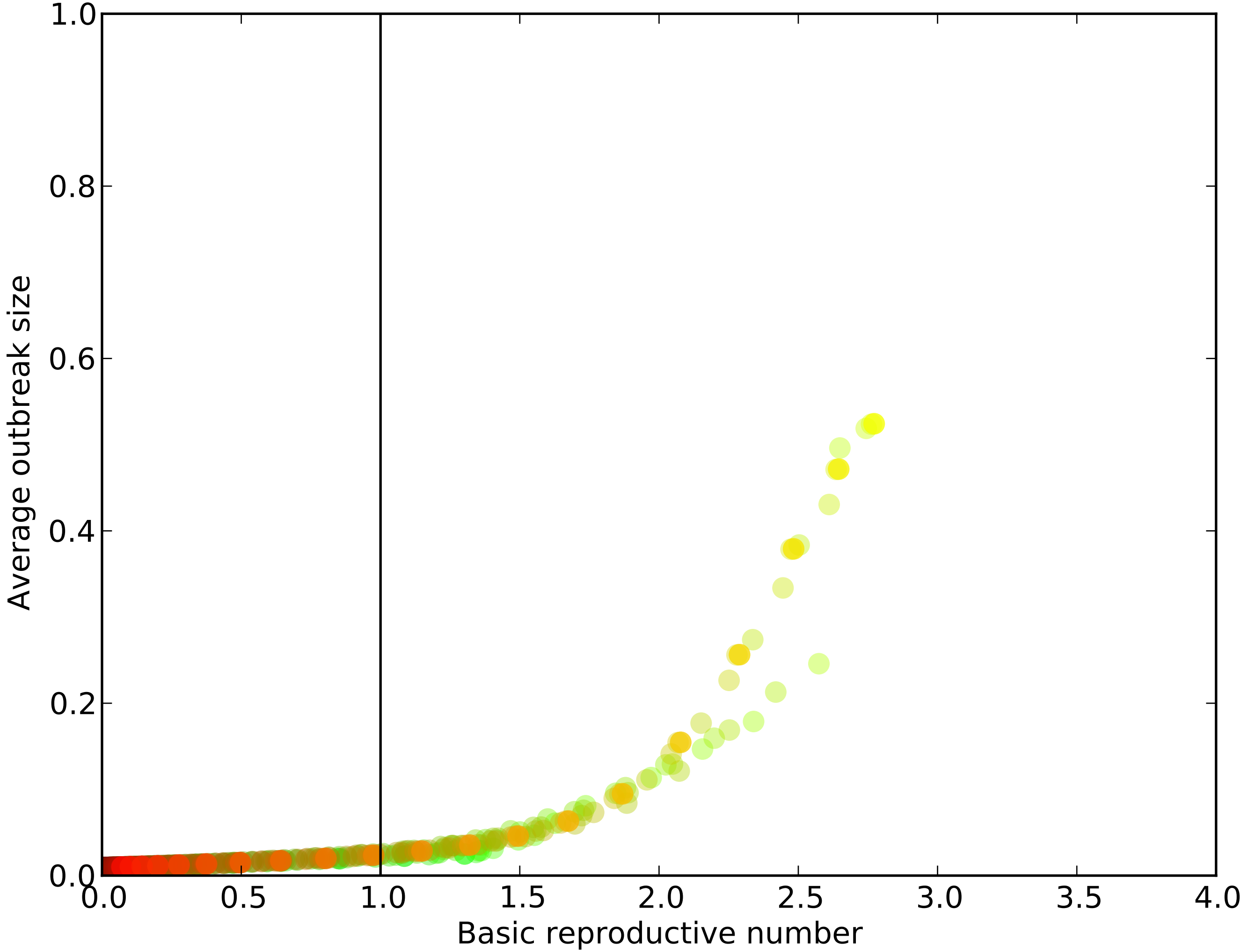

1.0
0.8
0.6
0.4
0.2
0.0
Average outbreak size
0.0
0.5
1.0
1.5
2.0
2.5
3.0
3.5
4.0
Basic reproductive number

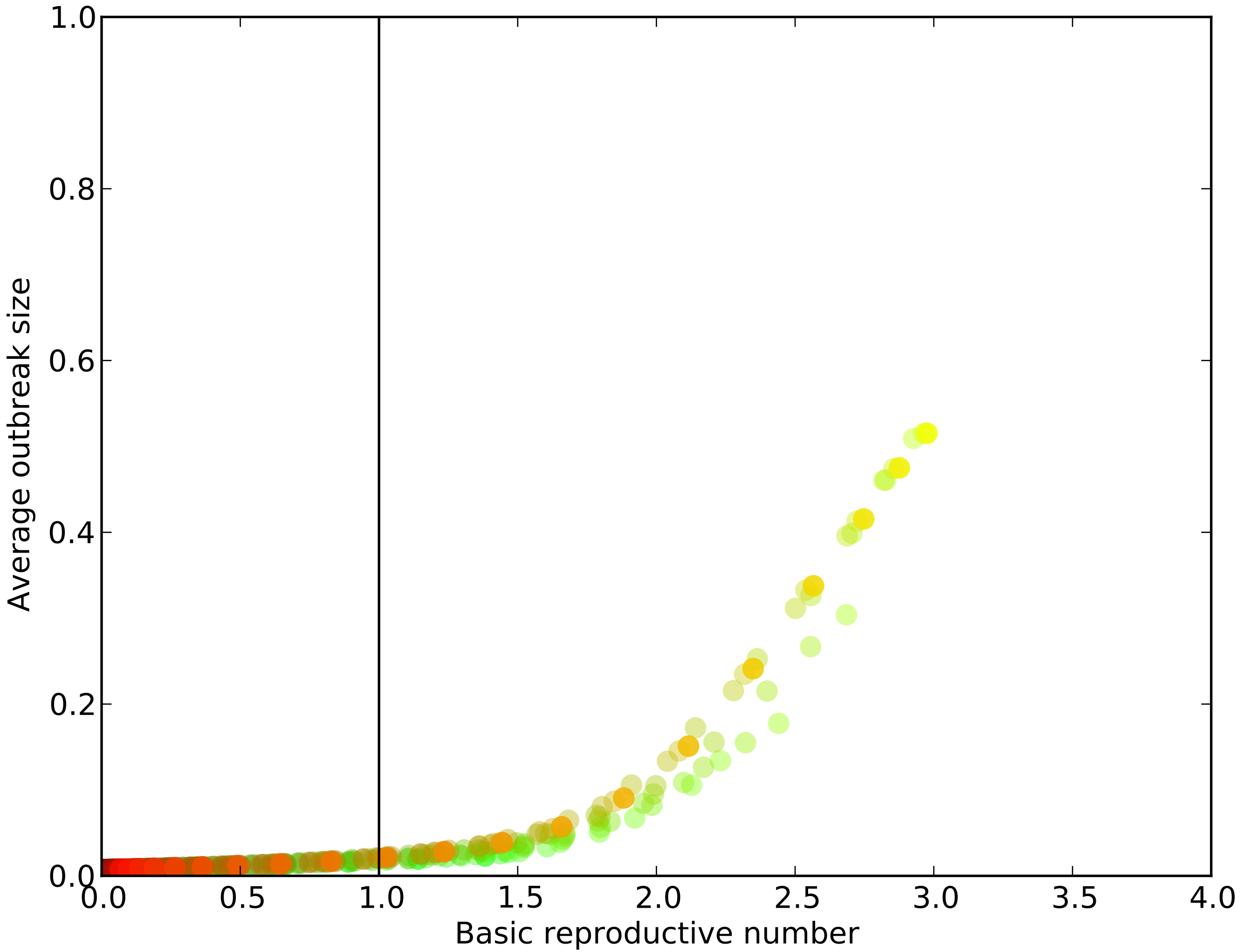

Average outbreak size
Basic reproductive number
1.0
0.8
0.6
0.4
0.2
0.0
0.0
0.5
1.0
1.5
2.0
2.5
3.0
3.5
4.0

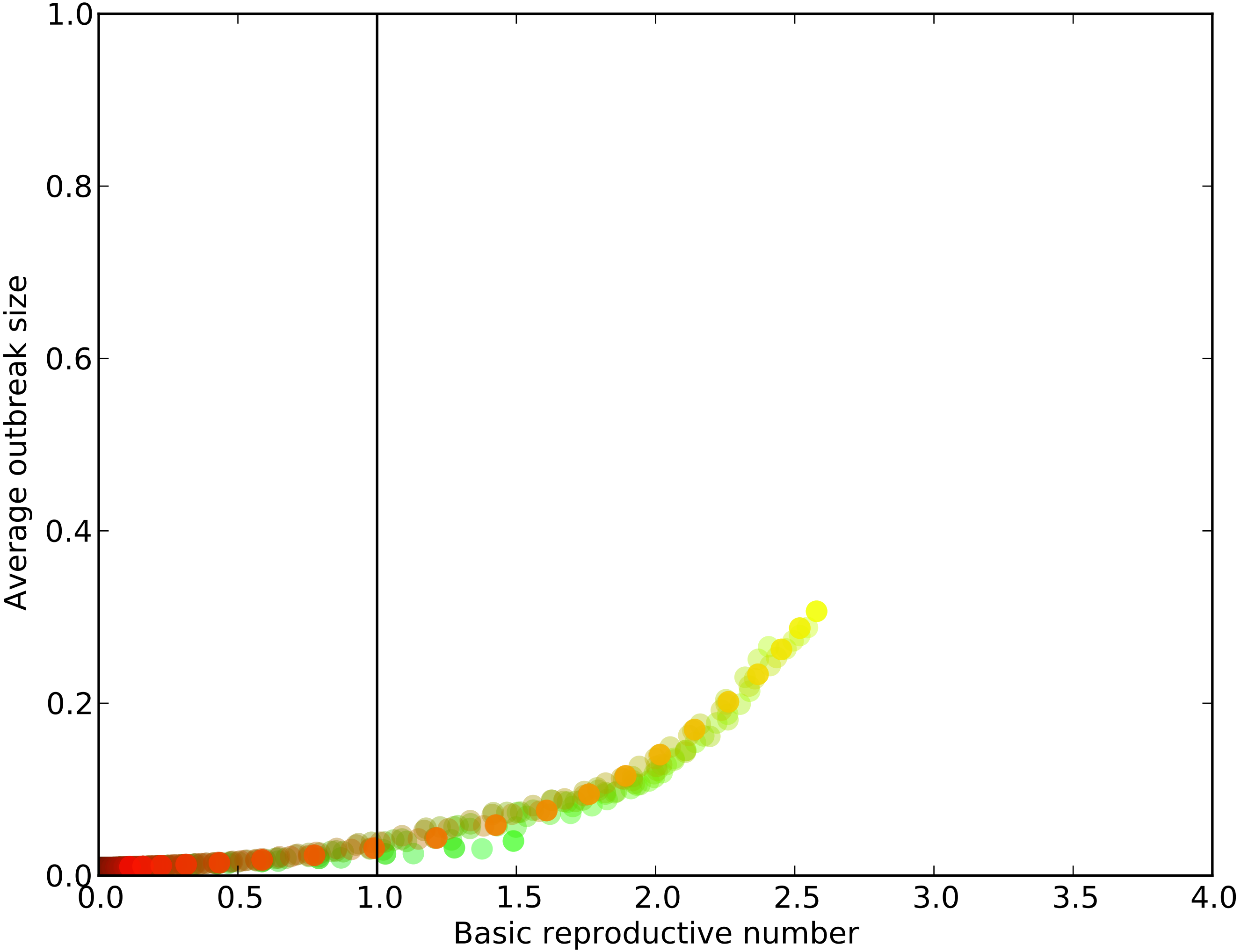

1.0
0.8
0.6
0.4
0.2
0.0
Average outbreak size
0.0
0.5
1.0
1.5
2.0
2.5
3.0
3.5
4.0
Basic reproductive number

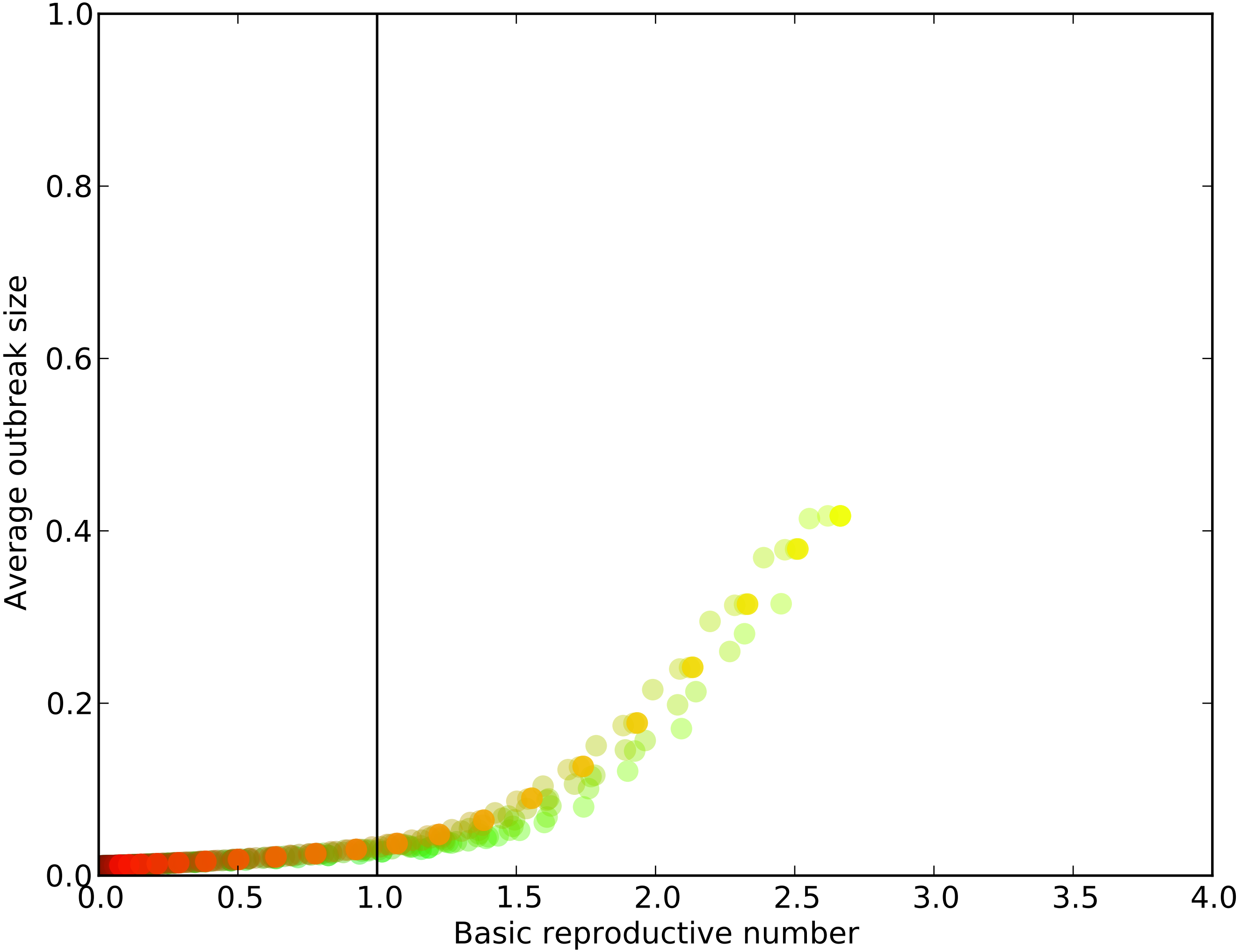

Average outbreak size
Basic reproductive number
0.0
0.2
0.4
0.6
0.8
1.0
0.0
0.5
1.0
1.5
2.0
2.5
3.0
3.5
4.0

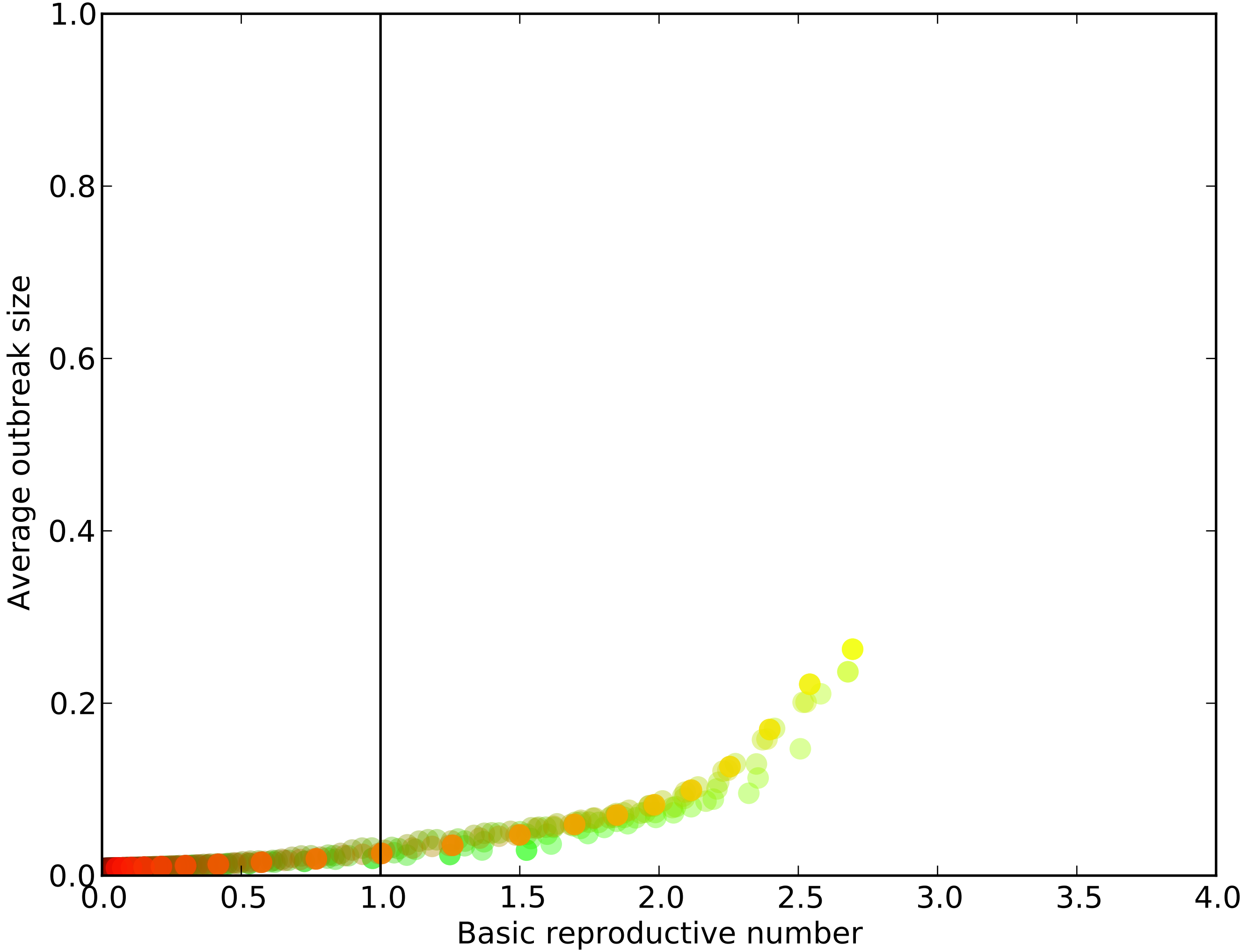
1.0
0.8
0.6
0.4
0.2
0.0
0.0
0.5
1.0
1.5
2.0
2.5
3.0
3.5
4.0
Average outbreak size
Basic reproductive number

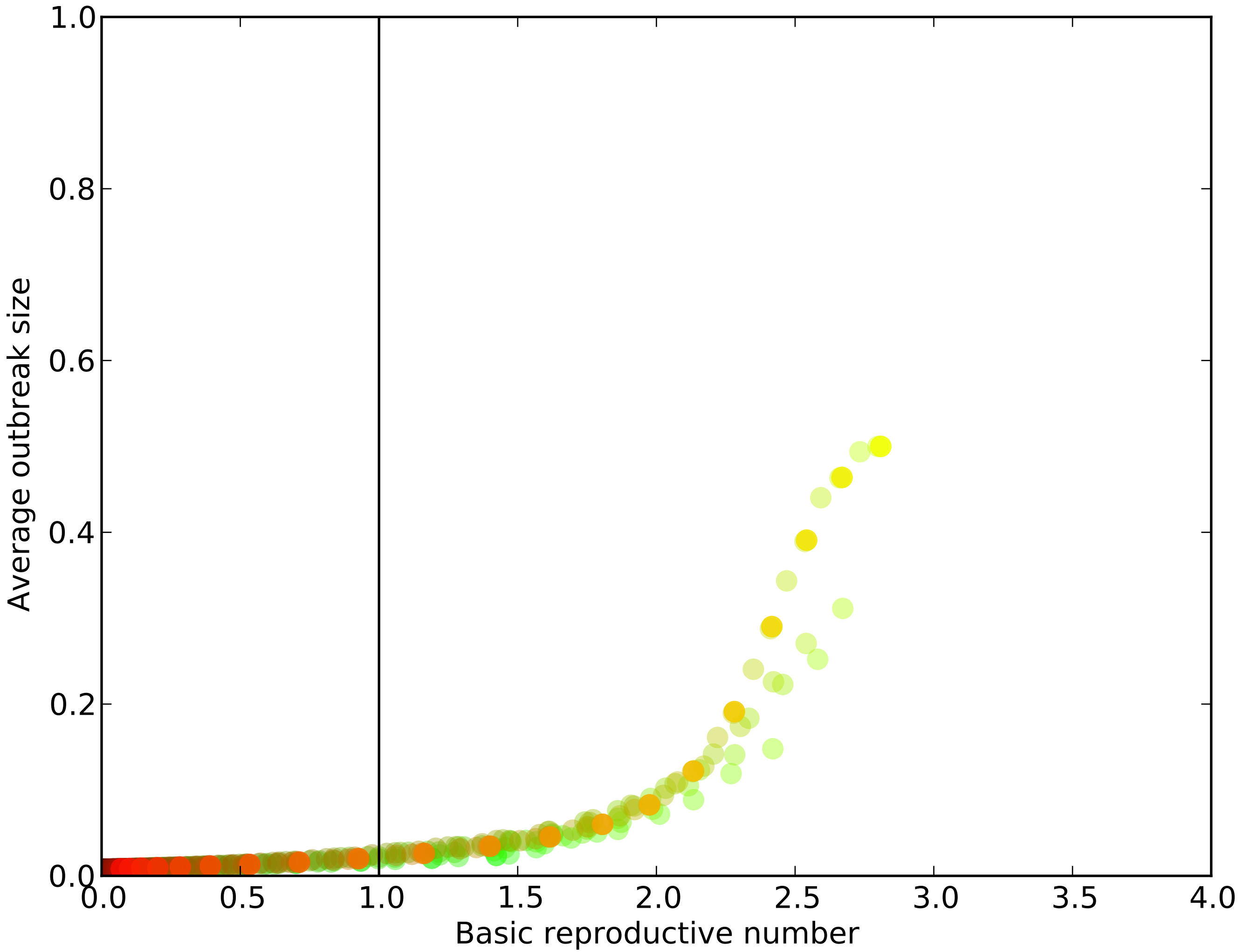

1.0
0.8
0.6
0.4
0.2
0.0
Average outbreak size
0.0
0.5
1.0
1.5
2.0
2.5
3.0
3.5
4.0
Basic reproductive number

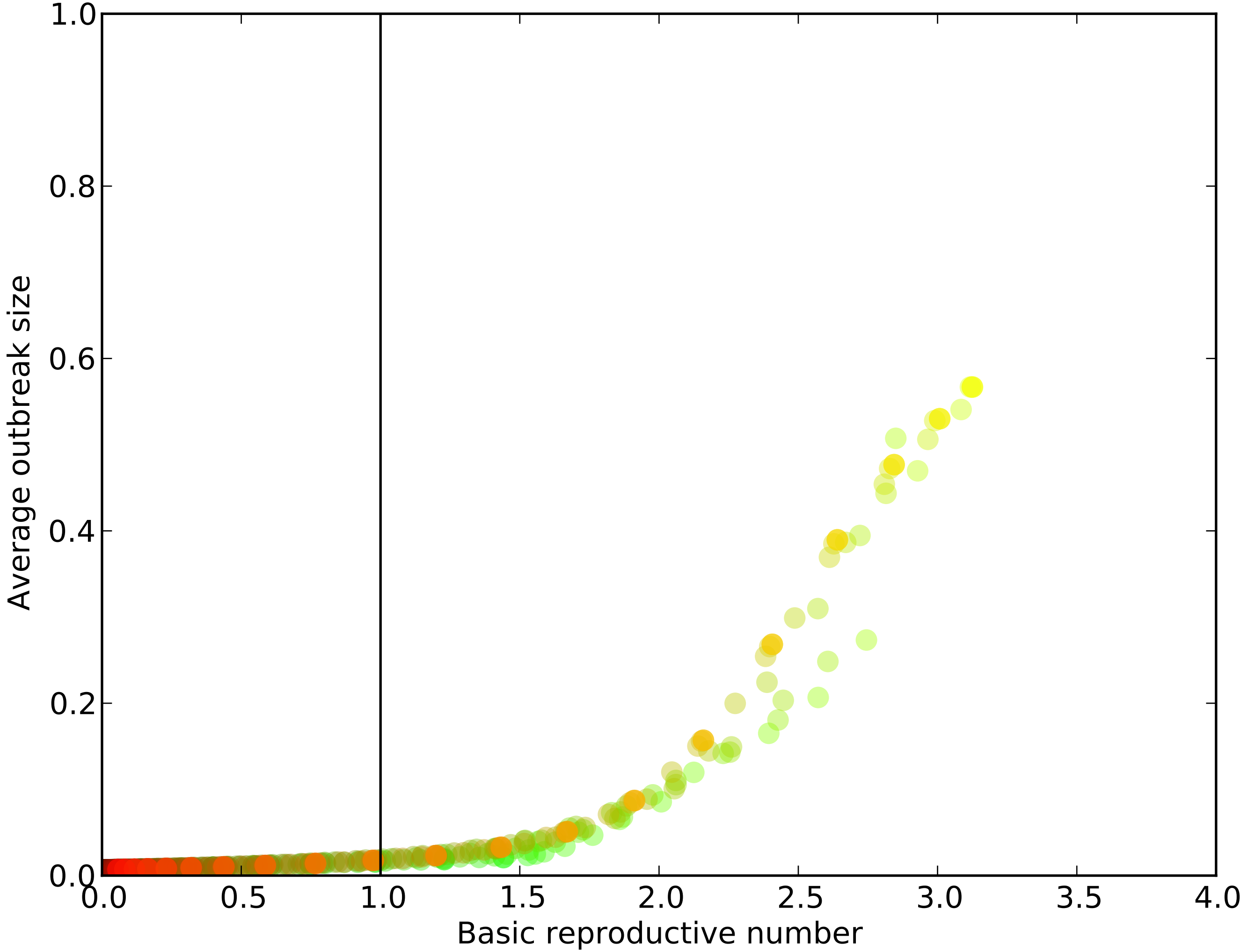

1.0
0.8
0.6
0.4
0.2
0.0
Average outbreak size
0.0
0.5
1.0
1.5
2.0
2.5
3.0
3.5
4.0
Basic reproductive number

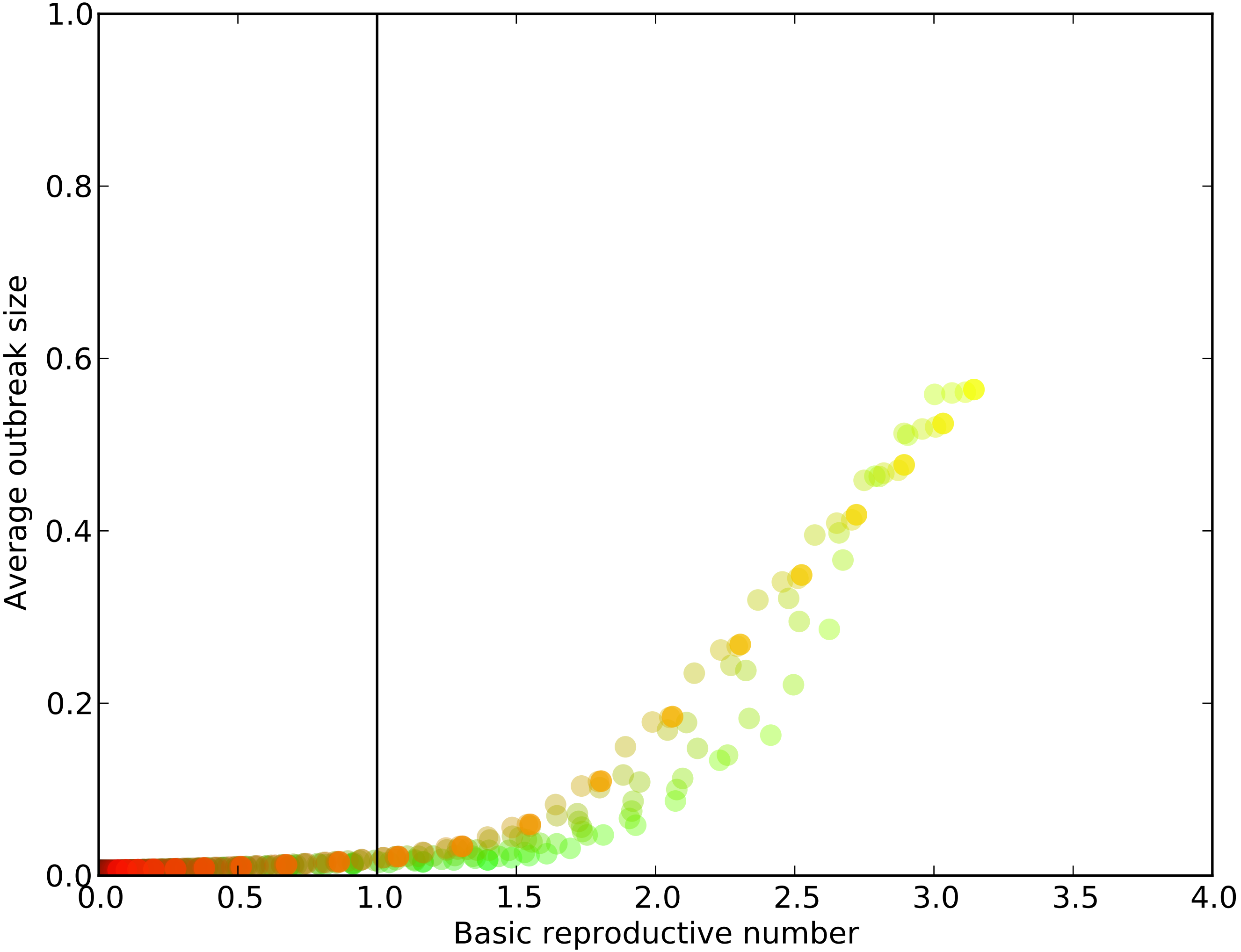
1.0
0.8
0.6
0.4
0.2
0.0
Average outbreak size
0.0
0.5
1.0
1.5
2.0
2.5
3.0
3.5
4.0
Basic reproductive number

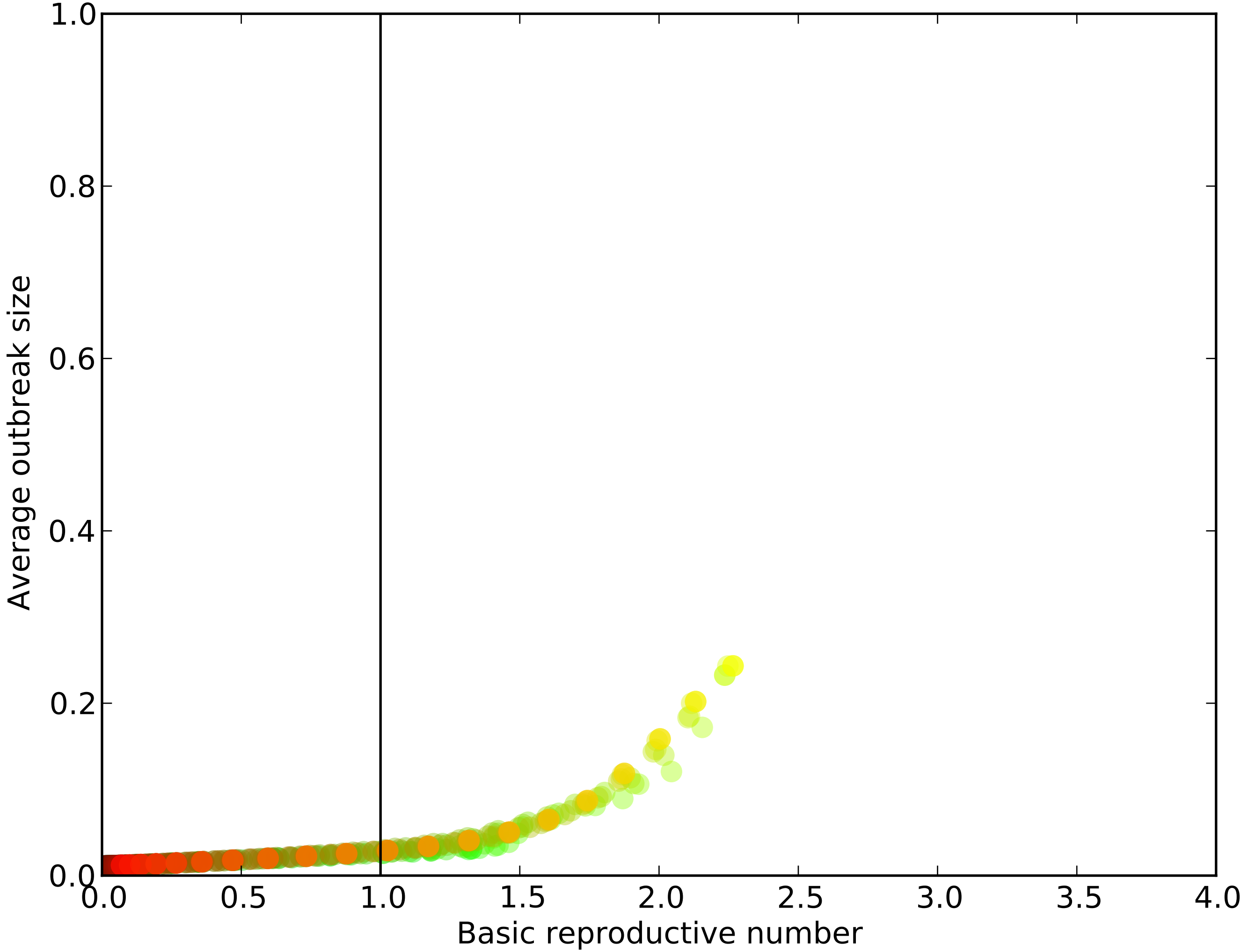

1.0
0.8
0.6
0.4
0.2
0.0
Average outbreak size
0.0
0.5
1.0
1.5
2.0
2.5
3.0
3.5
4.0
Basic reproductive number

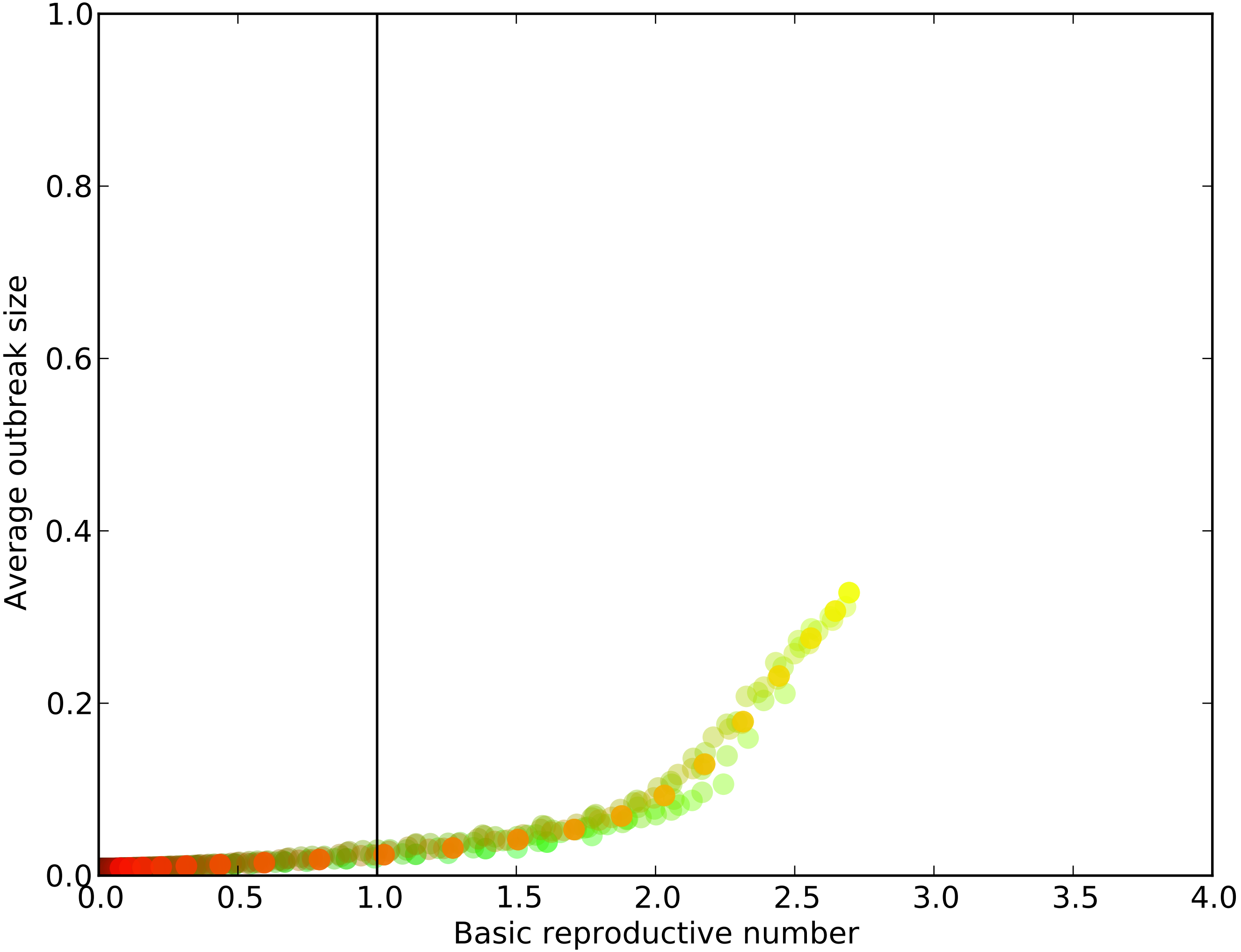

1.0
0.8
0.6
0.4
0.2
0.0
Average outbreak size
0.0
0.5
1.0
1.5
2.0
2.5
3.0
3.5
4.0
Basic reproductive number

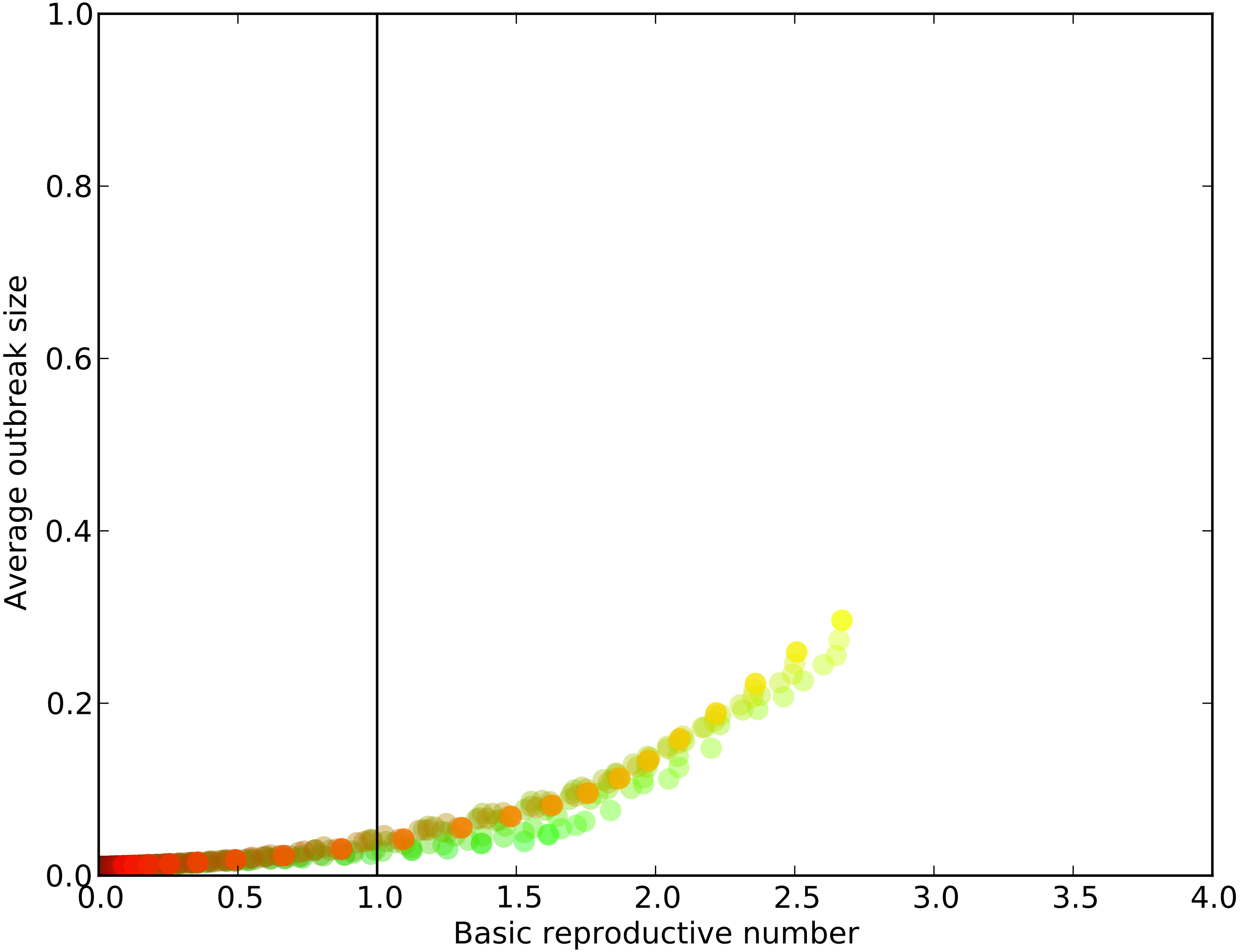

Average outbreak size
Basic reproductive number
0.0
0.2
0.4
0.6
0.8
1.0
0.5
1.0
1.5
2.0
2.5
3.0
3.5
4.0

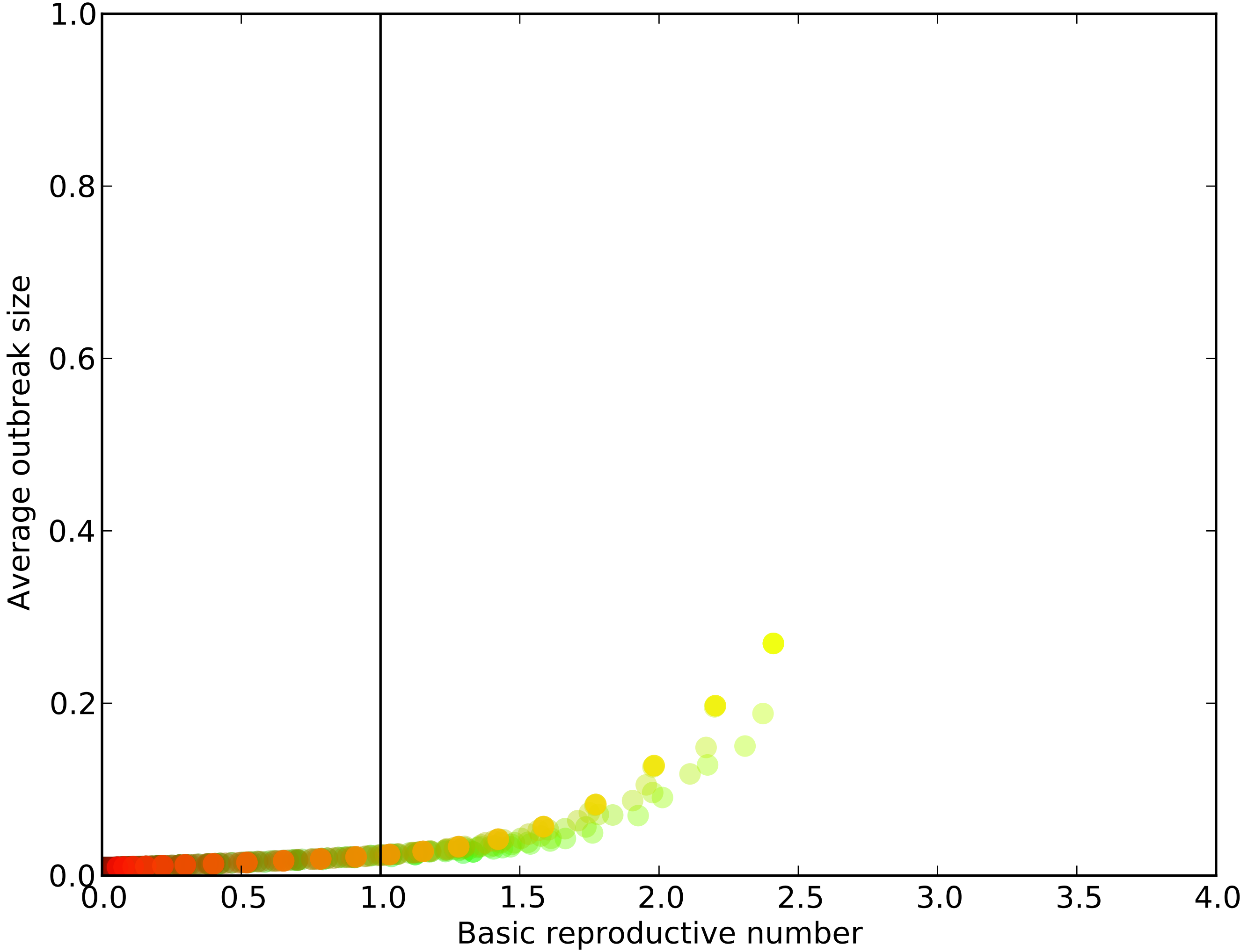

1.0
0.8
0.6
0.4
0.2
0.0
Average outbreak size
0.0
0.5
1.0
1.5
2.0
2.5
3.0
3.5
4.0
Basic reproductive number

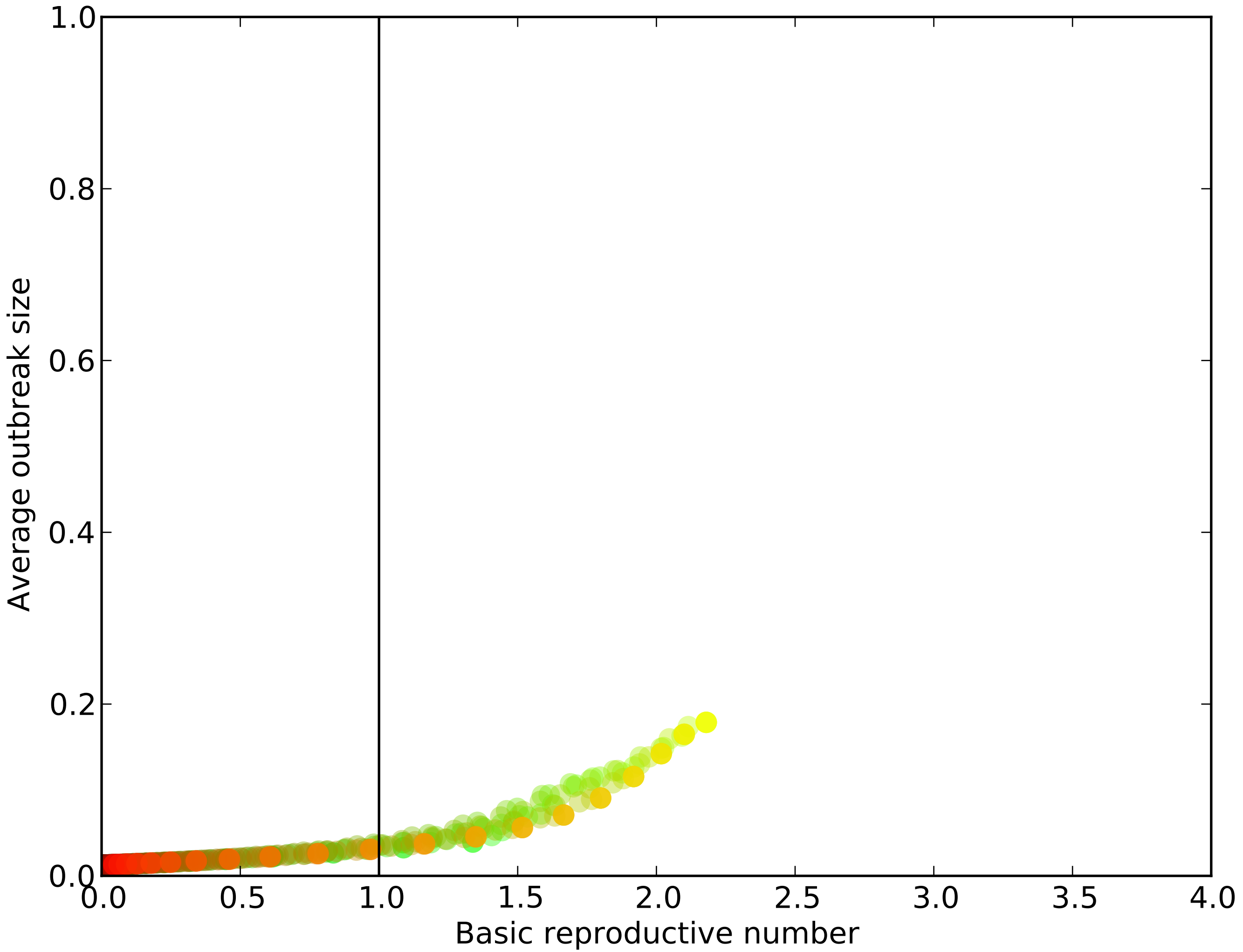

Average outbreak size
Basic reproductive number
1.0
0.8
0.6
0.4
0.2
0.0
0.0
0.5
1.0
1.5
2.0
2.5
3.0
3.5
4.0

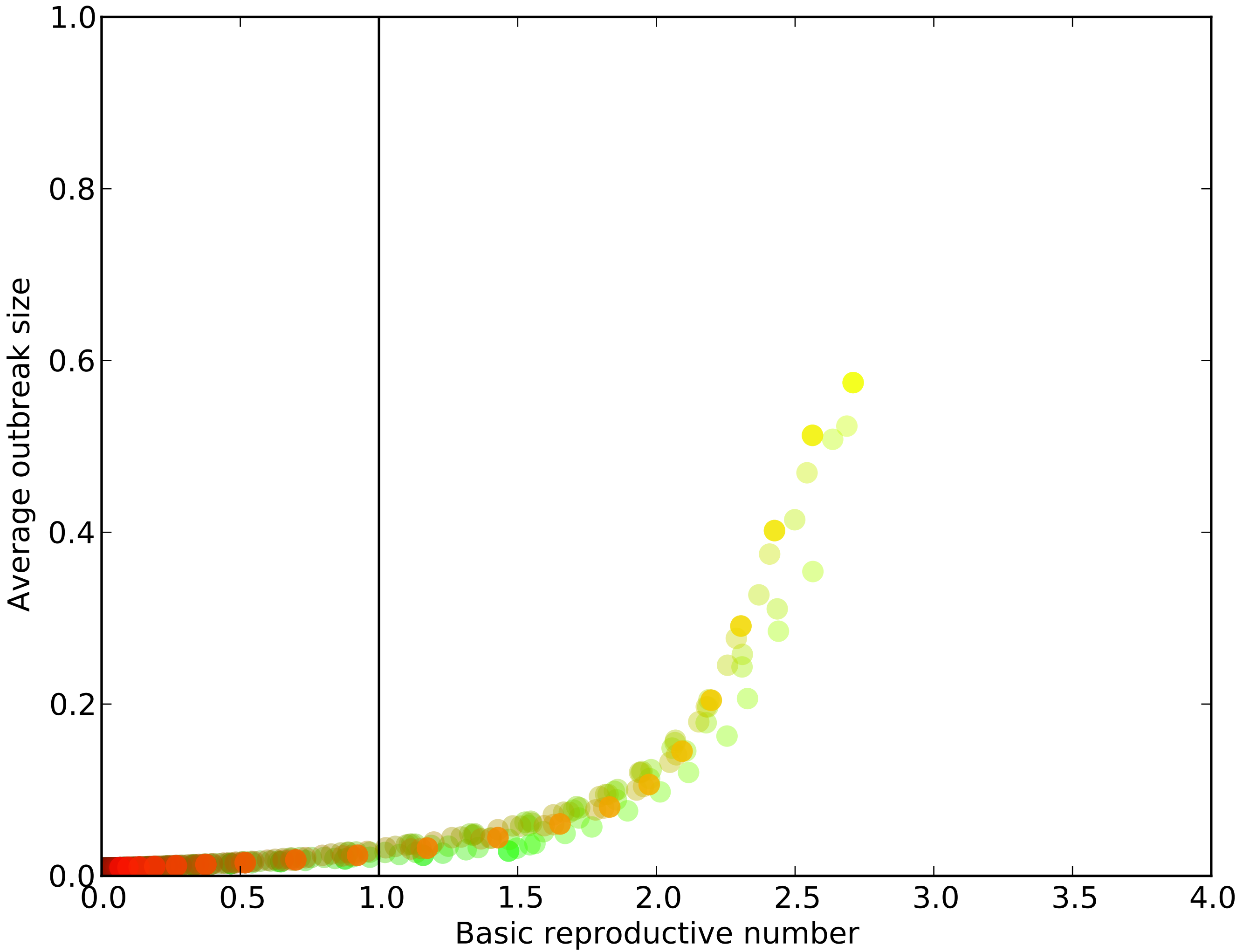

1.0
0.8
0.6
0.4
0.2
0.0
Average outbreak size
0.0
0.5
1.0
1.5
2.0
2.5
3.0
3.5
4.0
Basic reproductive number

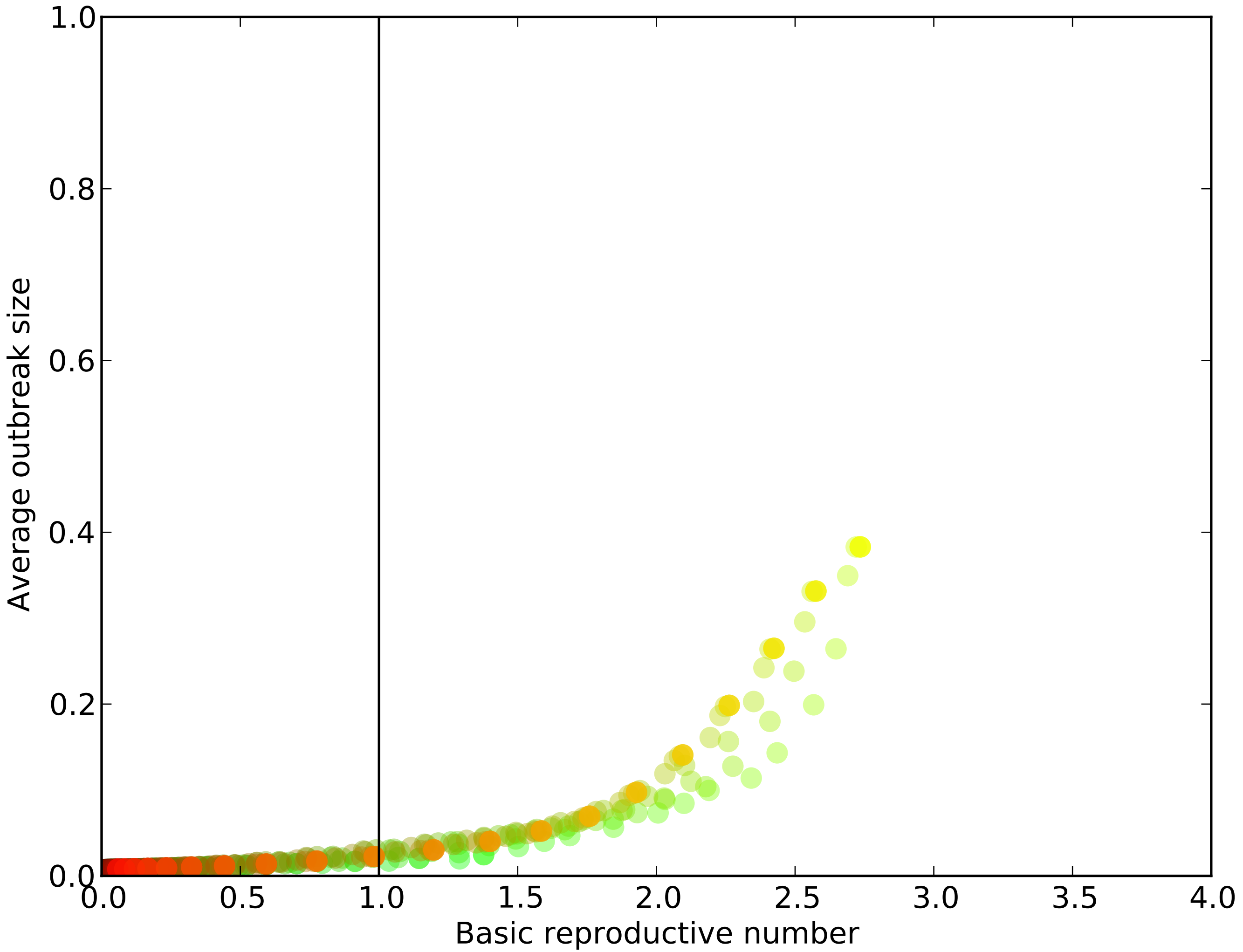

1.0
0.8
0.6
0.4
0.2
0.0
Average outbreak size
0.0
0.5
1.0
1.5
2.0
2.5
3.0
3.5
4.0
Basic reproductive number

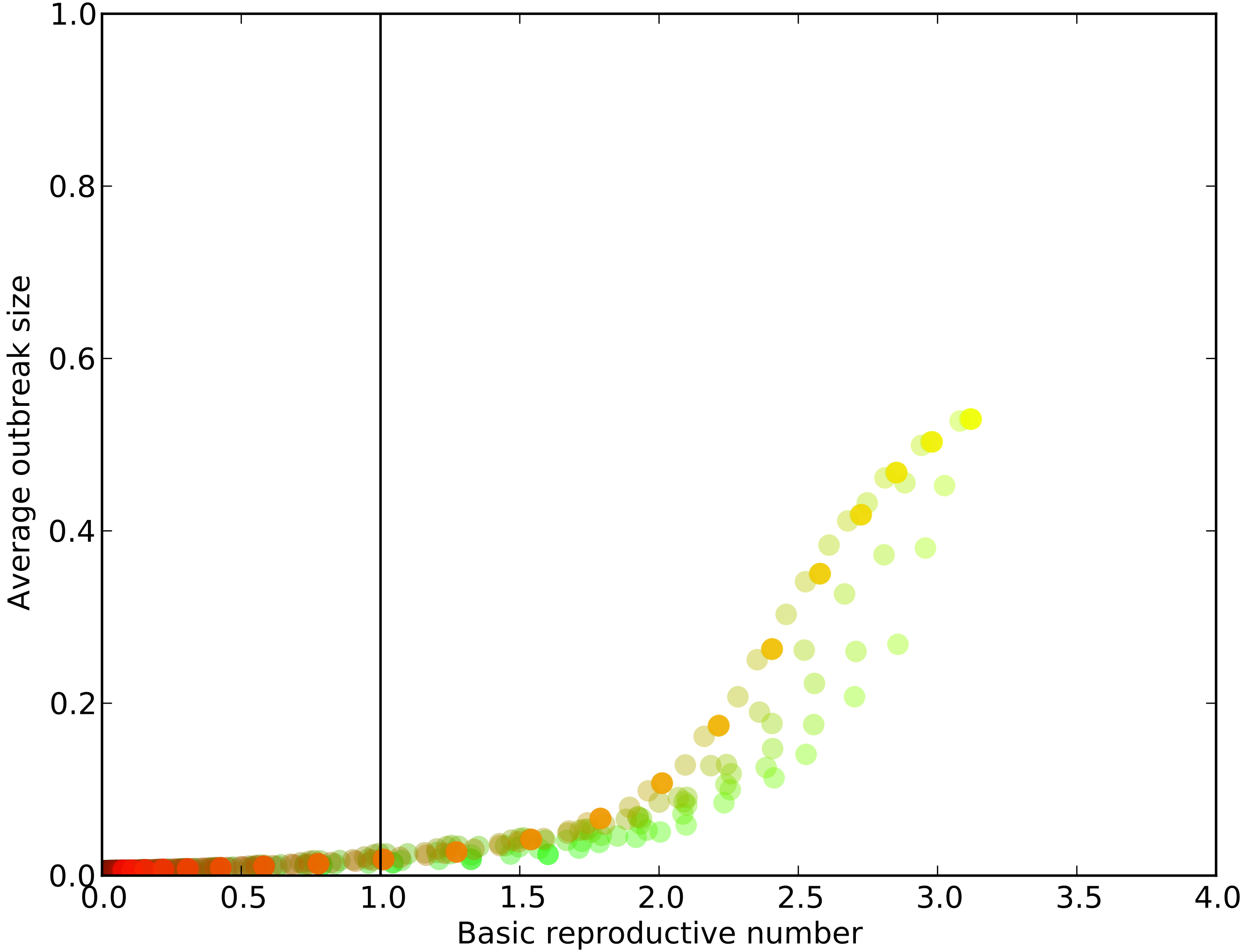
1.0
0.8
0.6
0.4
0.2
0.0
Average outbreak size
0.0
0.5
1.0
1.5
2.0
2.5
3.0
3.5
4.0
Basic reproductive number

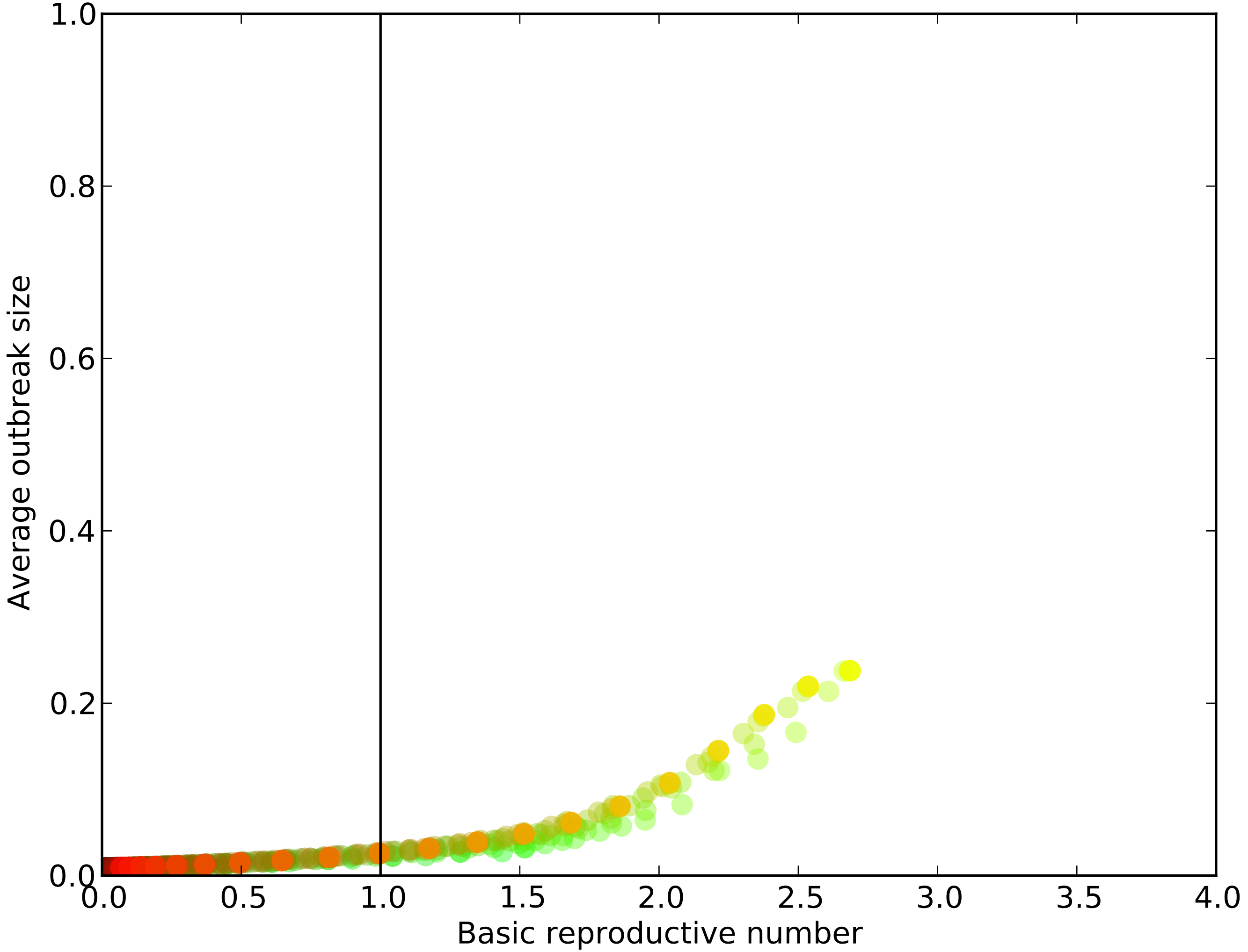

1.0
0.8
0.6
0.4
0.2
0.0
0.0
0.5
1.0
1.5
2.0
2.5
3.0
3.5
4.0
Average outbreak size
Basic reproductive number

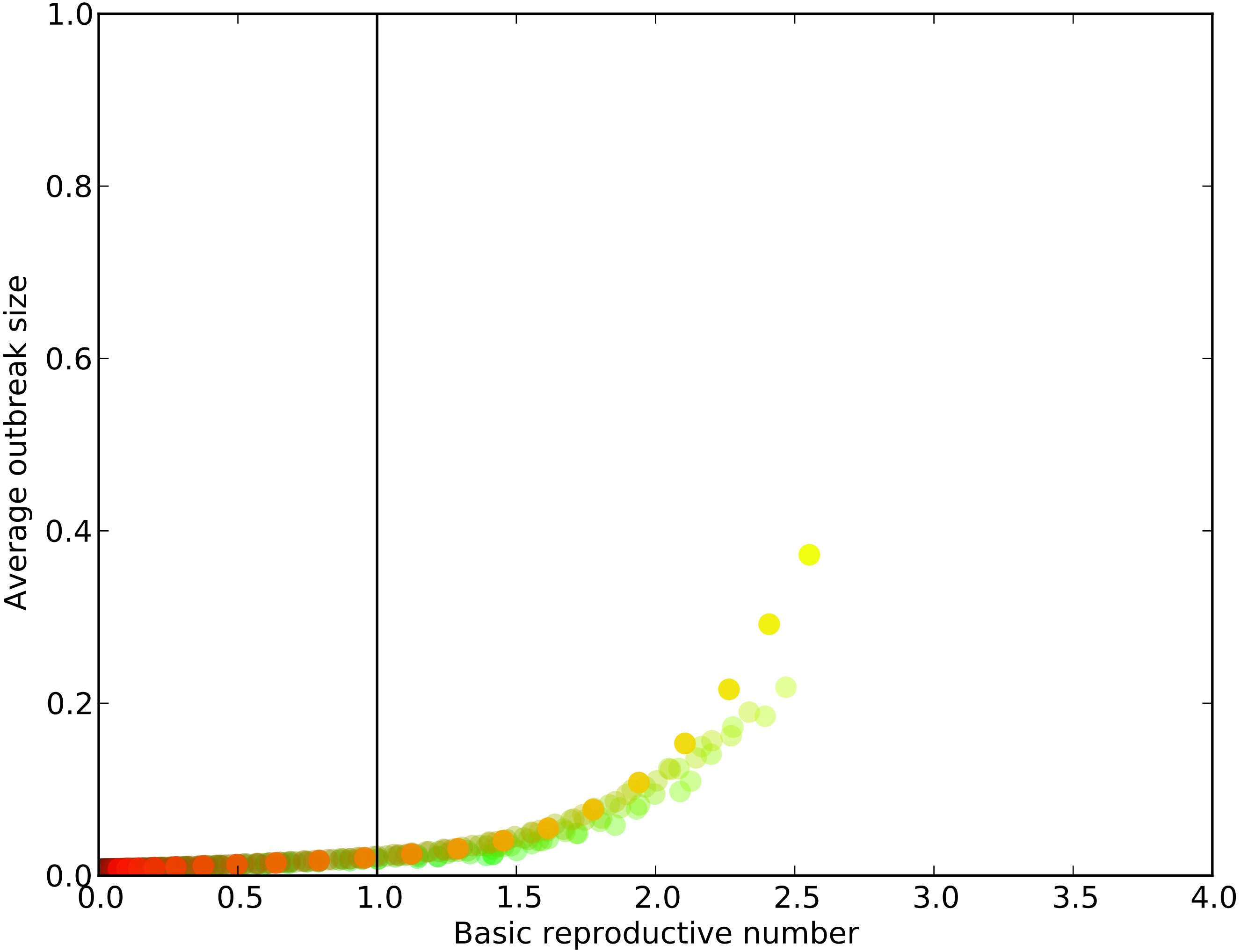

Average outbreak size
1.0
0.8
0.6
0.4
0.2
0.0
0.0
0.5
1.0
1.5
2.0
2.5
3.0
3.5
4.0
Basic reproductive number

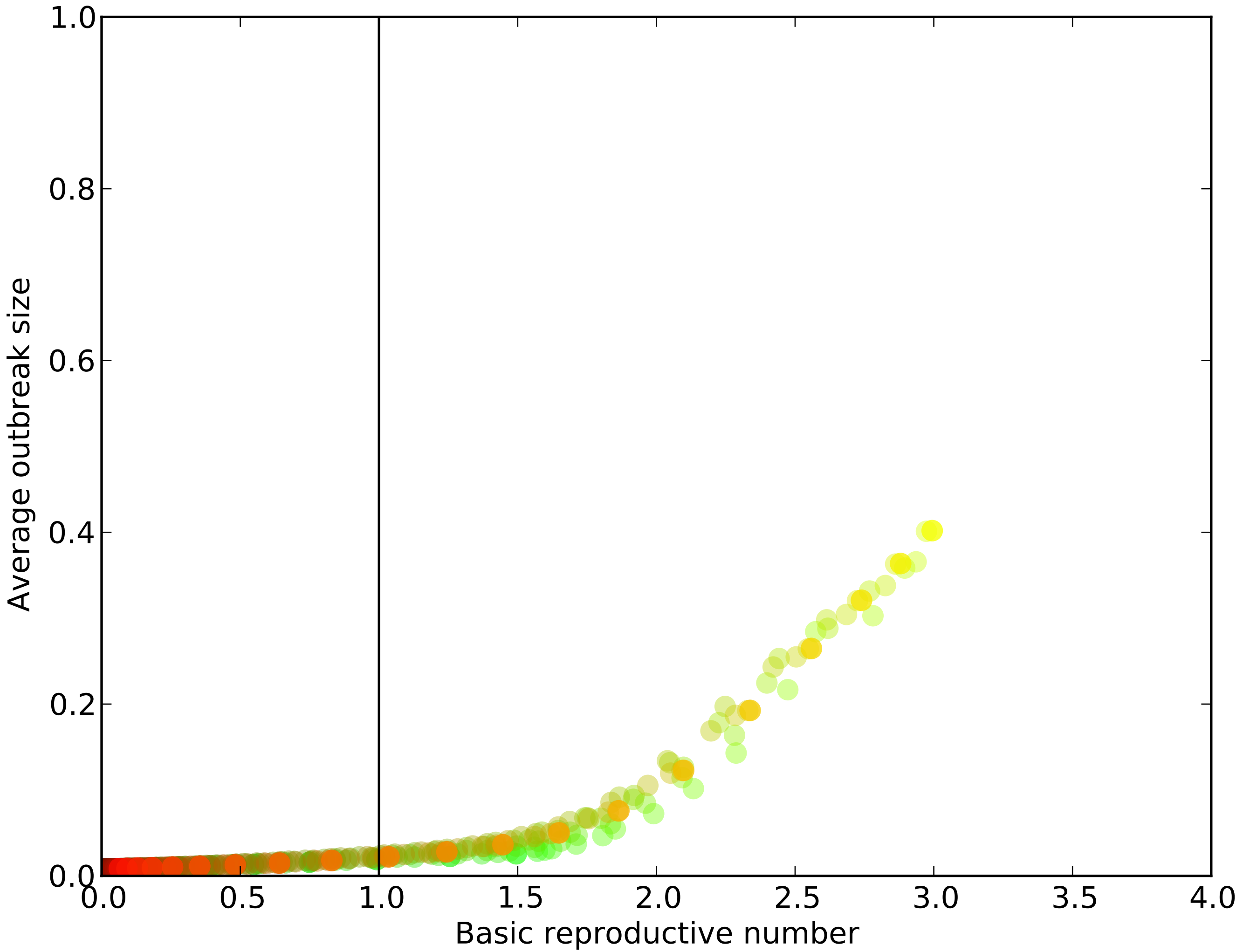
1.0
0.8
0.6
0.4
0.2
0.0
Average outbreak size
0.0
0.5
1.0
1.5
2.0
2.5
3.0
3.5
4.0
Basic reproductive number

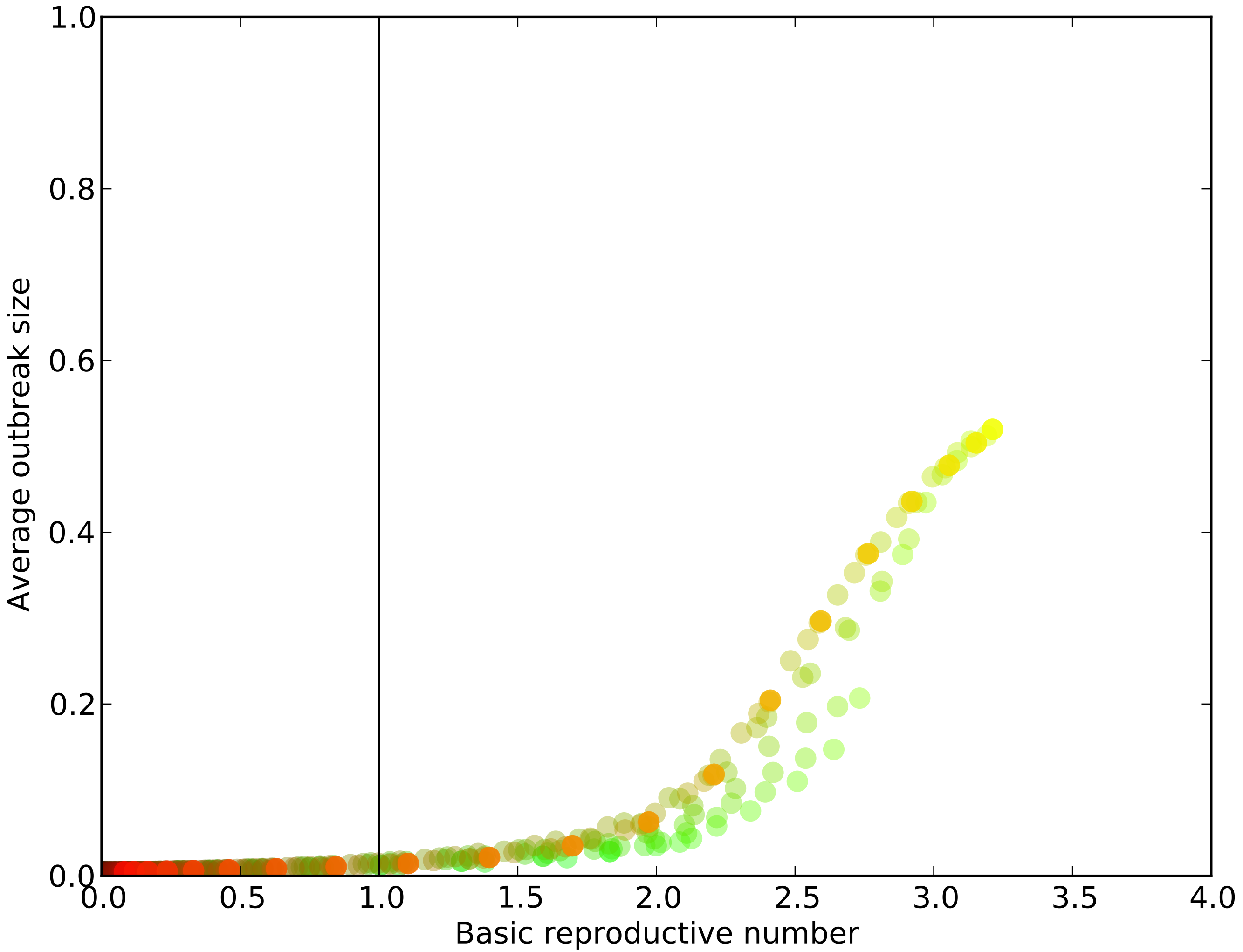

1.0
0.8
0.6
0.4
0.2
0.0
0.0
0.5
1.0
1.5
2.0
2.5
3.0
3.5
4.0
Average outbreak size
Basic reproductive number

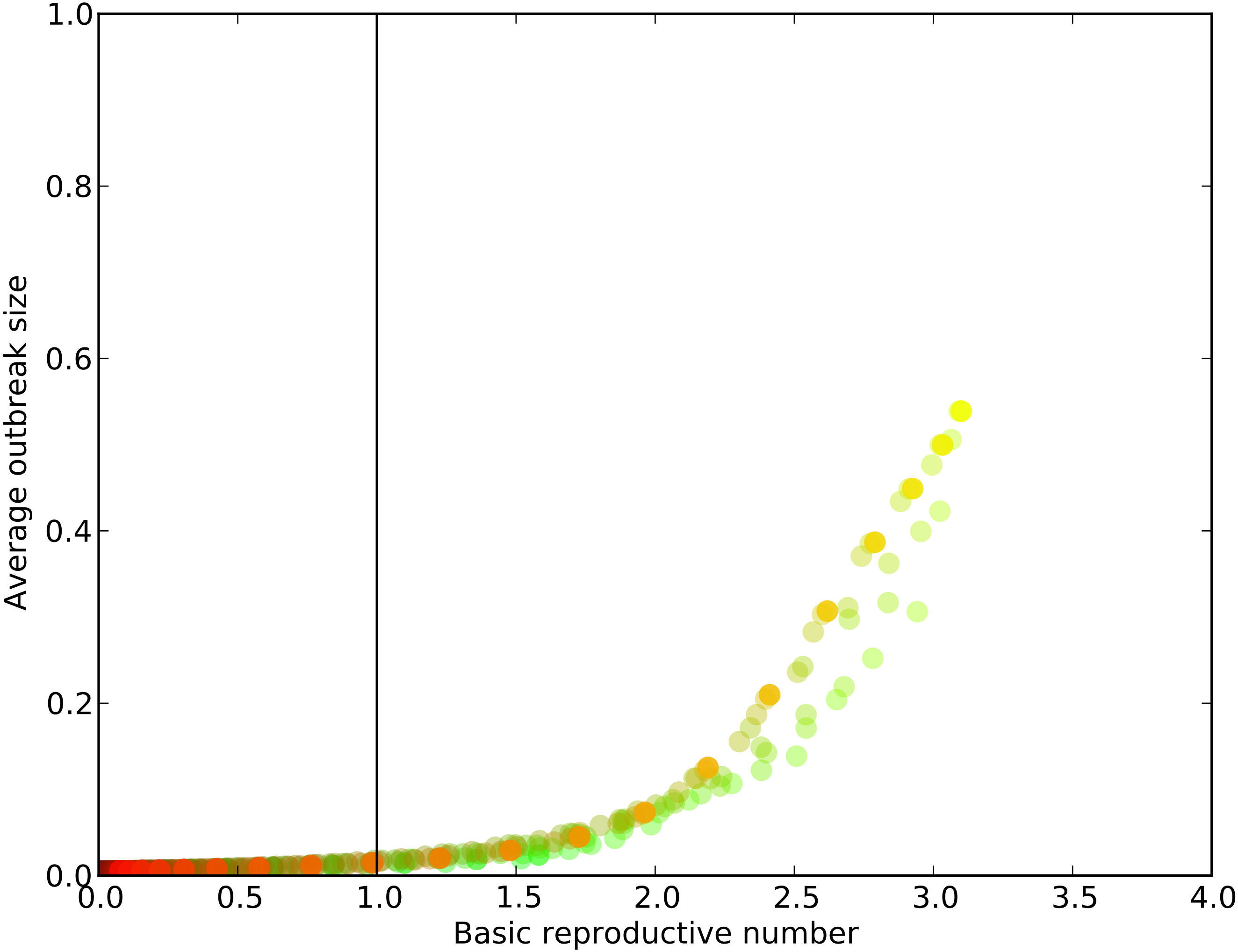

Average outbreak size
Basic reproductive number
0.0
0.2
0.4
0.6
0.8
1.0
0.5
1.5
2.0
2.5
3.0
3.5
4.0

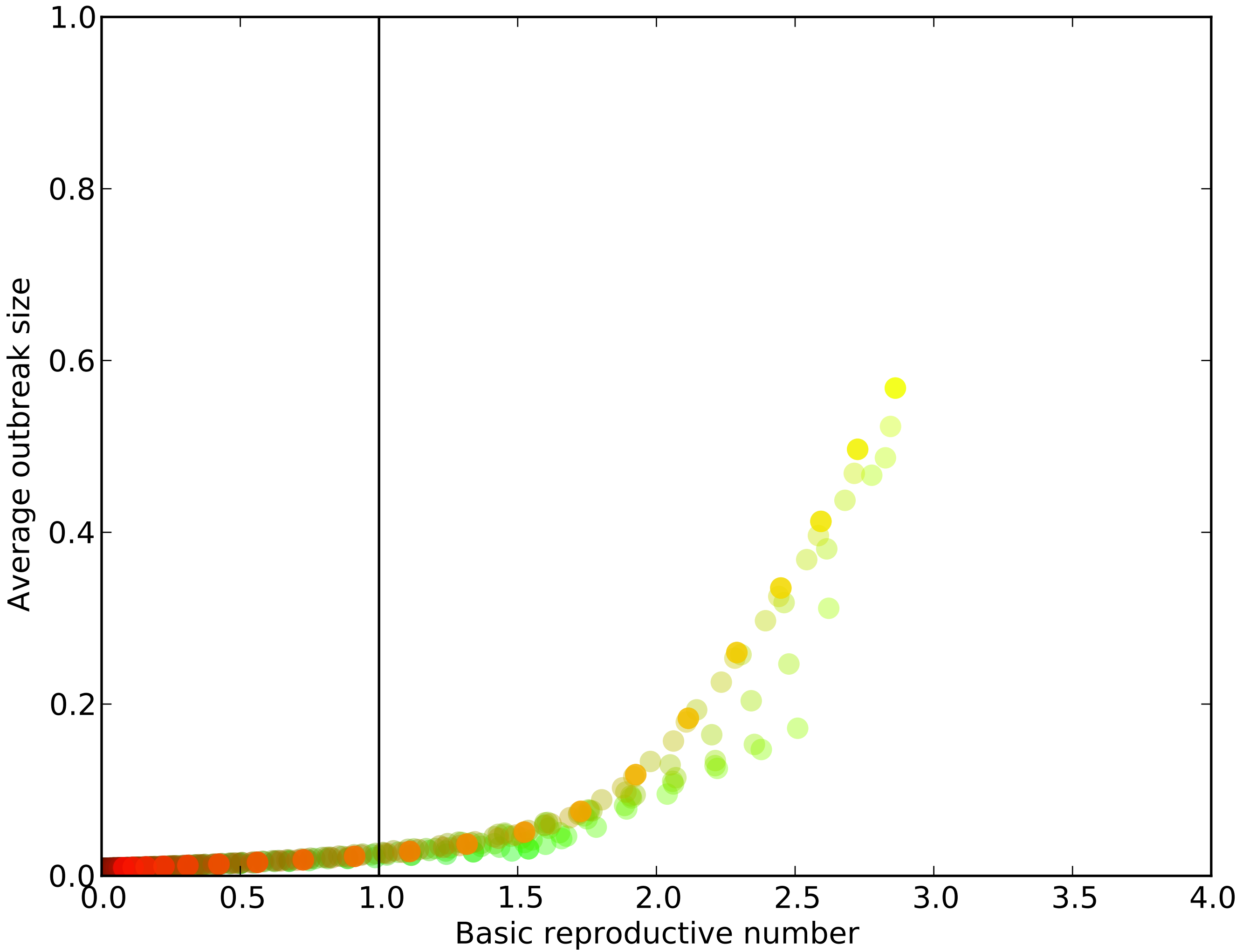

1.0
0.8
0.6
0.4
0.2
0.0
Average outbreak size
0.0
0.5
1.0
1.5
2.0
2.5
3.0
3.5
4.0
Basic reproductive number

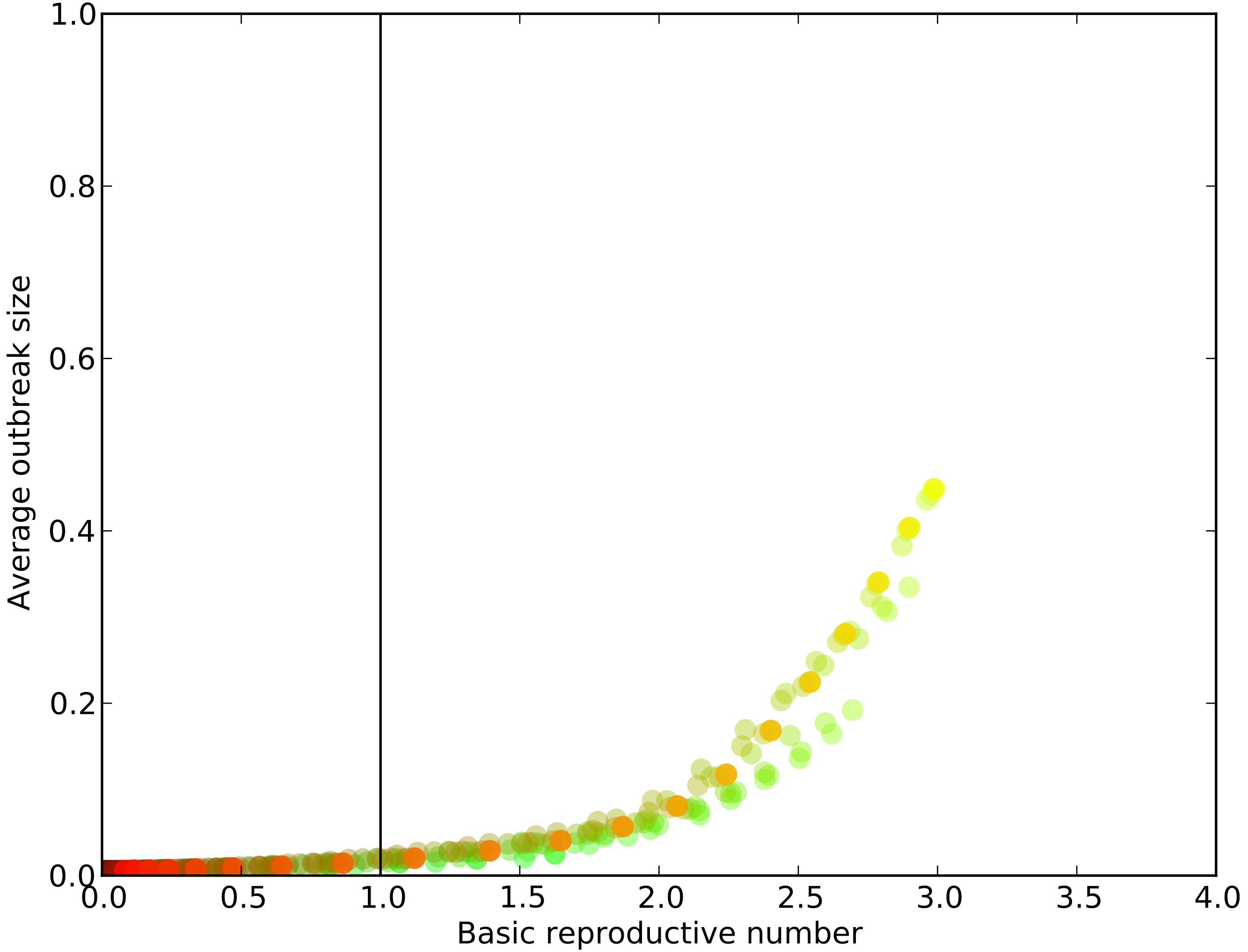

1.0
0.8
0.6
0.4
0.2
0.0
0.0
0.5
1.0
1.5
2.0
2.5
3.0
3.5
4.0
Average outbreak size
Basic reproductive number

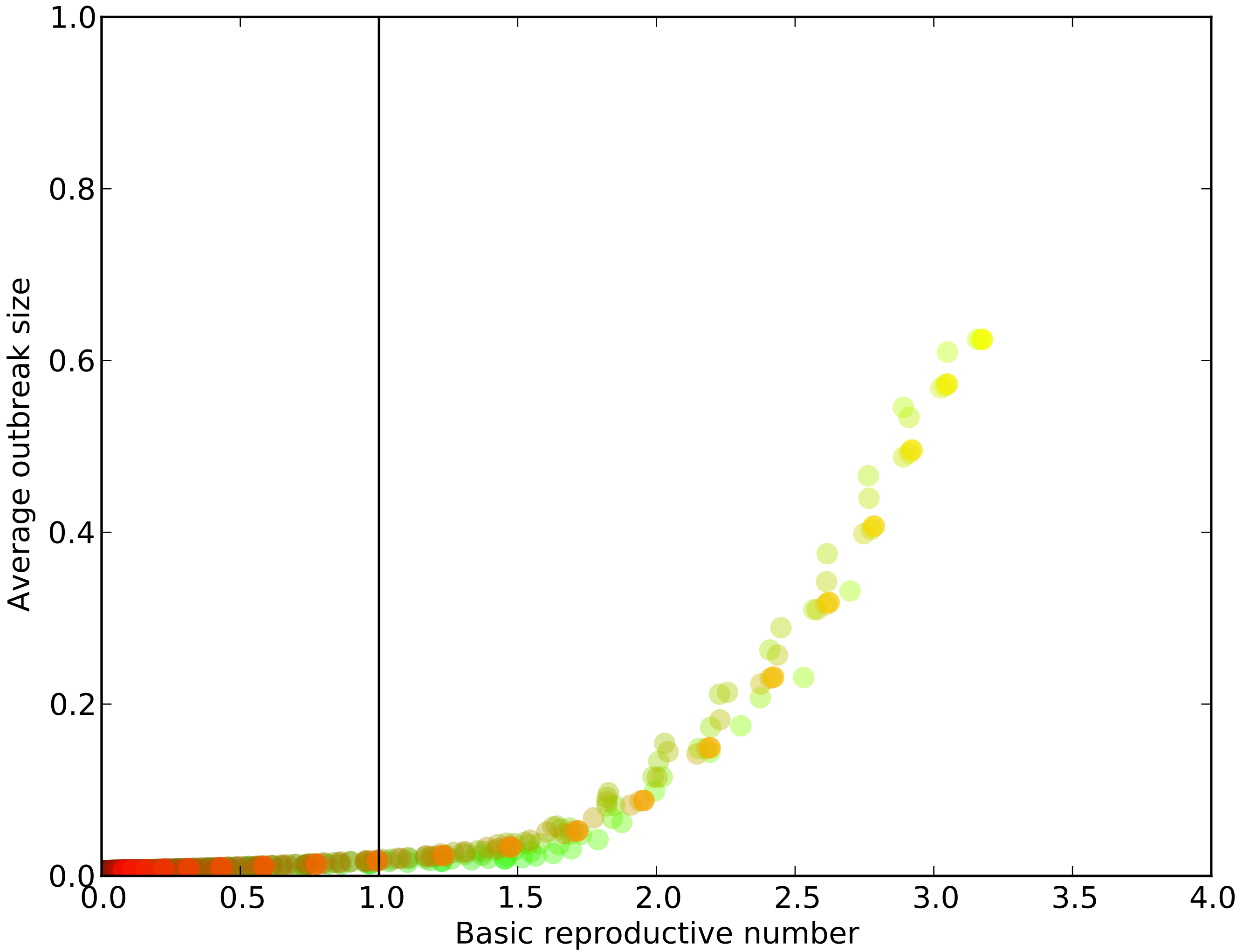

1.0
0.8
0.6
0.4
0.2
0.0
0.0
0.5
1.0
1.5
2.0
2.5
3.0
3.5
4.0
Average outbreak size
Basic reproductive number

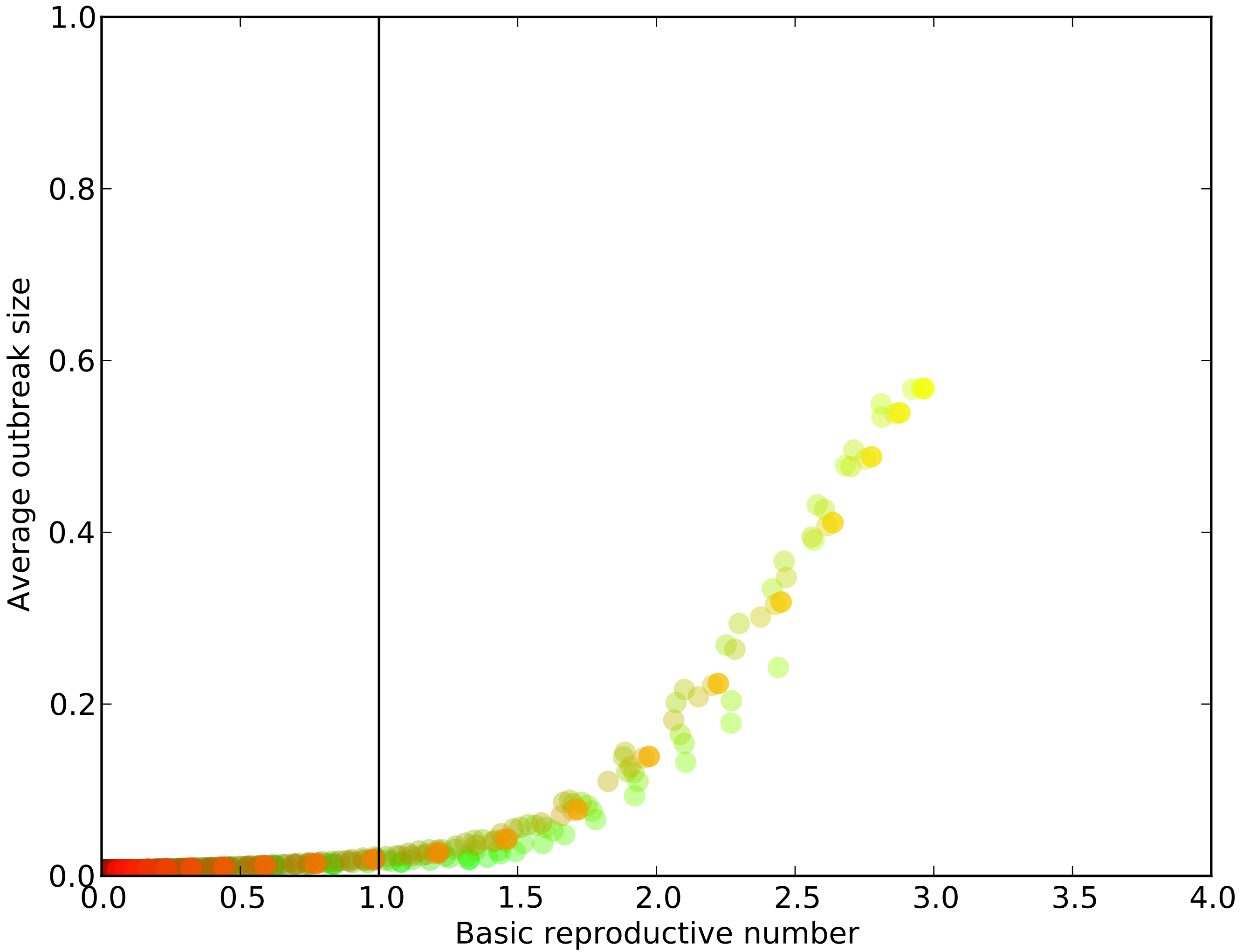

1.0
0.8
0.6
0.4
0.2
0.0
Average outbreak size
0.0
0.5
1.0
1.5
2.0
2.5
3.0
3.5
4.0
Basic reproductive number

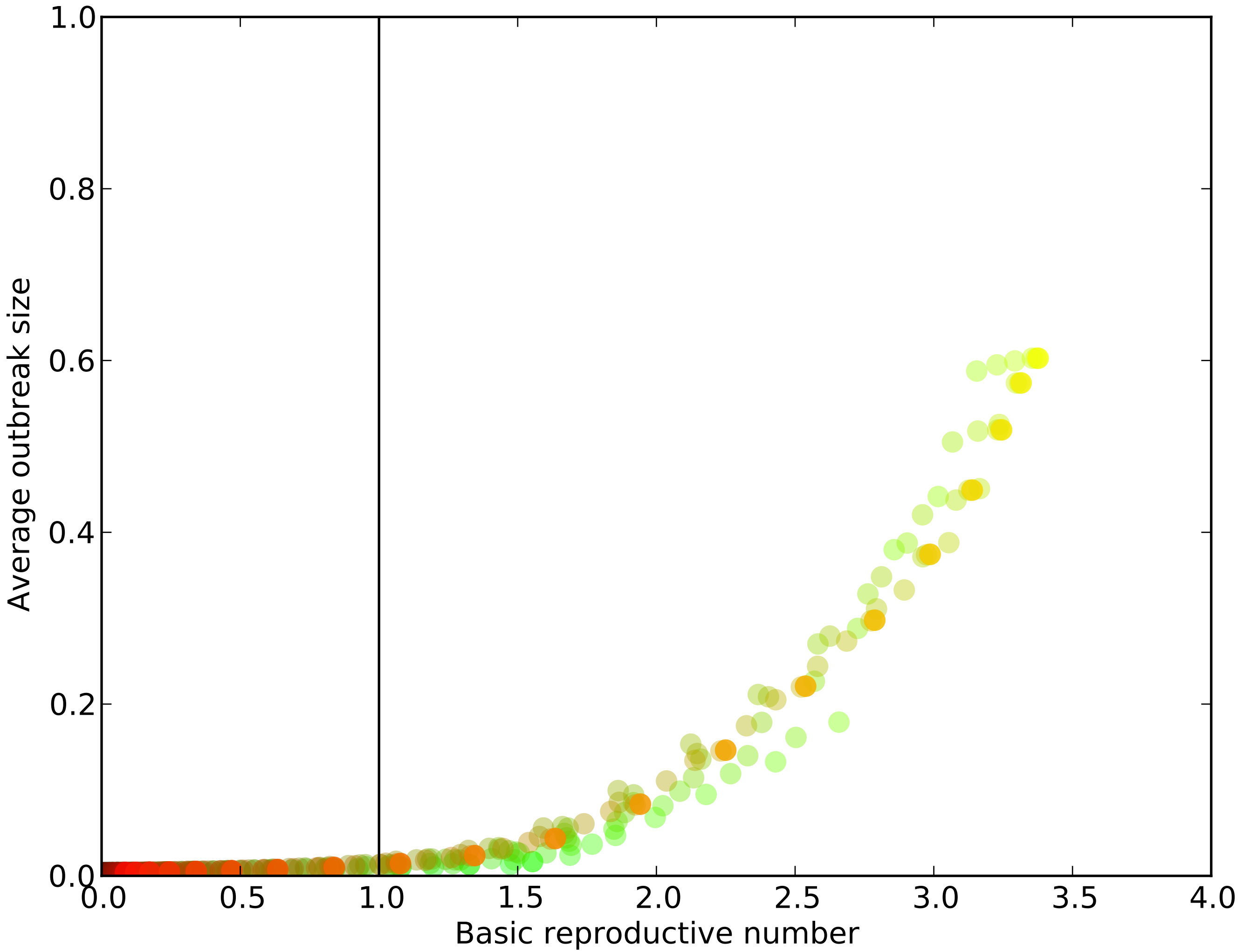

1.0
0.8
0.6
0.4
0.2
0.0
Average outbreak size
0.0
0.5
1.0
1.5
2.0
2.5
3.0
3.5
4.0
Basic reproductive number

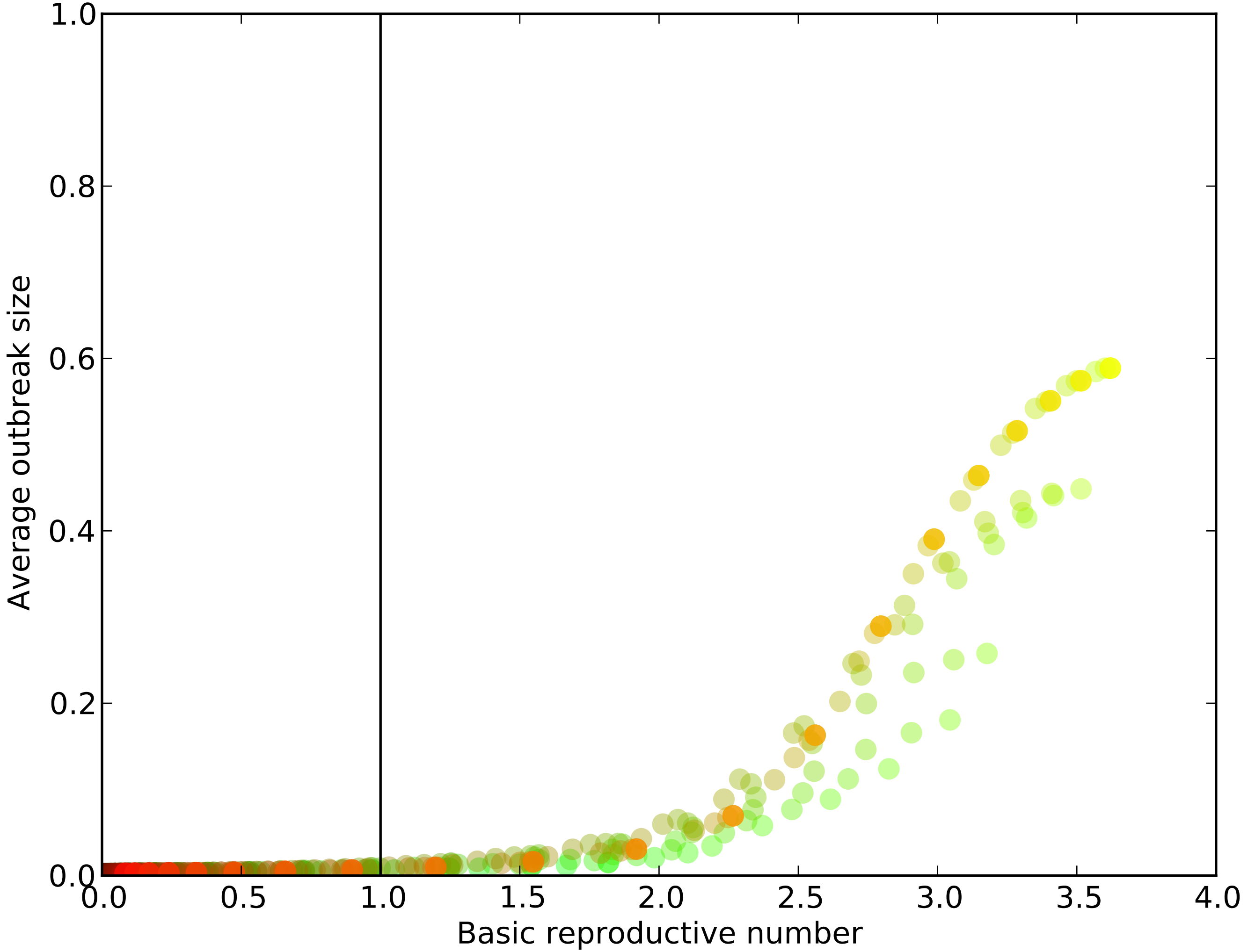
Average outbreak size
Basic reproductive number
0.0
0.2
0.4
0.6
0.8
1.0
0.0
0.5
1.0
1.5
2.0
2.5
3.0
3.5
4.0

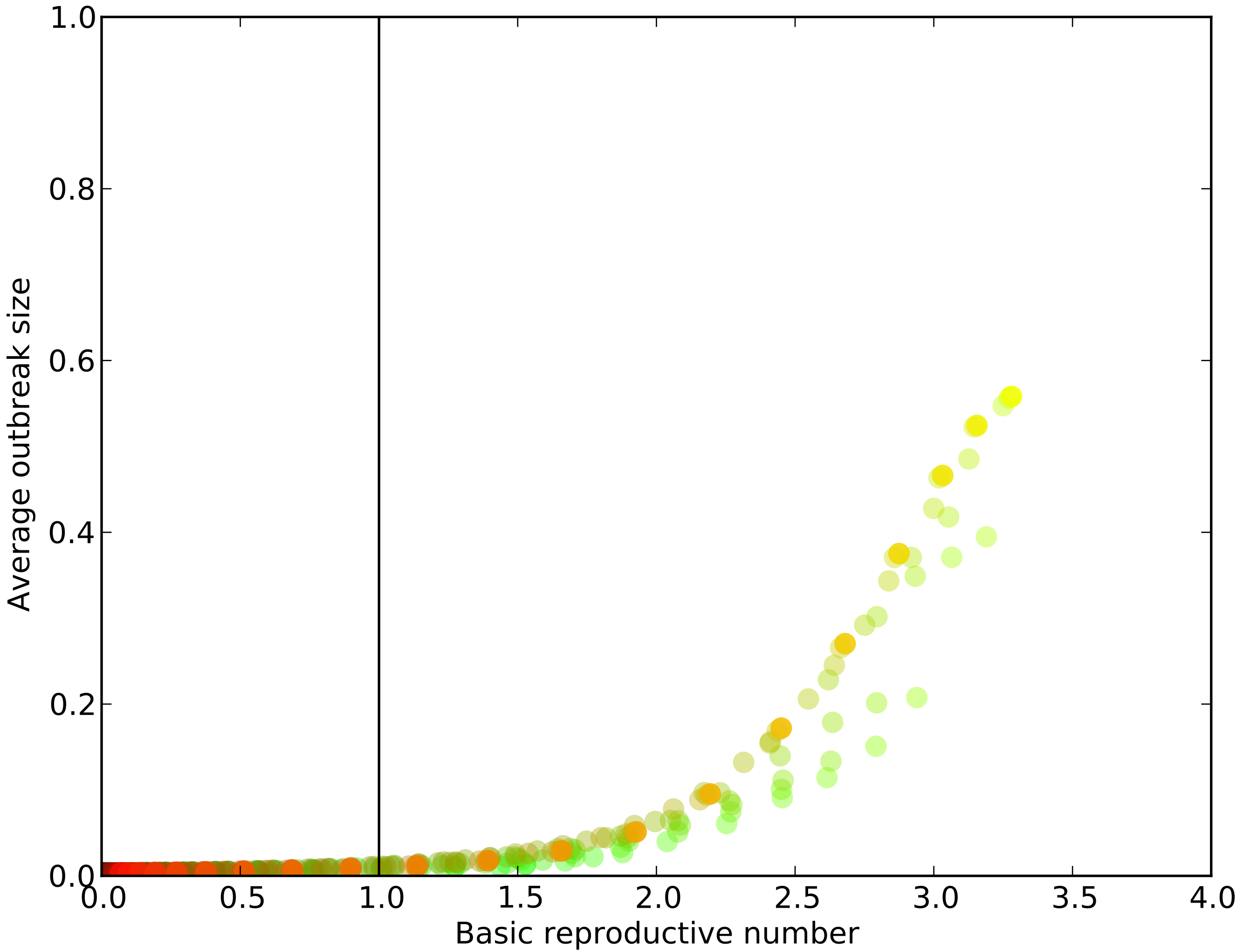

1.0
0.8
0.6
0.4
0.2
0.0
0.0
0.5
1.0
1.5
2.0
2.5
3.0
3.5
4.0
Average outbreak size
Basic reproductive number

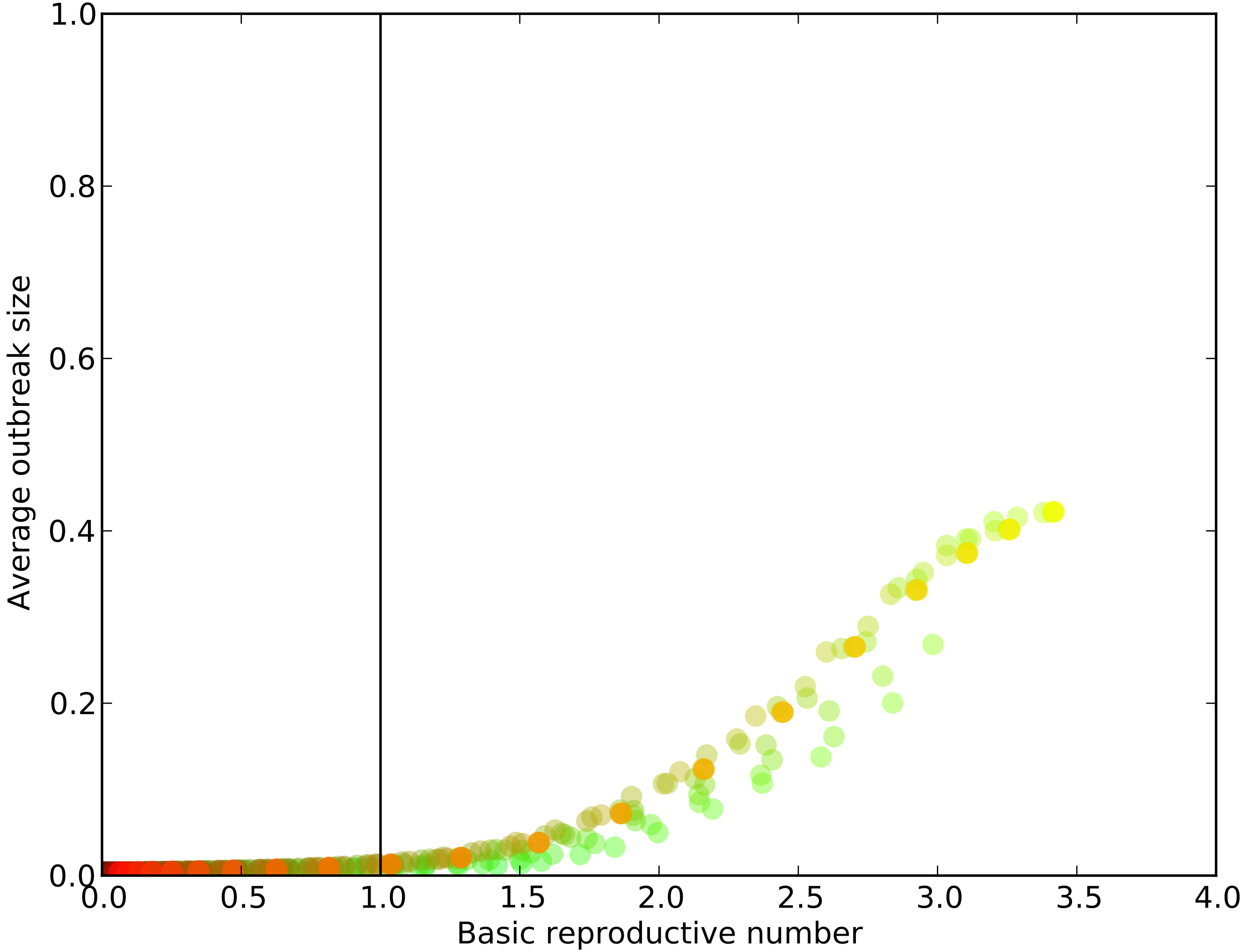
1.0
0.8
0.6
0.4
0.2
0.0
Average outbreak size
0.0
0.5
1.0
1.5
2.0
2.5
3.0
3.5
4.0
Basic reproductive number
