# Supplementary material for: The Basic Reproduction Number as a Predictor for Epidemic Outbreaks in Temporal Networks
Source: PLoS One. 2015 Mar 20;10(3):e0120567. doi: 10.1371/journal.pone.0120567 (PMC4368036; doi:10.1371/journal.pone.0120567)
Supplement: S2 Fig — The numbers identify the data sets as follows: Conference (1), Dating (2), E-mail 1 (3), E-mail 2 (4), Facebook (5), Forum (6), Gallery (7), Hospital (8), Online community (9), Prostitution (10), Reality mining (11), School (12). (PDF) [file pone.0120567.s002.pdf]

The basic reproductive number as a predictor for epidemic outbreaks in temporal networks

Supporting Information 2, Scatter plots of shape descriptors and measures of temporal network structure

Petter Holme, Naoki Masuda

Index of datasets:

1. *Prostitution*
2. *E-mail 2*
3. *Facebook*
4: *Online community*
5: *E-mail*
6: *Online forum*
7. *Dating*
8. *Conference*
9. *Hospital*
10. *Reality mining*
11. *School*
12. *Gallery*

$\tau_{R_0}\Omega$

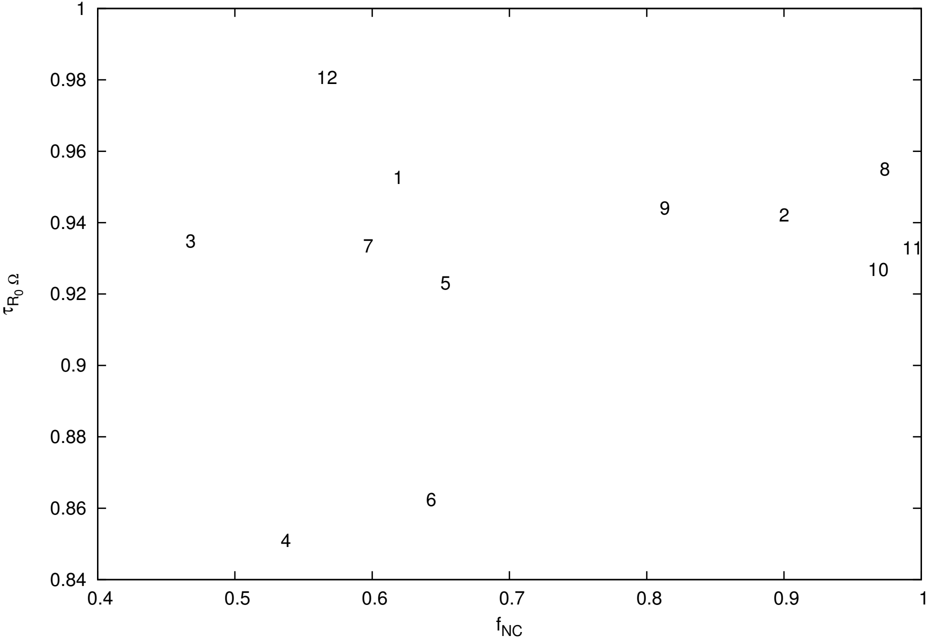

1
0.98
0.96
0.94
0.92
0.9
0.88
0.86
0.84
0.4
0.5
0.6
0.7
0.8
0.9
1
$\tau_{R_0}\Omega$
f_{NC}
12
1
8
9
2
3
7
11
10
5
6
4

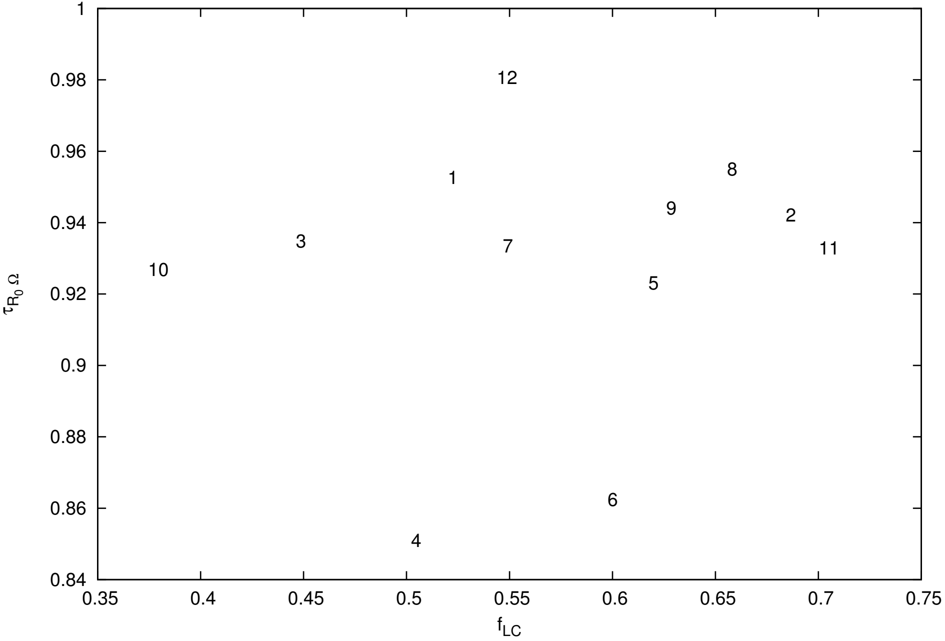
1
0.98
0.96
0.94
0.92
0.9
0.88
0.86
0.84
0.35
0.4
0.45
0.5
0.55
0.6
0.65
0.7
0.75
$\tau_{R_0\,\Omega}$
f_{LC}
12
1
8
9
2
3
7
11
10
5
6
4

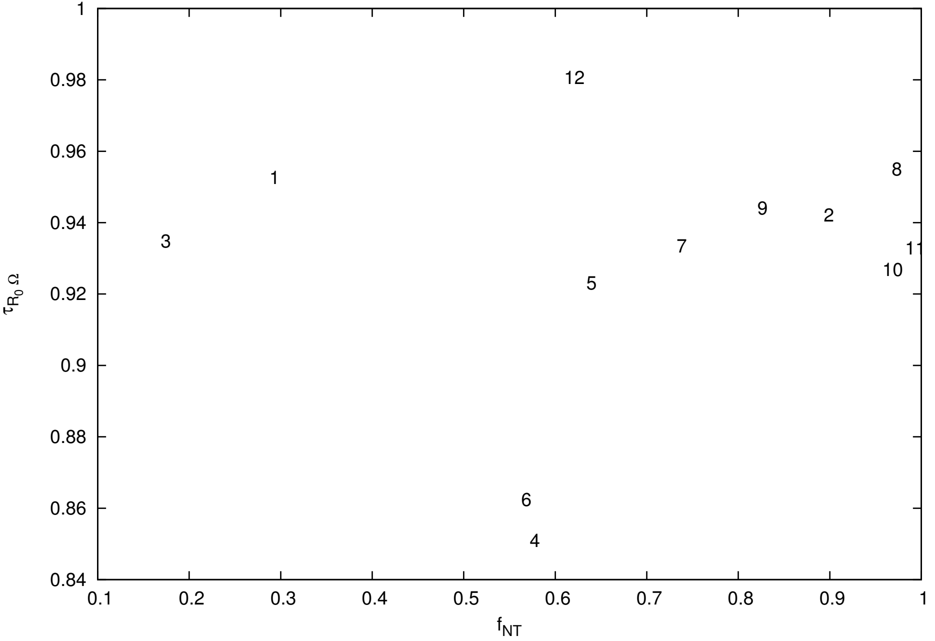

1
0.98
0.96
0.94
0.92
0.9
0.88
0.86
0.84
0.1
0.2
0.3
0.4
0.5
0.6
0.7
0.8
0.9
1
$\tau_{R_0\,\Omega}$
f_{NT}
12
1
3
8
9
2
7
11
10
5
6
4

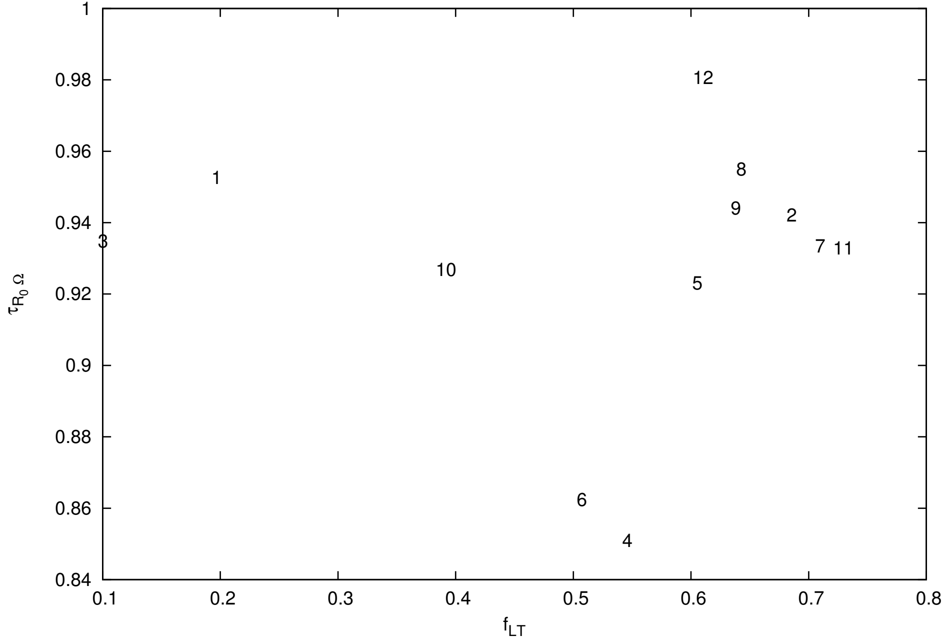

$\tau_{R_0\,\Omega}$
f_{LT}
1
0.98
0.96
0.94
0.92
0.9
0.88
0.86
0.84
0.1
0.2
0.3
0.4
0.5
0.6
0.7
0.8
1
2
3
4
5
6
7
8
9
10
11
12

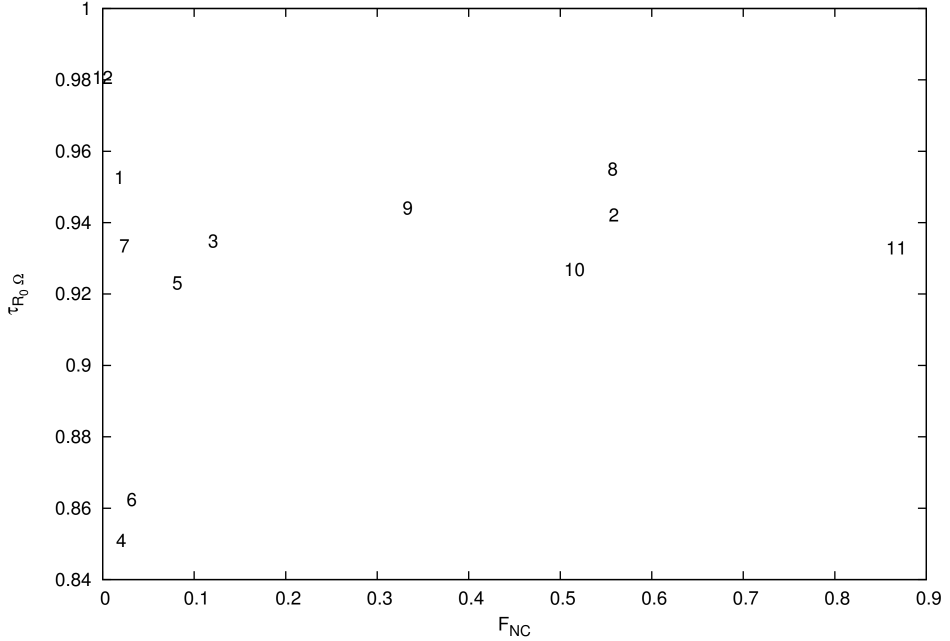

$\tau_{R_0 \Omega}$
F_{NC}
1
0.98
0.96
0.94
0.92
0.9
0.88
0.86
0.84
0
0.1
0.2
0.3
0.4
0.5
0.6
0.7
0.8
0.9
12
1
7
3
5
9
8
2
10
11
6
4

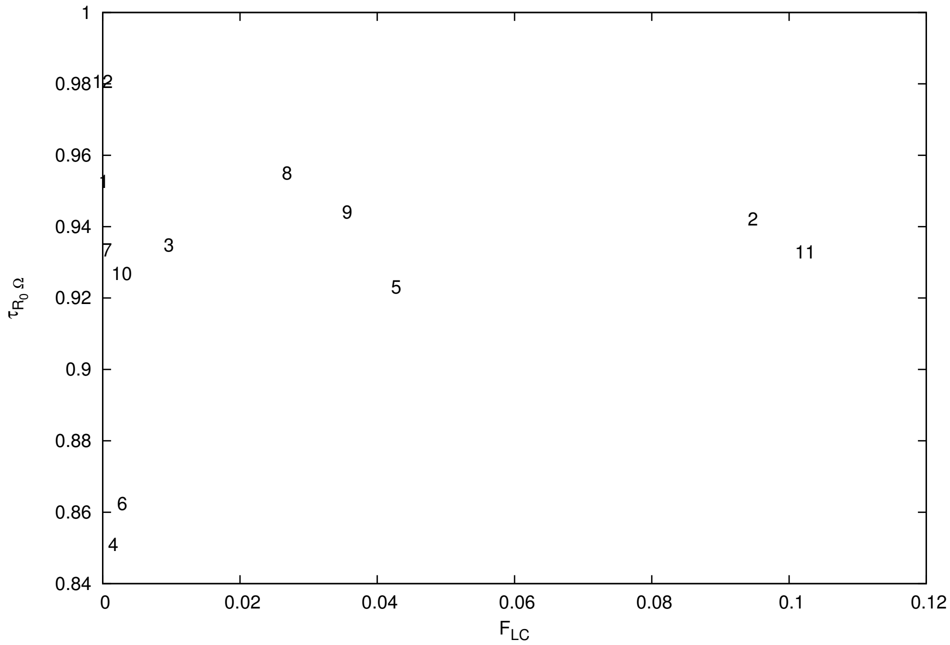

1
0.98
0.96
0.94
0.92
0.9
0.88
0.86
0.84
0
0.02
0.04
0.06
0.08
0.1
0.12
$\tau_{R_0\,\Omega}$
F_{LC}
12
1
8
9
2
7
3
10
11
5
6
4

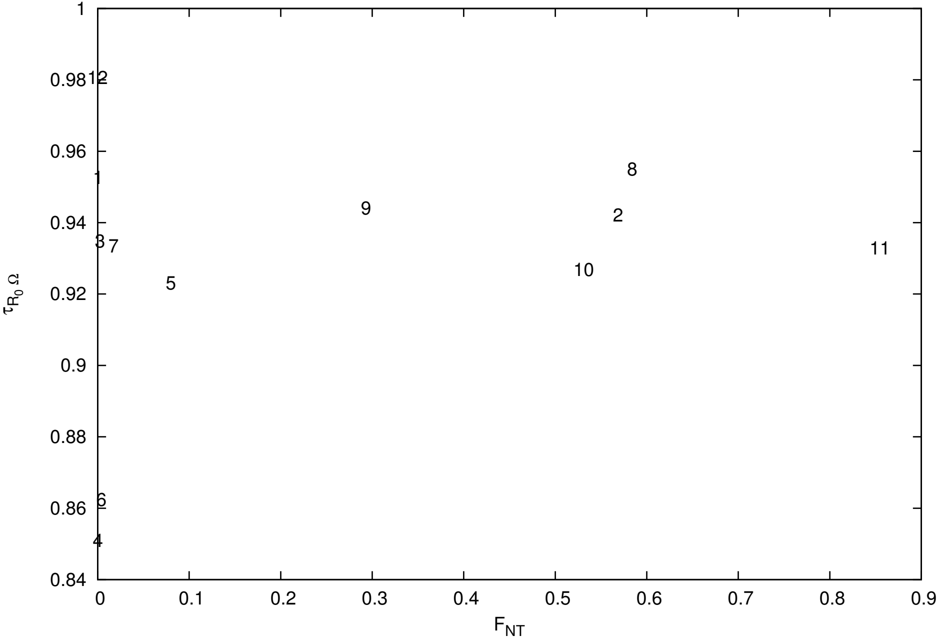

1
0.98
0.96
0.94
0.92
0.9
0.88
0.86
0.84
0
0.1
0.2
0.3
0.4
0.5
0.6
0.7
0.8
0.9
$\tau_{R_0\,\Omega}$
F_{NT}
12
1
3
7
5
9
10
2
8
11
6
4

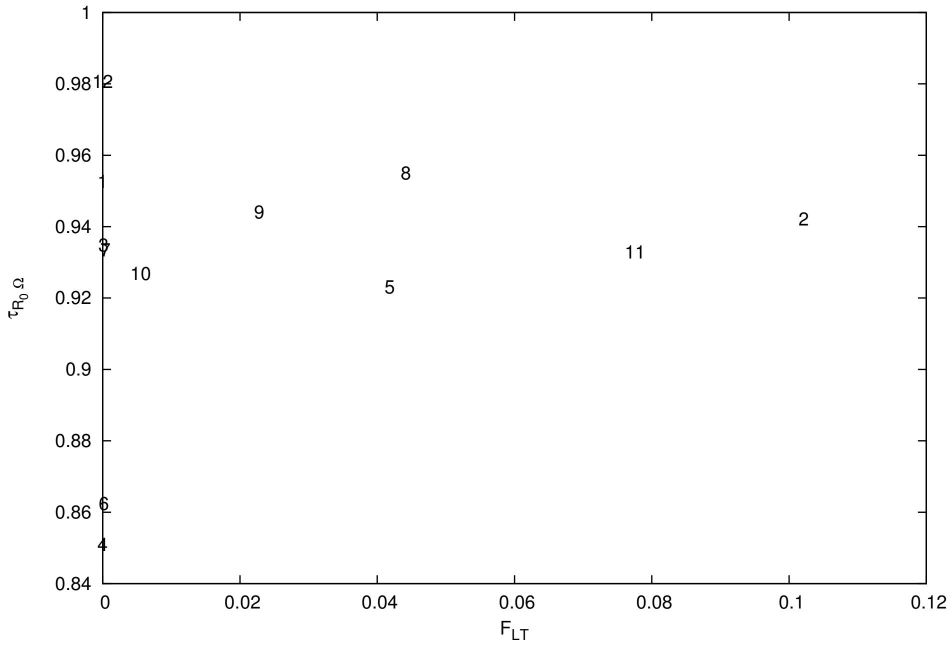

1
0.98
0.96
0.94
0.92
0.9
0.88
0.86
0.84
0
0.02
0.04
0.06
0.08
0.1
0.12
$\tau_{R_0\,\Omega}$
F_{LT}
12
1
8
9
2
3
7
11
10
5
6
4

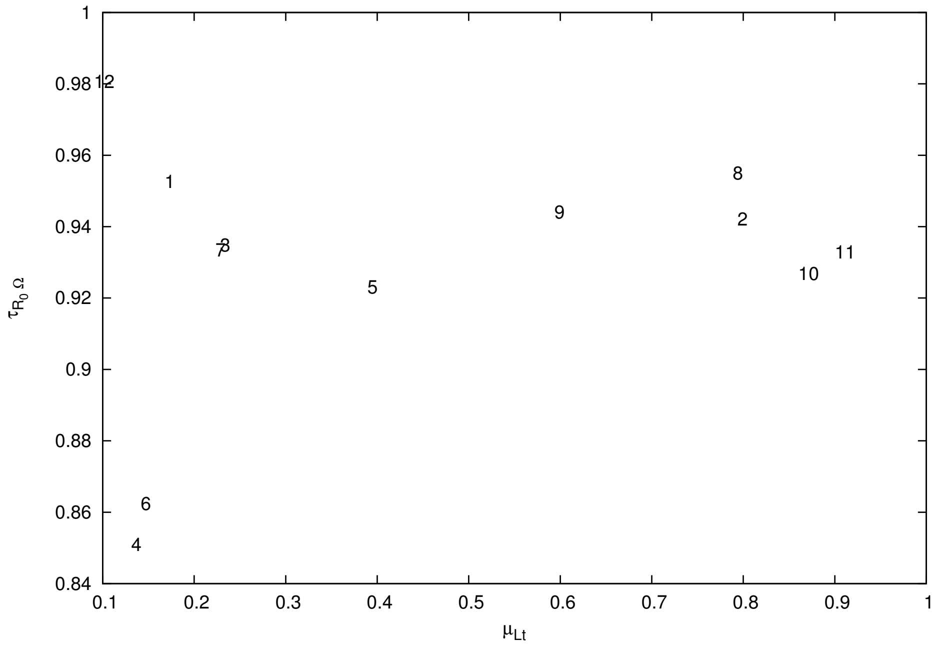

1
0.98
0.96
0.94
0.92
0.9
0.88
0.86
0.84
0.1
0.2
0.3
0.4
0.5
0.6
0.7
0.8
0.9
1
$\tau_{R_0\,\Omega}$
μ_{Lt}
12
1
3
7
5
9
8
2
11
10
6
4

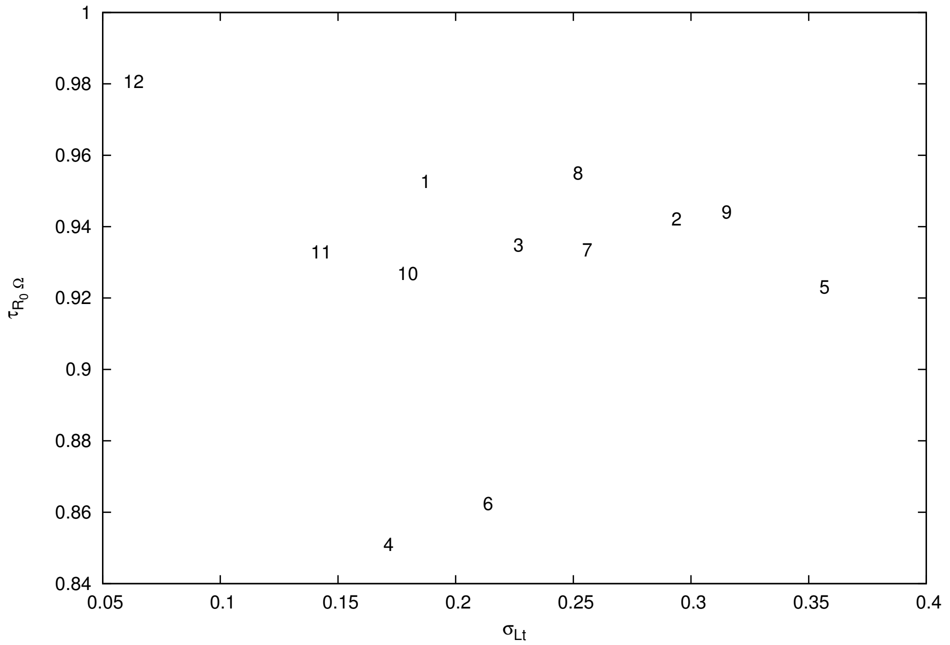

1
0.98
0.96
0.94
0.92
0.9
0.88
0.86
0.84
0.05
0.1
0.15
0.2
0.25
0.3
0.35
0.4
$\tau_{R_0\,\Omega}$
σ_{Lt}
12
1
8
2
9
11
10
3
7
5
6
4

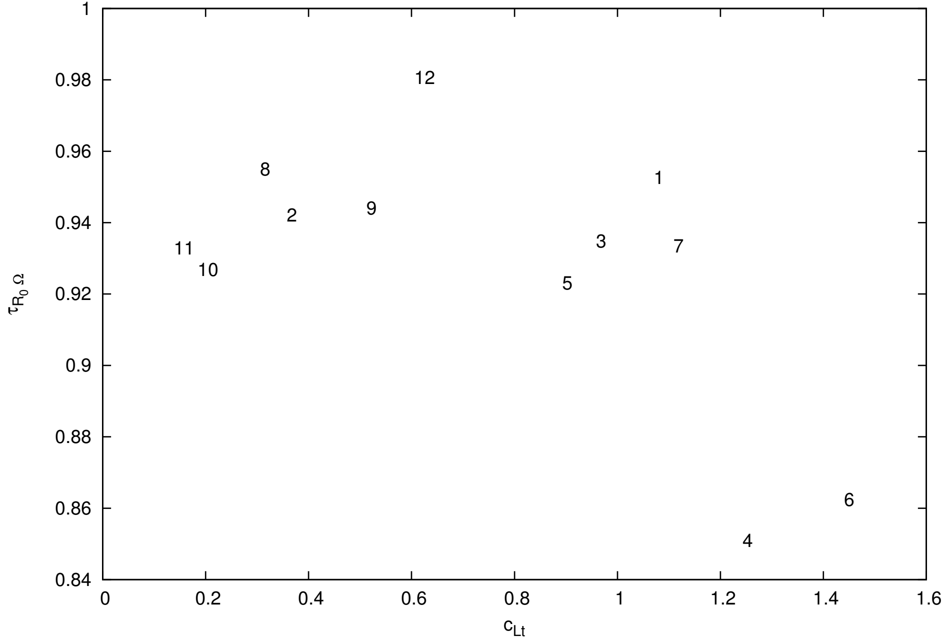

1
0.98
0.96
0.94
0.92
0.9
0.88
0.86
0.84
0
0.2
0.4
0.6
0.8
1
1.2
1.4
1.6
$\tau_{R_0\,\Omega}$
c_{Lt}
12
8
1
9
2
3
7
11
10
5
6
4

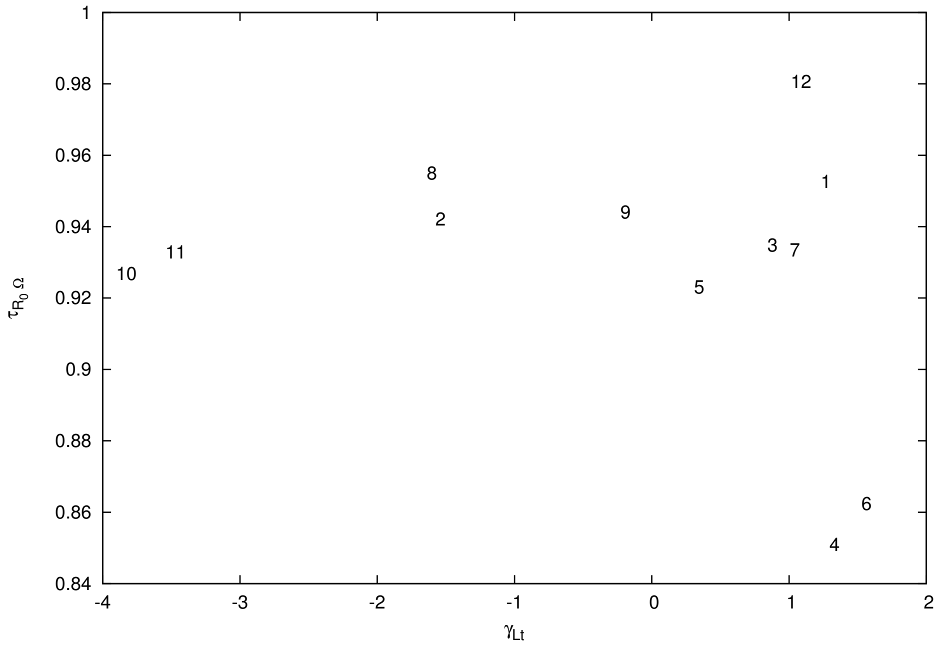

1
0.98
0.96
0.94
0.92
0.9
0.88
0.86
0.84
-4
-3
-2
-1
0
1
2
$\tau_{R_0\,\Omega}$
γ_{Lt}
12
8
1
9
2
11
3
7
10
5
6
4

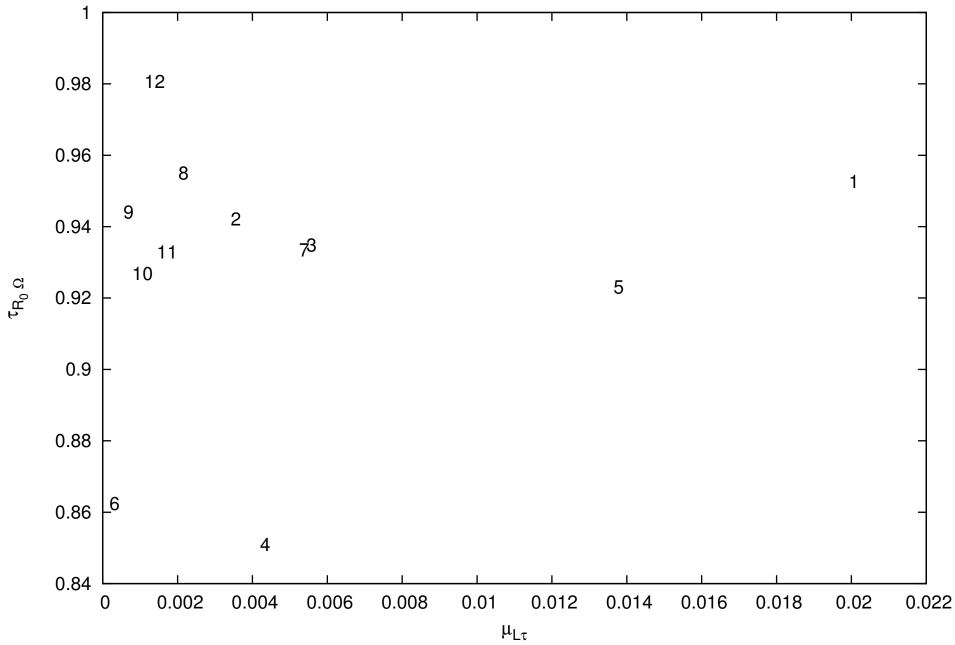

1
0.98
0.96
0.94
0.92
0.9
0.88
0.86
0.84
0
0.002
0.004
0.006
0.008
0.01
0.012
0.014
0.016
0.018
0.02
0.022
$\tau_{R_0\,\Omega}$
$\mu_{L\tau}$
12
8
1
9
2
7
3
11
10
5
6
4

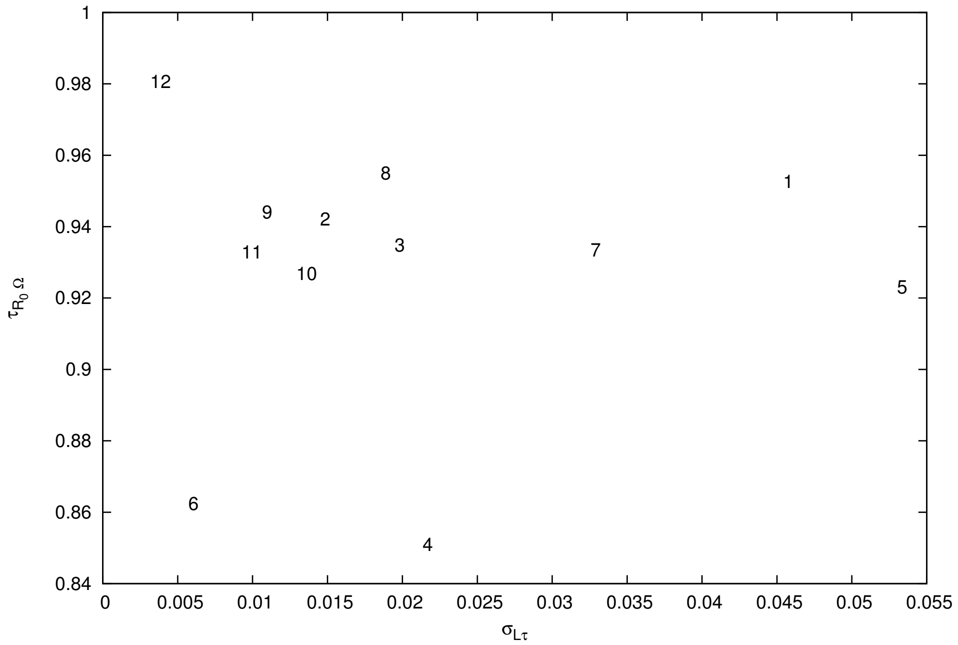

$\tau_{R_0\,\Omega}$
$\sigma_{L\tau}$
0.84
0.86
0.88
0.9
0.92
0.94
0.96
0.98
1
0
0.005
0.01
0.015
0.02
0.025
0.03
0.035
0.04
0.045
0.05
0.055
12
8
1
9
2
3
7
11
10
5
6
4

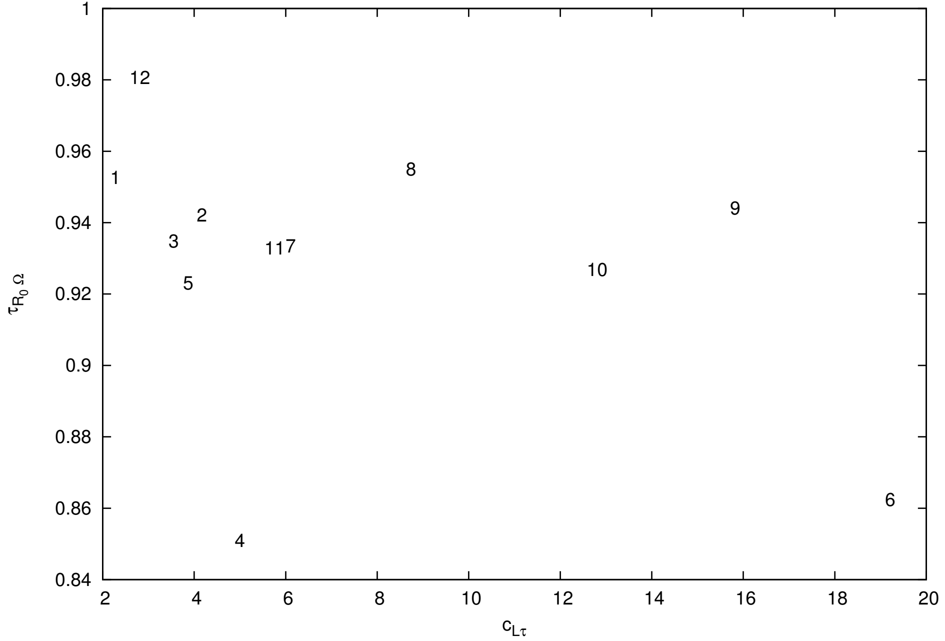

1
0.98
0.96
0.94
0.92
0.9
0.88
0.86
0.84
2
4
6
8
10
12
14
16
18
20
$\tau_{R_0 \Omega}$
$c_{L\tau}$
12
1
8
2
9
3
117
10
5
6
4

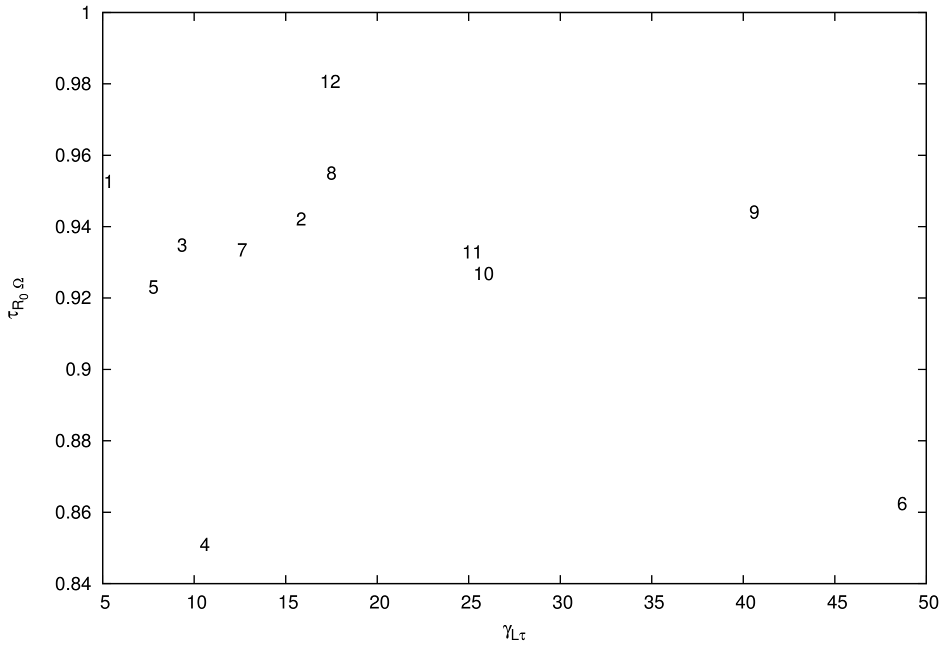

1
0.98
0.96
0.94
0.92
0.9
0.88
0.86
0.84
5
10
15
20
25
30
35
40
45
50
$\tau_{R_0\,\Omega}$
$\gamma_{L\tau}$
1
2
3
4
5
6
7
8
9
10
11
12

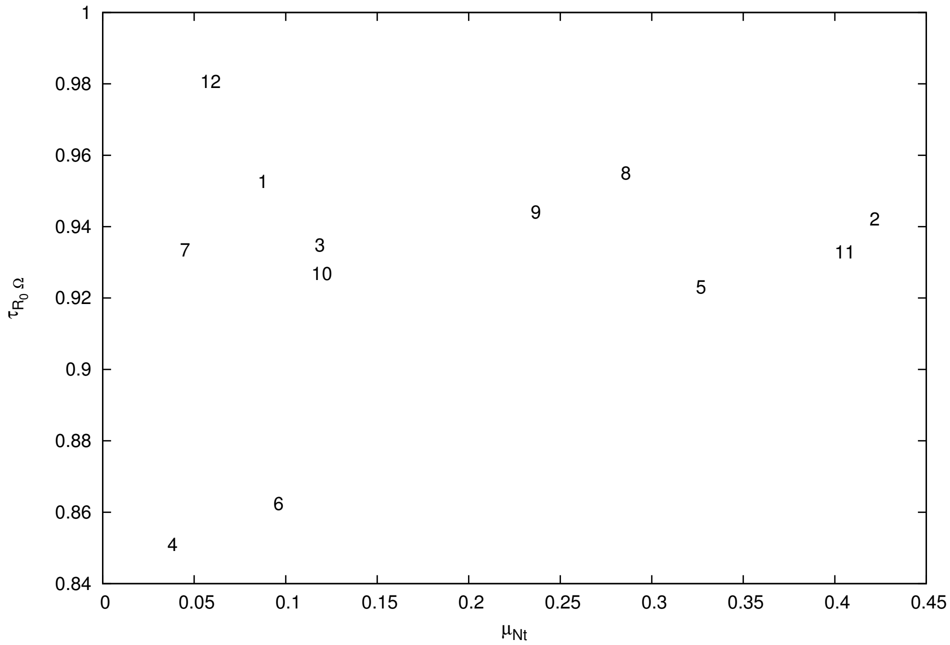

1
0.98
0.96
0.94
0.92
0.9
0.88
0.86
0.84
0
0.05
0.1
0.15
0.2
0.25
0.3
0.35
0.4
0.45
$\tau_{R_0\,\Omega}$
μ_{Nt}
12
1
8
9
2
7
3
11
10
5
6
4

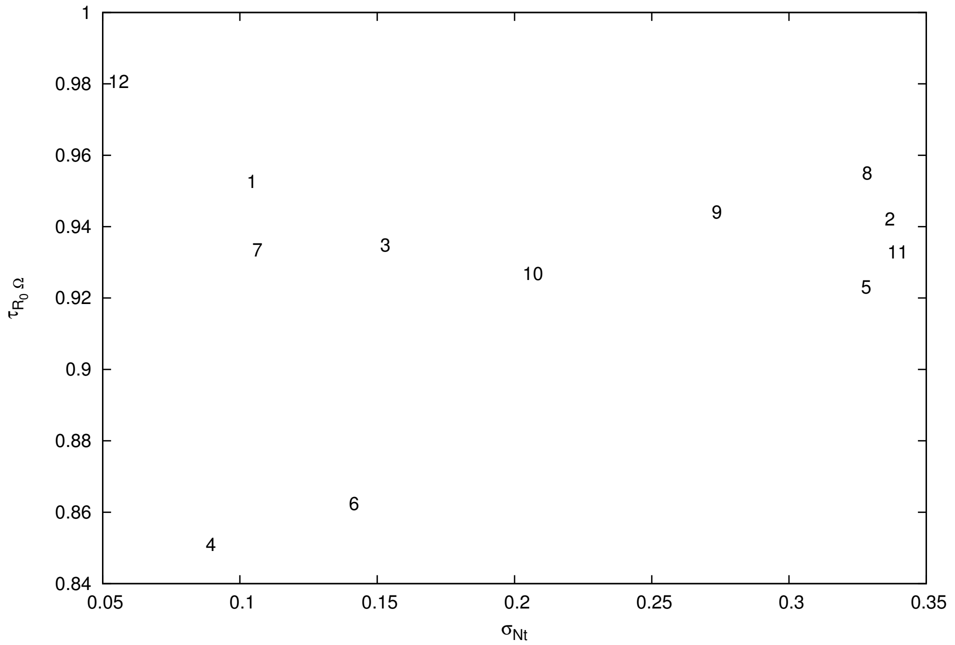

1
0.98
0.96
0.94
0.92
0.9
0.88
0.86
0.84
0.05
0.1
0.15
0.2
0.25
0.3
0.35
$\tau_{R_0\,\Omega}$
σ_{Nt}
12
1
7
3
6
4
10
9
8
2
11
5

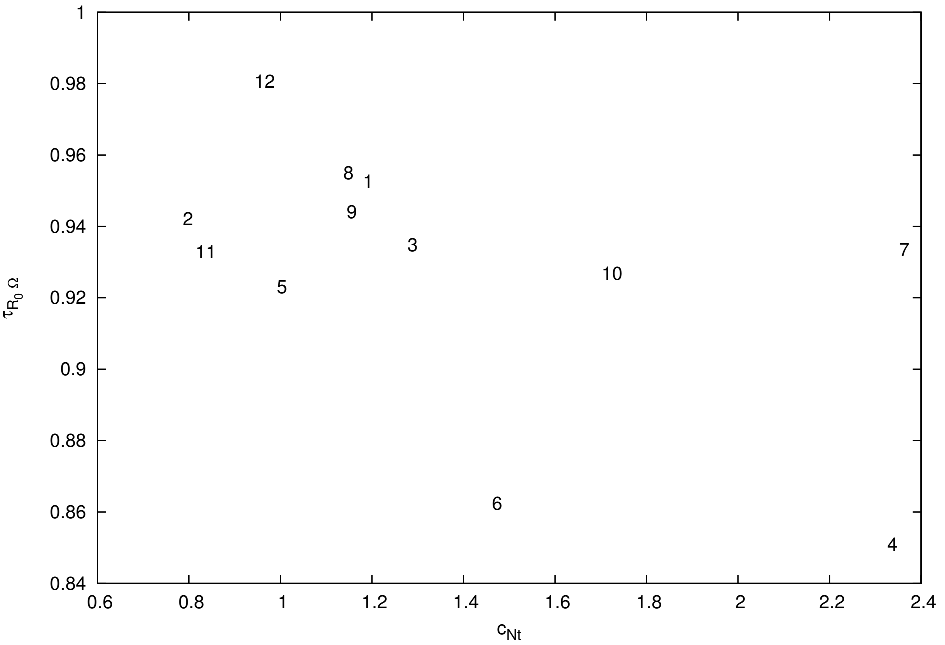

1
0.98
0.96
0.94
0.92
0.9
0.88
0.86
0.84
0.6
0.8
1
1.2
1.4
1.6
1.8
2
2.2
2.4
c_{Nt}
$\tau_{R_0\,\Omega}$
12
8
1
9
2
11
3
5
10
7
6
4

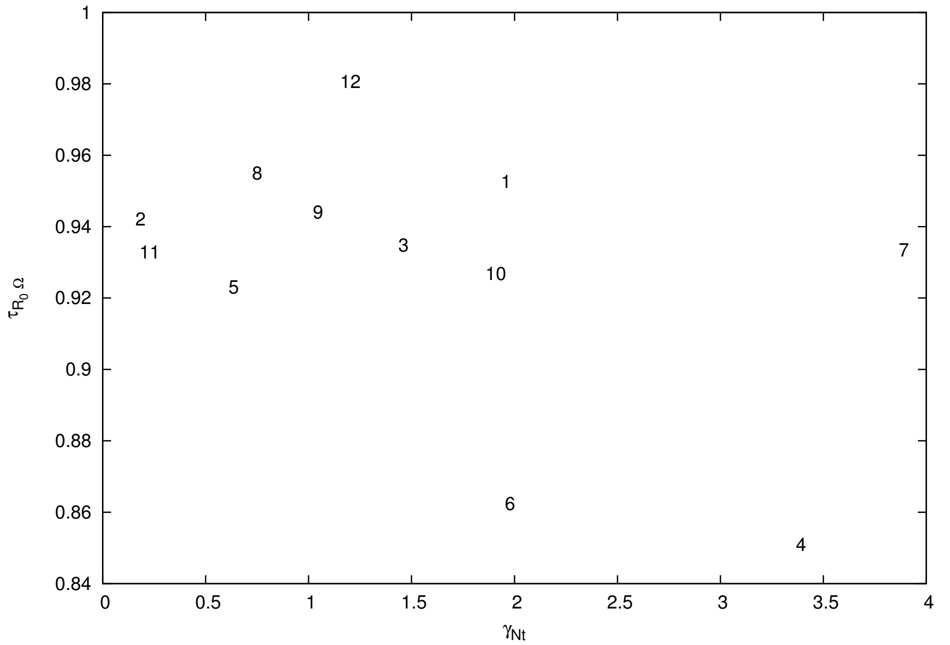

1
0.98
0.96
0.94
0.92
0.9
0.88
0.86
0.84
0
0.5
1
1.5
2
2.5
3
3.5
4
$\tau_{R_0\,\Omega}$
γ_{Nt}
12
8
1
2
9
11
3
7
10
5
6
4

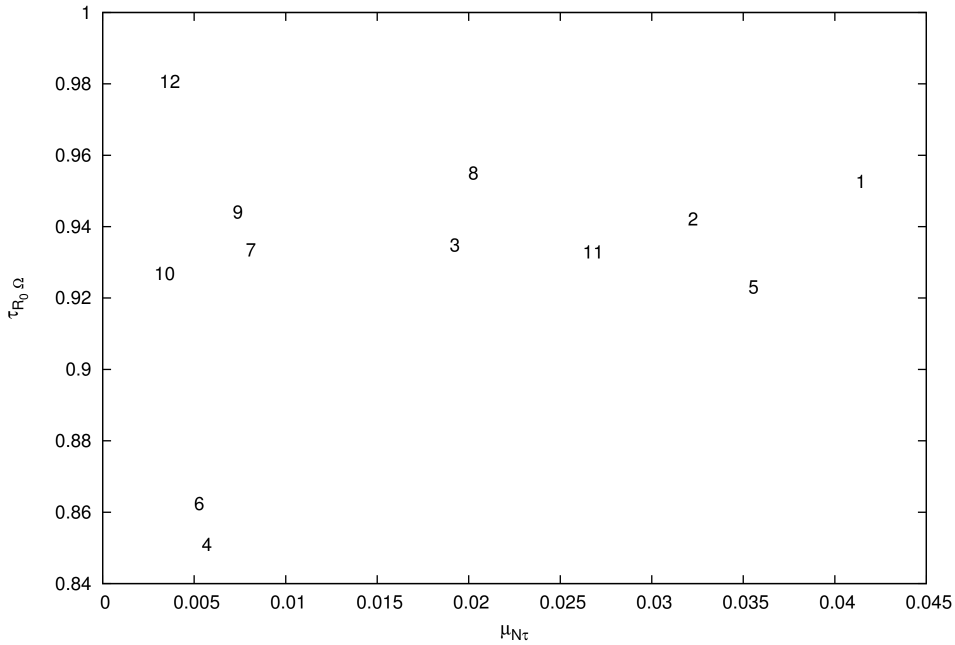

1
0.98
0.96
0.94
0.92
0.9
0.88
0.86
0.84
0
0.005
0.01
0.015
0.02
0.025
0.03
0.035
0.04
0.045
$\tau_{R_0\,\Omega}$
$\mu_{N\tau}$
12
8
1
9
2
7
3
11
10
5
6
4

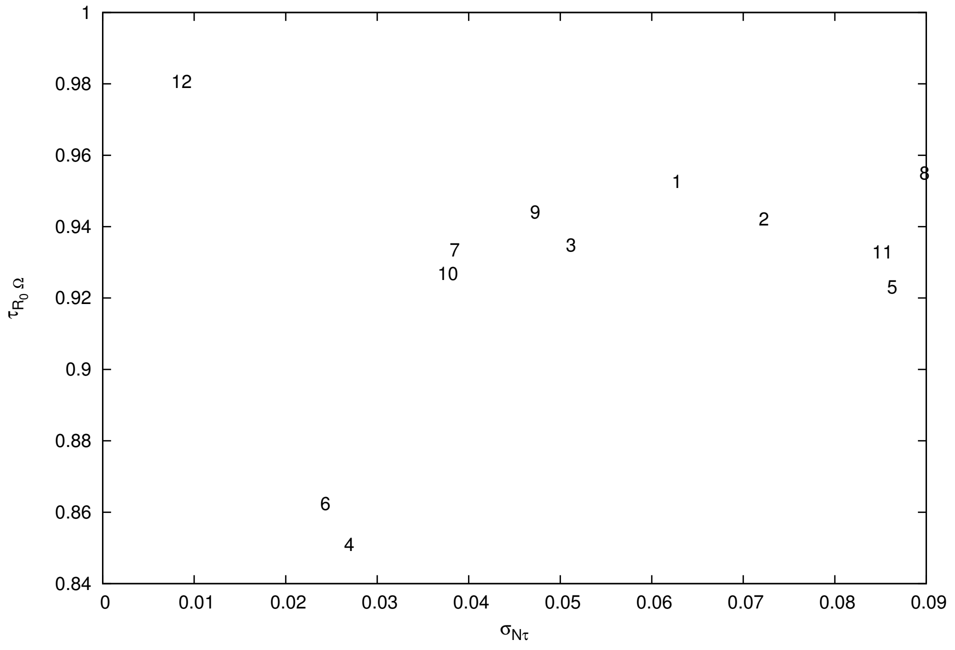

1
0.98
0.96
0.94
0.92
0.9
0.88
0.86
0.84
0
0.01
0.02
0.03
0.04
0.05
0.06
0.07
0.08
0.09
$\tau_{R_0\,\Omega}$
$\sigma_{N\tau}$
12
1
8
9
2
7
3
11
10
5
6
4

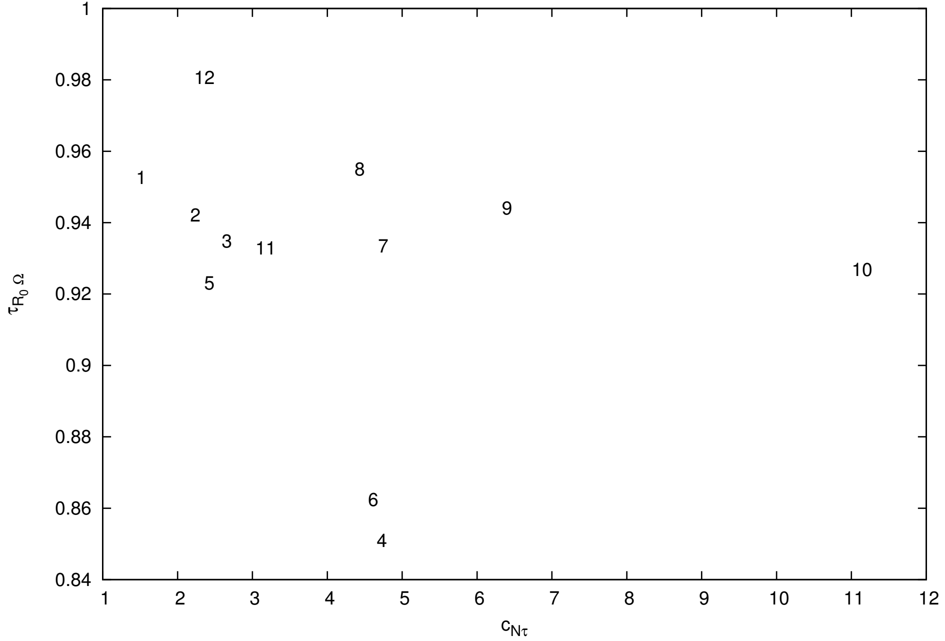

1
0.98
0.96
0.94
0.92
0.9
0.88
0.86
0.84
1
2
3
4
5
6
7
8
9
10
11
12
$\tau_{R_0\,\Omega}$
$c_{N\tau}$
12
1
8
2
9
3
11
7
5
10
6
4

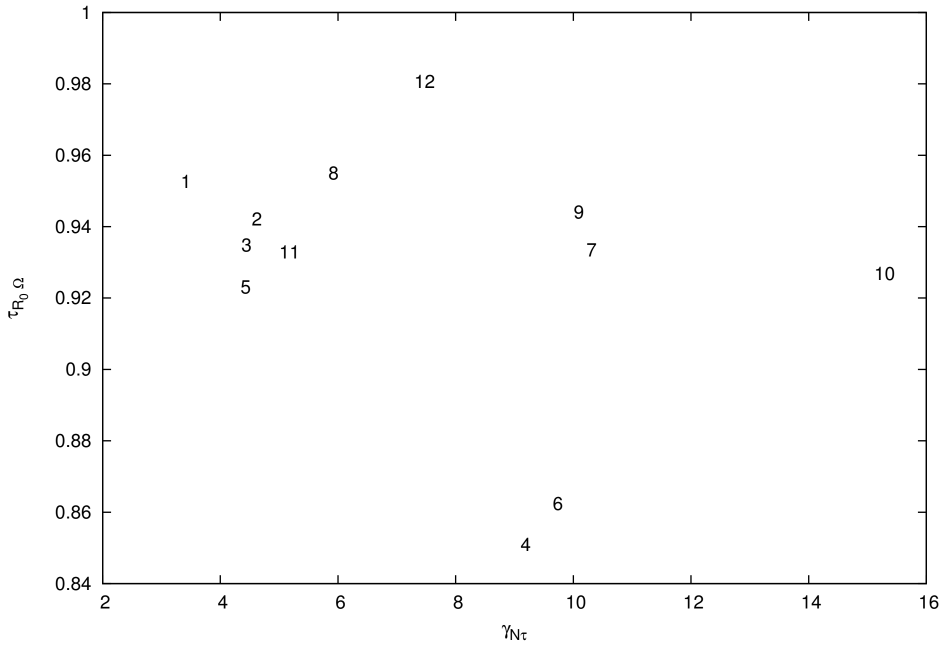

1
0.98
0.96
0.94
0.92
0.9
0.88
0.86
0.84
2
4
6
8
10
12
14
16
$\tau_{R_0\,\Omega}$
$\gamma_{N\tau}$
12
8
1
9
2
3
11
7
10
5
6
4

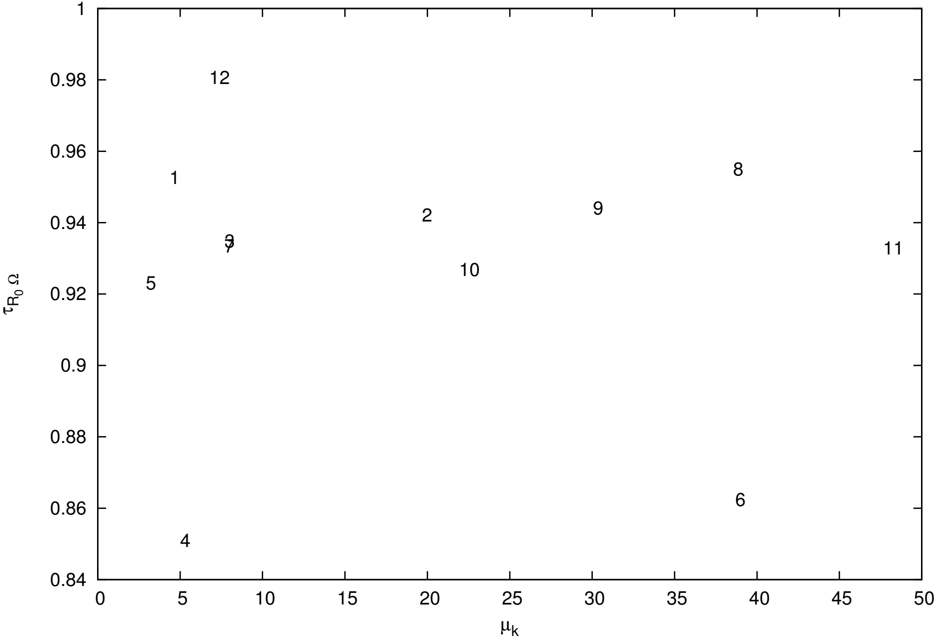

1
0.98
0.96
0.94
0.92
0.9
0.88
0.86
0.84
0
5
10
15
20
25
30
35
40
45
50
μ_k
$\tau_{R_0\,\Omega}$
12
1
8
9
2
3
7
11
10
5
6
4

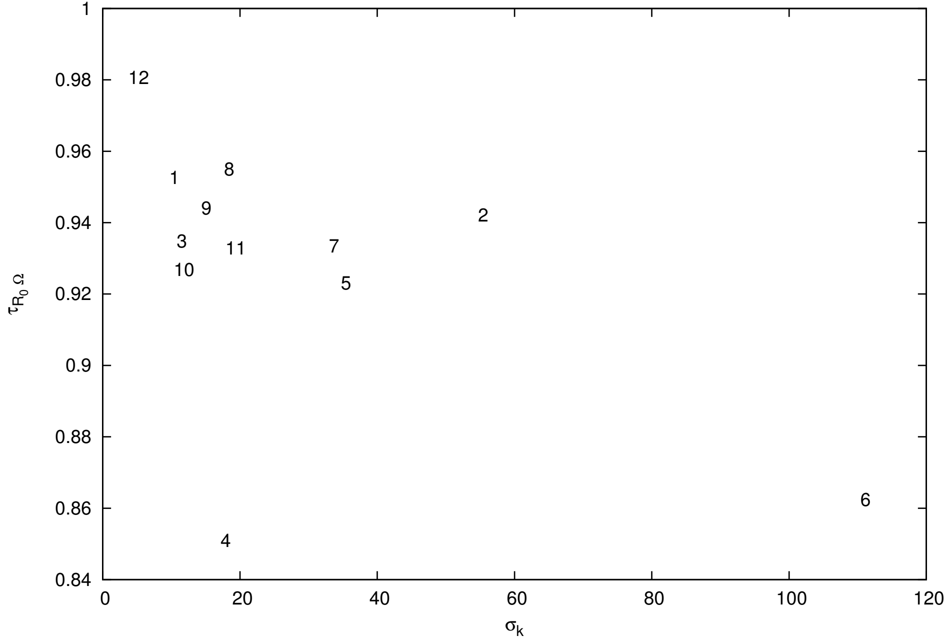

1
0.98
0.96
0.94
0.92
0.9
0.88
0.86
0.84
0
20
40
60
80
100
120
12
1
8
9
2
3
11
7
10
5
6
4
$\tau_{R_0}\,\Omega$
σ_k

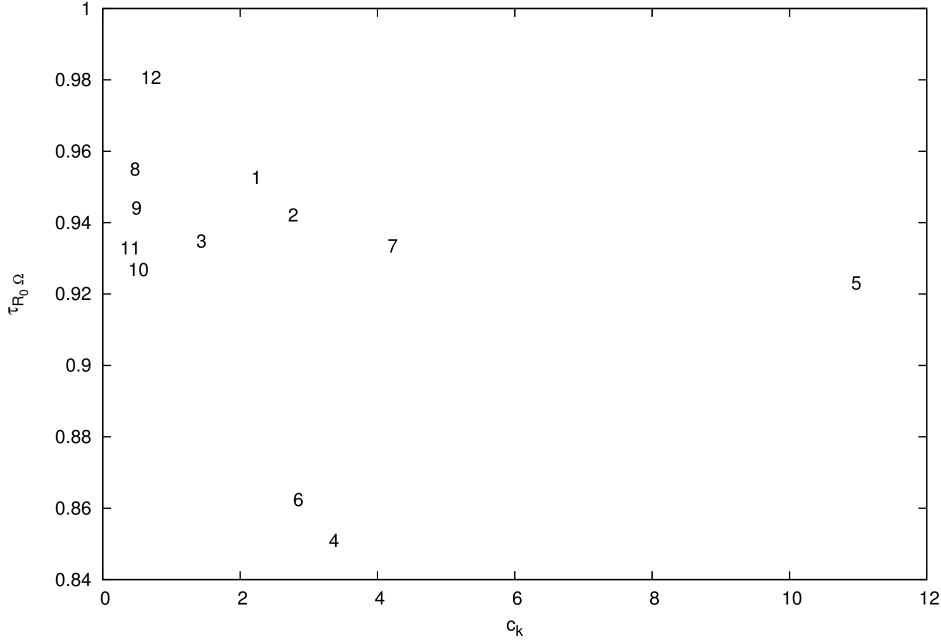

1
0.98
0.96
0.94
0.92
0.9
0.88
0.86
0.84
0
2
4
6
8
10
12
c_k
$\tau_{R_0}\,\Omega$
12
8
1
9
2
11
3
7
10
5
6
4

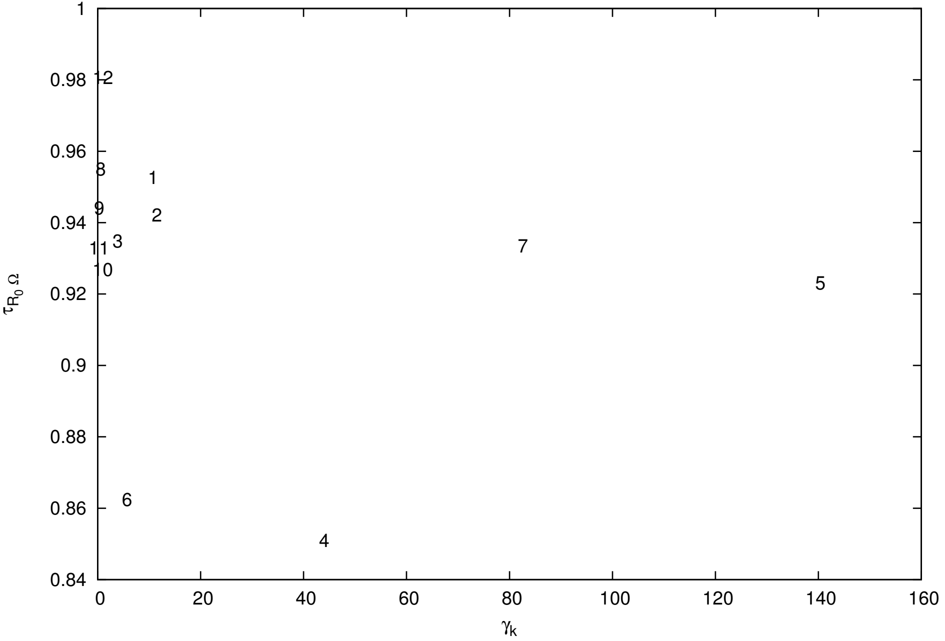

1
0.98
0.96
0.94
0.92
0.9
0.88
0.86
0.84
0
20
40
60
80
100
120
140
160
$\tau_{R_0\,\Omega}$
γ_k
12
8
1
9
2
3
11
10
7
5
6
4

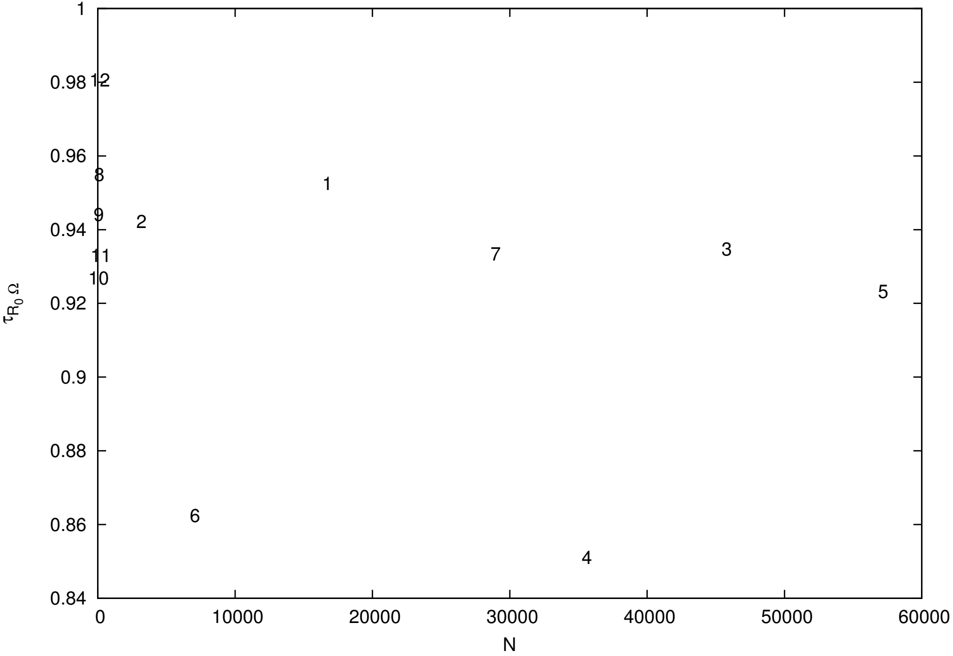

1
0.98
0.96
0.94
0.92
0.9
0.88
0.86
0.84
0
10000
20000
30000
40000
50000
60000
N
$\tau_{R_0}\,\Omega$
12
8
9
11
10
2
1
7
3
5
6
4

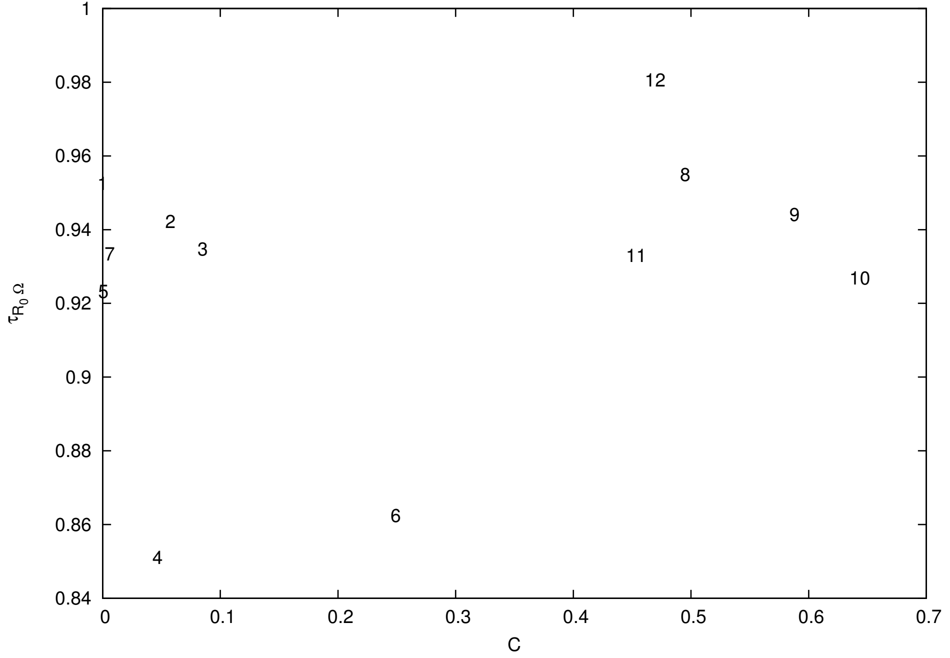

1
0.98
0.96
0.94
0.92
0.9
0.88
0.86
0.84
$\tau_{R_0}\,\Omega$
0
0.1
0.2
0.3
0.4
0.5
0.6
0.7
C
12
8
9
11
10
1
2
3
7
5
6
4

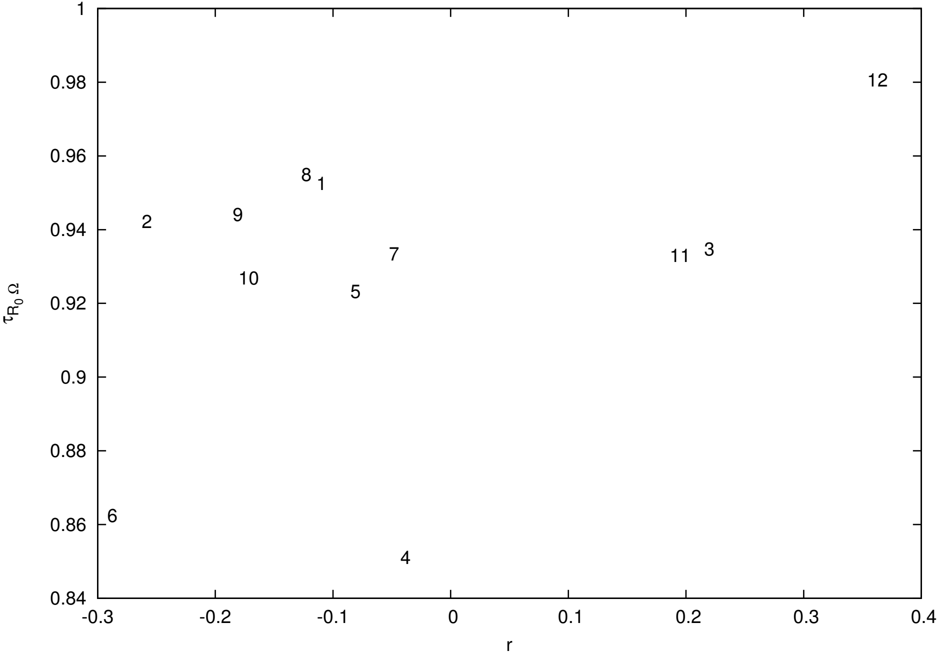

$\tau_{R_0}\Omega$
r
1
0.98
0.96
0.94
0.92
0.9
0.88
0.86
0.84
-0.3
-0.2
-0.1
0
0.1
0.2
0.3
0.4
1
2
3
4
5
6
7
8
9
10
11
12

ταΩ

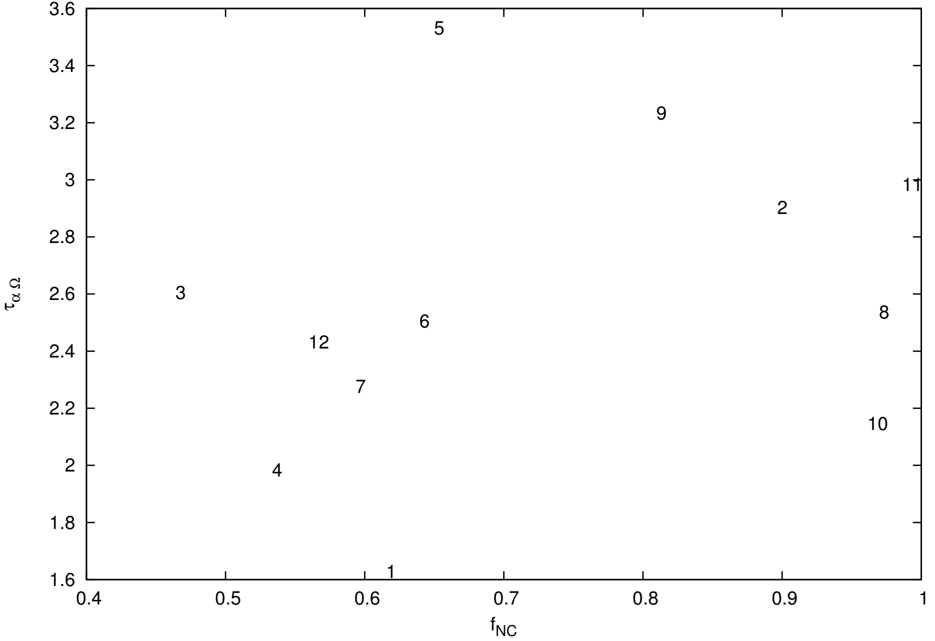

3.6
3.4
3.2
3
2.8
2.6
2.4
2.2
2
1.8
1.6
0.4
0.5
0.6
0.7
0.8
0.9
1
$\tau_{\alpha\Omega}$
f_{NC}
5
9
11
2
3
8
6
12
7
10
4
1

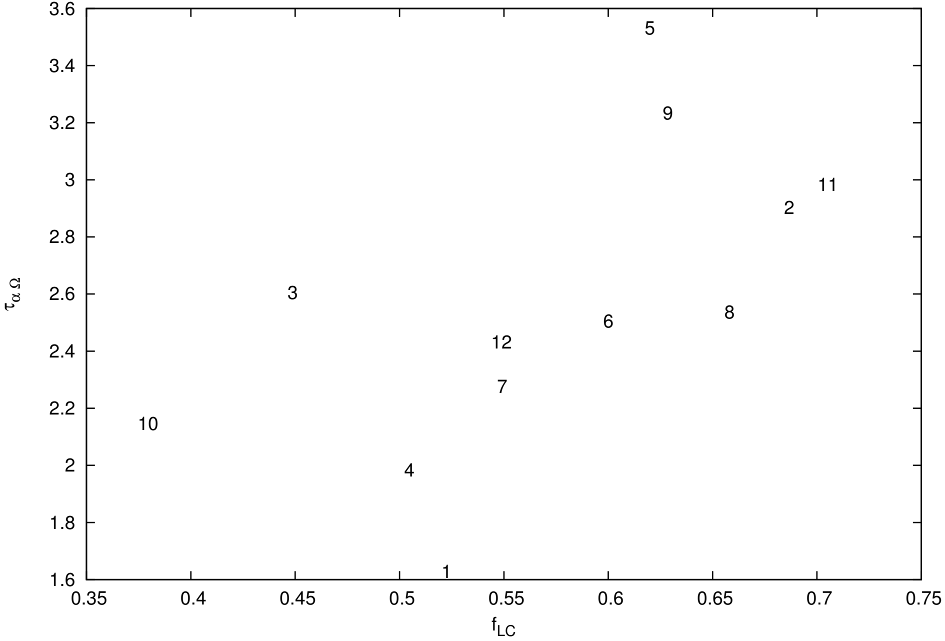

3.6
3.4
3.2
3
2.8
2.6
2.4
2.2
2
1.8
1.6
$\tau_{\alpha\Omega}$
0.35
0.4
0.45
0.5
0.55
0.6
0.65
0.7
0.75
f_{LC}
5
9
11
2
3
8
6
12
7
10
4
1

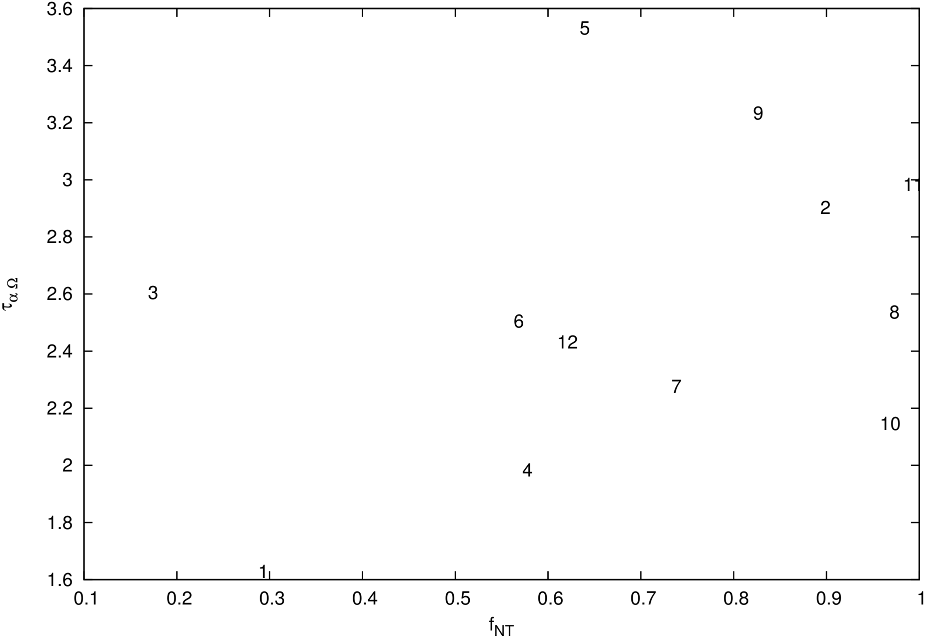

3.6
3.4
3.2
3
2.8
2.6
2.4
2.2
2
1.8
1.6
0.1
0.2
0.3
0.4
0.5
0.6
0.7
0.8
0.9
1
$\tau_{\alpha\Omega}$
f_{NT}
5
9
11
2
3
8
6
12
7
10
4
1

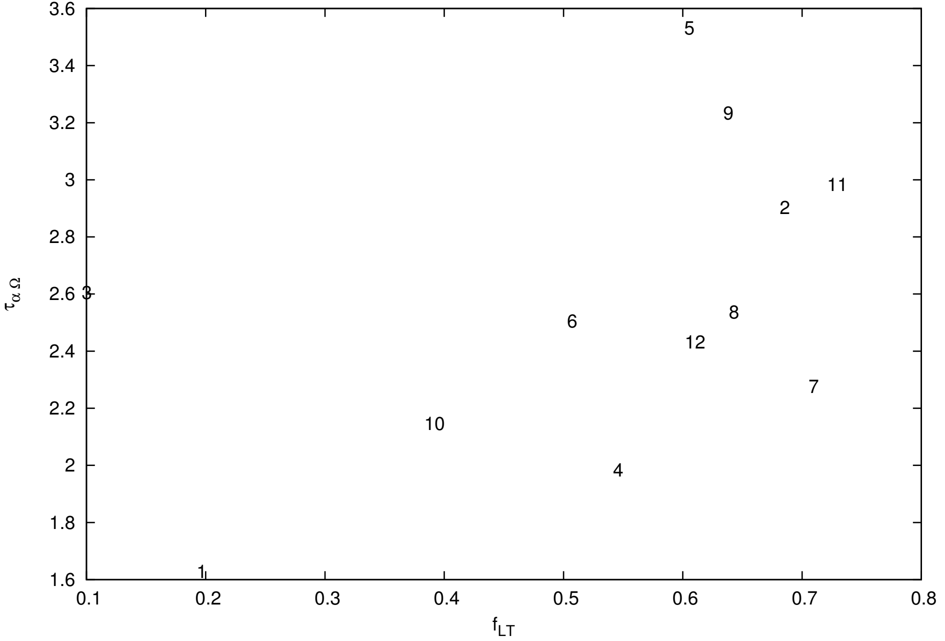

3.6
3.4
3.2
3
2.8
2.6
2.4
2.2
2
1.8
1.6
0.1
0.2
0.3
0.4
0.5
0.6
0.7
0.8
f_{LT}
$\tau_{\alpha\Omega}$
1
2
3
4
5
6
7
8
9
10
11
12

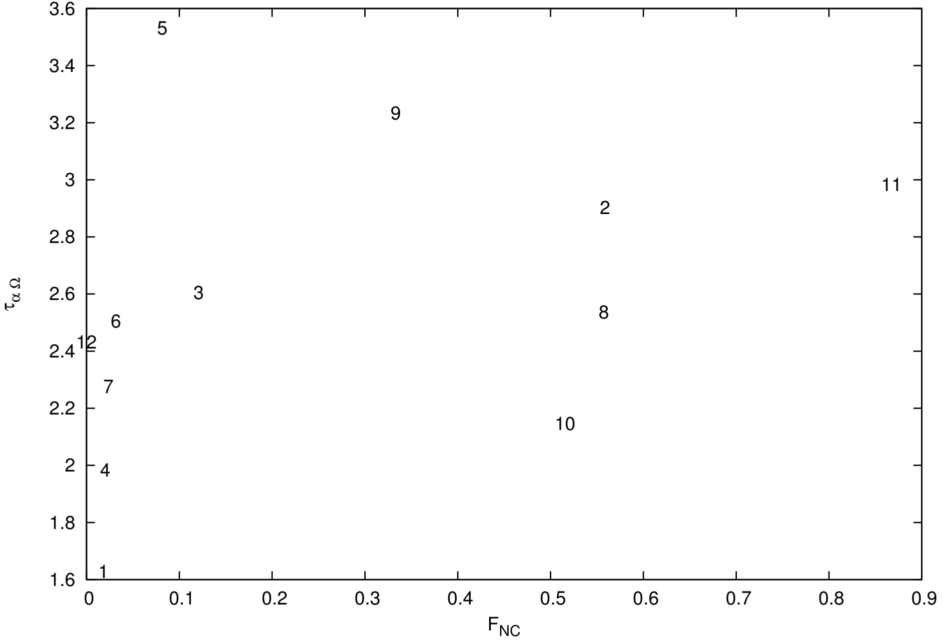

$\tau_{\alpha\,\Omega}$
F_{NC}
3.6
3.4
3.2
3
2.8
2.6
2.4
2.2
2
1.8
1.6
0
0.1
0.2
0.3
0.4
0.5
0.6
0.7
0.8
0.9
5
9
11
2
3
8
6
12
7
10
4
1

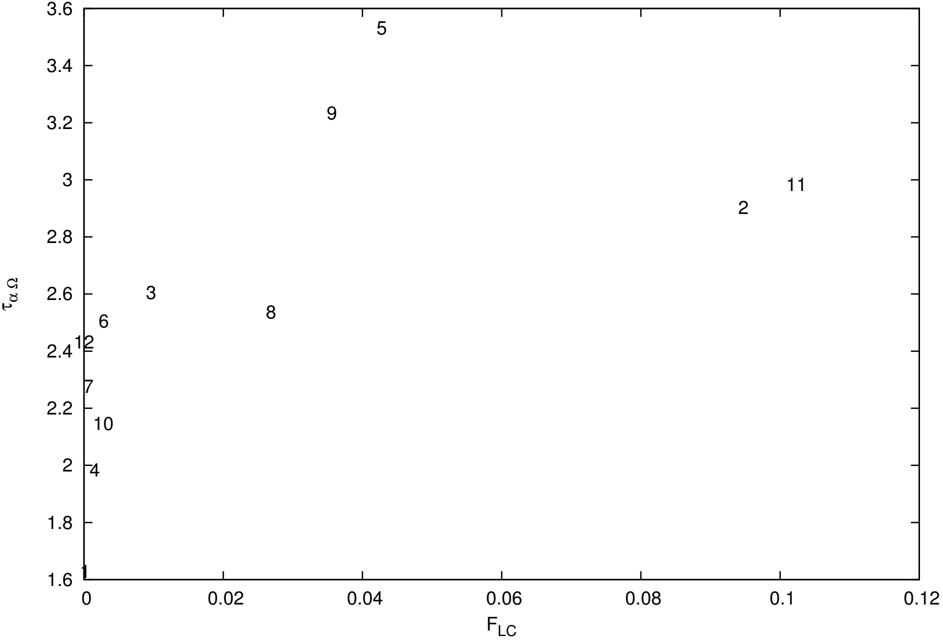

3.6
3.4
3.2
3
2.8
2.6
2.4
2.2
2
1.8
1.6
0
0.02
0.04
0.06
0.08
0.1
0.12
F_{LC}
$\tau_{\alpha\Omega}$
5
9
11
2
3
8
6
12
7
10
4
1

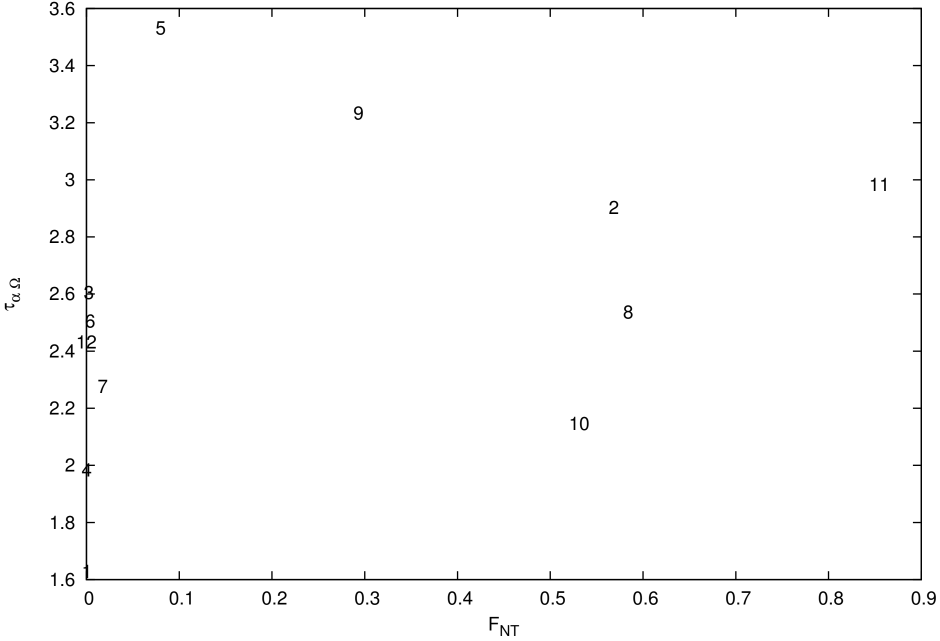

3.6
3.4
3.2
3
2.8
2.6
2.4
2.2
2
1.8
1.6
0
0.1
0.2
0.3
0.4
0.5
0.6
0.7
0.8
0.9
F_{NT}
$\tau_{\alpha\Omega}$
5
9
11
2
3
8
6
12
7
10
4
1

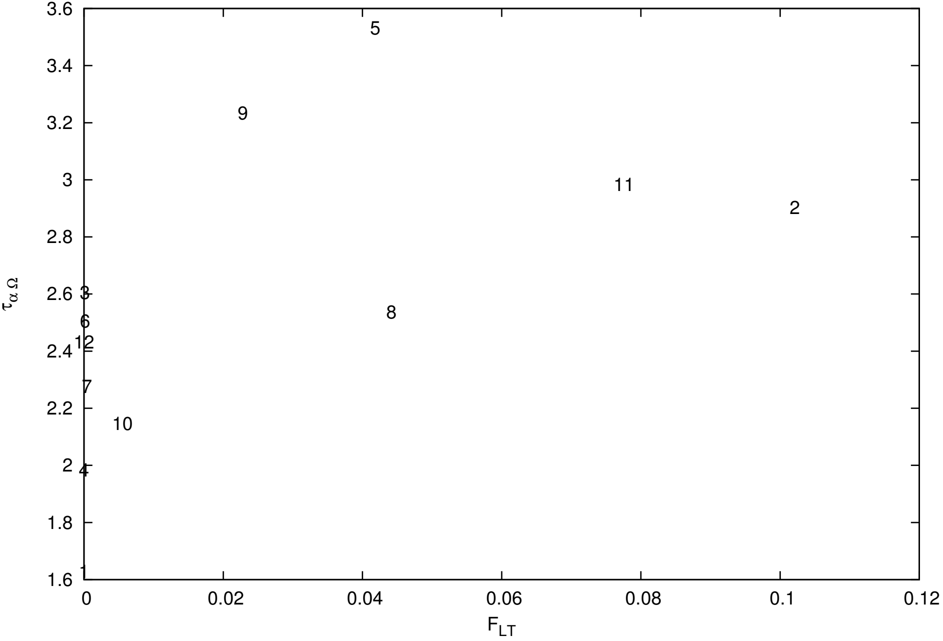

3.6
3.4
3.2
3
2.8
2.6
2.4
2.2
2
1.8
1.6
0
0.02
0.04
0.06
0.08
0.1
0.12
$\tau_{\alpha\,\Omega}$
F_{LT}
5
9
11
2
3
6
12
8
7
10
4
1

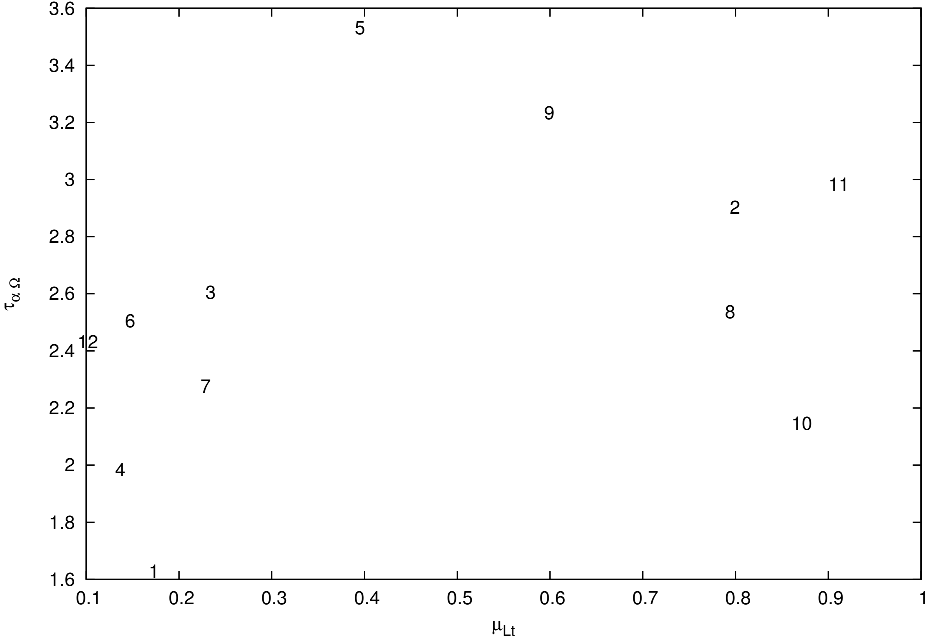

3.6
3.4
3.2
3
2.8
2.6
2.4
2.2
2
1.8
1.6
τ α Ω
0.1
0.2
0.3
0.4
0.5
0.6
0.7
0.8
0.9
1
μ Lt
1
2
3
4
5
6
7
8
9
10
11
12

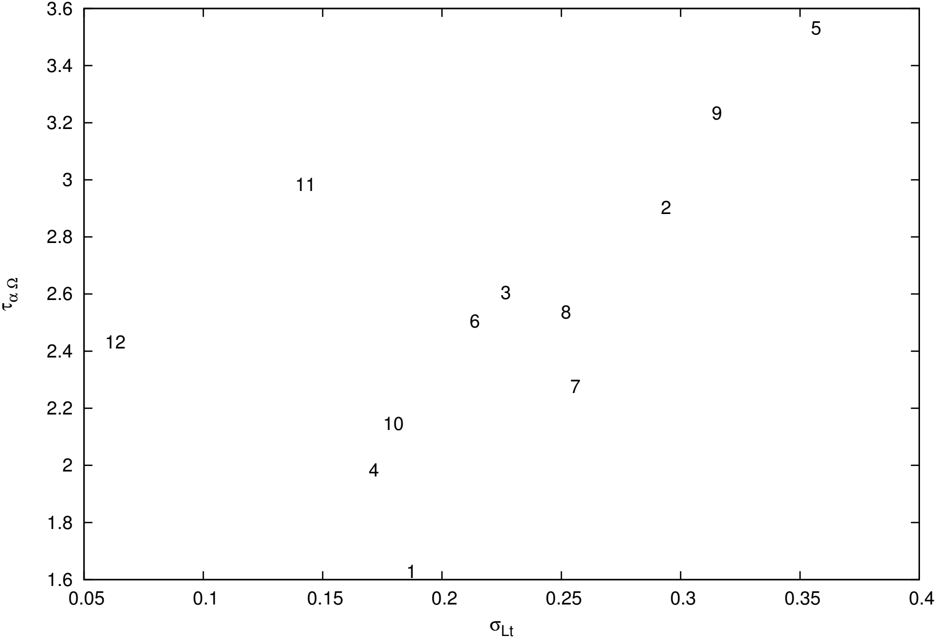

3.6
3.4
3.2
3
2.8
2.6
2.4
2.2
2
1.8
1.6
0.05
0.1
0.15
0.2
0.25
0.3
0.35
0.4
$\tau_{\alpha\Omega}$
σ_{Lt}
5
9
11
2
3
8
6
12
7
10
4
1

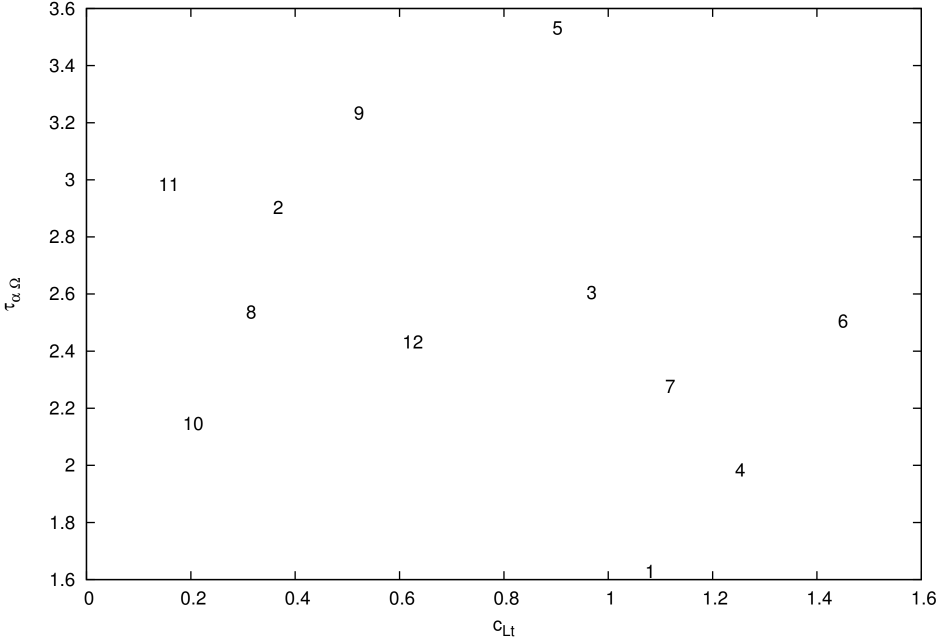

3.6
3.4
3.2
3
2.8
2.6
2.4
2.2
2
1.8
1.6
0
0.2
0.4
0.6
0.8
1
1.2
1.4
1.6
$\tau_{\alpha\Omega}$
c_{Lt}
5
9
11
2
3
8
6
12
7
10
4
1

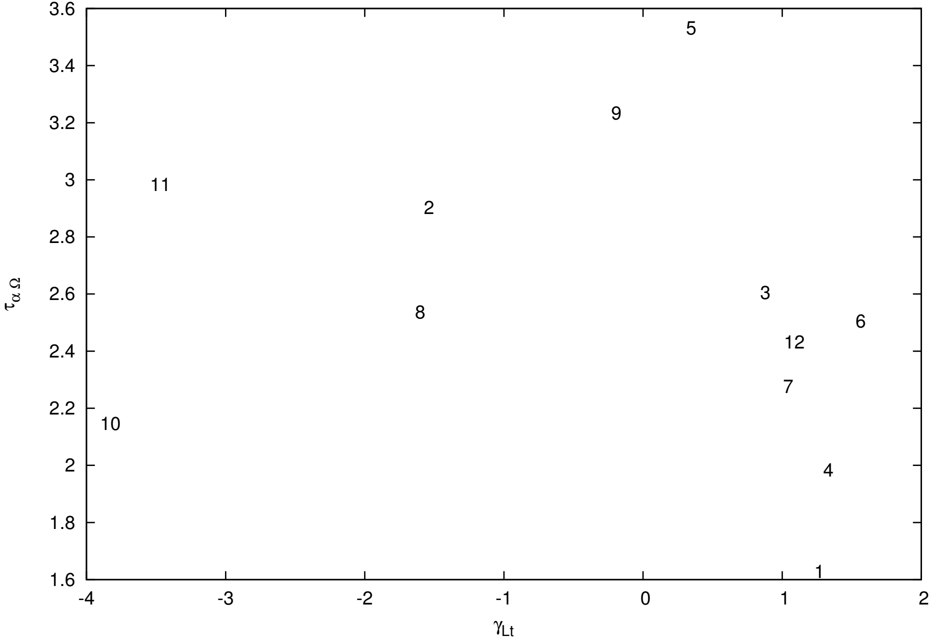

3.6
3.4
3.2
3
2.8
2.6
2.4
2.2
2
1.8
1.6
-4
-3
-2
-1
0
1
2
$\tau_{\alpha\,\Omega}$
γ_{Lt}
1
2
3
4
5
6
7
8
9
10
11
12

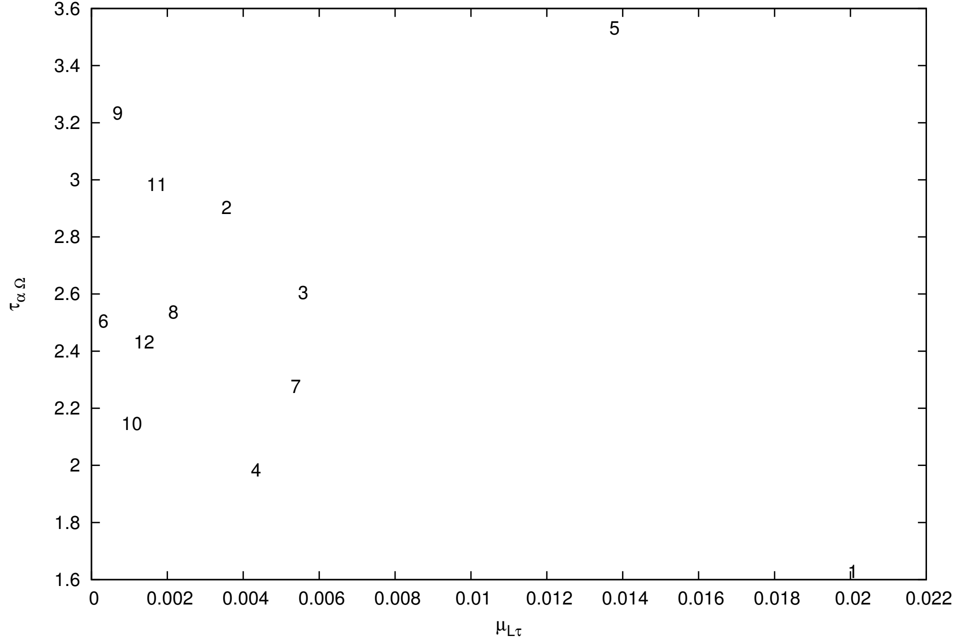

3.6
3.4
3.2
3
2.8
2.6
2.4
2.2
2
1.8
1.6
$\tau_{\alpha\Omega}$
0
0.002
0.004
0.006
0.008
0.01
0.012
0.014
0.016
0.018
0.02
0.022
$\mu_{L\tau}$
1
2
3
4
5
6
7
8
9
10
11
12

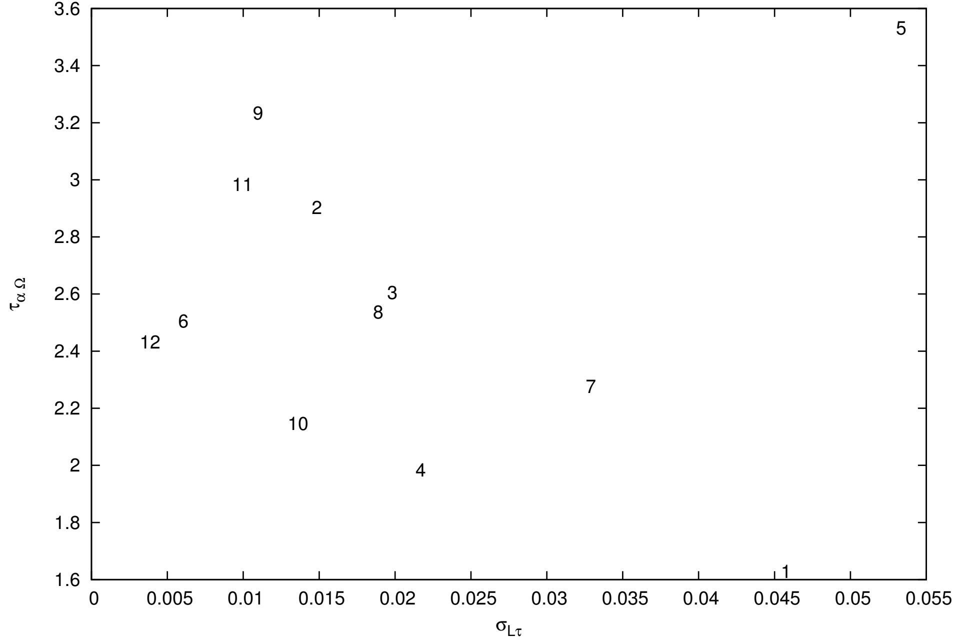

3.6
3.4
3.2
3
2.8
2.6
2.4
2.2
2
1.8
1.6
0
0.005
0.01
0.015
0.02
0.025
0.03
0.035
0.04
0.045
0.05
0.055
$\tau_{\alpha\Omega}$
$\sigma_{L\tau}$
1
2
3
4
5
6
7
8
9
10
11
12

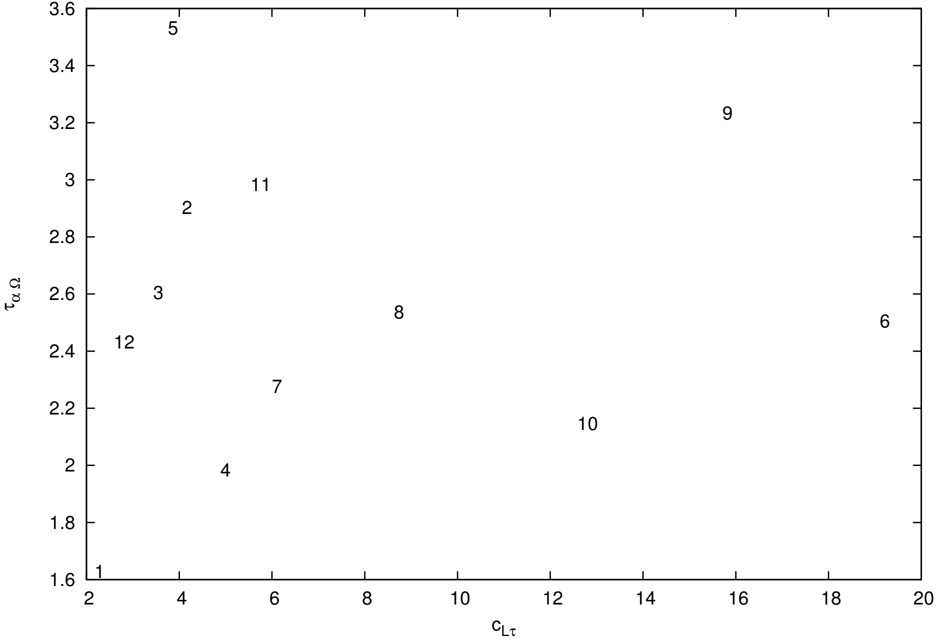

3.6
3.4
3.2
3
2.8
2.6
2.4
2.2
2
1.8
1.6
$\tau_{\alpha\Omega}$
5
9
11
2
3
8
6
12
7
10
4
1
2
4
6
8
10
12
14
16
18
20
$c_{L\tau}$

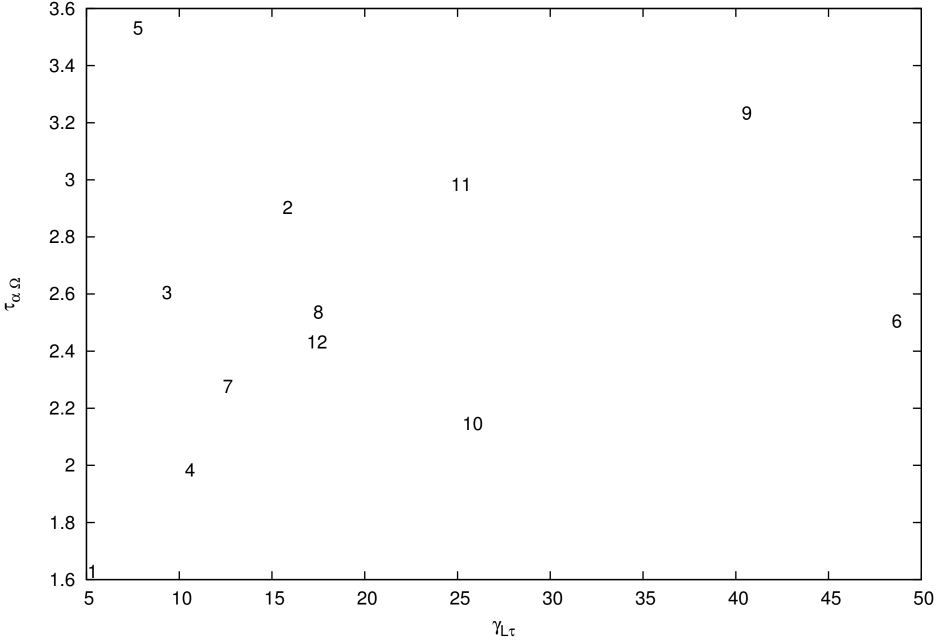

3.6
3.4
3.2
3
2.8
2.6
2.4
2.2
2
1.8
1.6
5
10
15
20
25
30
35
40
45
50
$\tau_{\alpha\,\Omega}$
$\gamma_{L\tau}$
5
9
11
2
3
8
6
12
7
10
4
1

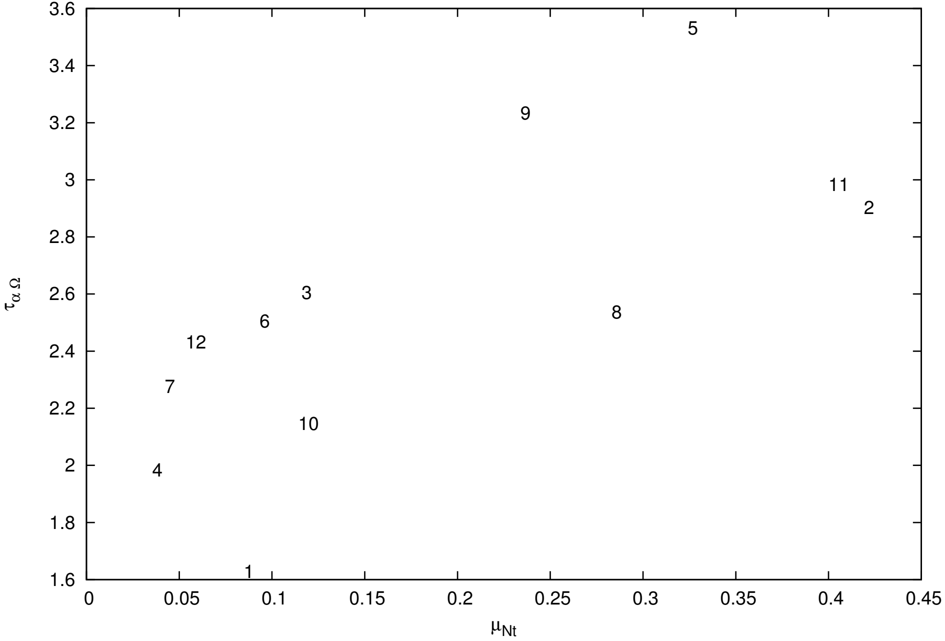

3.6
3.4
3.2
3
2.8
2.6
2.4
2.2
2
1.8
1.6
0
0.05
0.1
0.15
0.2
0.25
0.3
0.35
0.4
0.45
$\tau_{\alpha\,\Omega}$
μ_{Nt}
5
9
11
2
3
8
6
12
7
10
4
1

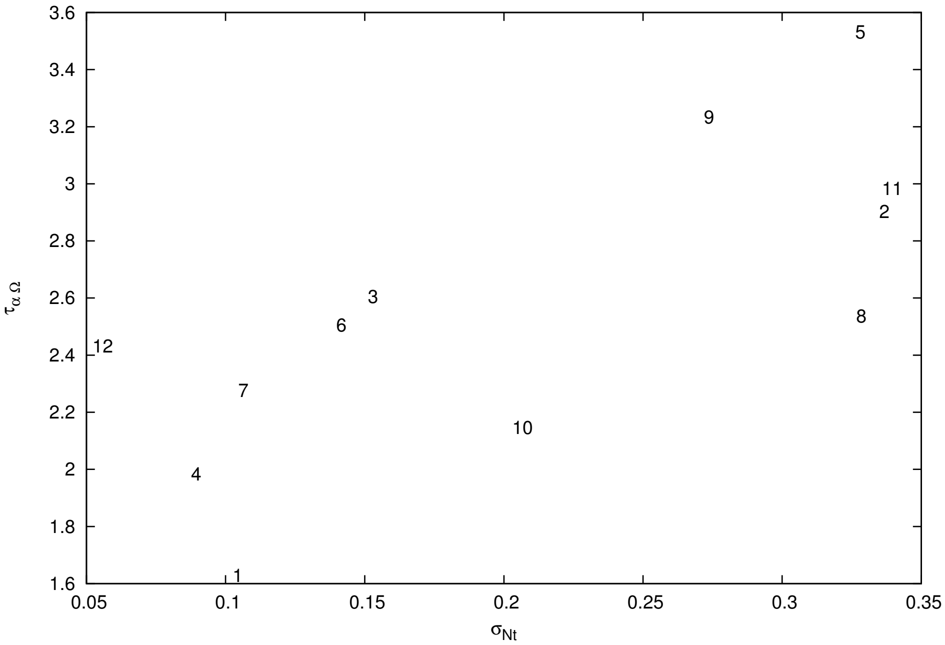

3.6
3.4
3.2
3
2.8
2.6
2.4
2.2
2
1.8
1.6
0.05
0.1
0.15
0.2
0.25
0.3
0.35
$\tau_{\alpha\Omega}$
σ_{Nt}
1
2
3
4
5
6
7
8
9
10
11
12

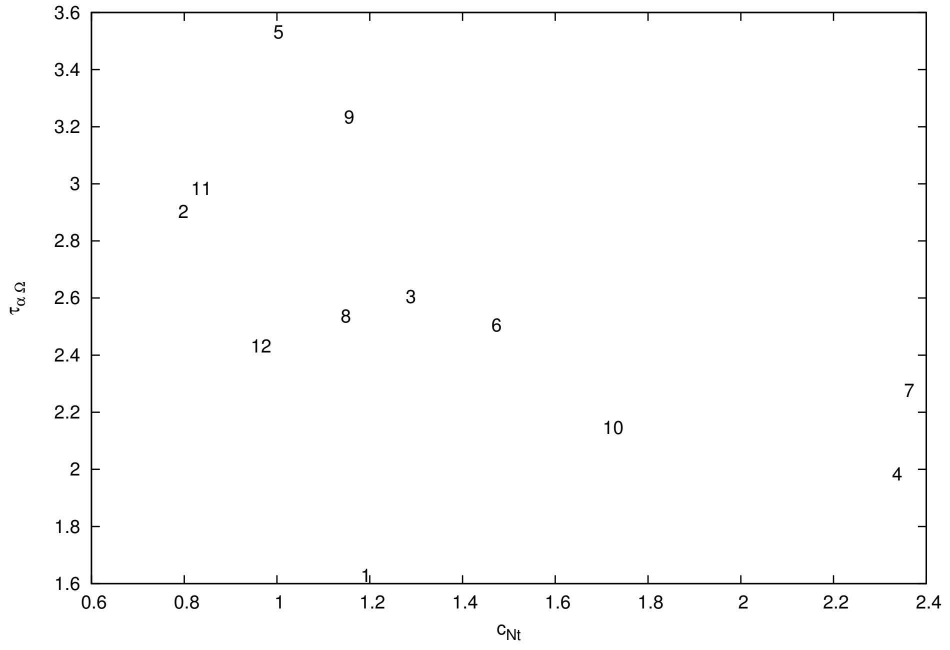

3.6
3.4
3.2
3
2.8
2.6
2.4
2.2
2
1.8
1.6
0.6
0.8
1
1.2
1.4
1.6
1.8
2
2.2
2.4
$\tau_{\alpha\,\Omega}$
c_{Nt}
5
9
11
2
3
8
6
12
7
10
4
1

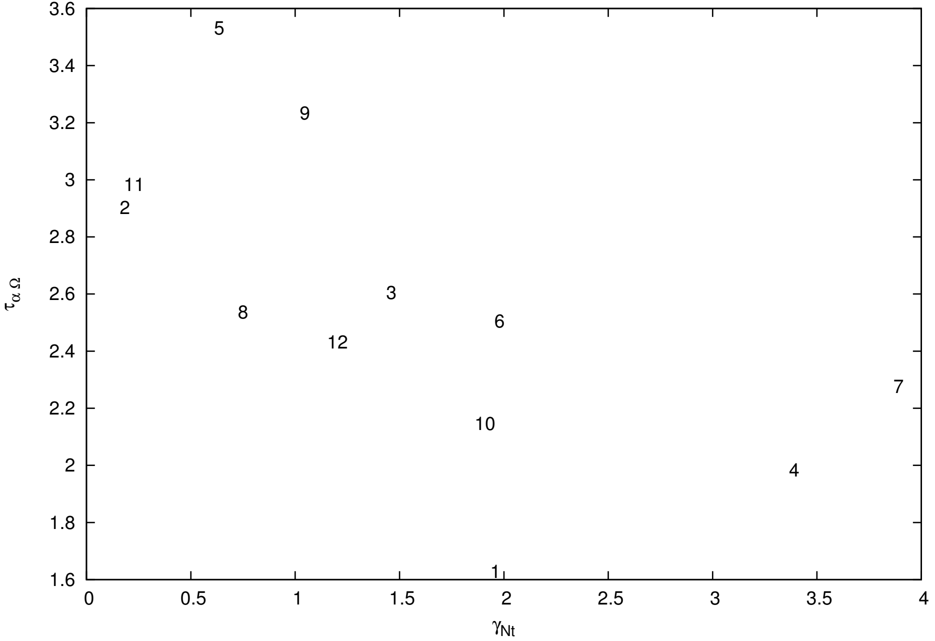

3.6
3.4
3.2
3
2.8
2.6
2.4
2.2
2
1.8
1.6
0
0.5
1
1.5
2
2.5
3
3.5
4
τα Ω
γNt
5
9
11
2
3
8
6
12
7
10
4
1

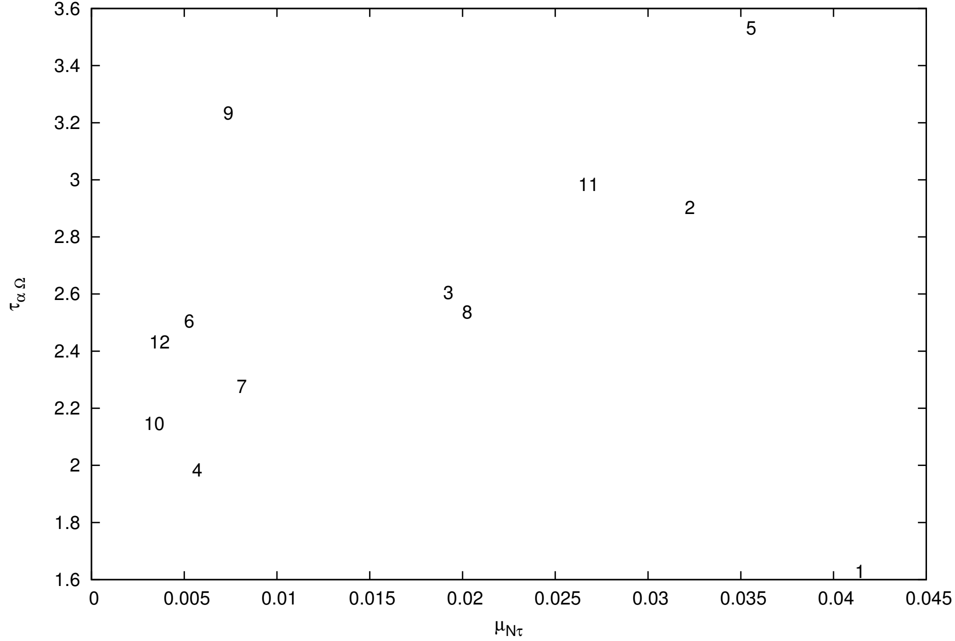

3.6
3.4
3.2
3
2.8
2.6
2.4
2.2
2
1.8
1.6
τ_{α Ω}
0
0.005
0.01
0.015
0.02
0.025
0.03
0.035
0.04
0.045
μ_{Nτ}
1
2
3
4
5
6
7
8
9
10
11
12

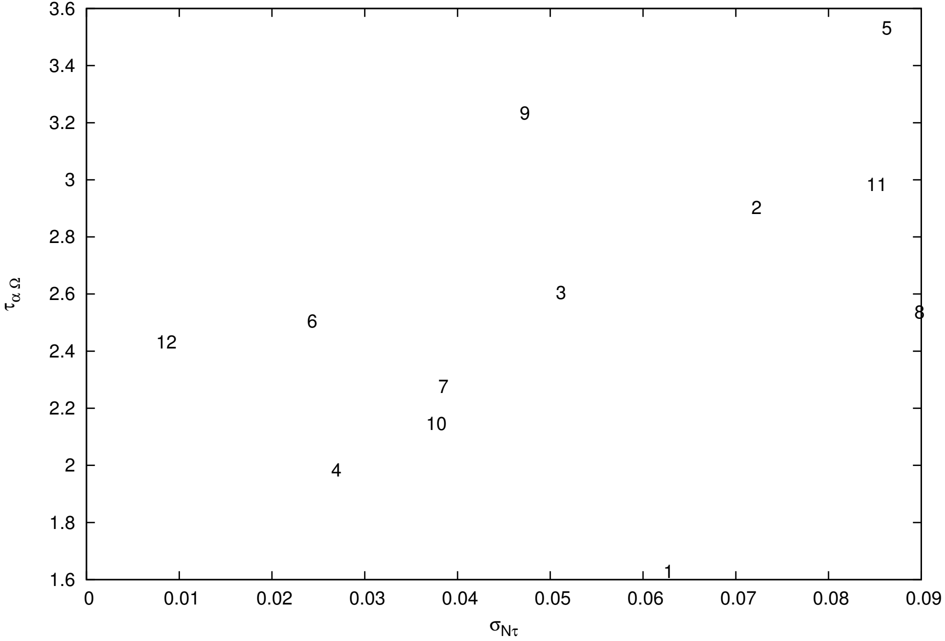

$\tau_{\alpha\,\Omega}$
$\sigma_{N\tau}$
3.6
3.4
3.2
3
2.8
2.6
2.4
2.2
2
1.8
1.6
0
0.01
0.02
0.03
0.04
0.05
0.06
0.07
0.08
0.09
1
2
3
4
5
6
7
8
9
10
11
12

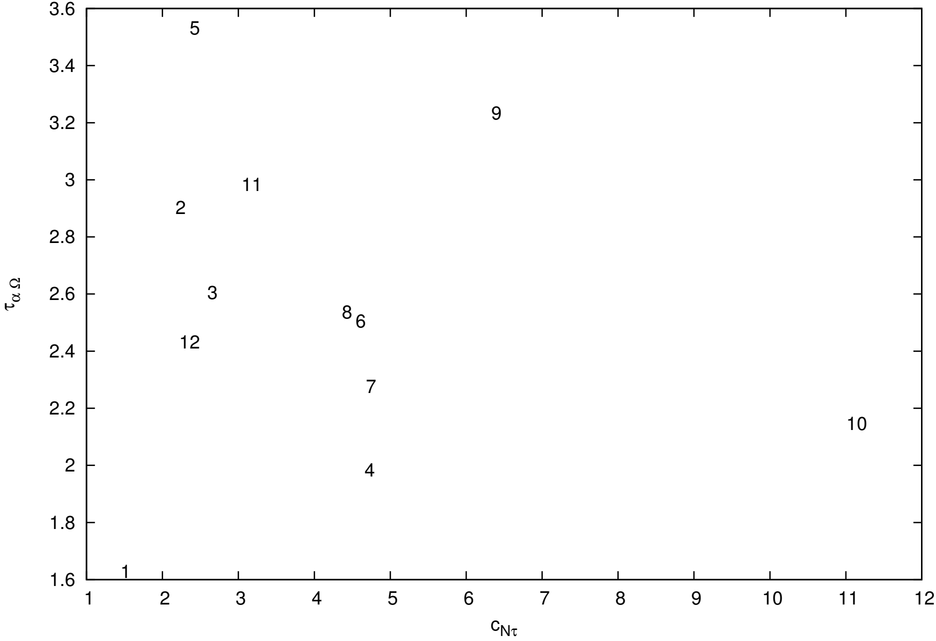

3.6
3.4
3.2
3
2.8
2.6
2.4
2.2
2
1.8
1.6
1
2
3
4
5
6
7
8
9
10
11
12
τ_{α Ω}
c_{Nτ}
5
9
11
2
3
8
6
12
7
10
4
1

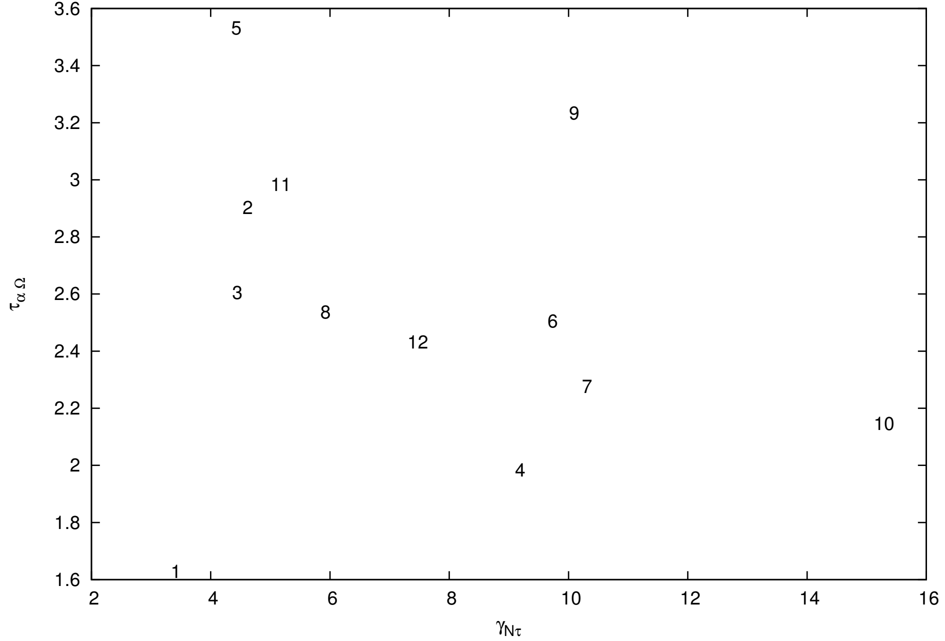

3.6
3.4
3.2
3
2.8
2.6
2.4
2.2
2
1.8
1.6
$\tau_{\alpha\,\Omega}$
2
4
6
8
10
12
14
16
$\gamma_{N\tau}$
5
9
11
2
3
8
6
12
7
10
4
1

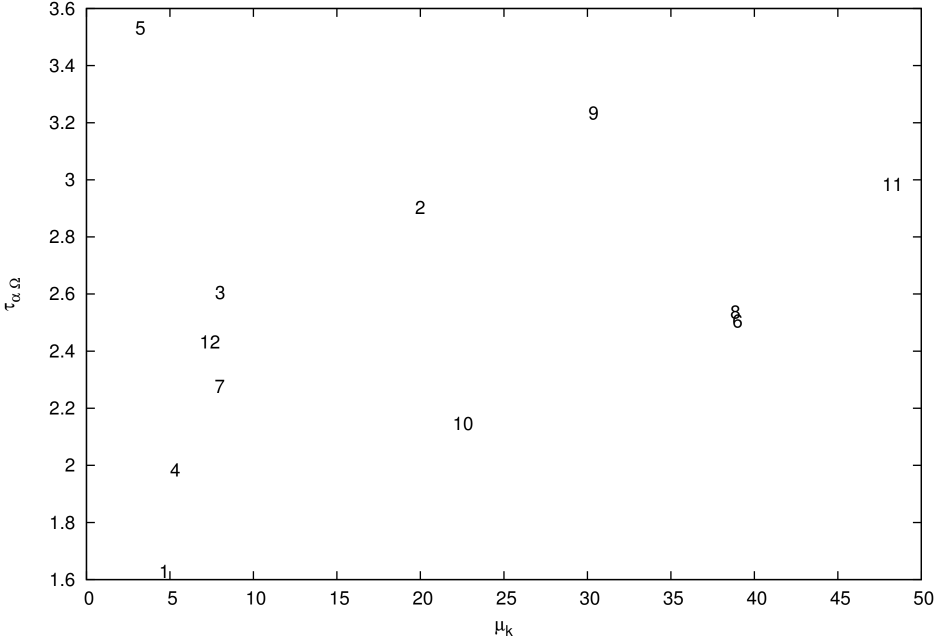
5
9
11
2
3
8
6
12
7
10
4
1
3.6
3.4
3.2
3
2.8
2.6
2.4
2.2
2
1.8
1.6
0
5
10
15
20
25
30
35
40
45
50
$\tau_{\alpha\Omega}$
μ_k

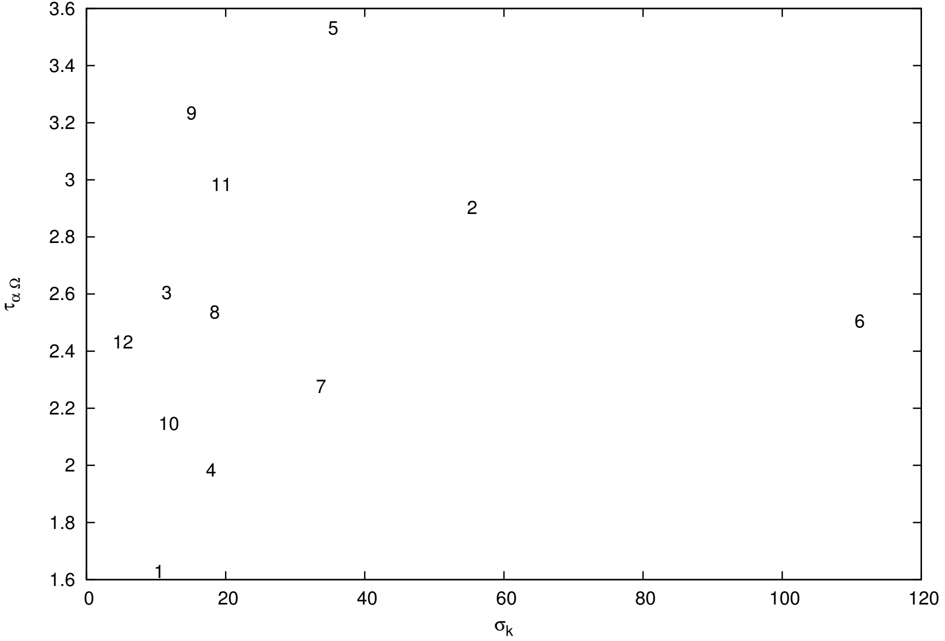

3.6
3.4
3.2
3
2.8
2.6
2.4
2.2
2
1.8
1.6
0
20
40
60
80
100
120
$\tau_{\alpha\,\Omega}$
σ_k
1
2
3
4
5
6
7
8
9
10
11
12

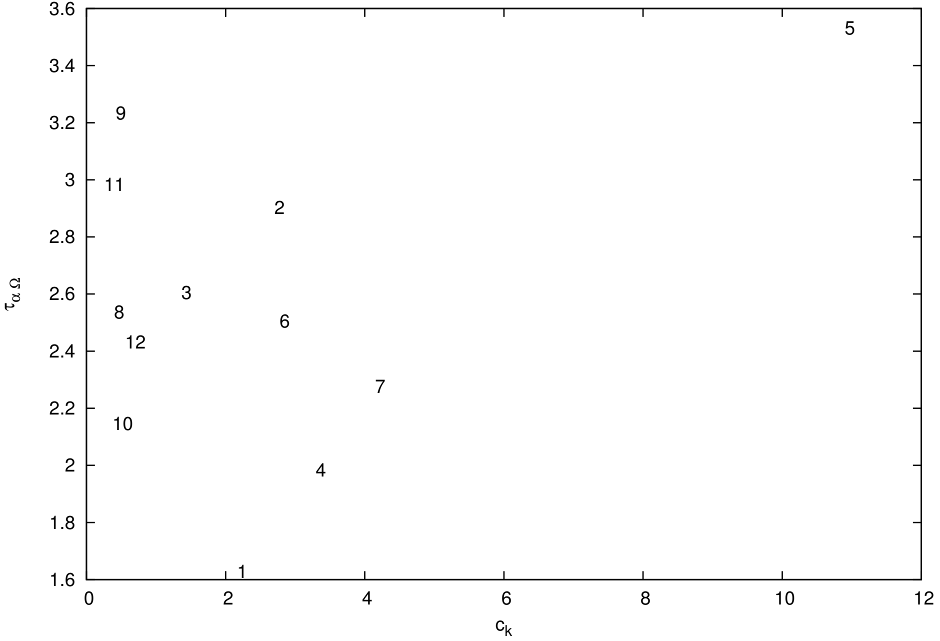

3.6
3.4
3.2
3
2.8
2.6
2.4
2.2
2
1.8
1.6
0
2
4
6
8
10
12
$\tau_{\alpha\Omega}$
c_k
5
9
11
2
3
8
6
12
7
10
4
1

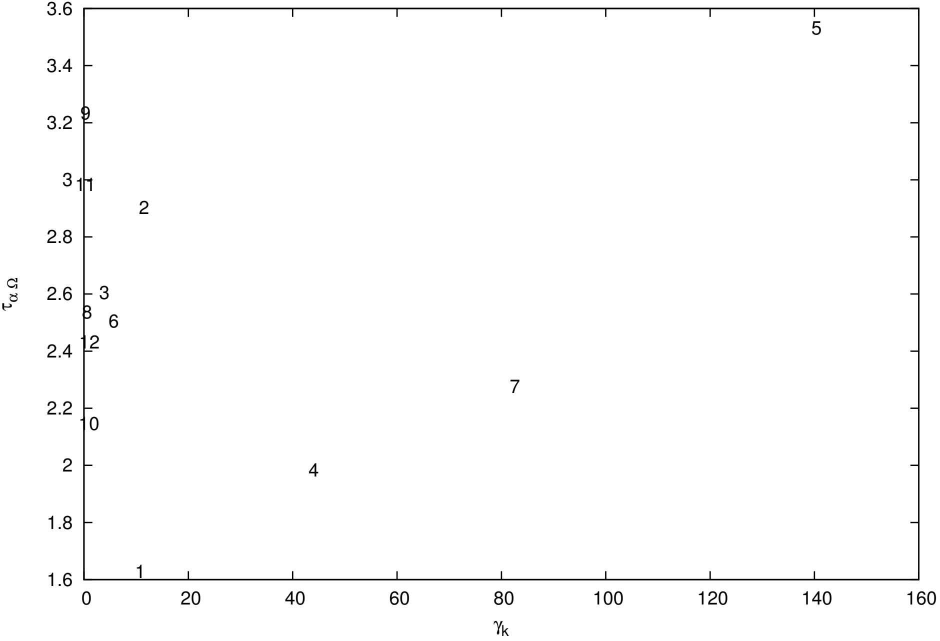

3.6
3.4
3.2
3
2.8
2.6
2.4
2.2
2
1.8
1.6
0
20
40
60
80
100
120
140
160
$\tau_{\alpha\,\Omega}$
γ_k
1
2
3
4
5
6
7
8
9
10
11
12

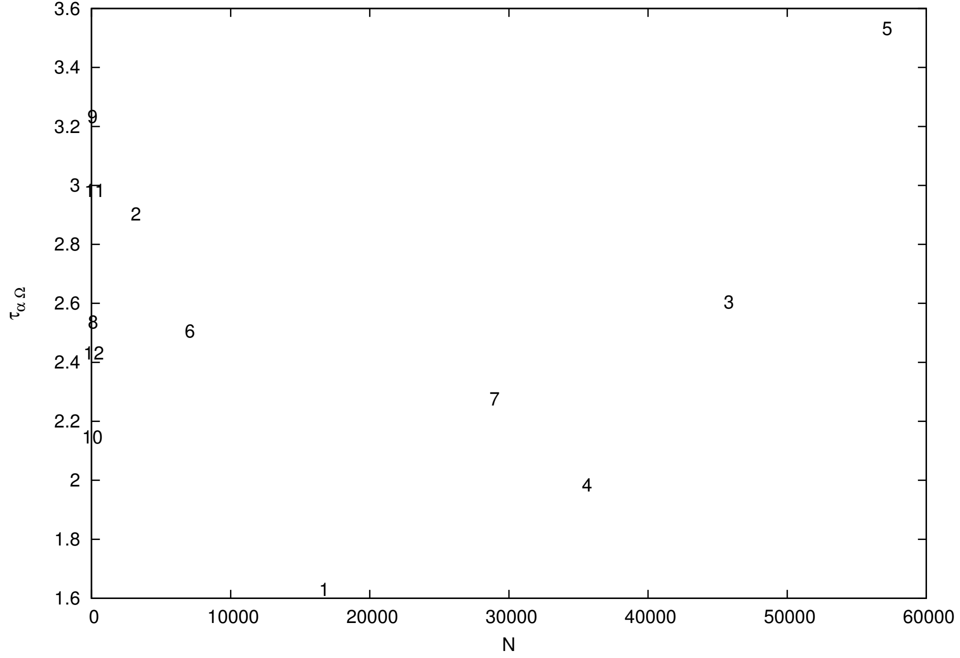

$\tau_{\alpha\Omega}$
N
1.6
1.8
2
2.2
2.4
2.6
2.8
3
3.2
3.4
3.6
0
10000
20000
30000
40000
50000
60000
1
2
3
4
5
6
7
8
9
10
11
12

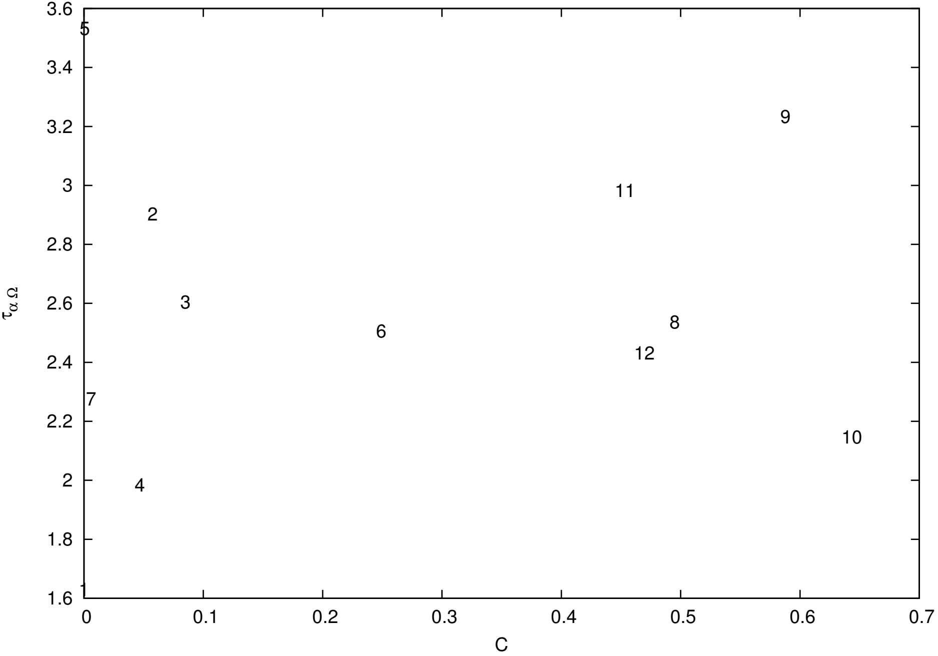

$\tau_{\alpha\,\Omega}$
C
5
9
11
2
3
8
6
12
7
10
4
1
3.6
3.4
3.2
3
2.8
2.6
2.4
2.2
2
1.8
1.6
0
0.1
0.2
0.3
0.4
0.5
0.6
0.7

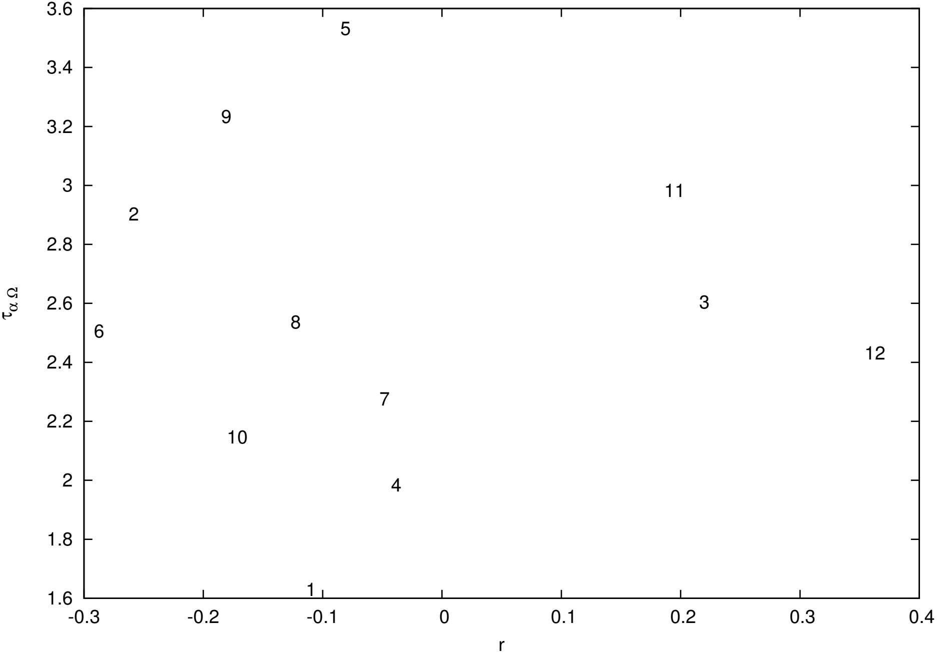

3.6
3.4
3.2
3
2.8
2.6
2.4
2.2
2
1.8
1.6
-0.3
-0.2
-0.1
0
0.1
0.2
0.3
0.4
r
$\tau_{\alpha\,\Omega}$
1
2
3
4
5
6
7
8
9
10
11
12

ρ_{R_0}

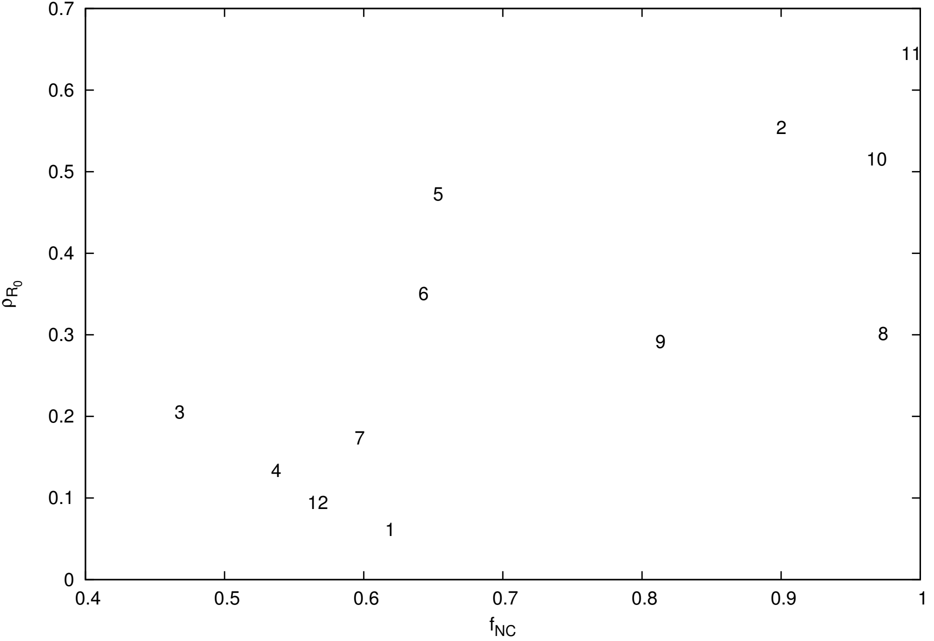

0.7
0.6
0.5
0.4
0.3
0.2
0.1
0
0.4
0.5
0.6
0.7
0.8
0.9
1
ρ_{R_0}
f_{NC}
1
2
3
4
5
6
7
8
9
10
11
12

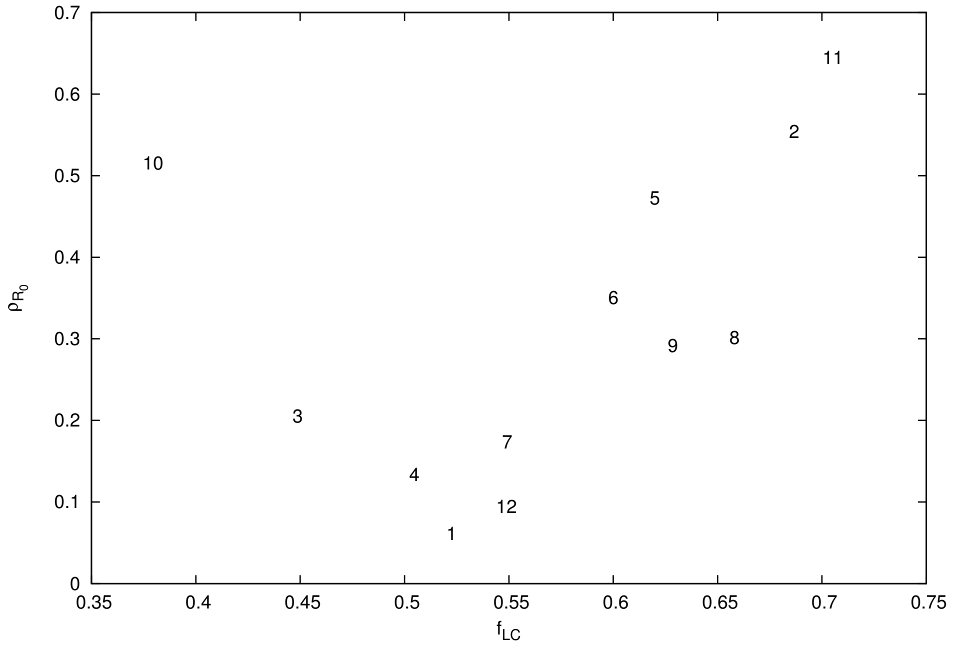

0.7
0.6
0.5
0.4
0.3
0.2
0.1
0
0.35
0.4
0.45
0.5
0.55
0.6
0.65
0.7
0.75
ρ_{R_0}
f_{LC}
1
2
3
4
5
6
7
8
9
10
11
12

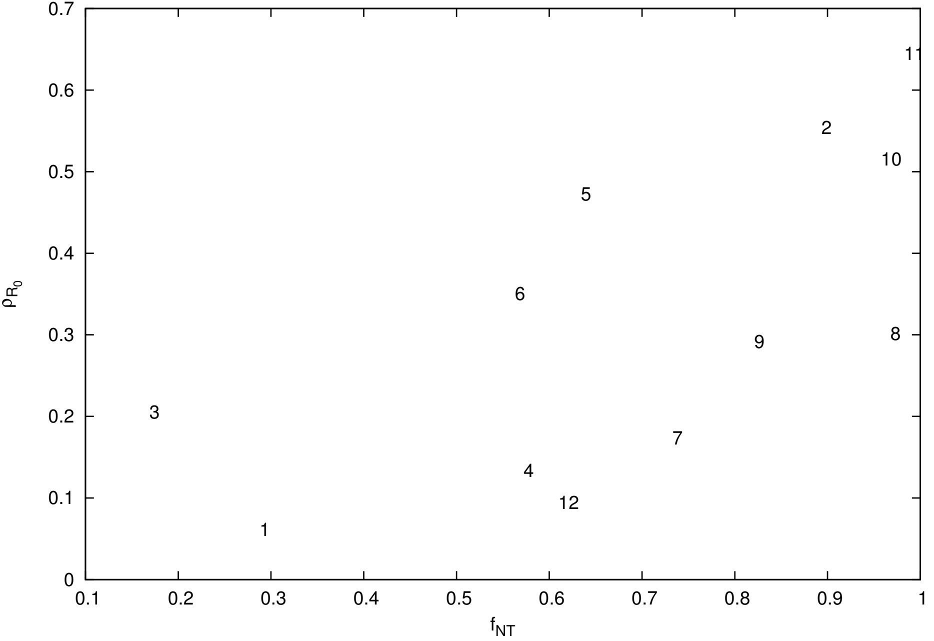

0.7
0.6
0.5
0.4
0.3
0.2
0.1
0
0.1
0.2
0.3
0.4
0.5
0.6
0.7
0.8
0.9
1
ρ_{R_0}
f_{NT}
11
2
10
5
6
9
8
3
7
4
12
1

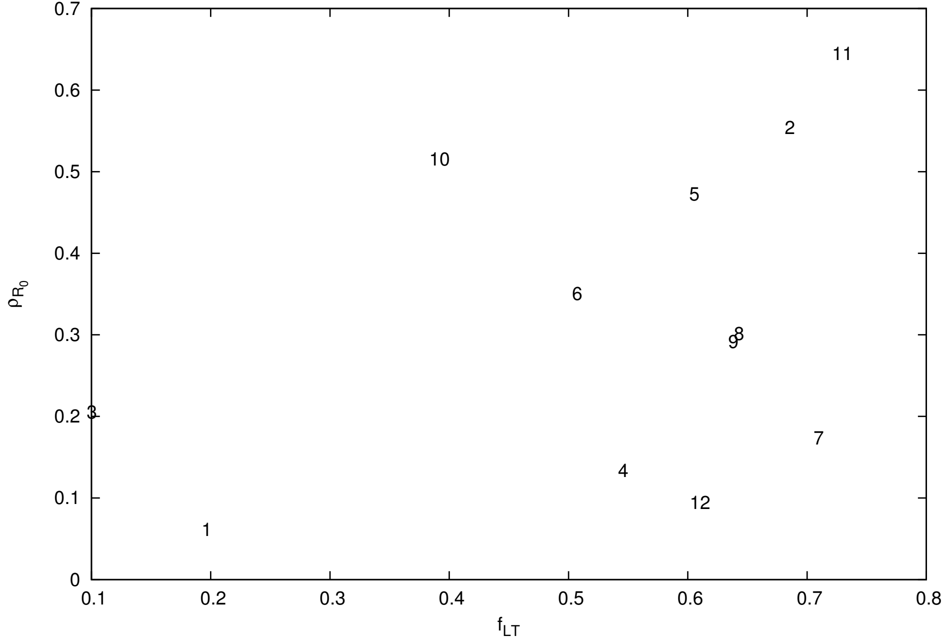

0.7
0.6
0.5
0.4
0.3
0.2
0.1
0
0.1
0.2
0.3
0.4
0.5
0.6
0.7
0.8
ρ_{R_0}
f_{LT}
11
2
10
5
6
8
9
3
7
4
12
1

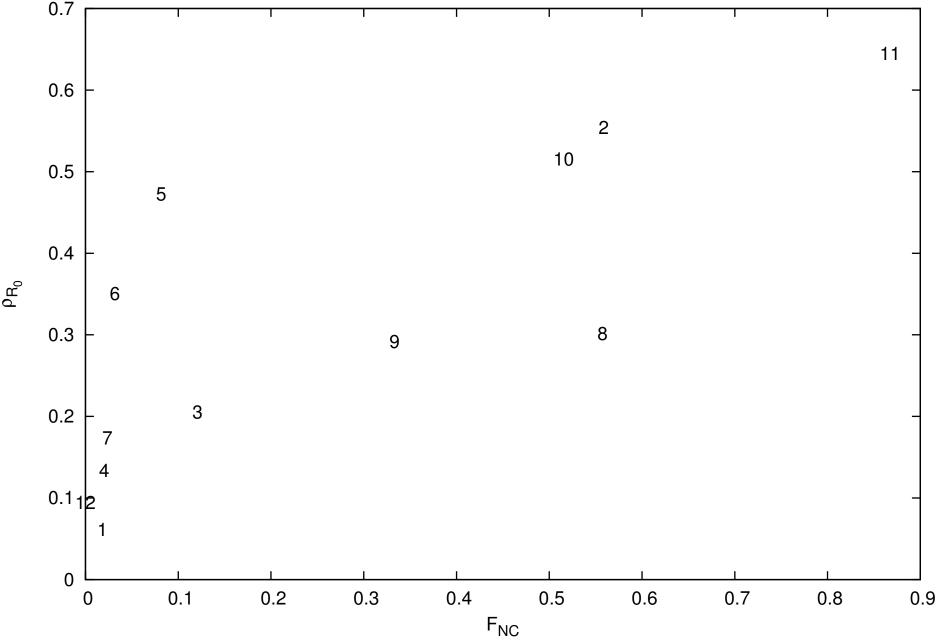

0.7
0.6
0.5
0.4
0.3
0.2
0.1
0
0
0.1
0.2
0.3
0.4
0.5
0.6
0.7
0.8
0.9
ρ_{R_0}
F_{NC}
11
2
10
5
6
8
9
3
7
4
12
1

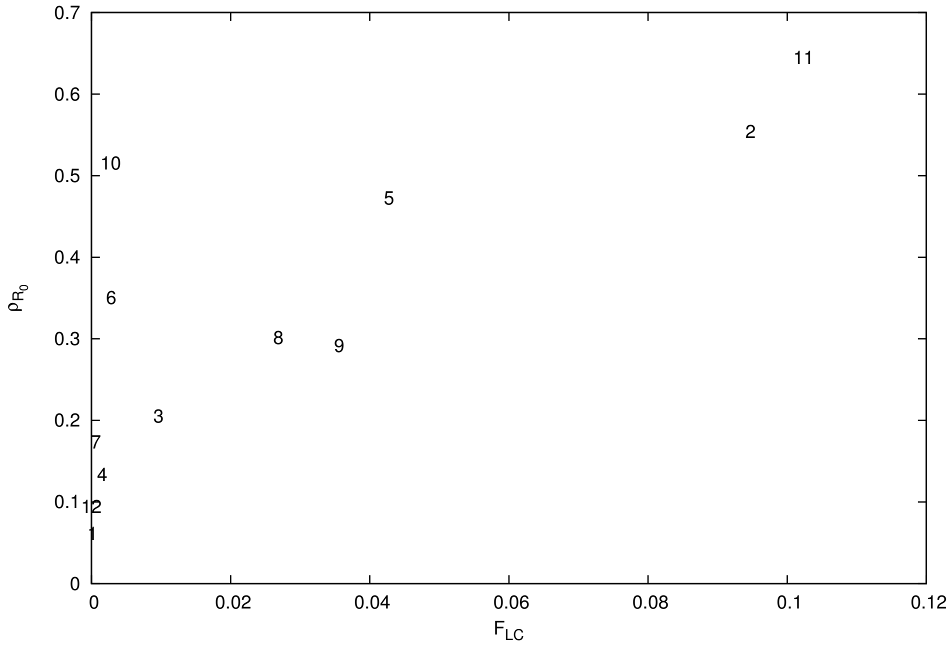

0.7
0.6
0.5
0.4
0.3
0.2
0.1
0
0
0.02
0.04
0.06
0.08
0.1
0.12
ρ_{R_0}
F_{LC}
11
2
10
5
6
8
9
3
7
4
12
1

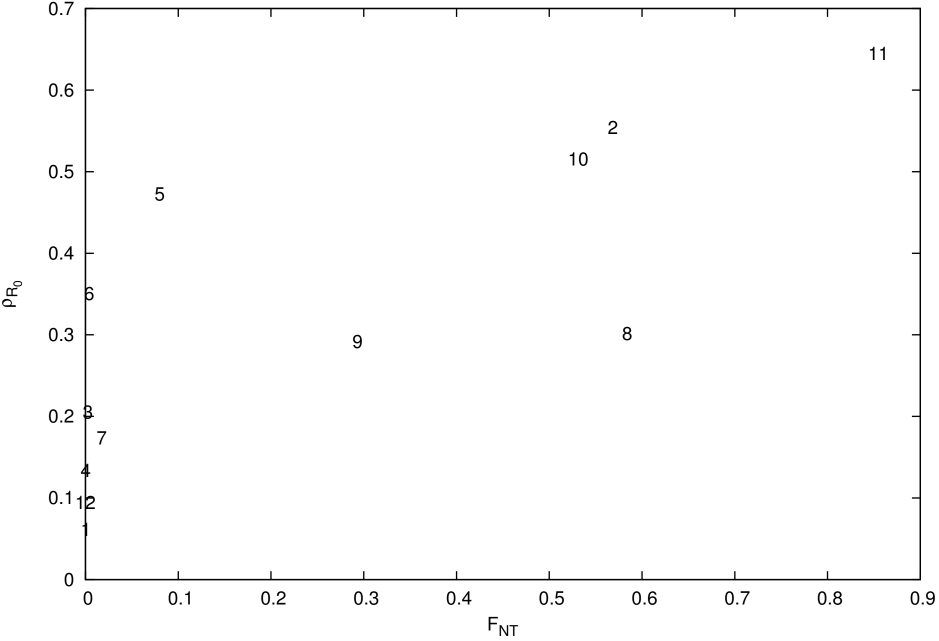

0.7
0.6
0.5
0.4
0.3
0.2
0.1
0
0
0.1
0.2
0.3
0.4
0.5
0.6
0.7
0.8
0.9
ρ_{R_0}
F_{NT}
11
2
10
5
6
9
8
3
7
4
12
1

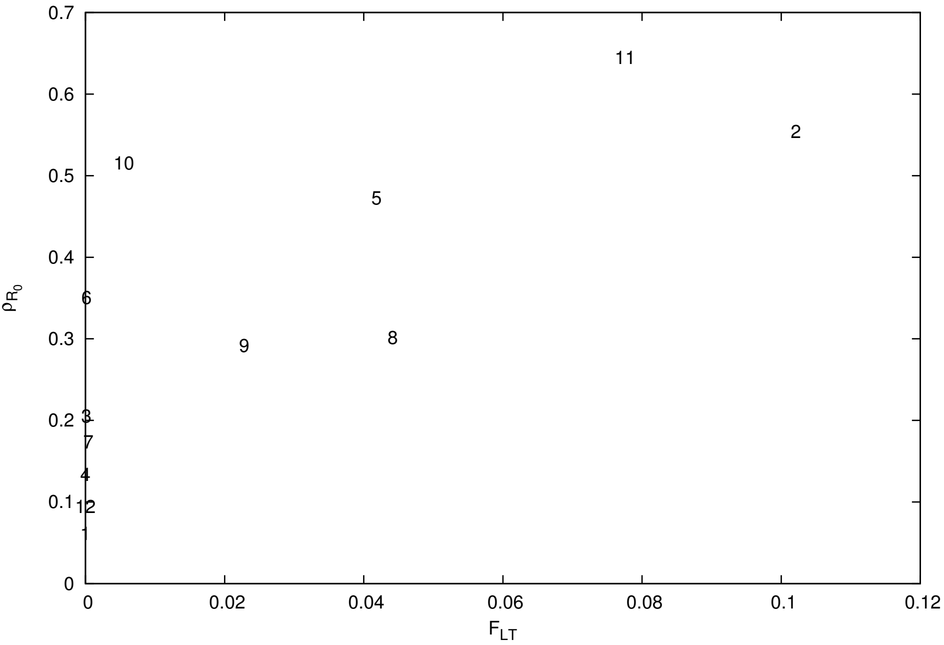

0.7
0.6
0.5
0.4
0.3
0.2
0.1
0
0
0.02
0.04
0.06
0.08
0.1
0.12
ρ_{R_0}
F_{LT}
11
2
10
5
6
9
8
3
7
4
12
1

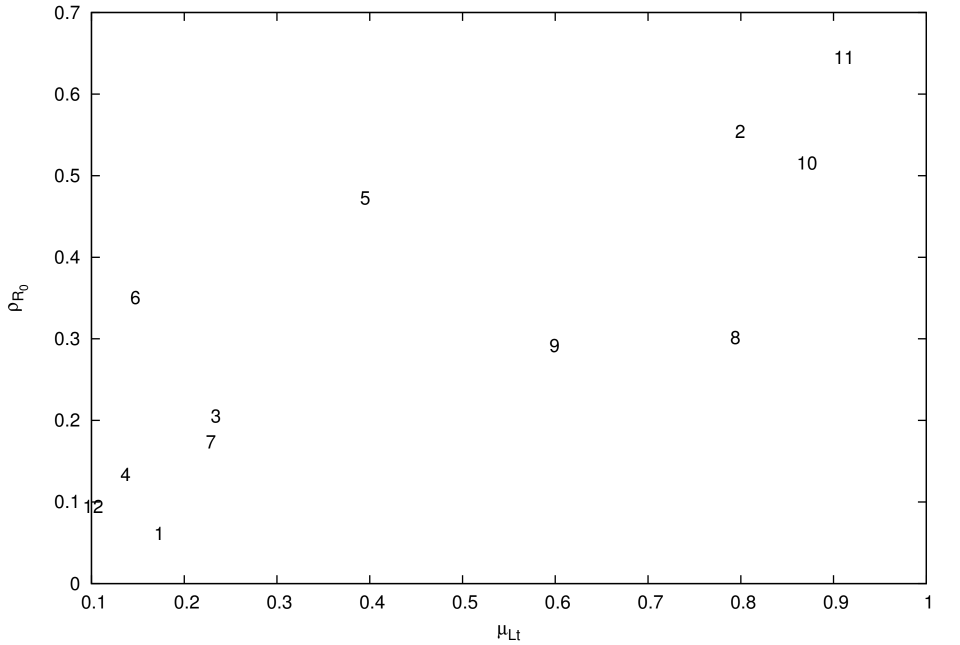

0.7
0.6
0.5
0.4
0.3
0.2
0.1
0
0.1
0.2
0.3
0.4
0.5
0.6
0.7
0.8
0.9
1
ρ_{R_0}
μ_{Lt}
11
2
10
5
6
9
8
3
7
4
12
1

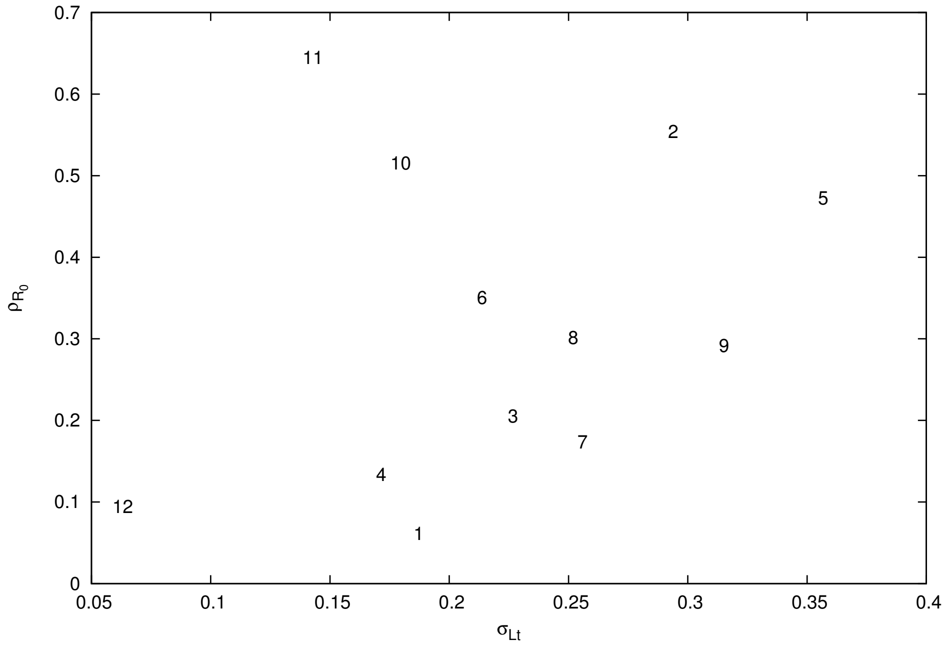

0.7
0.6
0.5
0.4
0.3
0.2
0.1
0
ρ_{R_0}
0.05
0.1
0.15
0.2
0.25
0.3
0.35
0.4
σ_{Lt}
11
2
10
5
6
8
9
3
7
4
12
1

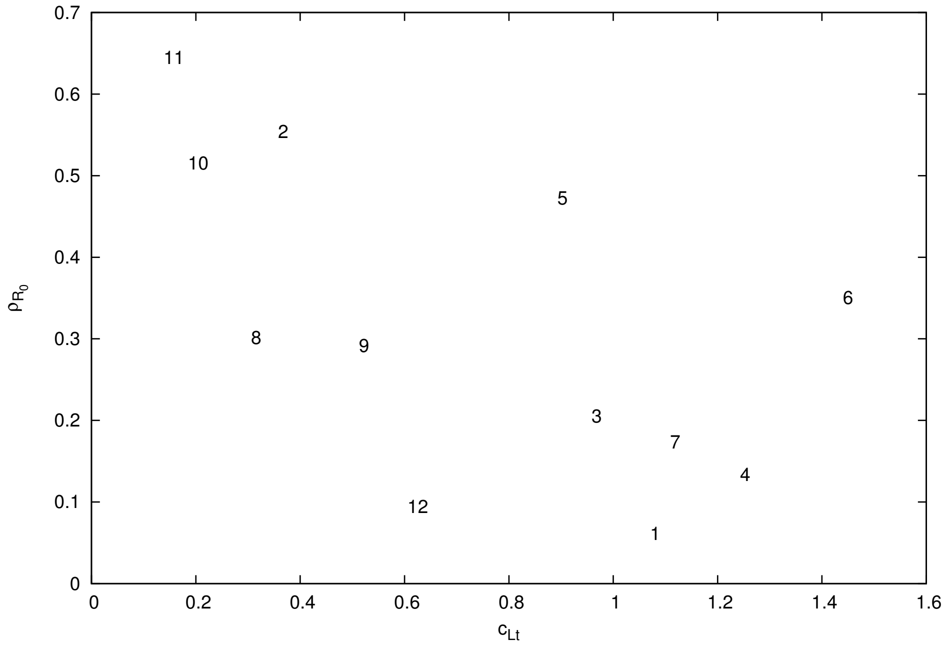

0.7
0.6
0.5
0.4
0.3
0.2
0.1
0
0
0.2
0.4
0.6
0.8
1
1.2
1.4
1.6
ρ_{R_0}
c_{Lt}
11
2
10
5
6
8
9
3
7
4
12
1

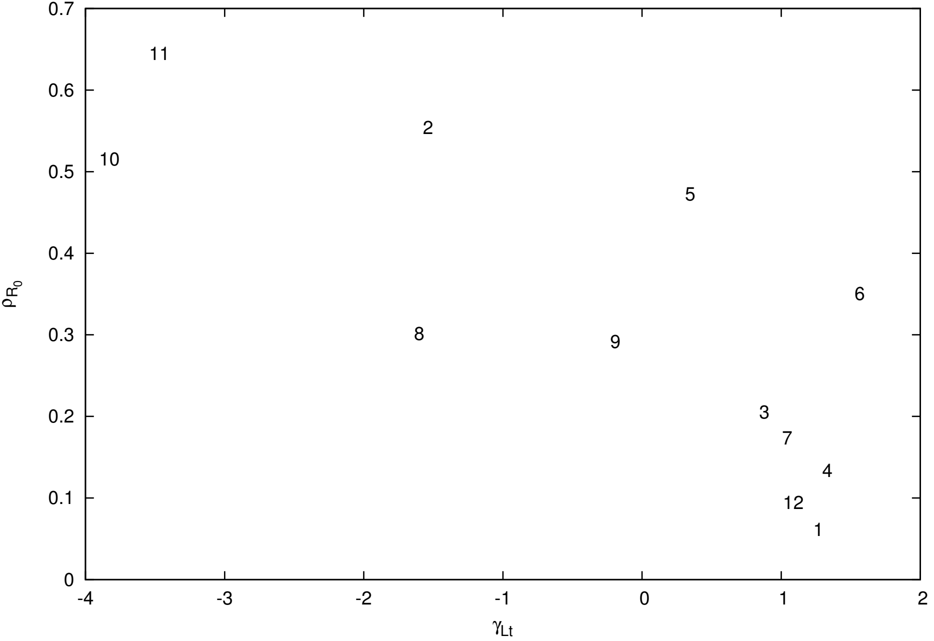

0.7
0.6
0.5
0.4
0.3
0.2
0.1
0
-4
-3
-2
-1
0
1
2
ρ_{R_0}
γ_{Lt}
11
2
10
5
6
8
9
3
7
4
12
1

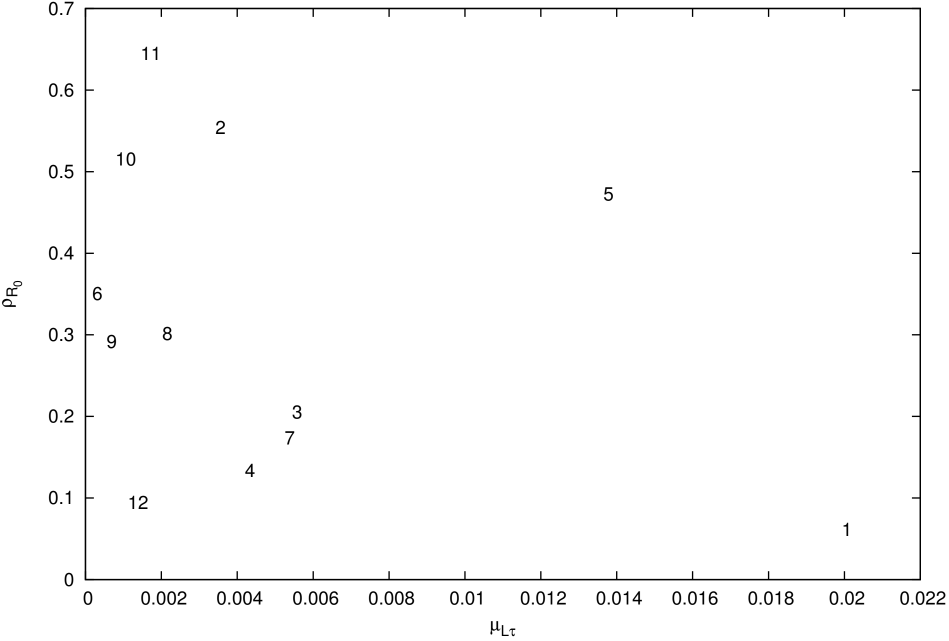

0.7
0.6
0.5
0.4
0.3
0.2
0.1
0
ρ_{R_0}
0
0.002
0.004
0.006
0.008
0.01
0.012
0.014
0.016
0.018
0.02
0.022
$\mu_{L\tau}$
11
2
10
5
6
9
8
3
7
4
12
1

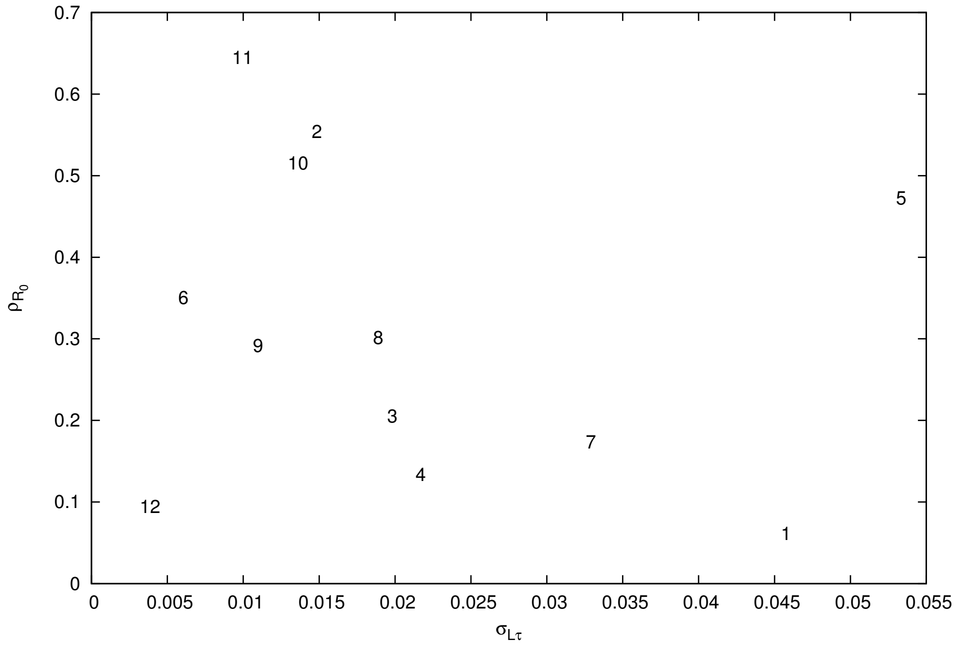
0.7
0.6
0.5
0.4
0.3
0.2
0.1
0
ρ_{R_0}
0
0.005
0.01
0.015
0.02
0.025
0.03
0.035
0.04
0.045
0.05
0.055
$\sigma_{L\tau}$
11
2
10
5
6
8
9
3
7
4
12
1

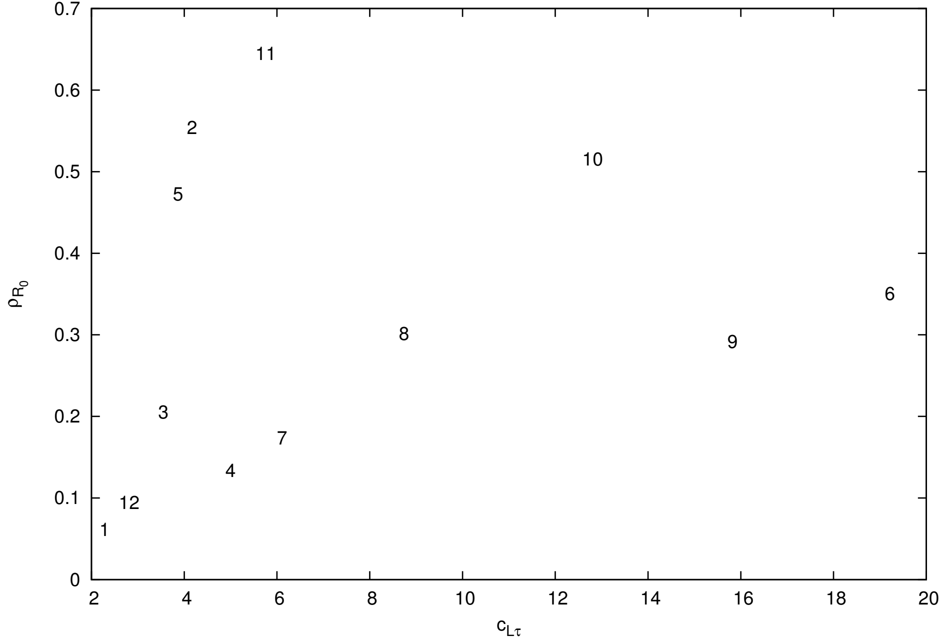

0.7
0.6
0.5
0.4
0.3
0.2
0.1
0
2
4
6
8
10
12
14
16
18
20
ρ_{R_0}
$c_{L\tau}$
11
2
10
5
6
8
9
3
7
4
12
1

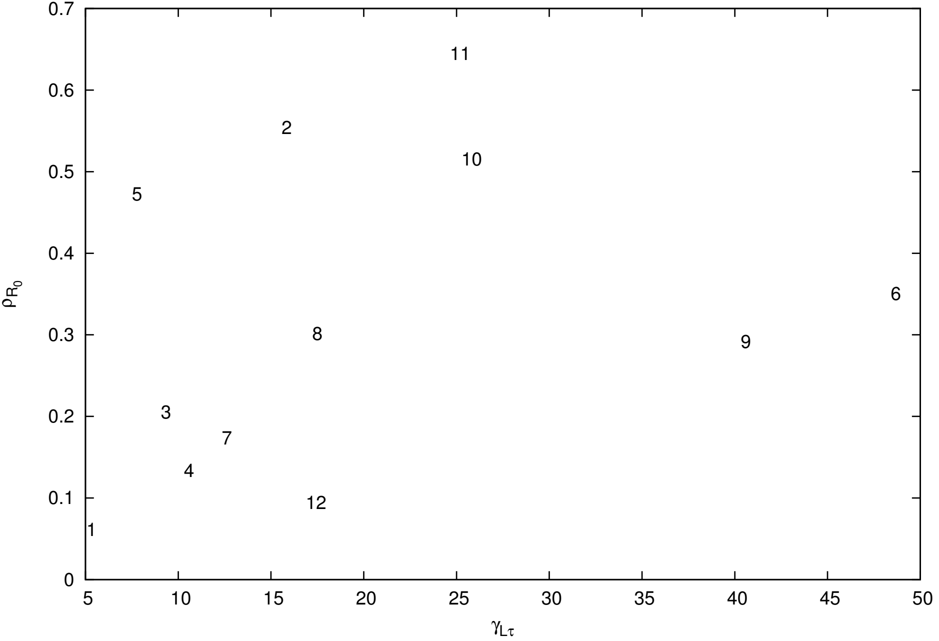

0.7
0.6
0.5
0.4
0.3
0.2
0.1
0
5
10
15
20
25
30
35
40
45
50
ρ_{R_0}
$\gamma_{L\tau}$
11
2
10
5
6
8
9
3
7
4
12
1

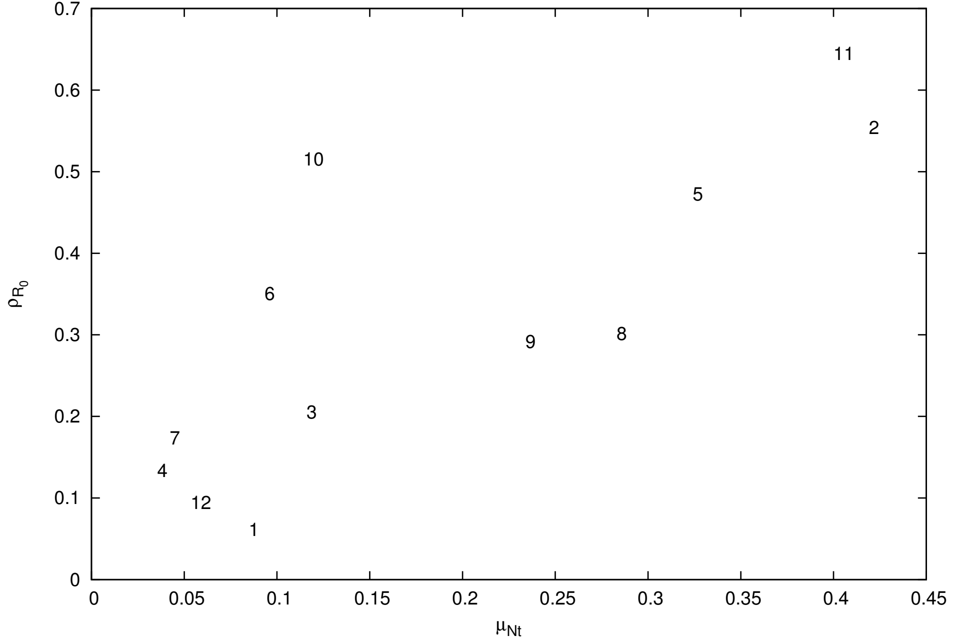

0.7
0.6
0.5
0.4
0.3
0.2
0.1
0
ρ_{R_0}
0
0.05
0.1
0.15
0.2
0.25
0.3
0.35
0.4
0.45
μ_{Nt}
11
2
10
5
6
9
8
3
7
4
12
1

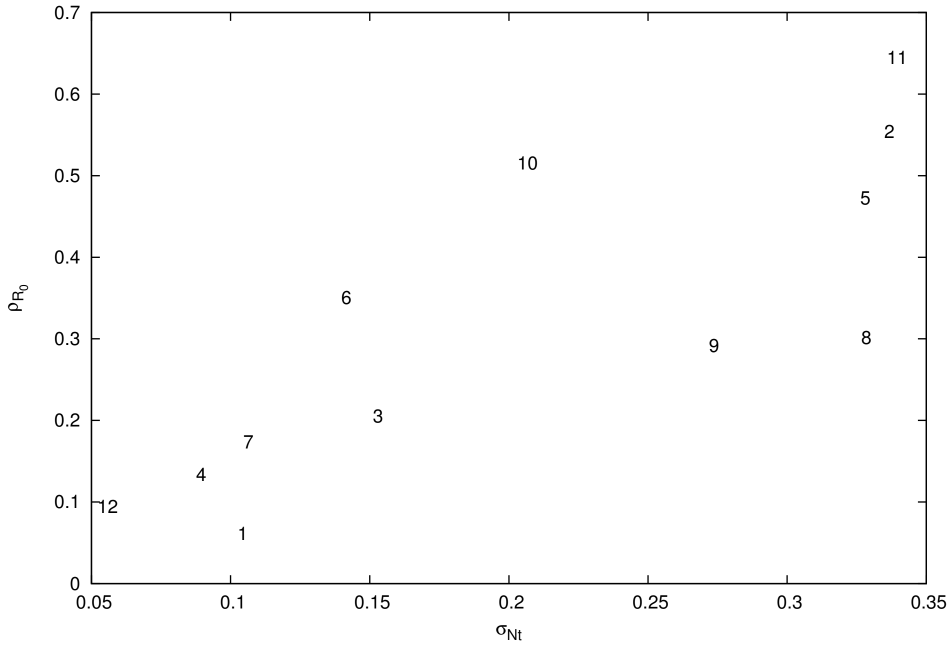

ρ_{R_0}
σ_{Nt}
0
0.1
0.2
0.3
0.4
0.5
0.6
0.7
0.05
0.1
0.15
0.2
0.25
0.3
0.35
1
2
3
4
5
6
7
8
9
10
11
12

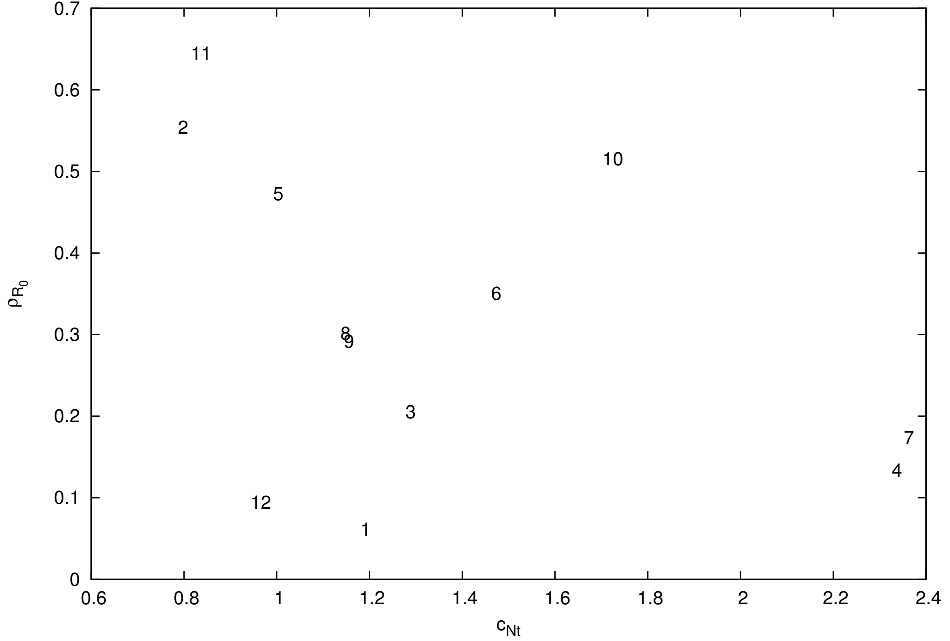

0.7
0.6
0.5
0.4
0.3
0.2
0.1
0
ρ_{R_0}
0.6
0.8
1
1.2
1.4
1.6
1.8
2
2.2
2.4
c_{Nt}
11
2
10
5
6
8
9
3
7
4
12
1

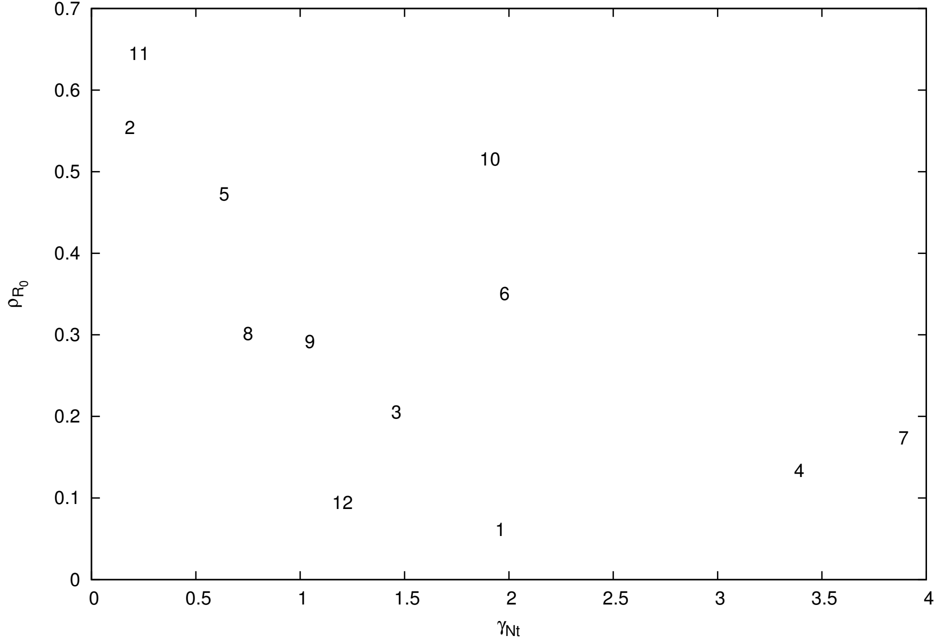

0.7
0.6
0.5
0.4
0.3
0.2
0.1
0
ρ_{R_0}
0
0.5
1
1.5
2
2.5
3
3.5
4
γ_{Nt}
11
2
10
5
6
8
9
3
7
4
12
1

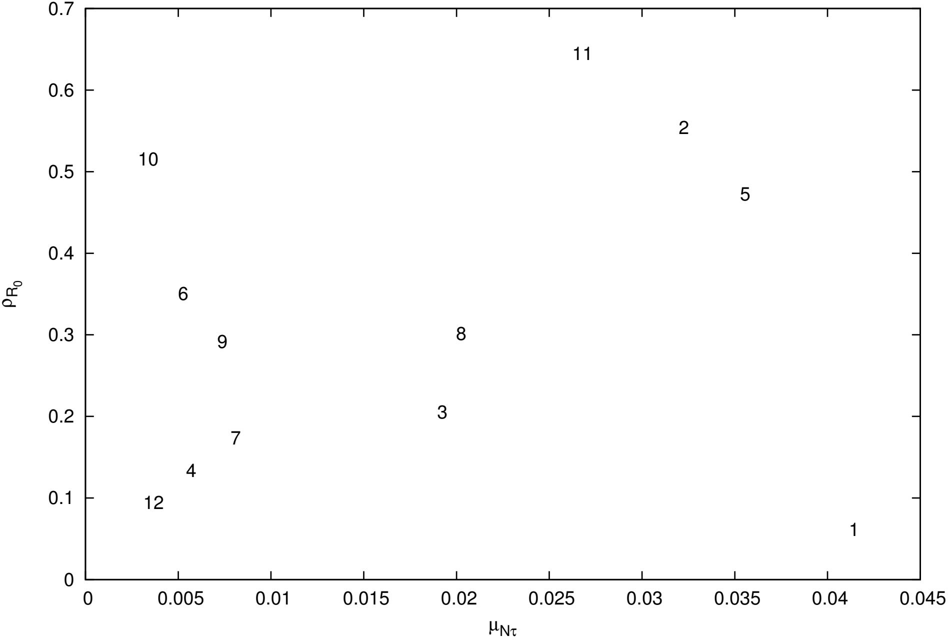

0.7
0.6
0.5
0.4
0.3
0.2
0.1
0
0
0.005
0.01
0.015
0.02
0.025
0.03
0.035
0.04
0.045
ρR0
μNτ
11
2
10
5
6
8
9
3
7
4
12
1

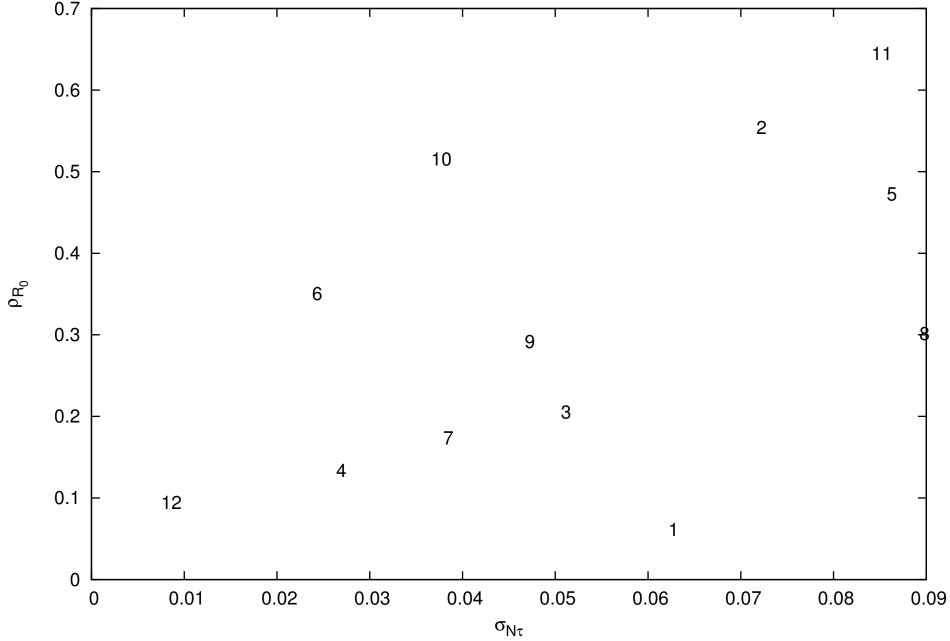

0.7
0.6
0.5
0.4
0.3
0.2
0.1
0
0
0.01
0.02
0.03
0.04
0.05
0.06
0.07
0.08
0.09
ρ_{R_0}
$\sigma_{N\tau}$
11
2
10
5
6
9
8
3
7
4
12
1

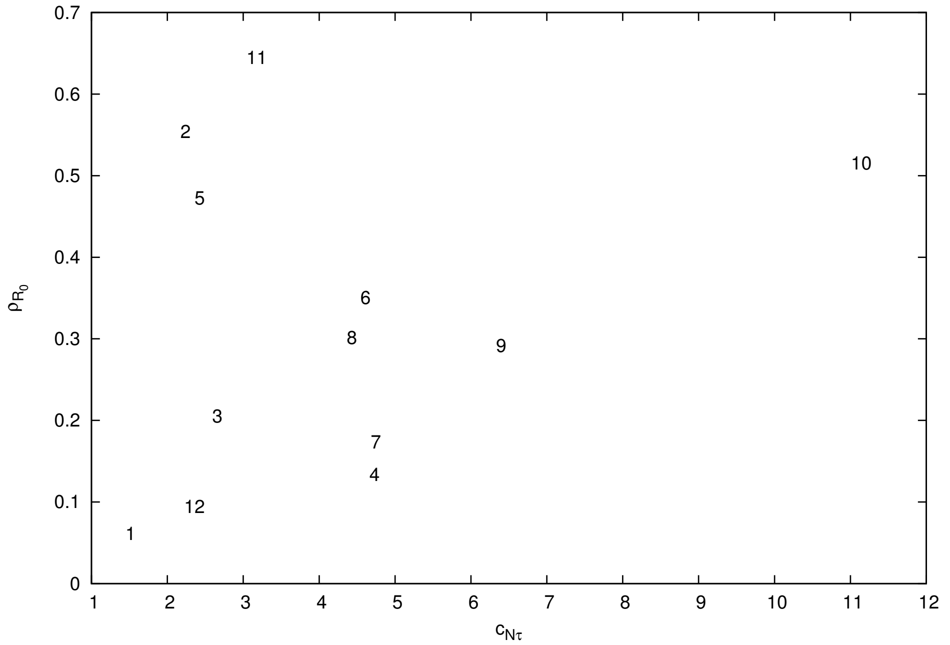

0.7
0.6
0.5
0.4
0.3
0.2
0.1
0
1
2
3
4
5
6
7
8
9
10
11
12
ρ_{R_0}
$c_{N\tau}$
11
2
10
5
6
8
9
3
7
4
12
1

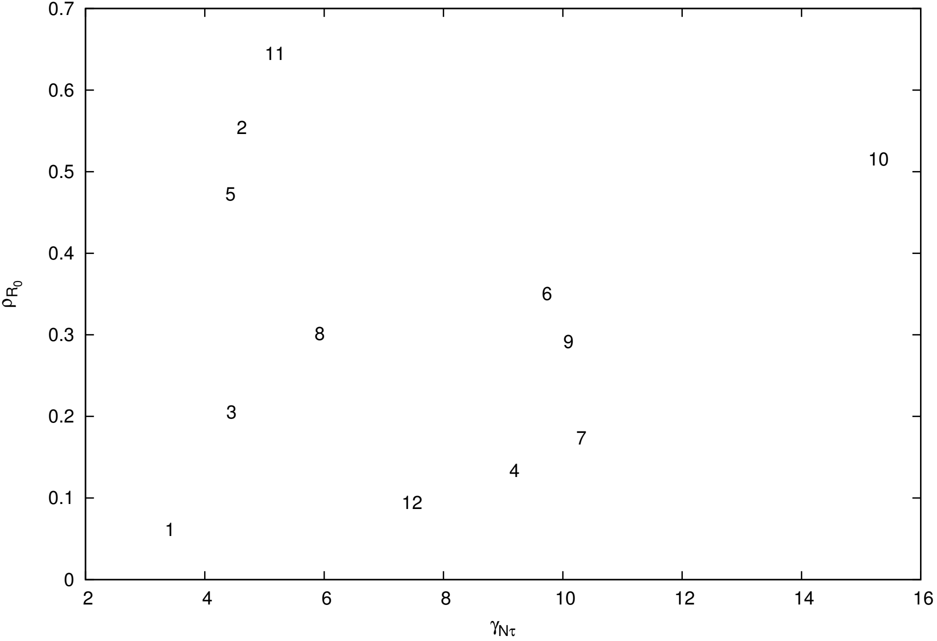

0.7
0.6
0.5
0.4
0.3
0.2
0.1
0
2
4
6
8
10
12
14
16
ρ_{R_0}
$\gamma_{N\tau}$
11
2
10
5
6
8
9
3
7
4
12
1

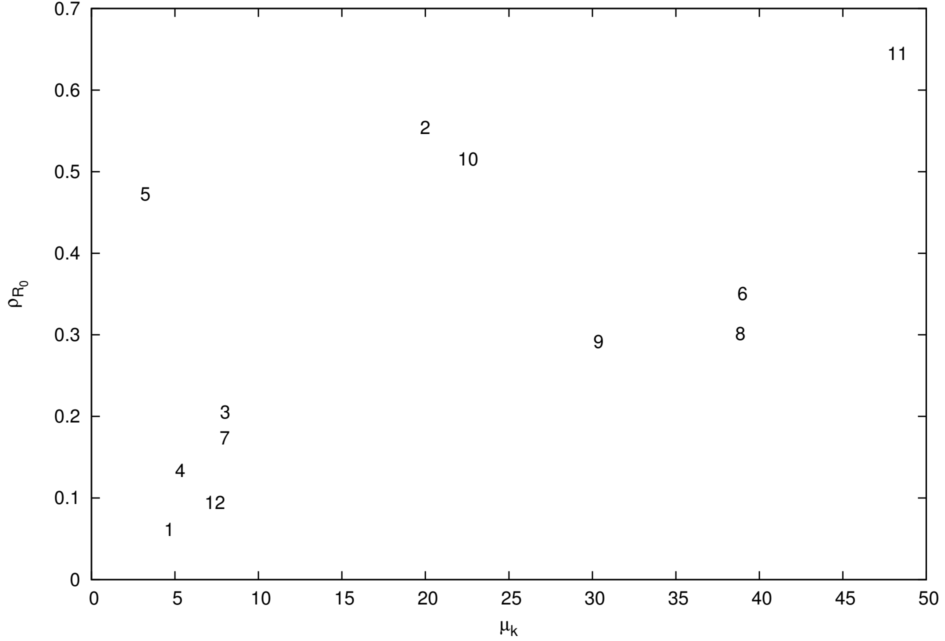

0.7
0.6
0.5
0.4
0.3
0.2
0.1
0
ρ_{R_0}
0
5
10
15
20
25
30
35
40
45
50
μ_k
11
2
10
5
6
9
8
3
7
4
12
1

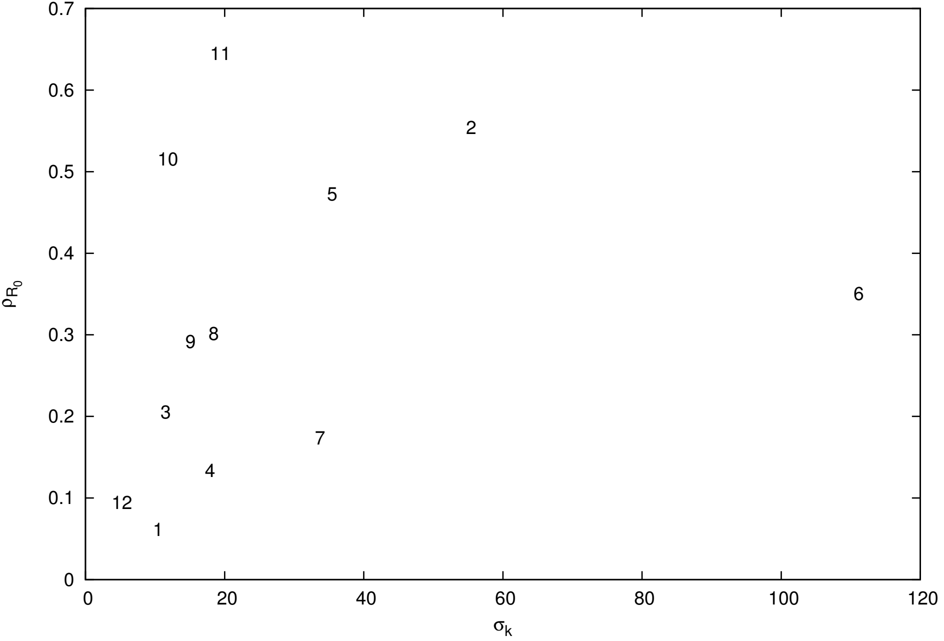

0.7
0.6
0.5
0.4
0.3
0.2
0.1
0
0
20
40
60
80
100
120
ρ_{R_0}
σ_k
11
2
10
5
6
9
8
3
7
4
12
1

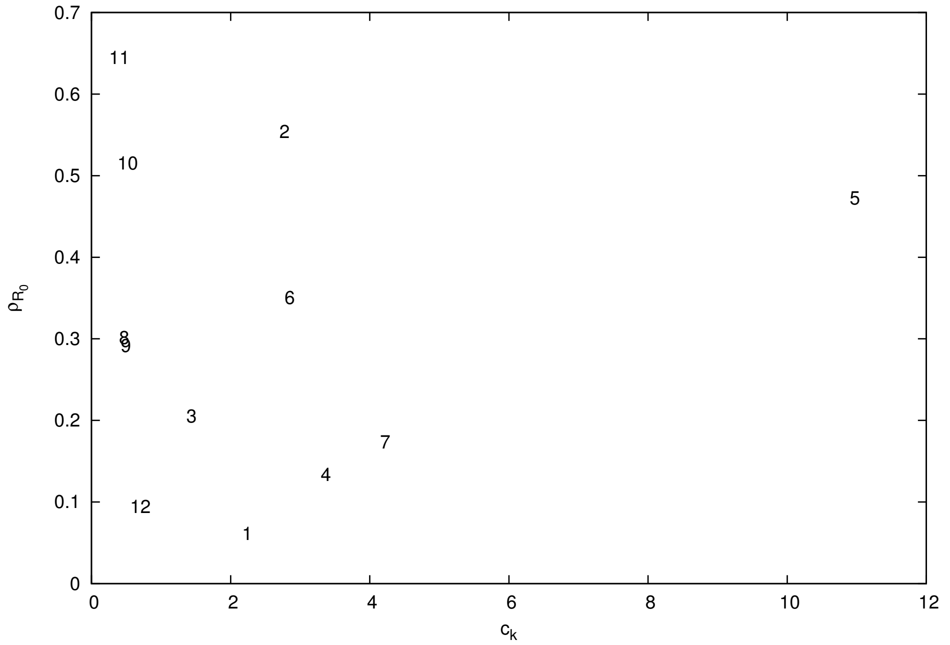
0.7
0.6
0.5
0.4
0.3
0.2
0.1
0
0
2
4
6
8
10
12
ρ_{R_0}
c_k
11
2
10
5
6
8
9
3
7
4
12
1

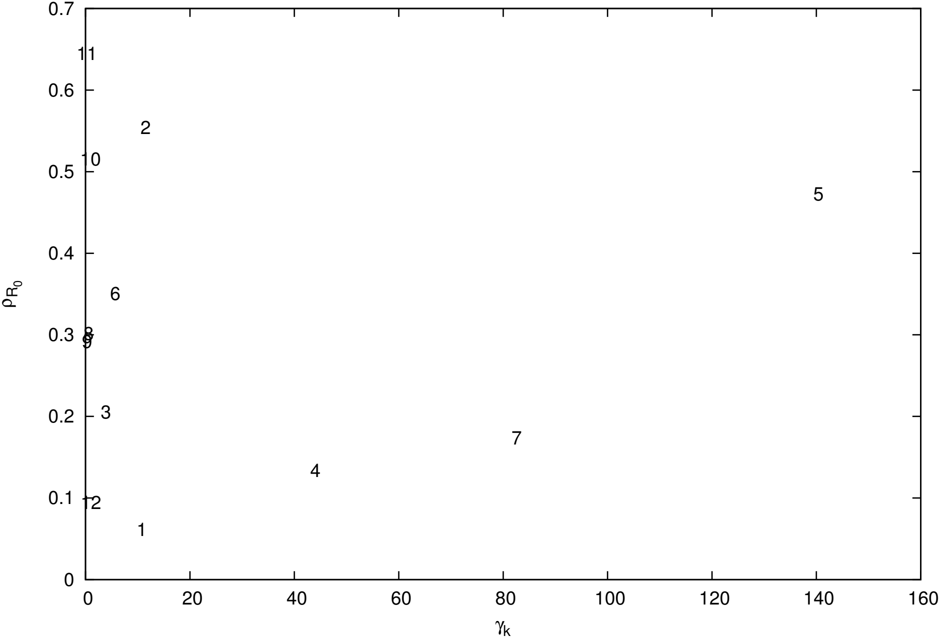

0.7
0.6
0.5
0.4
0.3
0.2
0.1
0
0
20
40
60
80
100
120
140
160
ρ_{R_0}
γ_k
11
2
10
5
6
8
9
3
7
4
12
1

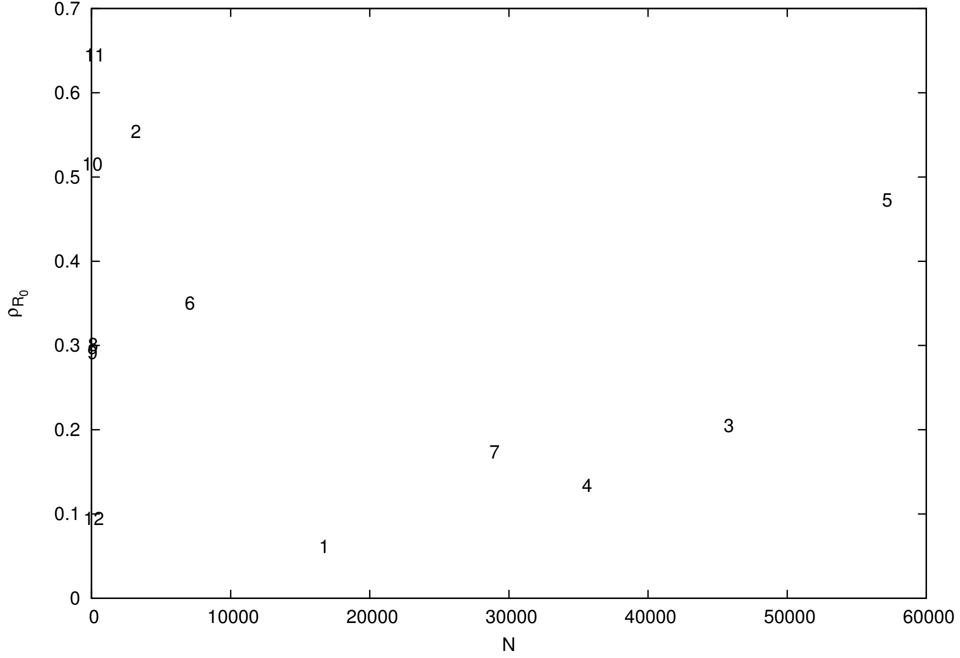

0.7
0.6
0.5
0.4
0.3
0.2
0.1
0
0
10000
20000
30000
40000
50000
60000
N
ρ_{R_0}
11
2
10
5
6
8
9
3
7
4
12
1

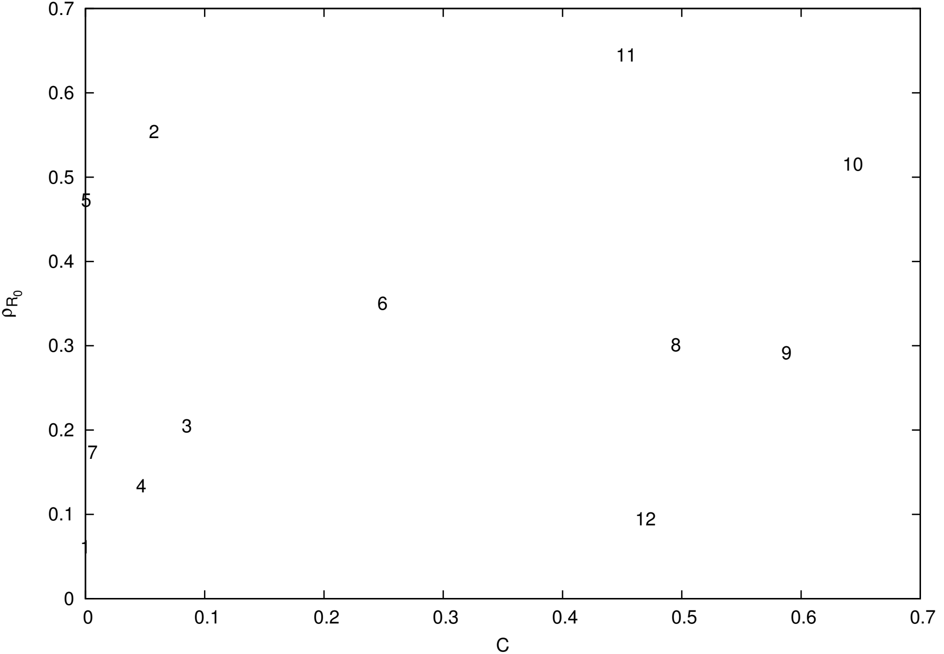

0.7
0.6
0.5
0.4
0.3
0.2
0.1
0
0
0.1
0.2
0.3
0.4
0.5
0.6
0.7
C
ρ_{R_0}
1
2
3
4
5
6
7
8
9
10
11
12

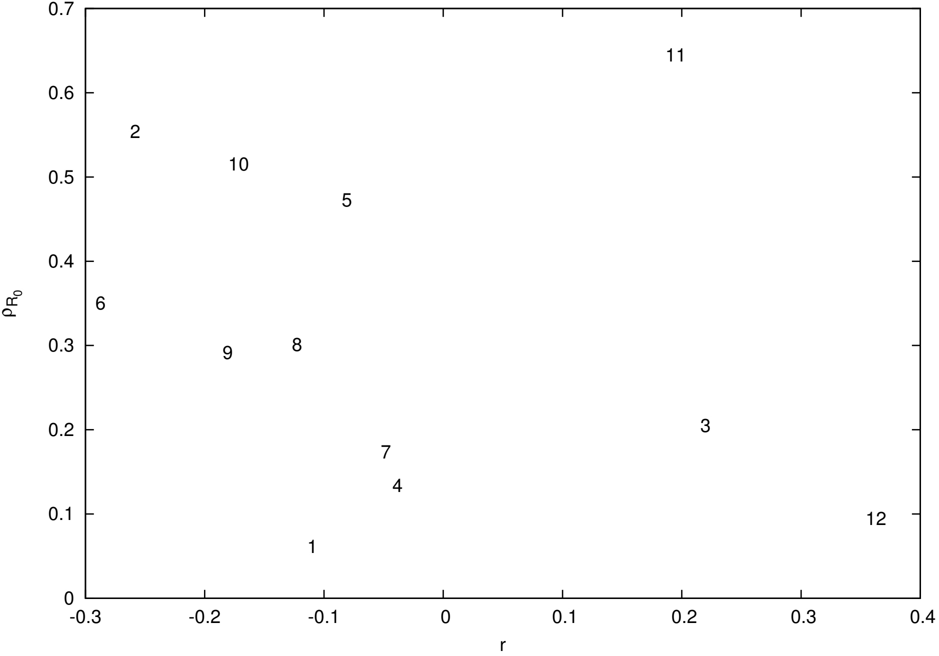

0.7
0.6
0.5
0.4
0.3
0.2
0.1
0
-0.3
-0.2
-0.1
0
0.1
0.2
0.3
0.4
ρ_{R_0}
r
11
2
10
5
6
9
8
3
7
4
12
1

Δ_{R_0}

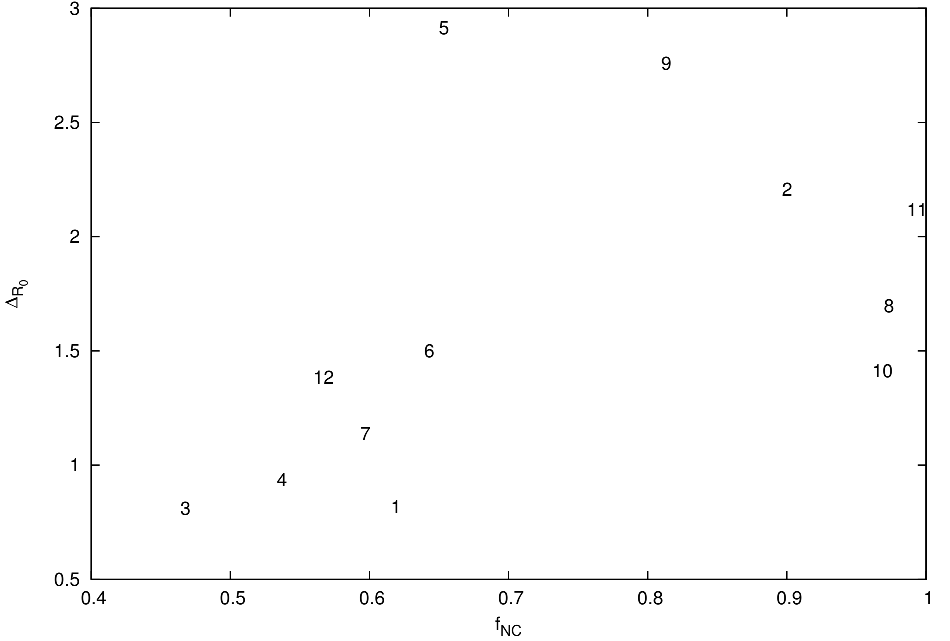

3
2.5
2
1.5
1
0.5
0.4
0.5
0.6
0.7
0.8
0.9
1
Δ_{R_0}
f_{NC}
5
9
2
11
8
6
10
12
7
4
3
1

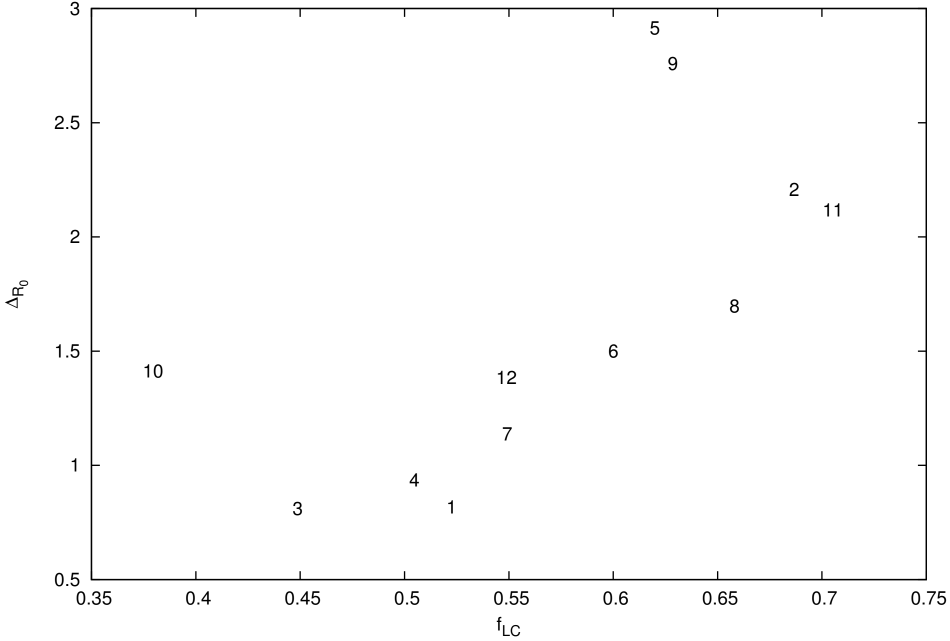

Δ_{R_0}
f_{LC}
0.5
1
1.5
2
2.5
3
0.35
0.4
0.45
0.5
0.55
0.6
0.65
0.7
0.75
5
9
2
11
8
6
10
12
7
4
3
1

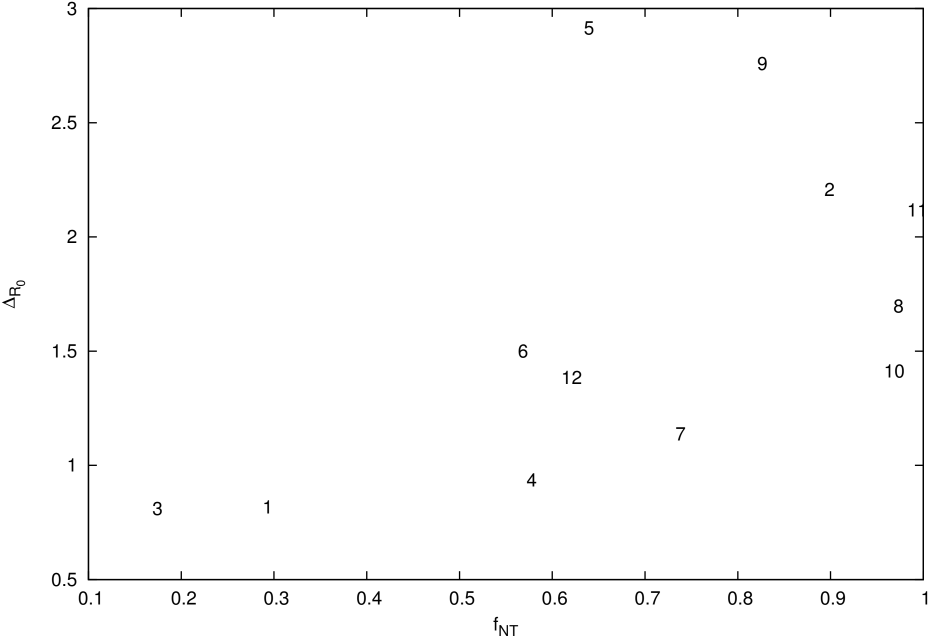

3
2.5
2
1.5
1
0.5
0.1
0.2
0.3
0.4
0.5
0.6
0.7
0.8
0.9
1
Δ_{R_0}
f_{NT}
5
9
2
11
8
6
12
10
7
4
3
1

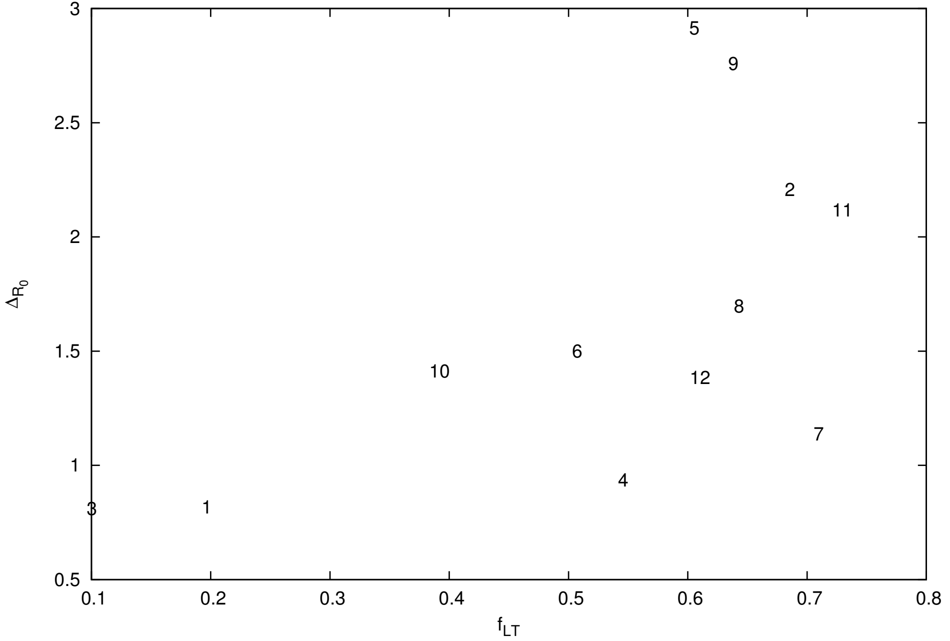

Δ_{R_0}
f_{LT}
0.1
0.2
0.3
0.4
0.5
0.6
0.7
0.8
0.5
1
1.5
2
2.5
3
1
2
3
4
5
6
7
8
9
10
11
12

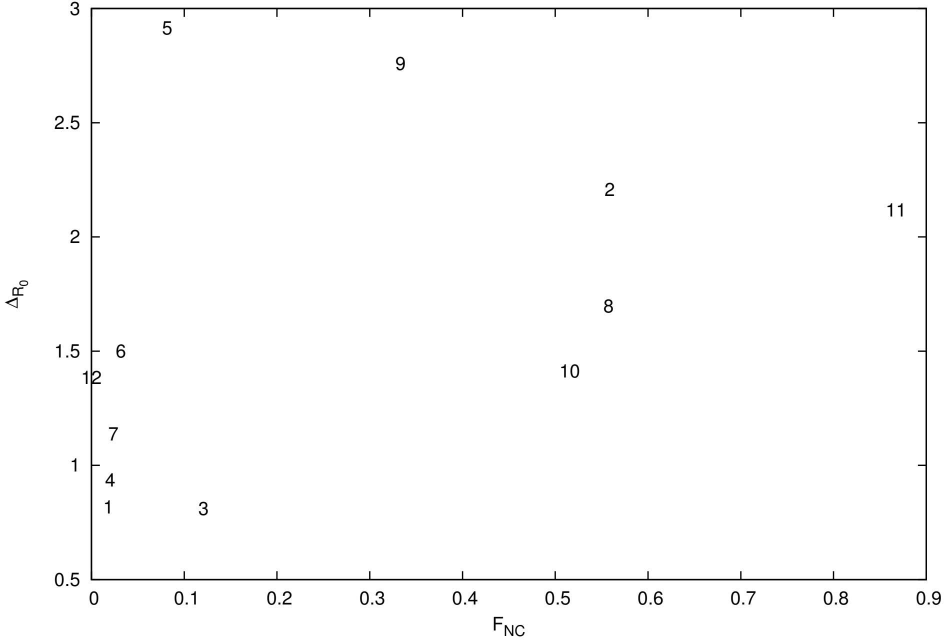

3
2.5
2
1.5
1
0.5
0
0.1
0.2
0.3
0.4
0.5
0.6
0.7
0.8
0.9
Δ_{R_0}
F_{NC}
5
9
2
11
8
6
12
10
7
4
1
3

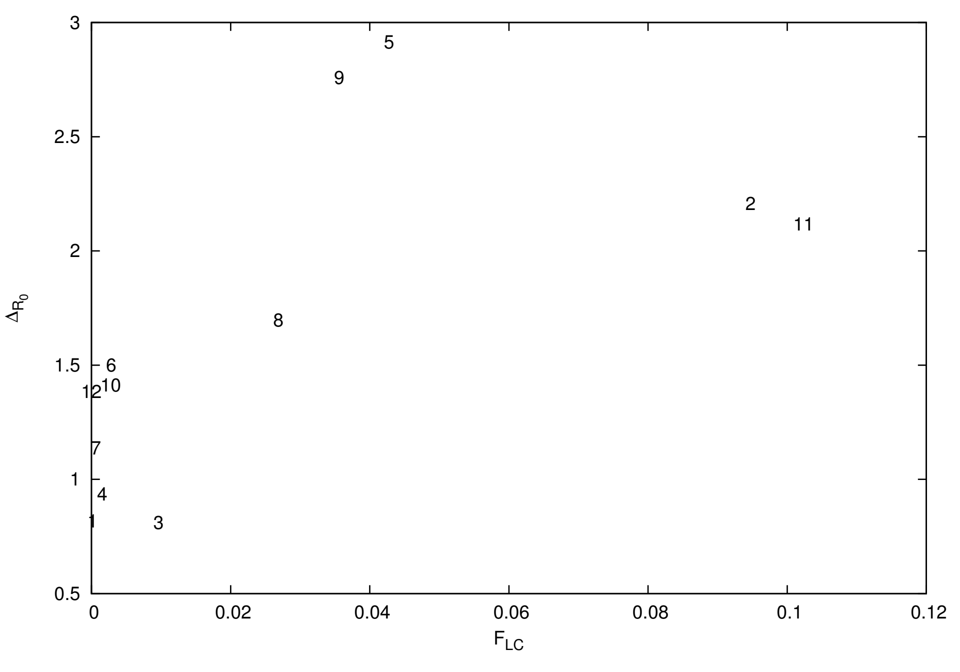

3
2.5
2
1.5
1
0.5
0
0.02
0.04
0.06
0.08
0.1
0.12
F_{LC}
Δ_{R_0}
5
9
2
11
8
6
12
10
7
4
1
3

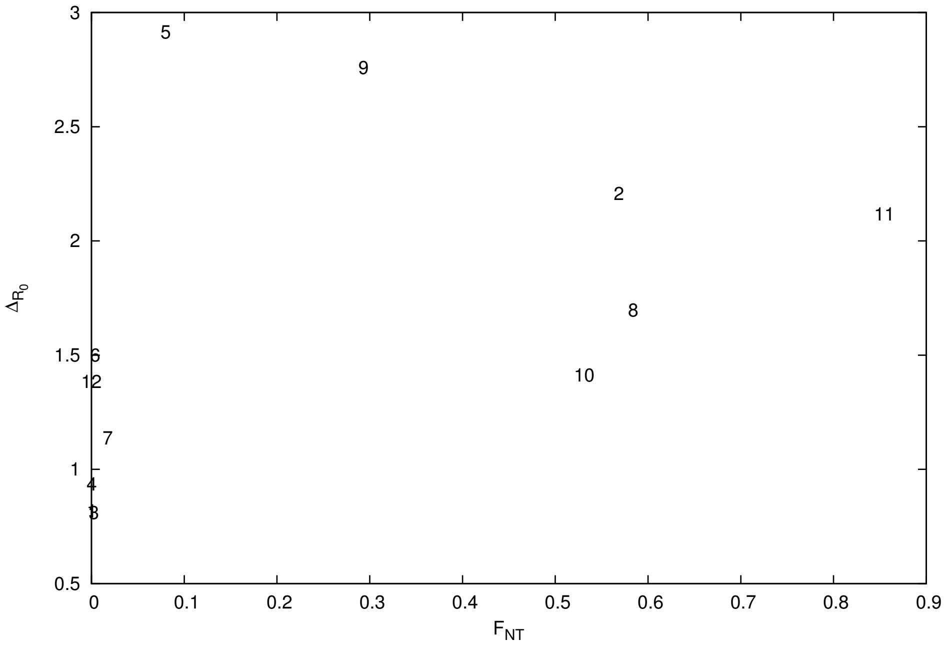

3
2.5
2
1.5
1
0.5
0
0.1
0.2
0.3
0.4
0.5
0.6
0.7
0.8
0.9
Δ_{R_0}
F_{NT}
5
9
2
11
8
6
12
10
7
4
3

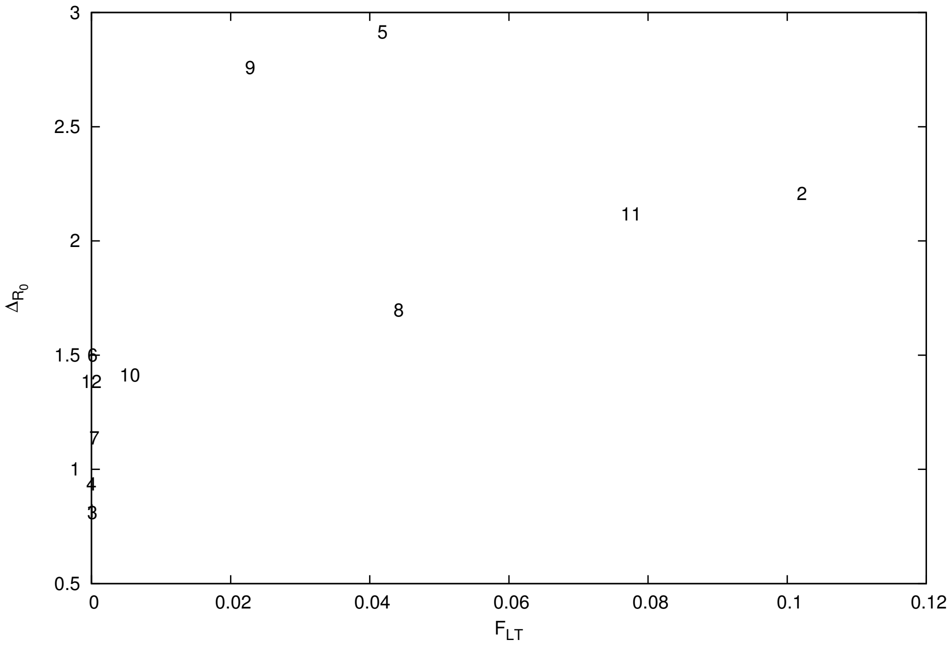

3
2.5
2
1.5
1
0.5
0
0.02
0.04
0.06
0.08
0.1
0.12
Δ_{R_0}
F_{LT}
5
9
2
11
8
6
12
10
7
4
3

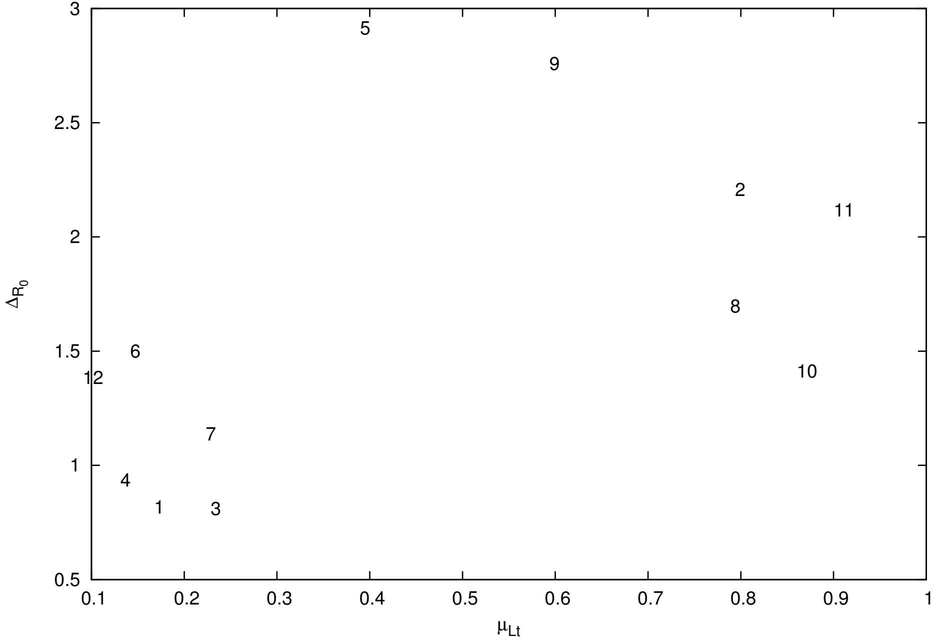

Δ_{R_0}
μ_{Lt}
0.5
1
1.5
2
2.5
3
0.1
0.2
0.3
0.4
0.5
0.6
0.7
0.8
0.9
1
1
2
3
4
5
6
7
8
9
10
11
12

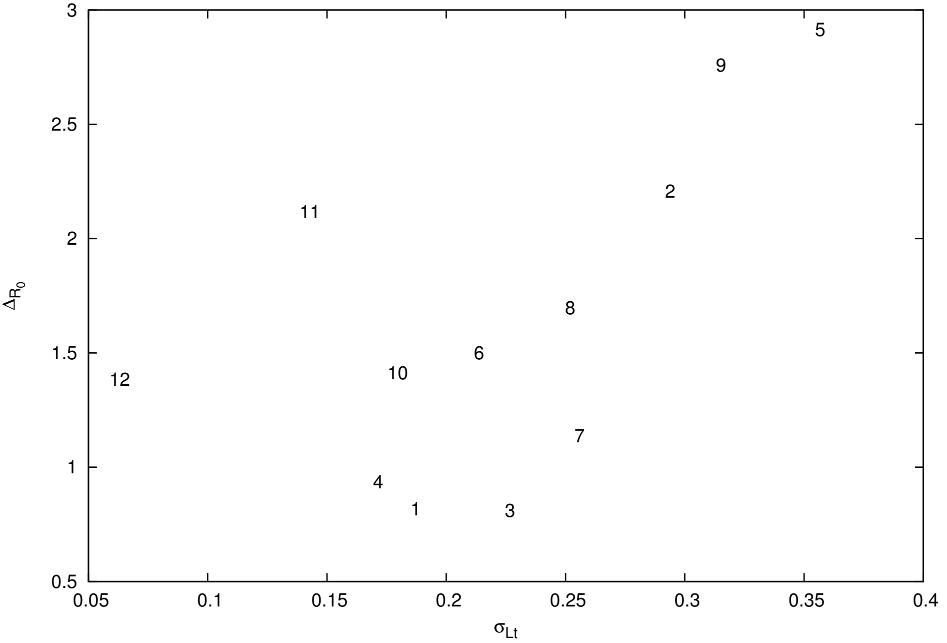

3
2.5
2
1.5
1
0.5
0.05
0.1
0.15
0.2
0.25
0.3
0.35
0.4
Δ_{R_0}
σ_{Lt}
5
9
2
11
8
6
10
12
7
4
1
3

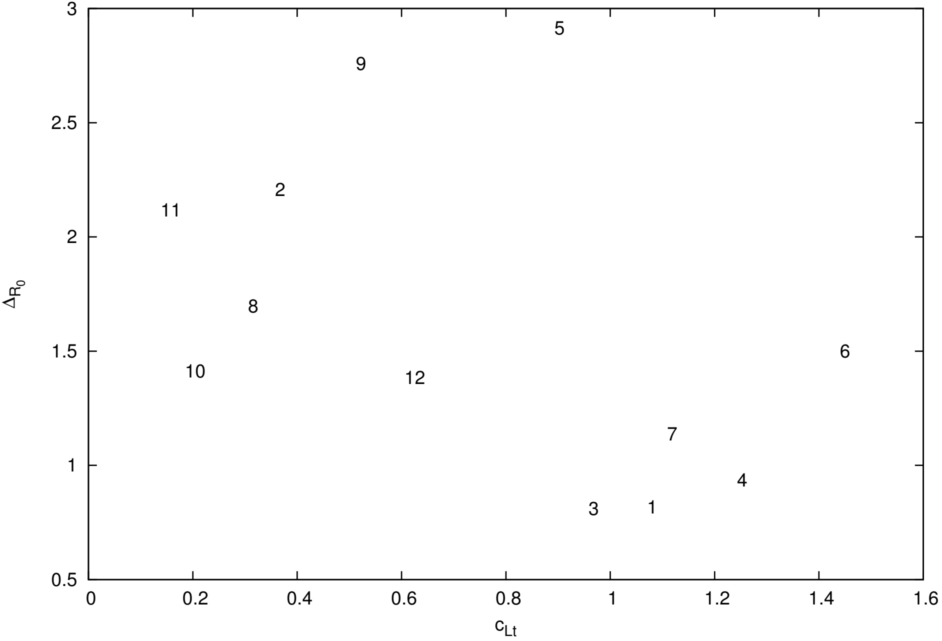

3
2.5
2
1.5
1
0.5
0
0.2
0.4
0.6
0.8
1
1.2
1.4
1.6
Δ_{R_0}
c_{Lt}
1
2
3
4
5
6
7
8
9
10
11
12

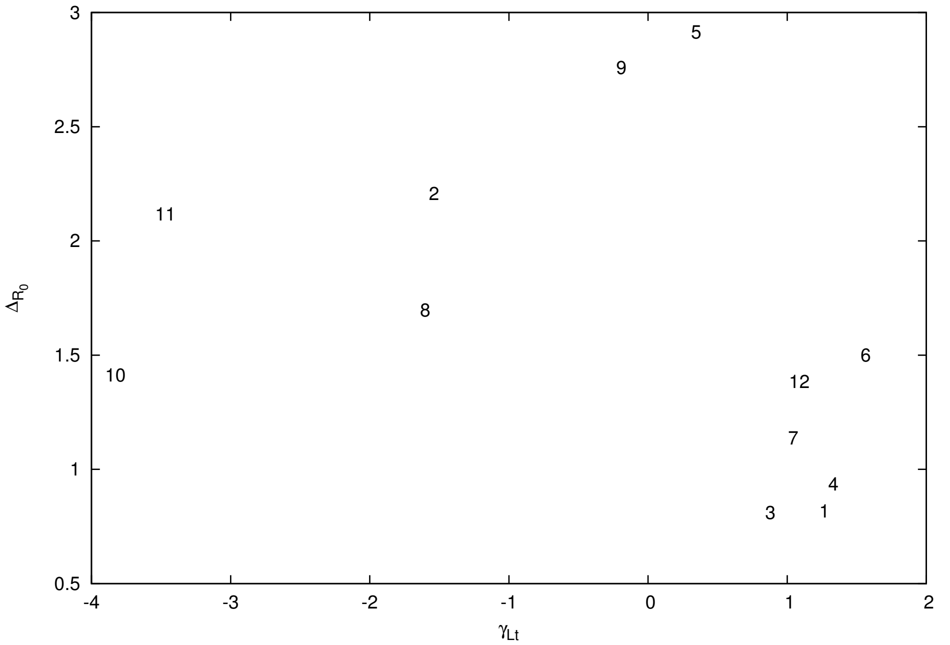

3
2.5
2
1.5
1
0.5
-4
-3
-2
-1
0
1
2
γ_{Lt}
Δ_{R_0}
5
9
2
11
8
6
10
12
7
4
3
1

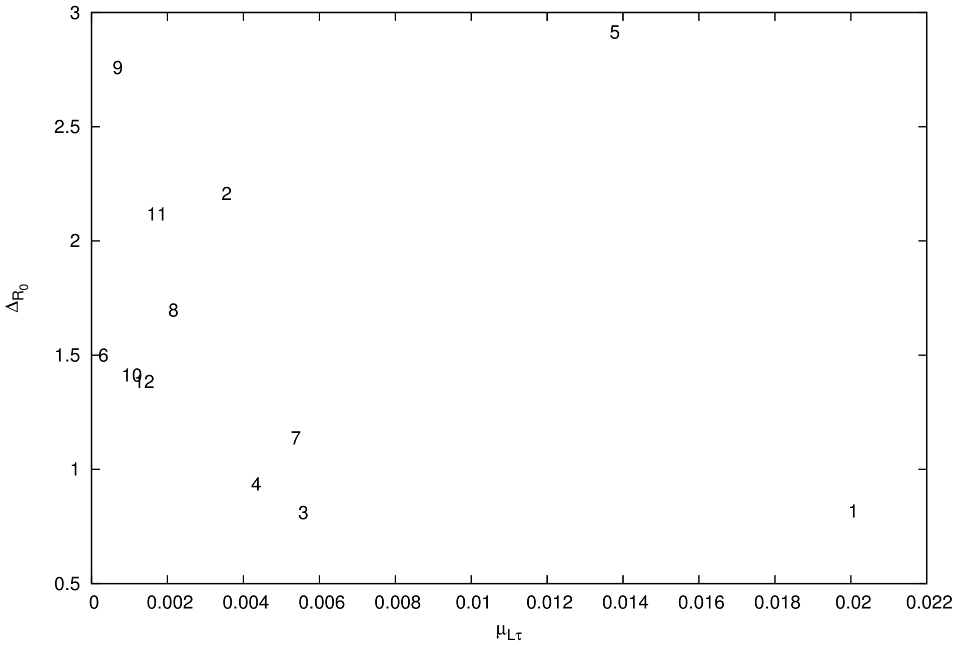

3
2.5
2
1.5
1
0.5
0
0.002
0.004
0.006
0.008
0.01
0.012
0.014
0.016
0.018
0.02
0.022
Δ_{R_0}
$\mu_{L\tau}$
1
2
3
4
5
6
7
8
9
10
11
12

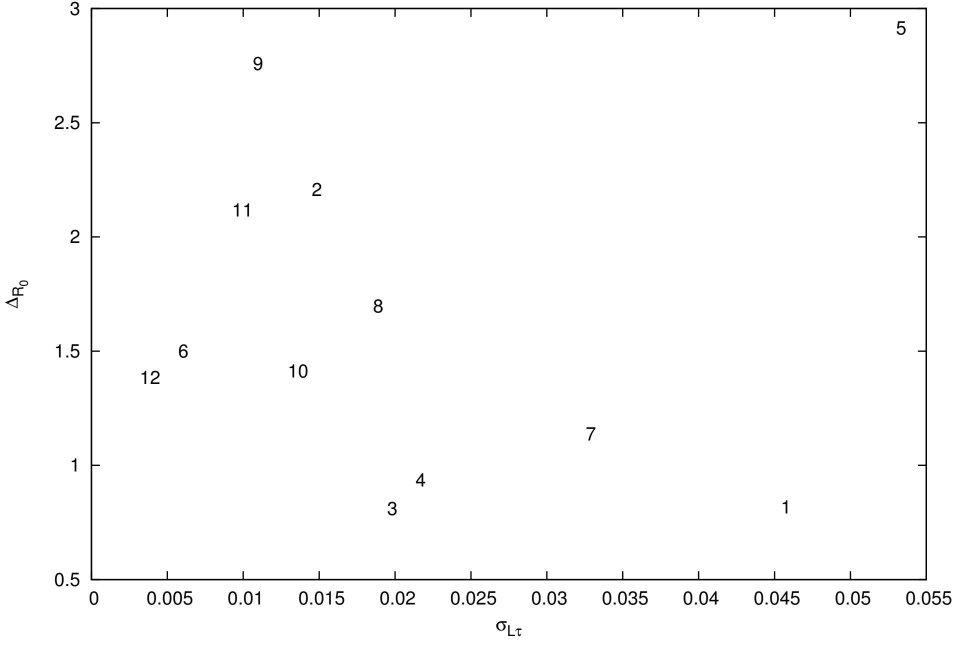
3
2.5
2
1.5
1
0.5
Δ_{R_0}
0
0.005
0.01
0.015
0.02
0.025
0.03
0.035
0.04
0.045
0.05
0.055
$\sigma_{L\tau}$
5
9
2
11
8
6
10
12
7
4
3
1

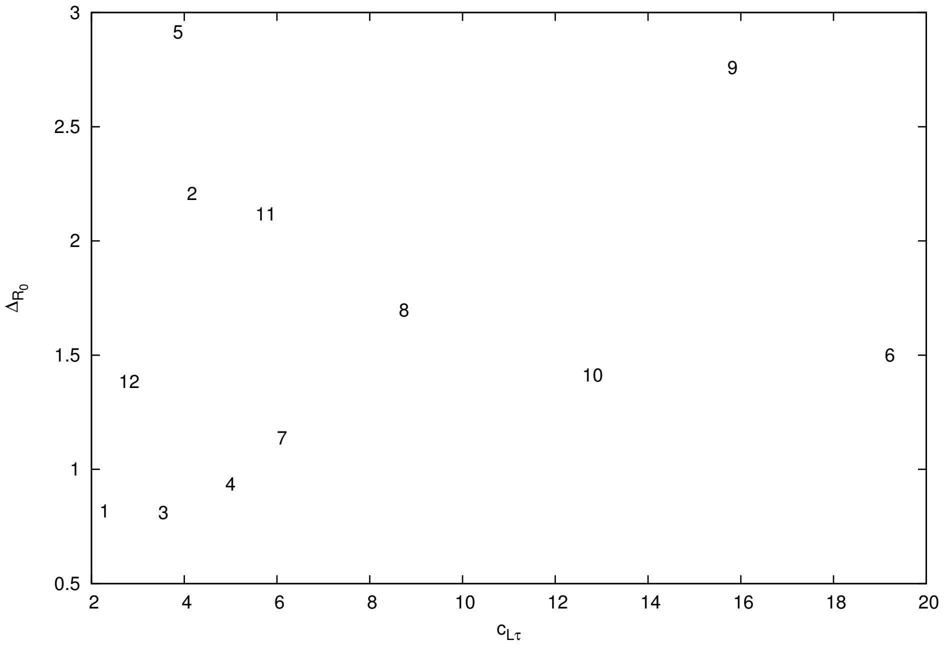

3
2.5
2
1.5
1
0.5
Δ_{R_0}
2
4
6
8
10
12
14
16
18
20
$c_{L\tau}$
5
9
2
11
8
6
12
10
7
4
1
3

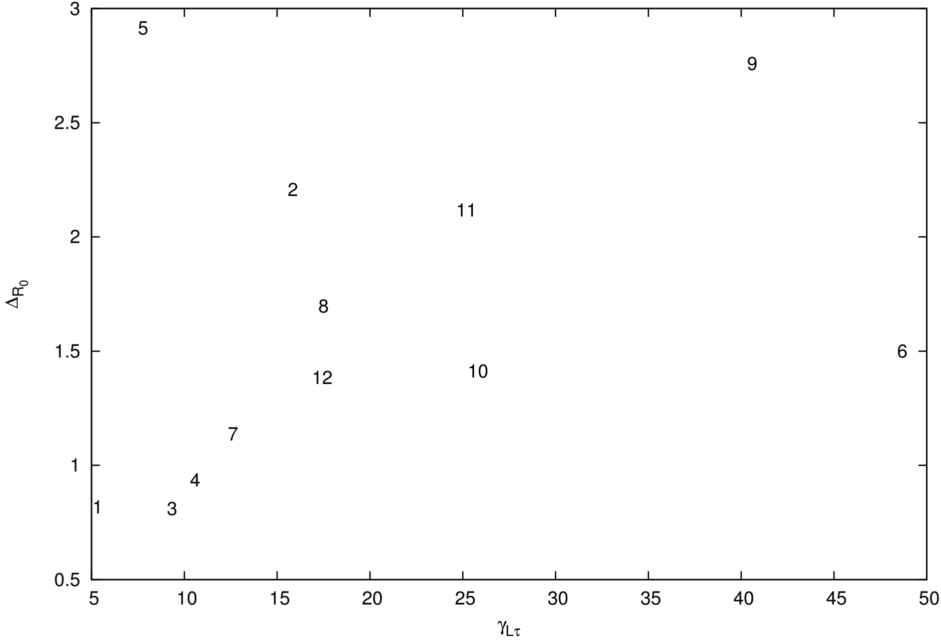

3
2.5
2
1.5
1
0.5
5
10
15
20
25
30
35
40
45
50
Δ_{R_0}
$\gamma_{L\tau}$
5
9
2
11
8
6
12
10
7
4
1
3

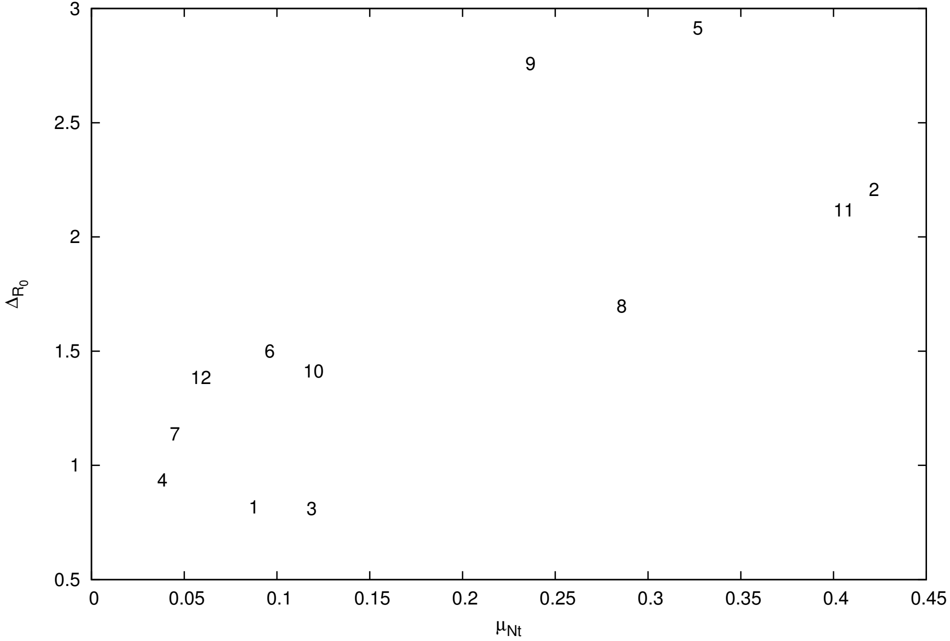

3
2.5
2
1.5
1
0.5
0
0.05
0.1
0.15
0.2
0.25
0.3
0.35
0.4
0.45
Δ_{R_0}
μ_{Nt}
1
2
3
4
5
6
7
8
9
10
11
12

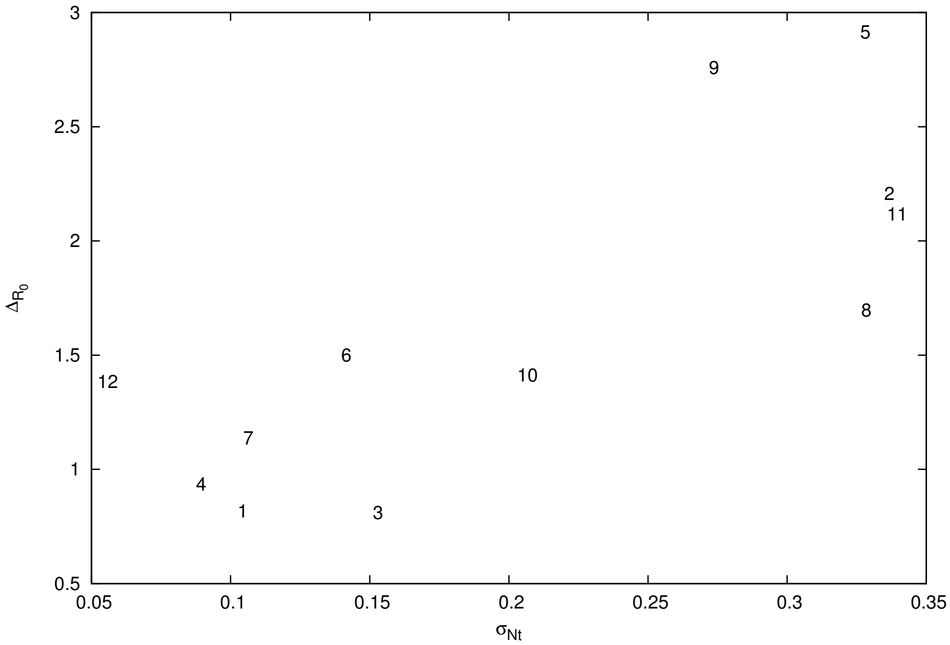

3
2.5
2
1.5
1
0.5
0.05
0.1
0.15
0.2
0.25
0.3
0.35
Δ_{R_0}
σ_{Nt}
5
9
2
11
8
6
10
12
7
4
1
3

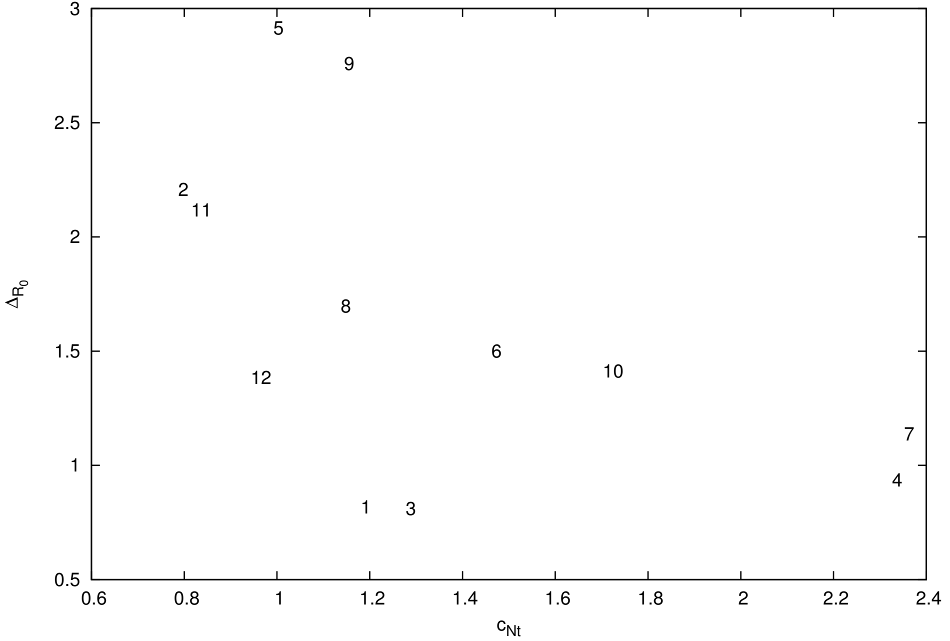

3
2.5
2
1.5
1
0.5
0.6
0.8
1
1.2
1.4
1.6
1.8
2
2.2
2.4
Δ_{R_0}
c_{Nt}
5
9
2
11
8
6
10
12
7
4
1
3

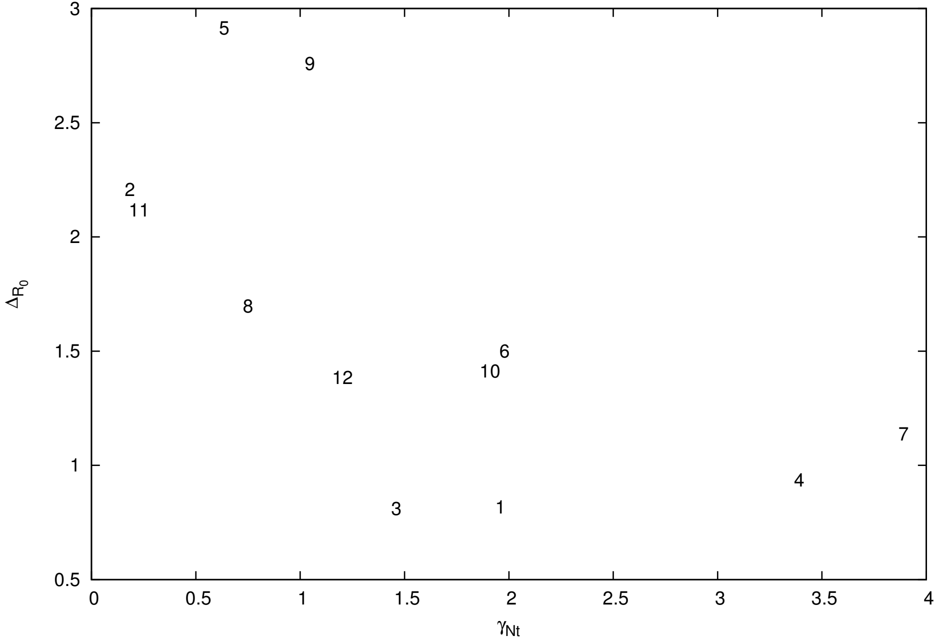

3
2.5
2
1.5
1
0.5
0
0.5
1
1.5
2
2.5
3
3.5
4
Δ_{R_0}
γ_{Nt}
5
9
2
11
8
6
10
12
7
4
3
1

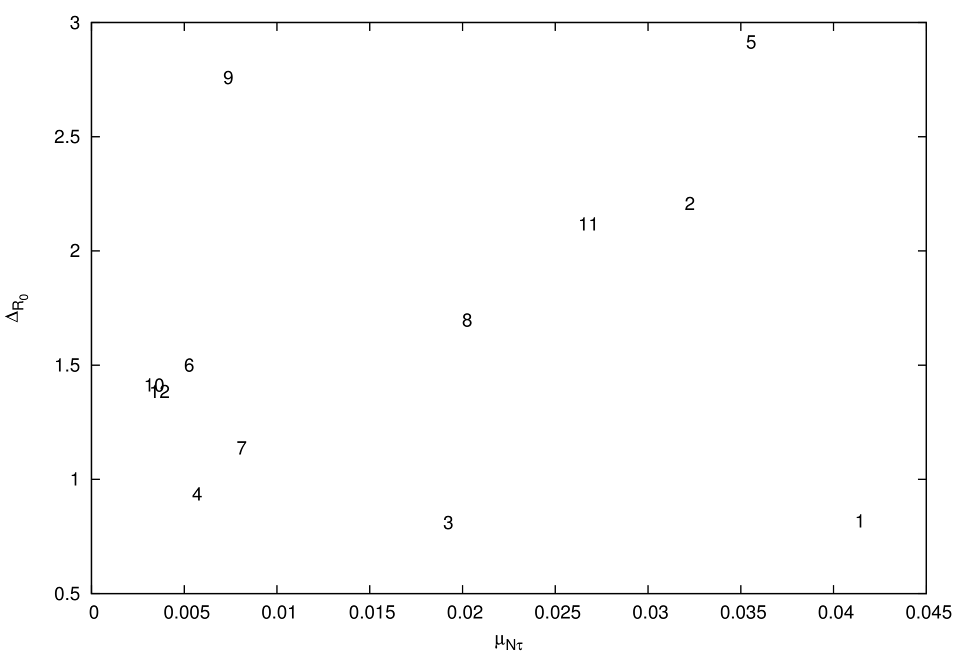

3
2.5
2
1.5
1
0.5
0
0.005
0.01
0.015
0.02
0.025
0.03
0.035
0.04
0.045
Δ_{R_0}
$\mu_{N\tau}$
5
9
2
11
8
6
10
12
7
4
3
1

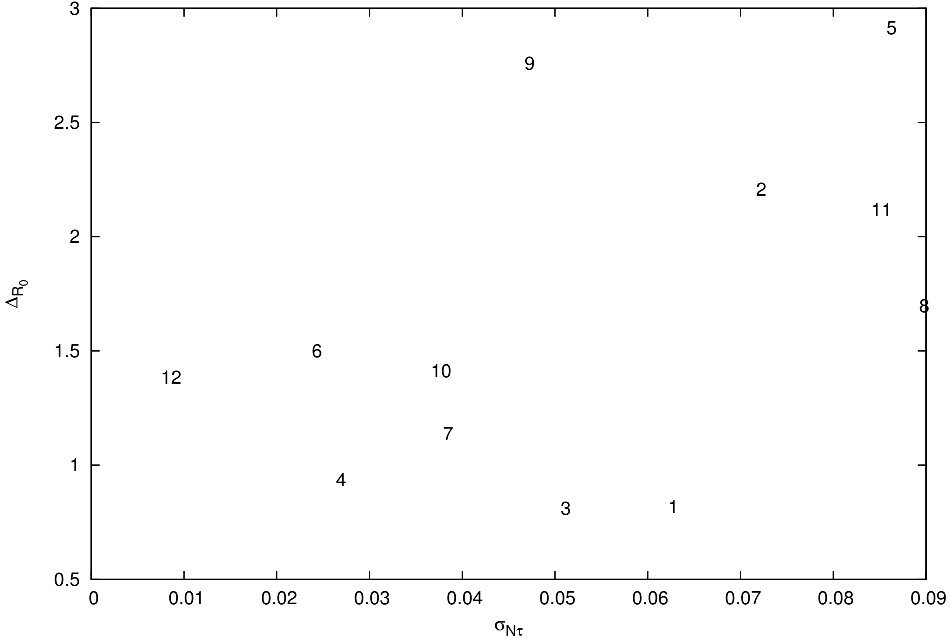

3
2.5
2
1.5
1
0.5
0
0.01
0.02
0.03
0.04
0.05
0.06
0.07
0.08
0.09
Δ_{R_0}
$\sigma_{N\tau}$
9
5
2
11
8
6
12
10
7
4
3
1

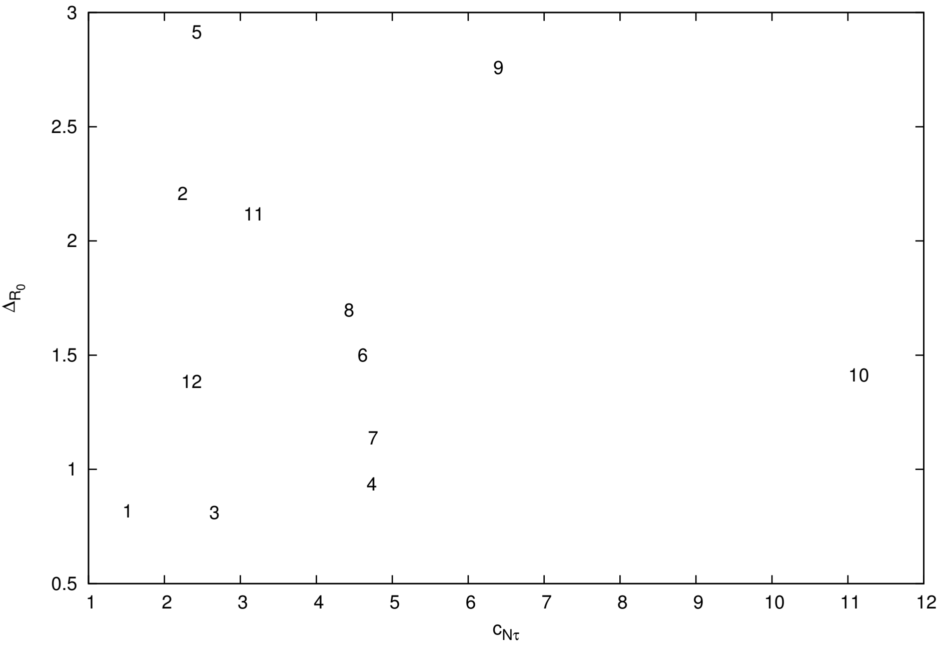

3
2.5
2
1.5
1
0.5
Δ_{R_0}
1
2
3
4
5
6
7
8
9
10
11
12
$c_{N\tau}$
5
9
2
11
8
6
12
10
7
4
1
3

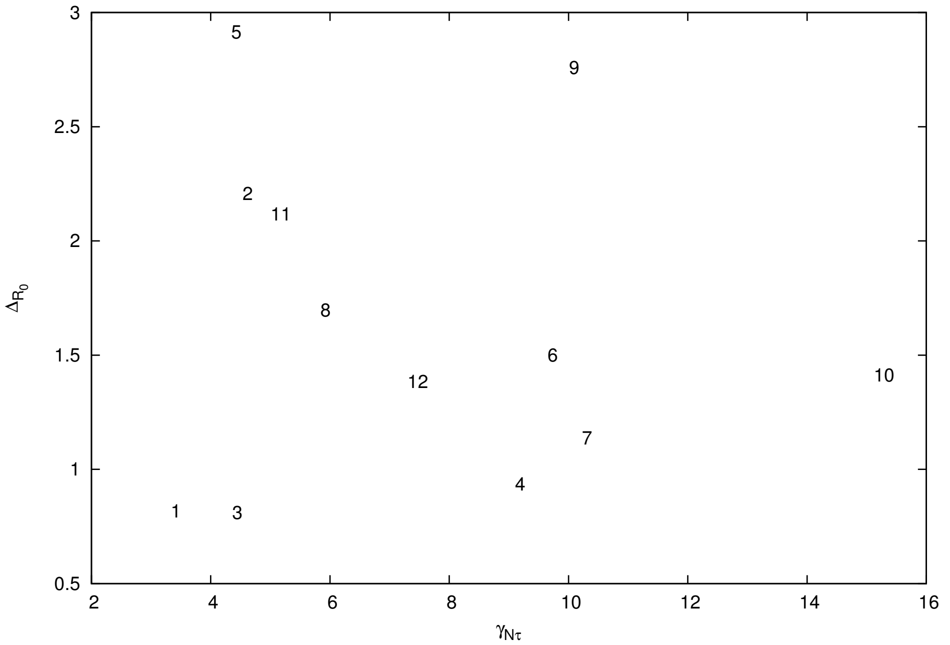

3
2.5
2
1.5
1
0.5
Δ_{R_0}
2
4
6
8
10
12
14
16
$\gamma_{N\tau}$
5
9
2
11
8
6
12
10
7
4
1
3

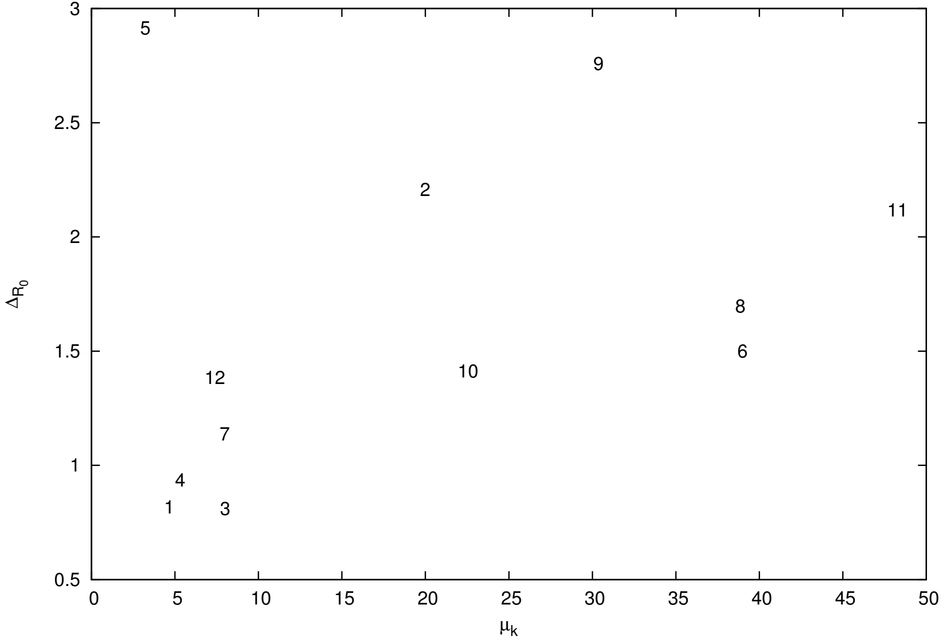

3
2.5
2
1.5
1
0.5
0
5
10
15
20
25
30
35
40
45
50
Δ_{R_0}
μ_k
5
9
2
11
8
6
10
12
7
4
1
3

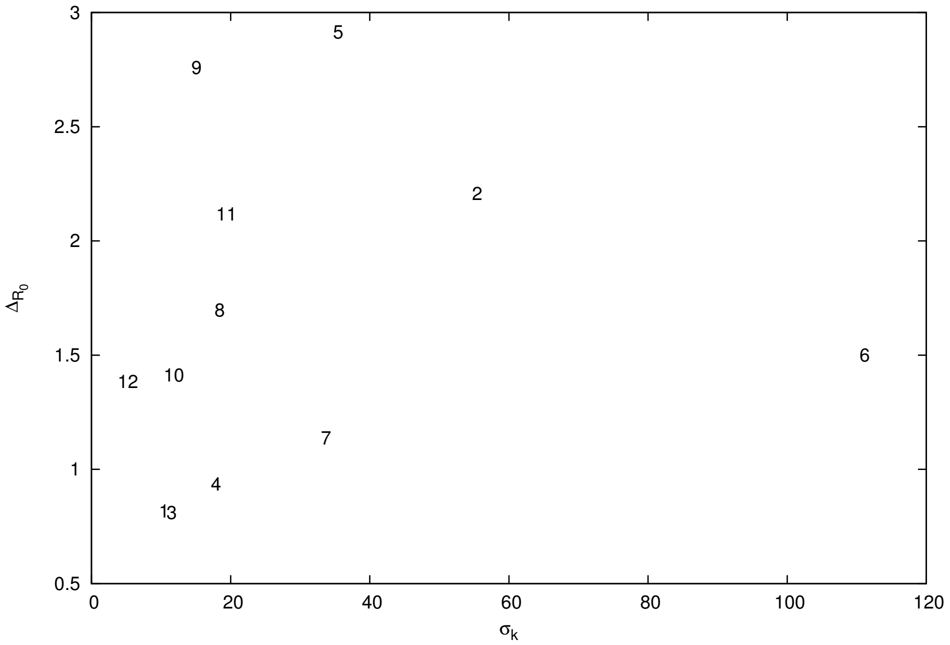

3
2.5
2
1.5
1
0.5
0
20
40
60
80
100
120
Δ_{R_0}
σ_k
5
9
2
11
8
6
12
10
7
4
13

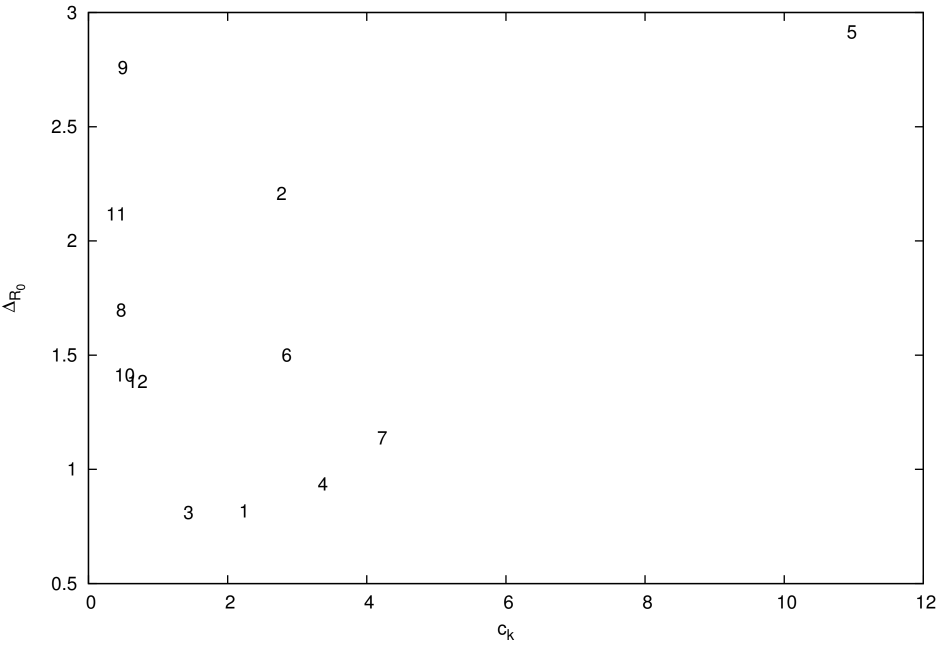

3
2.5
2
1.5
1
0.5
0
2
4
6
8
10
12
Δ_{R_0}
c_k
1
2
3
4
5
6
7
8
9
10
11
12

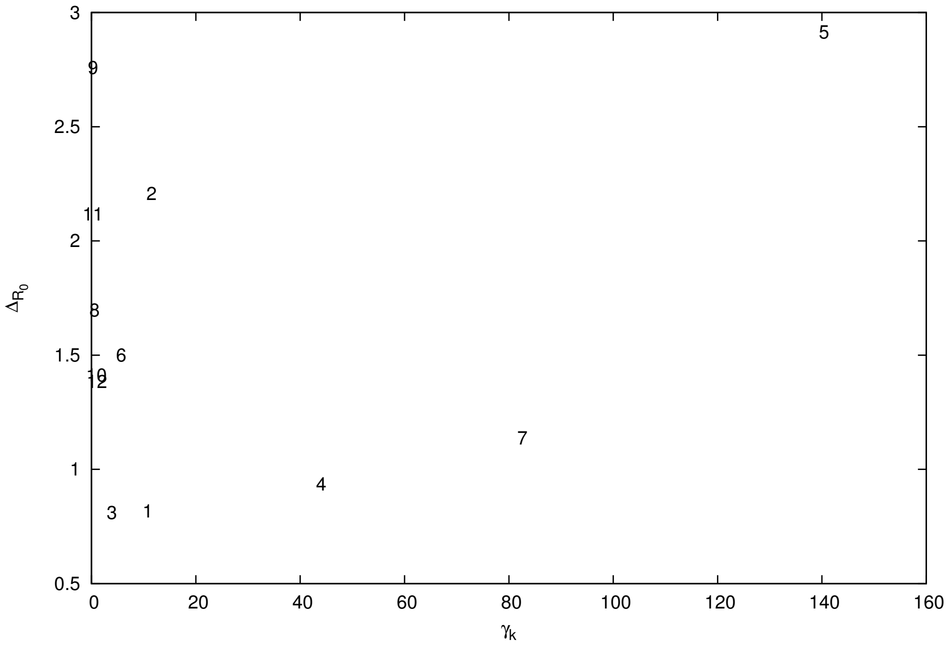

3
2.5
2
1.5
1
0.5
0
20
40
60
80
100
120
140
160
Δ_{R_0}
γ_k
1
2
3
4
5
6
7
8
9
10
11
12

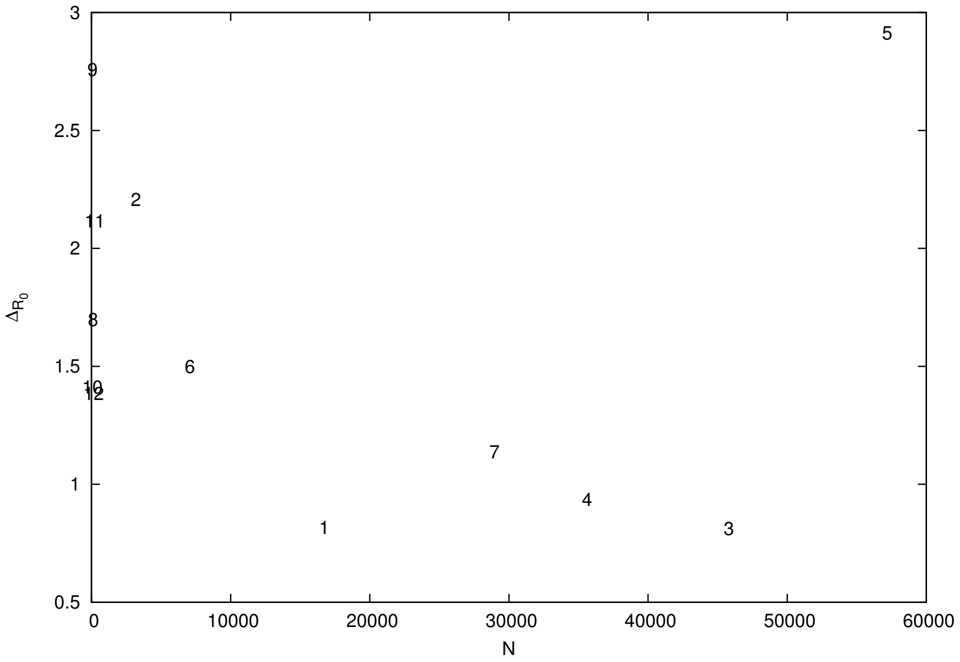

3
2.5
2
1.5
1
0.5
0
10000
20000
30000
40000
50000
60000
N
Δ_{R_0}
1
2
3
4
5
6
7
8
9
10
11
12

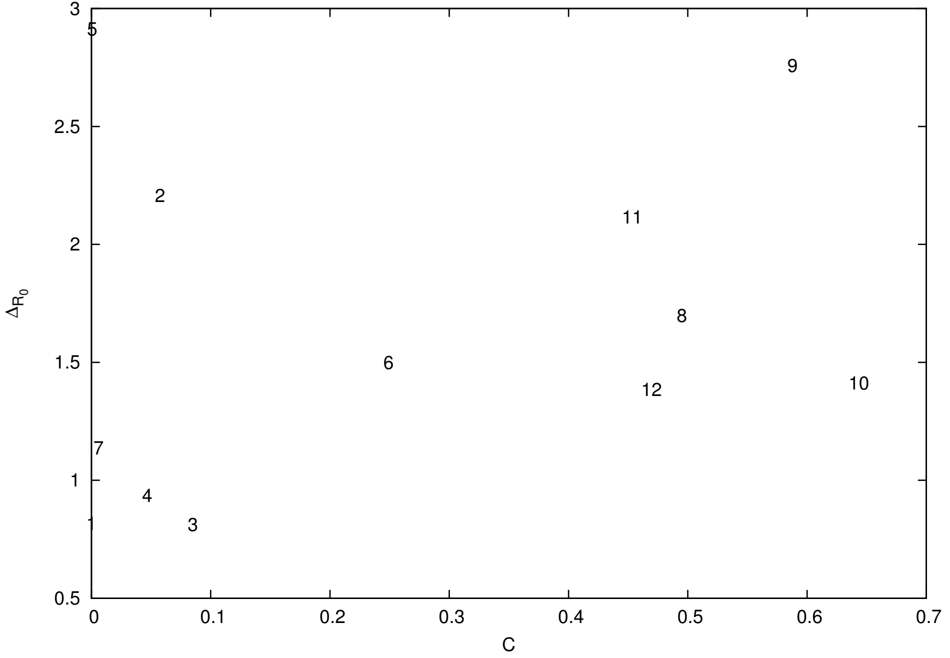

3
2.5
2
1.5
1
0.5
0
0.1
0.2
0.3
0.4
0.5
0.6
0.7
C
Δ_{R_0}
1
2
3
4
5
6
7
8
9
10
11
12

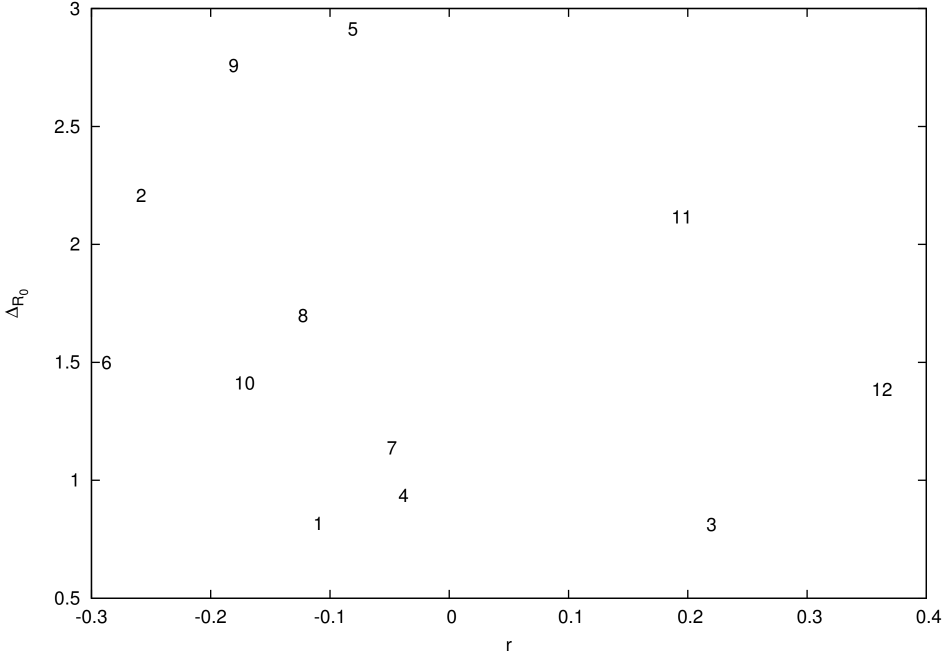

3
2.5
2
1.5
1
0.5
Δ_{R_0}
-0.3
-0.2
-0.1
0
0.1
0.2
0.3
0.4
r
5
9
2
11
8
6
10
12
7
4
1
3

ρΩ

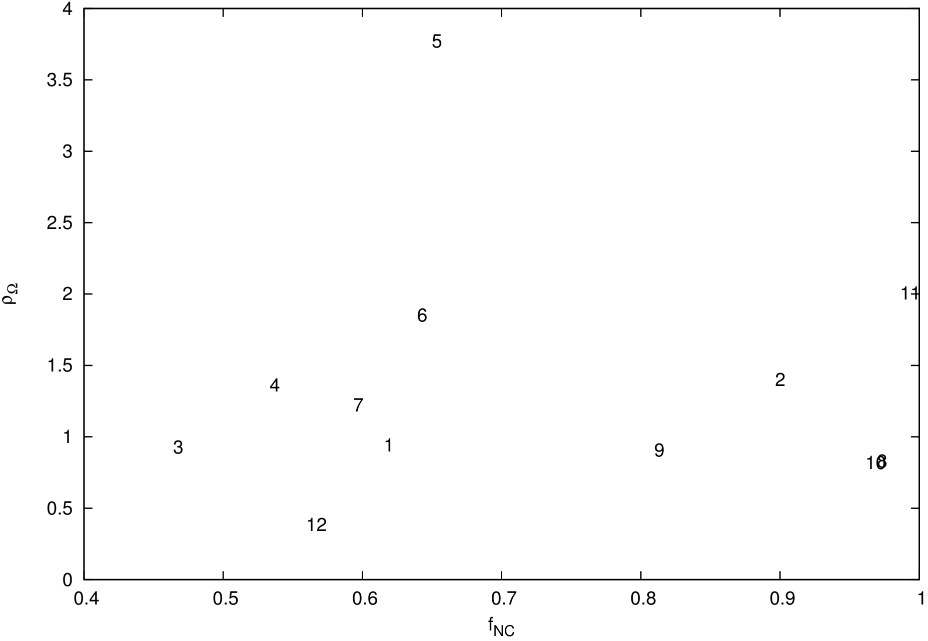

4
3.5
3
2.5
2
1.5
1
0.5
0
0.4
0.5
0.6
0.7
0.8
0.9
1
ρ_Ω
f_NC
5
6
11
2
4
7
3
1
9
10
8
12

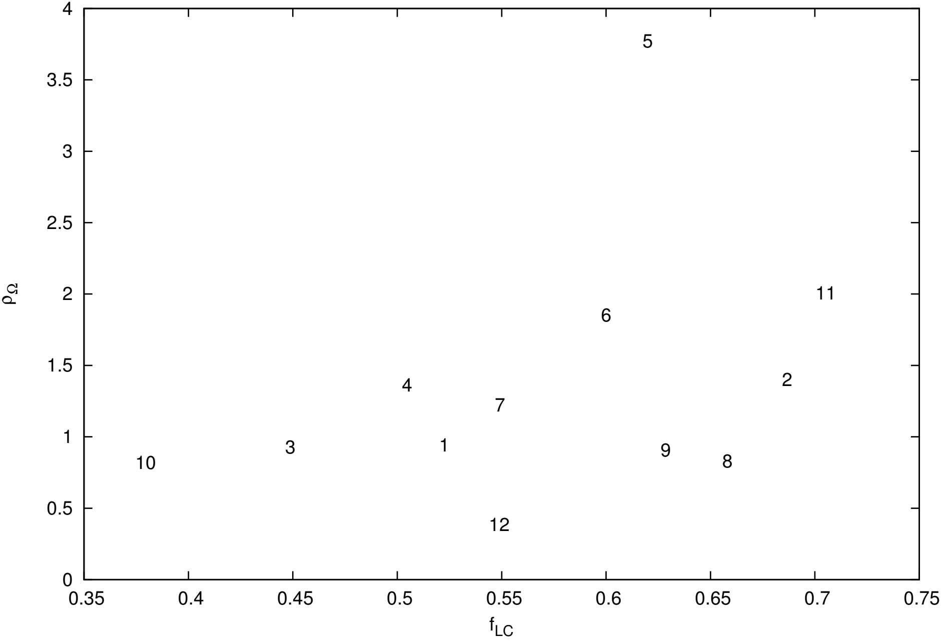

4
3.5
3
2.5
2
1.5
1
0.5
0
0.35
0.4
0.45
0.5
0.55
0.6
0.65
0.7
0.75
ρ_Ω
f_{LC}
5
11
6
2
4
7
1
3
9
8
10
12

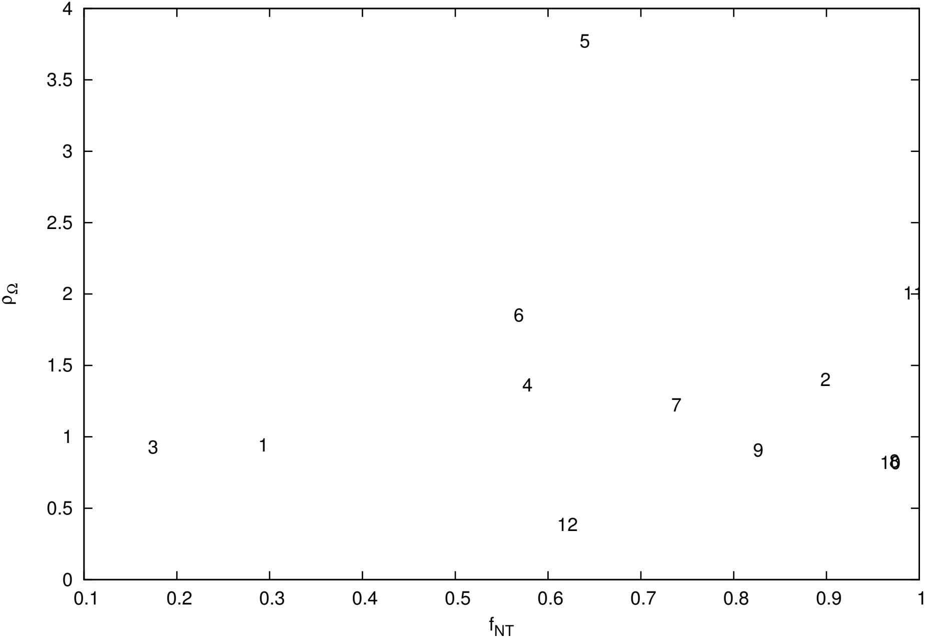

ρ_Ω
f_{NT}
0
0.5
1
1.5
2
2.5
3
3.5
4
0.1
0.2
0.3
0.4
0.5
0.6
0.7
0.8
0.9
1
5
6
4
7
2
3
1
9
12

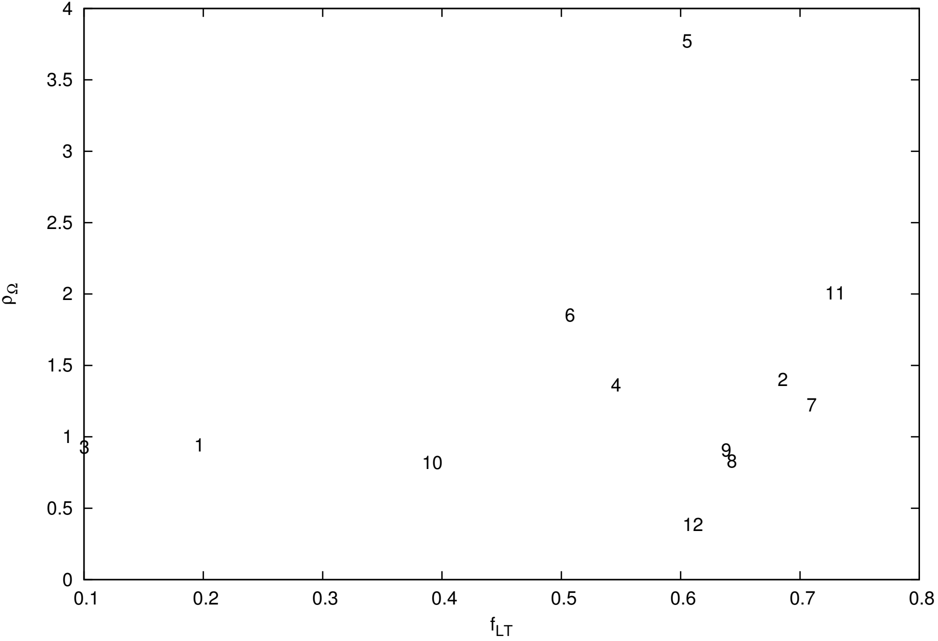

4
3.5
3
2.5
2
1.5
1
0.5
0
0.1
0.2
0.3
0.4
0.5
0.6
0.7
0.8
ρ_Ω
f_{LT}
5
11
6
2
4
7
3
1
9
10
8
12

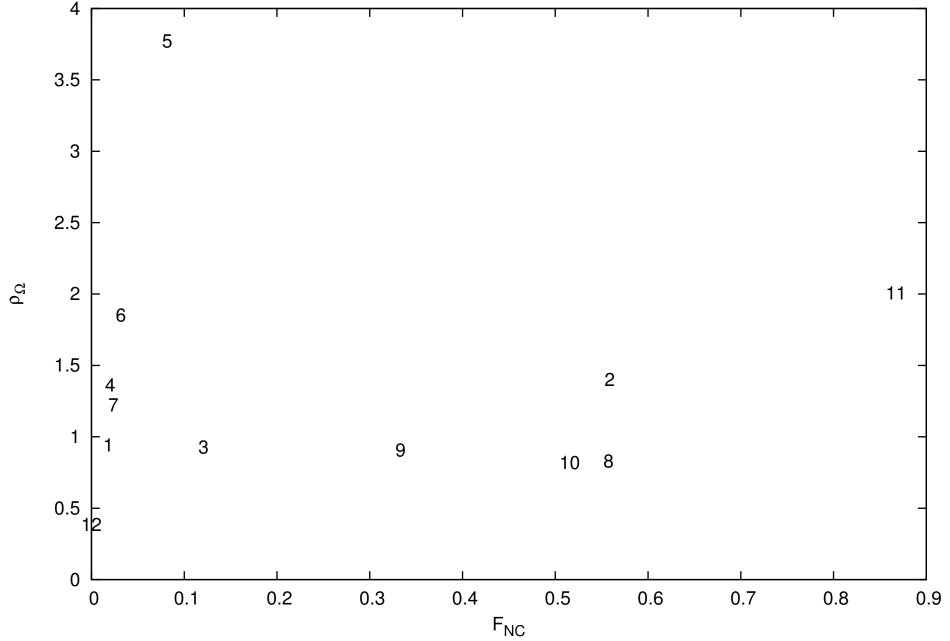

ρ_Ω
F_{NC}
0
0.5
1
1.5
2
2.5
3
3.5
4
0
0.1
0.2
0.3
0.4
0.5
0.6
0.7
0.8
0.9
1
2
3
4
5
6
7
8
9
10
11
12

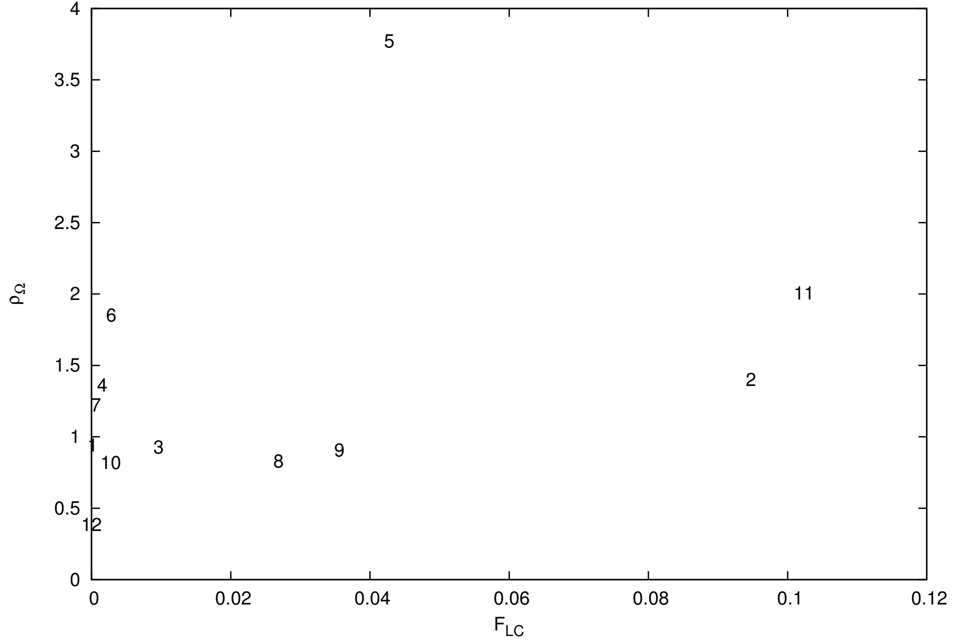

4
3.5
3
2.5
2
1.5
1
0.5
0
0
0.02
0.04
0.06
0.08
0.1
0.12
ρ_Ω
F_{LC}
5
11
6
2
4
7
1
3
9
10
8
12

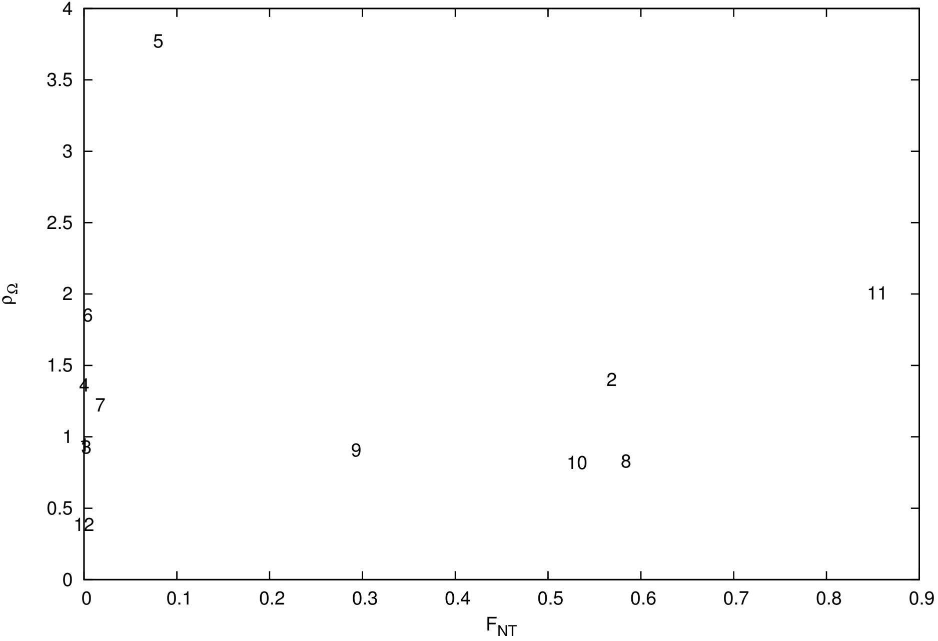

ρ_Ω
F_{NT}
0
0.5
1
1.5
2
2.5
3
3.5
4
0.1
0.2
0.3
0.4
0.5
0.6
0.7
0.8
0.9
5
6
4
7
3
12
9
10
8
2
11

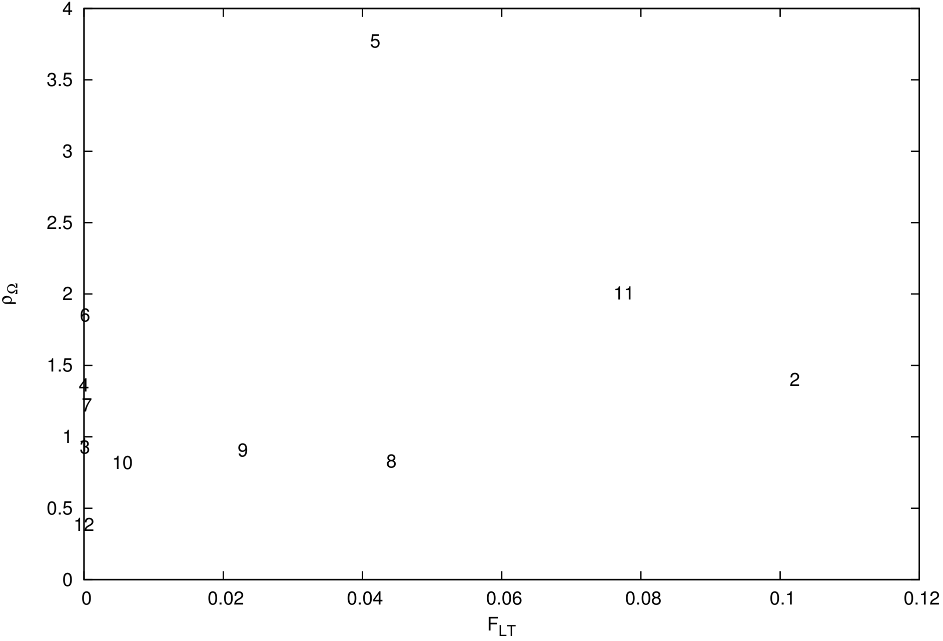

4
3.5
3
2.5
2
1.5
1
0.5
0
ρ_Ω
0
0.02
0.04
0.06
0.08
0.1
0.12
F_{LT}
5
11
6
2
4
7
3
9
10
8
12

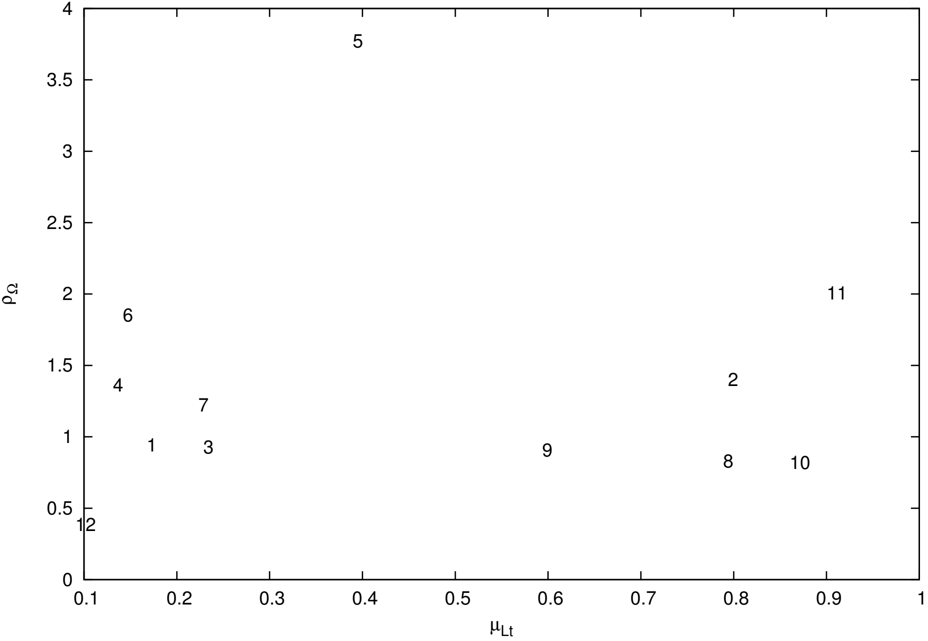

4
3.5
3
2.5
2
1.5
1
0.5
0
0.1
0.2
0.3
0.4
0.5
0.6
0.7
0.8
0.9
1
ρ_{Ω}
μ_{Lt}
5
11
6
2
4
7
1
3
9
8
10
12

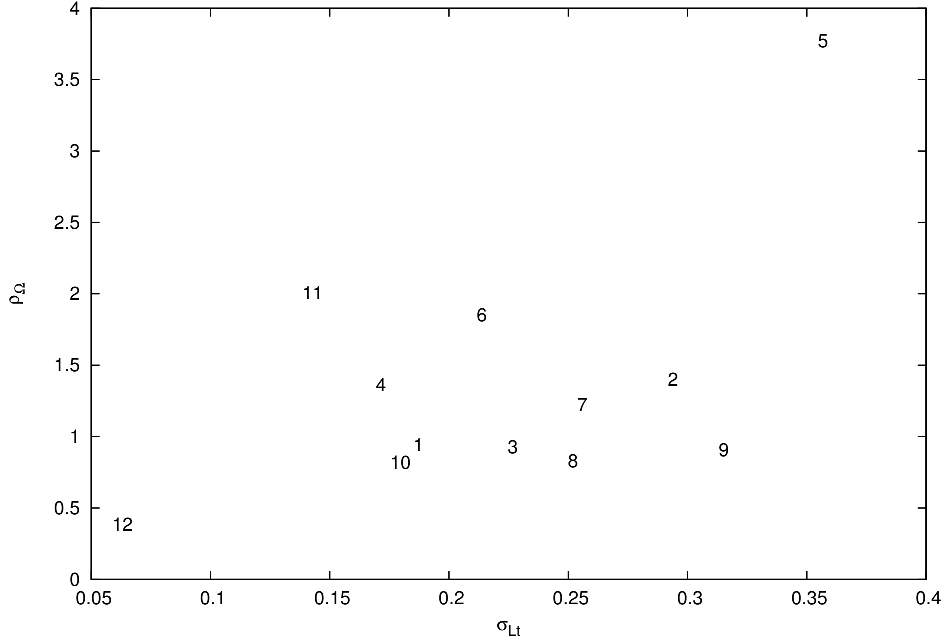

4
3.5
3
2.5
2
1.5
1
0.5
0
0.05
0.1
0.15
0.2
0.25
0.3
0.35
0.4
ρ_Ω
σ_{Lt}
5
11
6
4
2
7
1
3
9
10
8
12

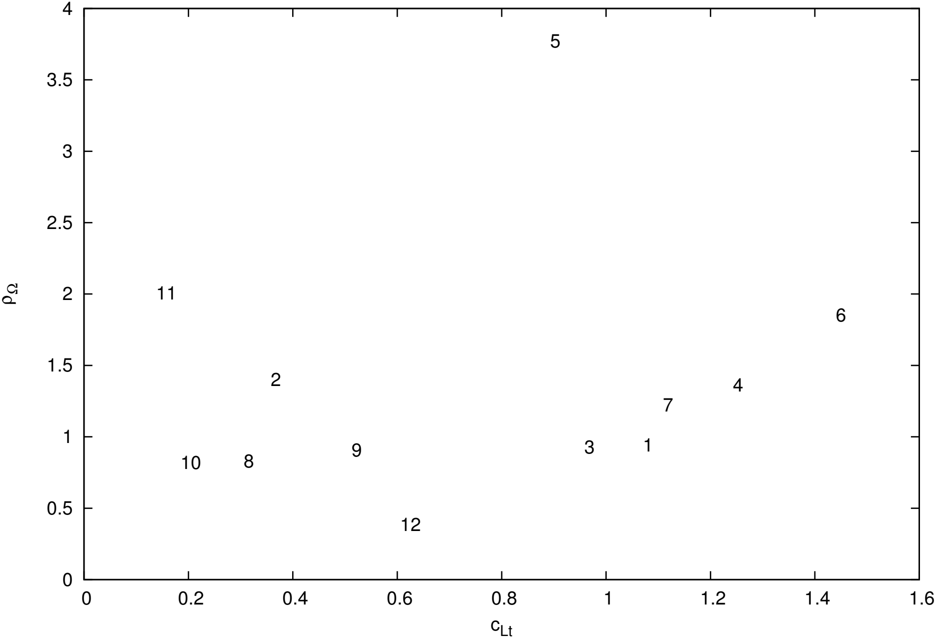

4
3.5
3
2.5
2
1.5
1
0.5
0
0
0.2
0.4
0.6
0.8
1
1.2
1.4
1.6
ρ_Ω
c_Lt
5
11
6
2
4
7
3
1
9
10
8
12

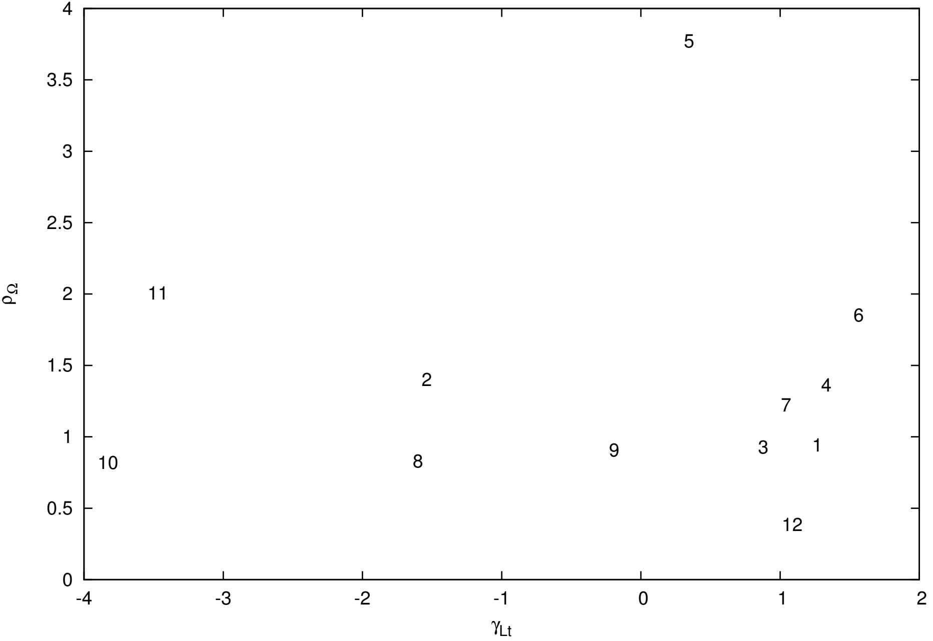

4
3.5
3
2.5
2
1.5
1
0.5
0
-4
-3
-2
-1
0
1
2
ρΩ
γLt
5
11
6
2
4
7
9
3
1
10
8
12

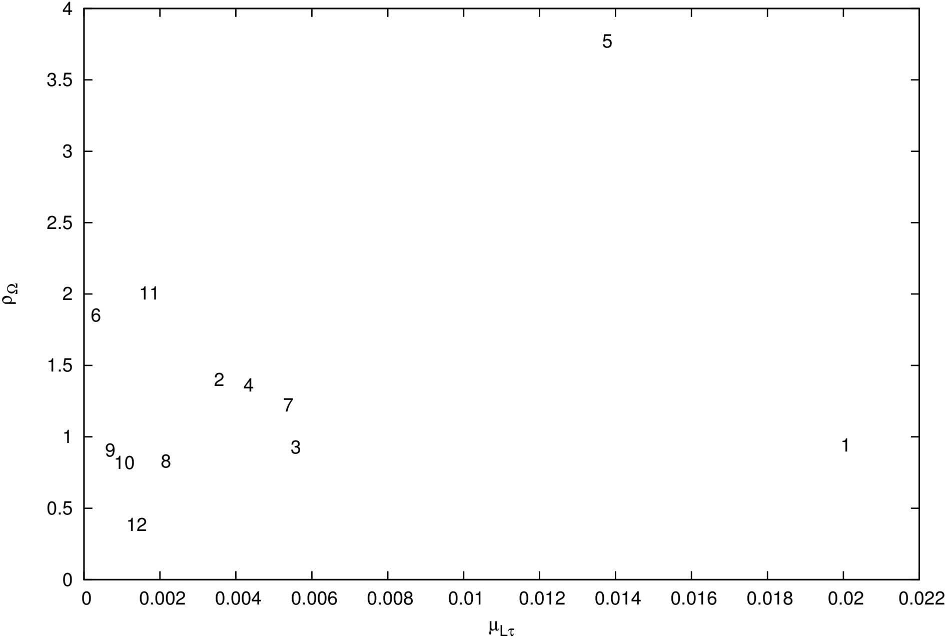

4
3.5
3
2.5
2
1.5
1
0.5
0
0
0.002
0.004
0.006
0.008
0.01
0.012
0.014
0.016
0.018
0.02
0.022
ρ_Ω
$\mu_{L\tau}$
5
11
6
2
4
7
3
1
9
10
8
12

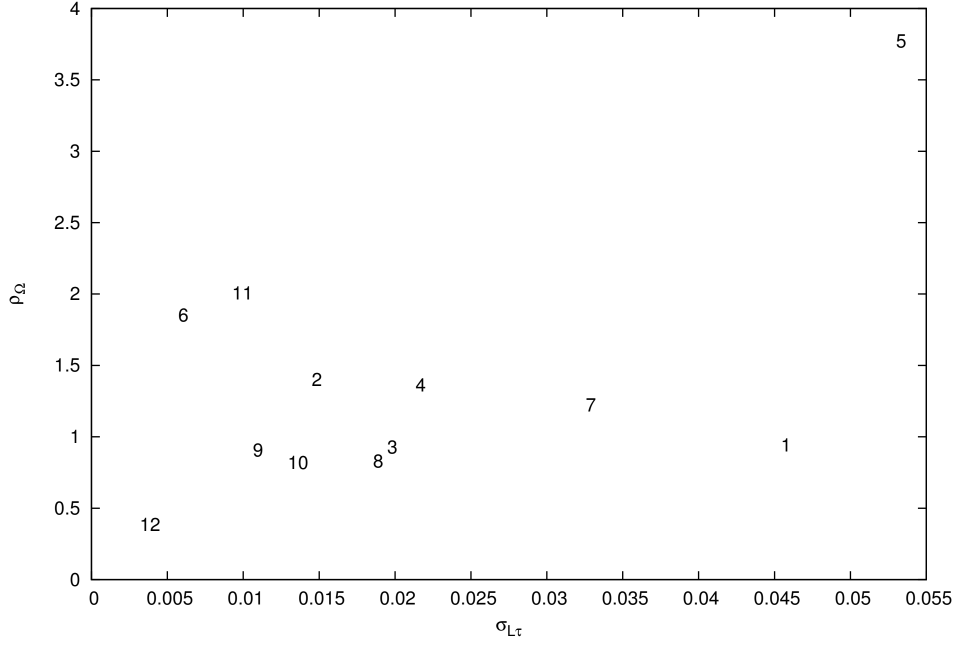

ρ_Ω
$\sigma_{L\tau}$
0
0.5
1
1.5
2
2.5
3
3.5
4
0
0.005
0.01
0.015
0.02
0.025
0.03
0.035
0.04
0.045
0.05
0.055
1
2
3
4
5
6
7
8
9
10
11
12

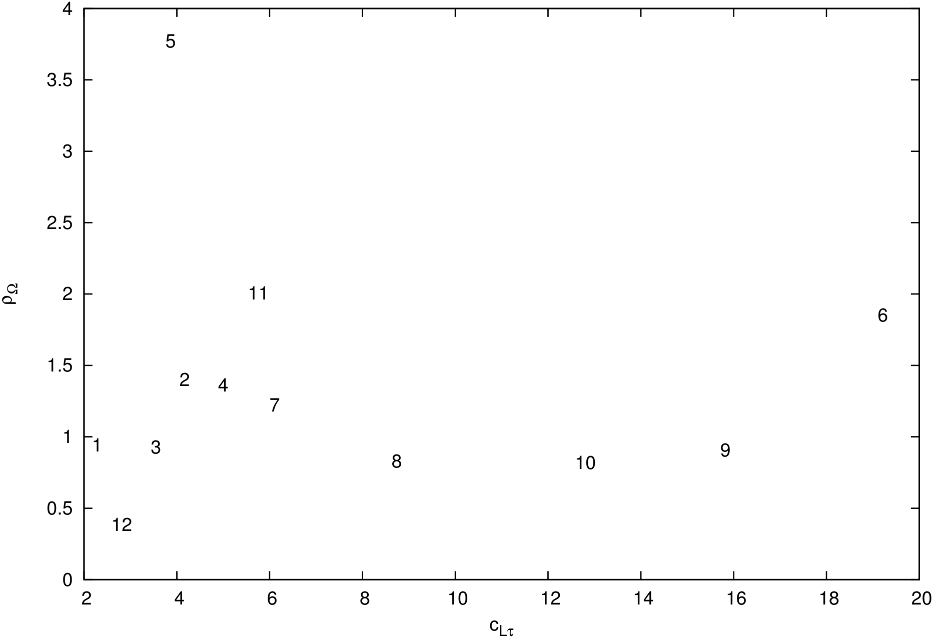

ρ_Ω
c_Lτ
0
0.5
1
1.5
2
2.5
3
3.5
4
2
4
6
8
10
12
14
16
18
20
1
2
3
4
5
6
7
8
9
10
11
12

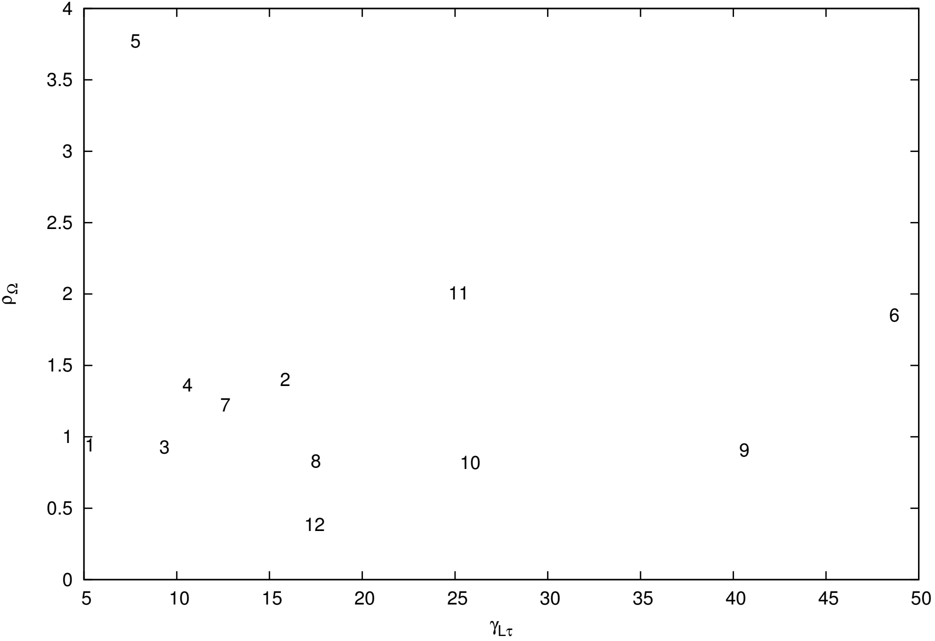

4
3.5
3
2.5
2
1.5
1
0.5
0
5
10
15
20
25
30
35
40
45
50
ρ_Ω
$\gamma_{L\tau}$
5
11
6
2
4
7
1
3
8
10
9
12

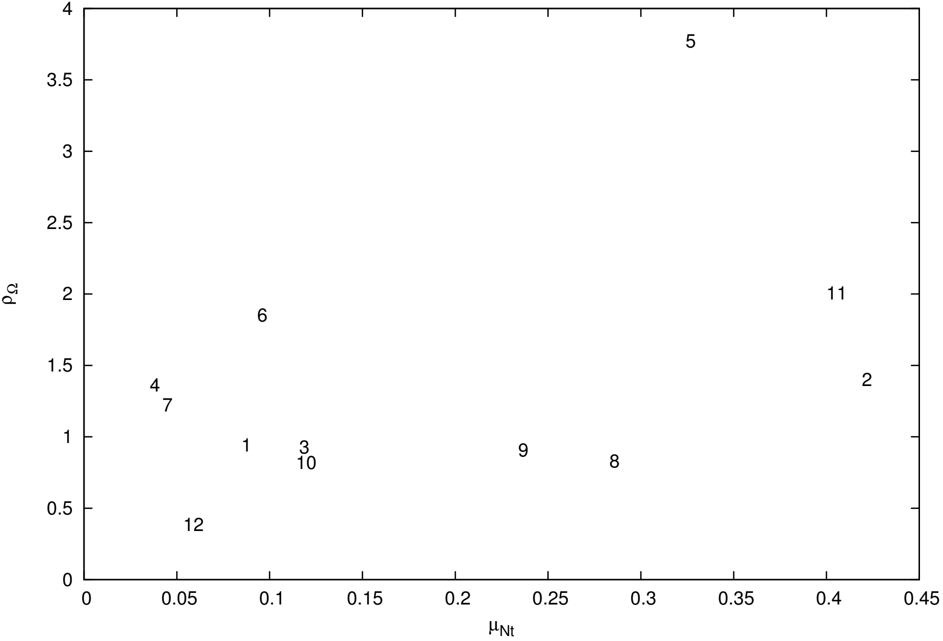

4
3.5
3
2.5
2
1.5
1
0.5
0
0
0.05
0.1
0.15
0.2
0.25
0.3
0.35
0.4
0.45
ρΩ
μNt
1
2
3
4
5
6
7
8
9
10
11
12

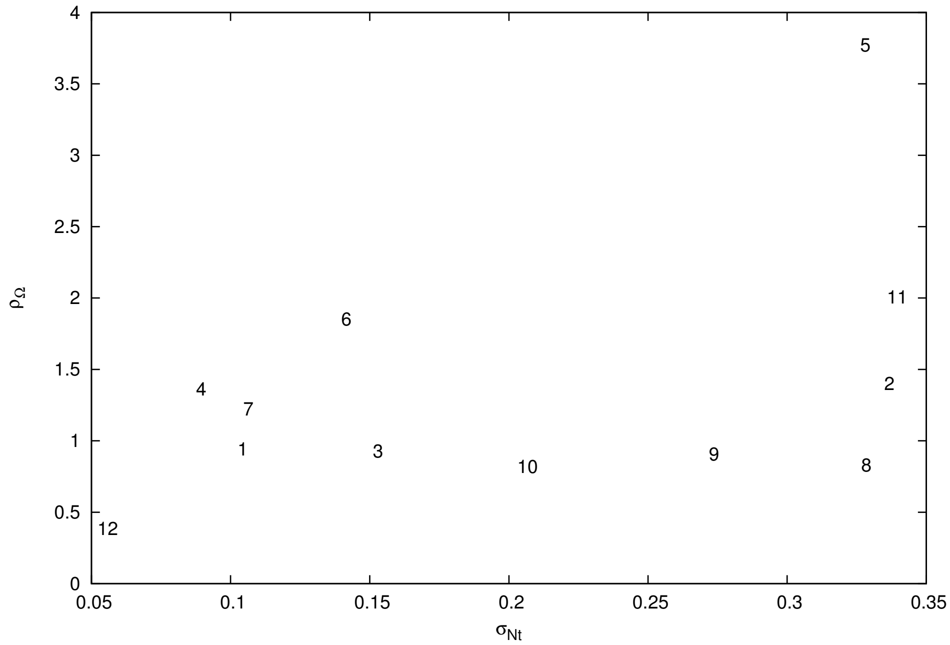

ρ_{Ω}
σ_{Nt}
0
0.5
1
1.5
2
2.5
3
3.5
4
0.05
0.1
0.15
0.2
0.25
0.3
0.35
1
2
3
4
5
6
7
8
9
10
11
12

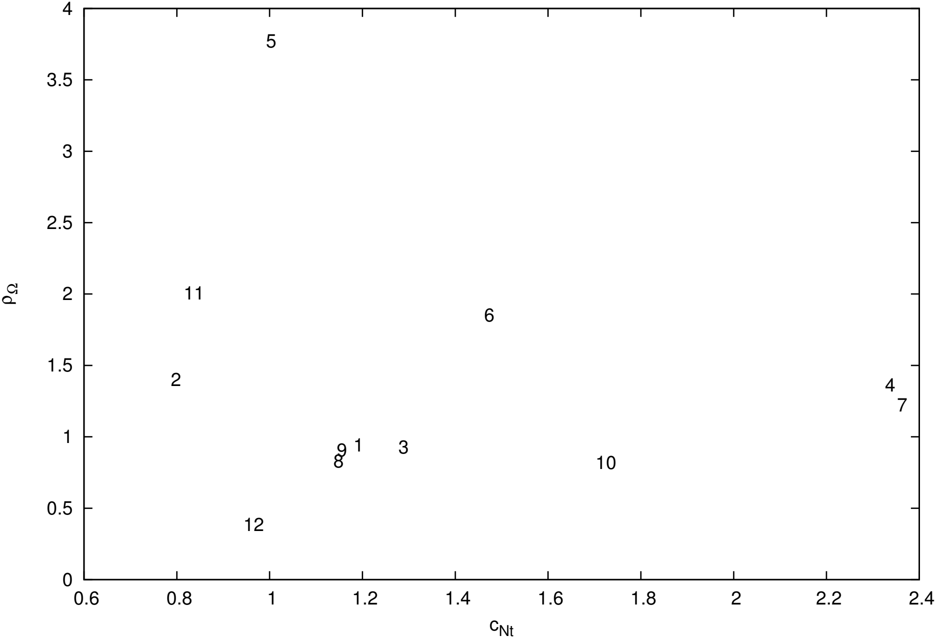

4
3.5
3
2.5
2
1.5
1
0.5
0
0.6
0.8
1
1.2
1.4
1.6
1.8
2
2.2
2.4
ρ_Ω
c_{Nt}
5
11
6
2
4
7
9
1
3
8
10
12

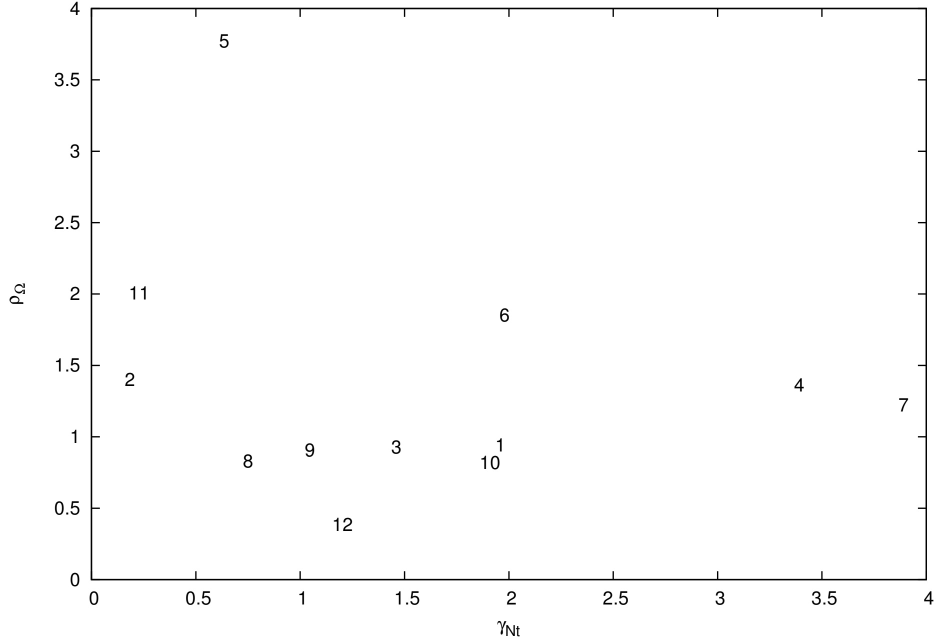

ρ_Ω
γ_{Nt}
0
0.5
1
1.5
2
2.5
3
3.5
4
5
11
6
2
4
7
9
3
1
8
10
12

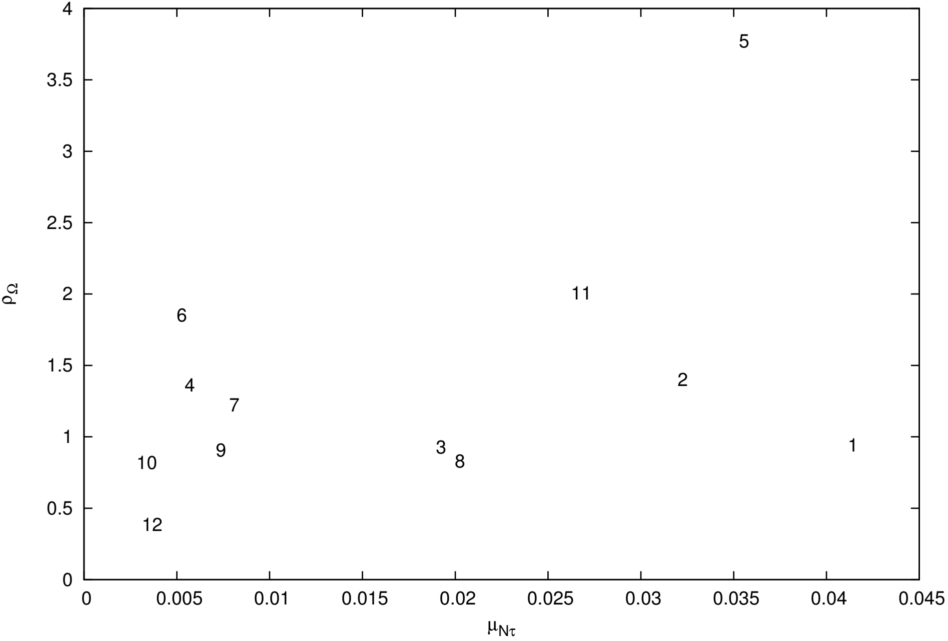

ρ_Ω
$\mu_{N\tau}$
0
0.5
1
1.5
2
2.5
3
3.5
4
0
0.005
0.01
0.015
0.02
0.025
0.03
0.035
0.04
0.045
1
2
3
4
5
6
7
8
9
10
11
12

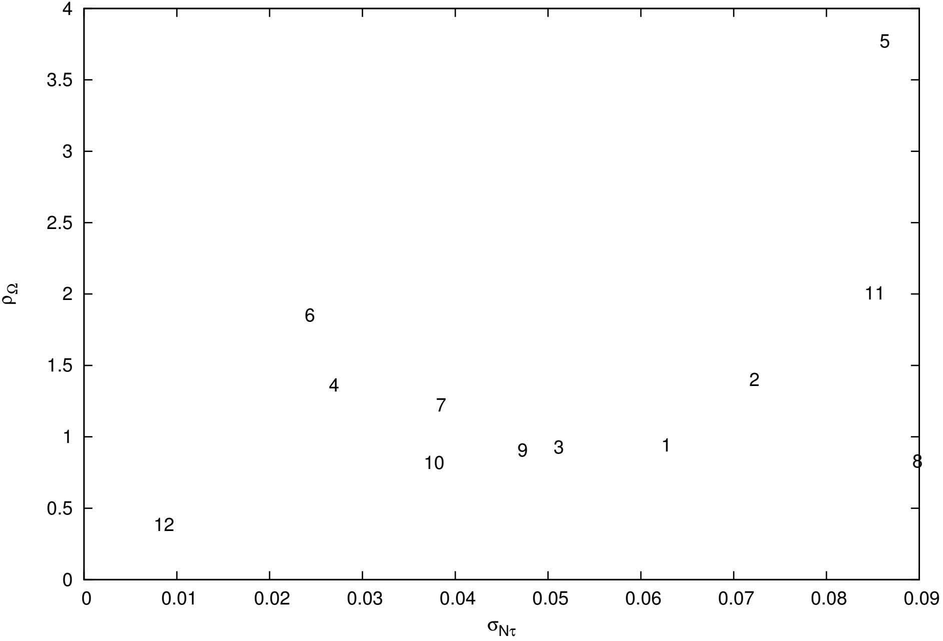

4
3.5
3
2.5
2
1.5
1
0.5
0
ρ_Ω
0
0.01
0.02
0.03
0.04
0.05
0.06
0.07
0.08
0.09
$\sigma_{N\tau}$
5
11
6
4
2
7
9
3
1
10
8
12

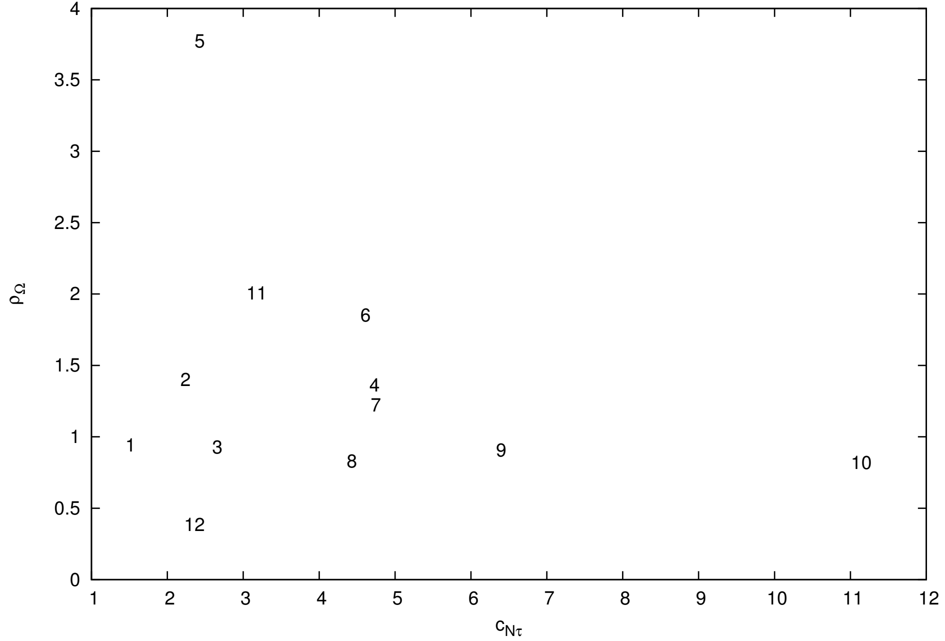

ρ_Ω
$c_{N\tau}$
0
0.5
1
1.5
2
2.5
3
3.5
4
1
2
3
4
5
6
7
8
9
10
11
12
5
11
6
2
4
7
1
3
9
8
10
12

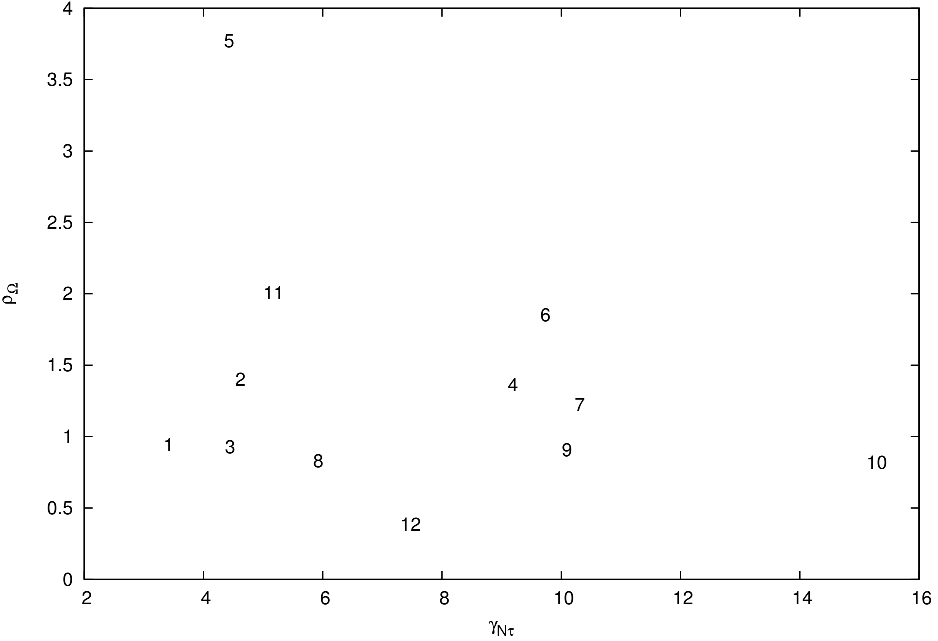

4
3.5
3
2.5
2
1.5
1
0.5
0
2
4
6
8
10
12
14
16
ρ_Ω
γ_Nτ
5
11
6
2
4
7
1
3
9
8
10
12

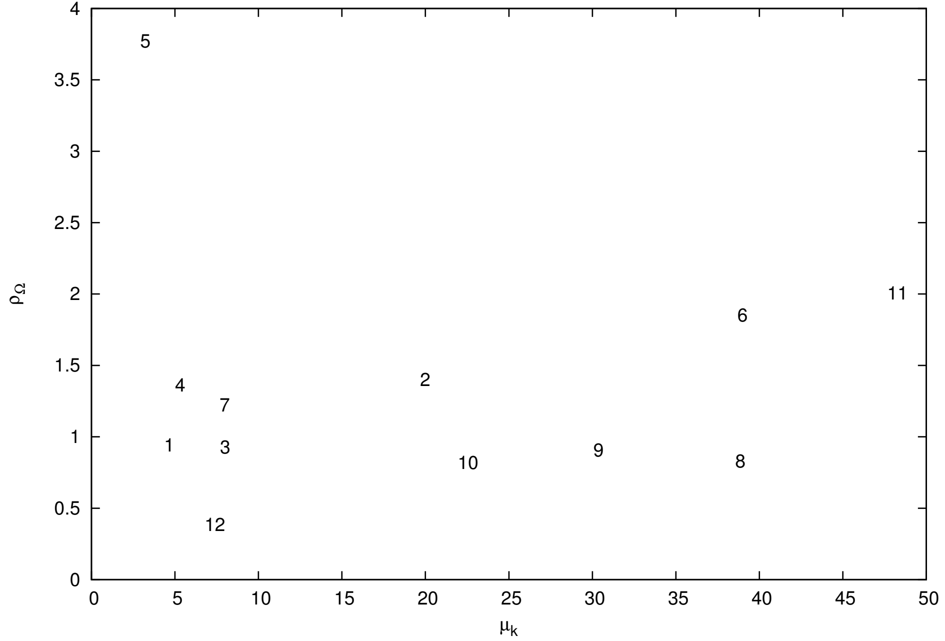

ρ_Ω
μ_k
0
0.5
1
1.5
2
2.5
3
3.5
4
0
5
10
15
20
25
30
35
40
45
50
5
11
6
2
4
7
1
3
10
9
8
12

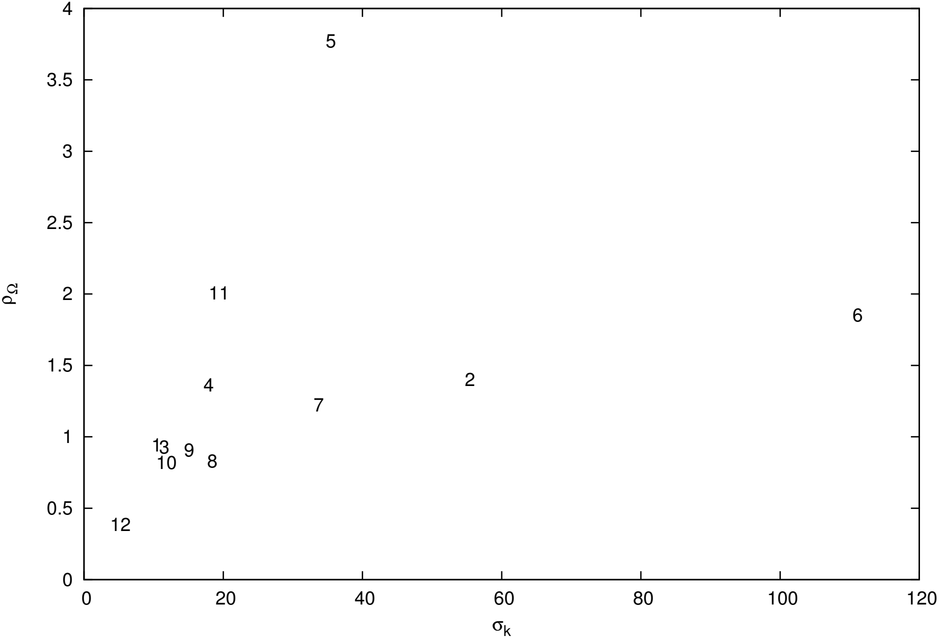

ρ_Ω
σ_k
0
0.5
1
1.5
2
2.5
3
3.5
4
0
20
40
60
80
100
120
5
11
6
2
4
7
13
9
10
8
12

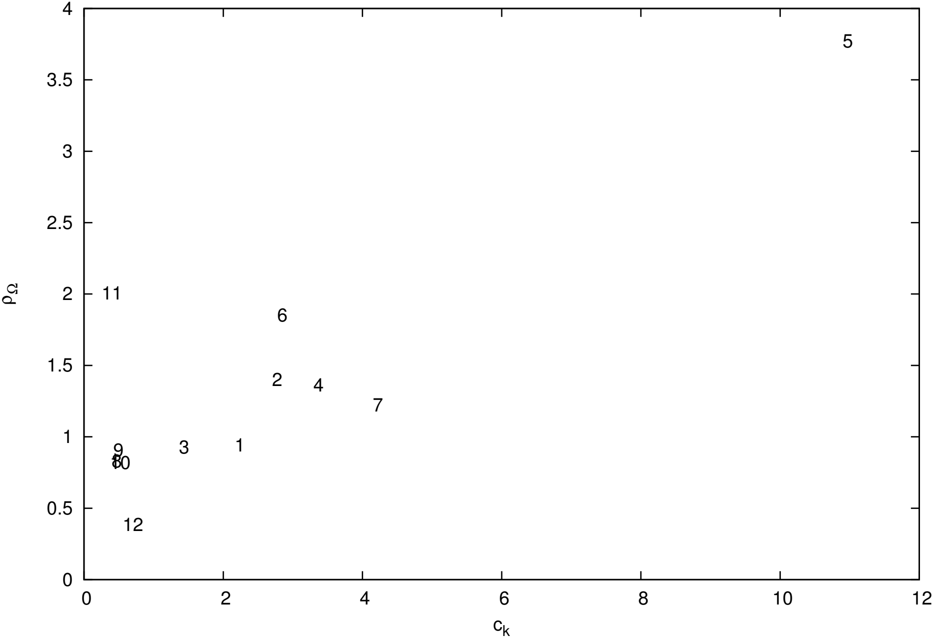

4
3.5
3
2.5
2
1.5
1
0.5
0
0
2
4
6
8
10
12
ρ_Ω
c_k
5
11
6
2
4
7
9
3
1
8
10
12

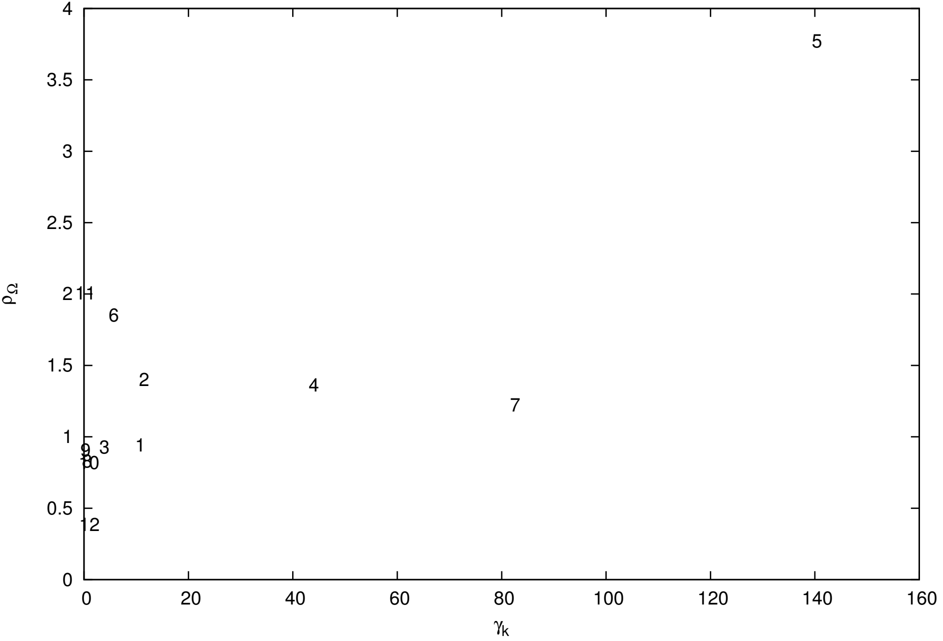

ρ_Ω
γ_k
0
0.5
1
1.5
2
2.5
3
3.5
4
0
20
40
60
80
100
120
140
160
5
11
6
2
4
7
1
3
9
8
10
12

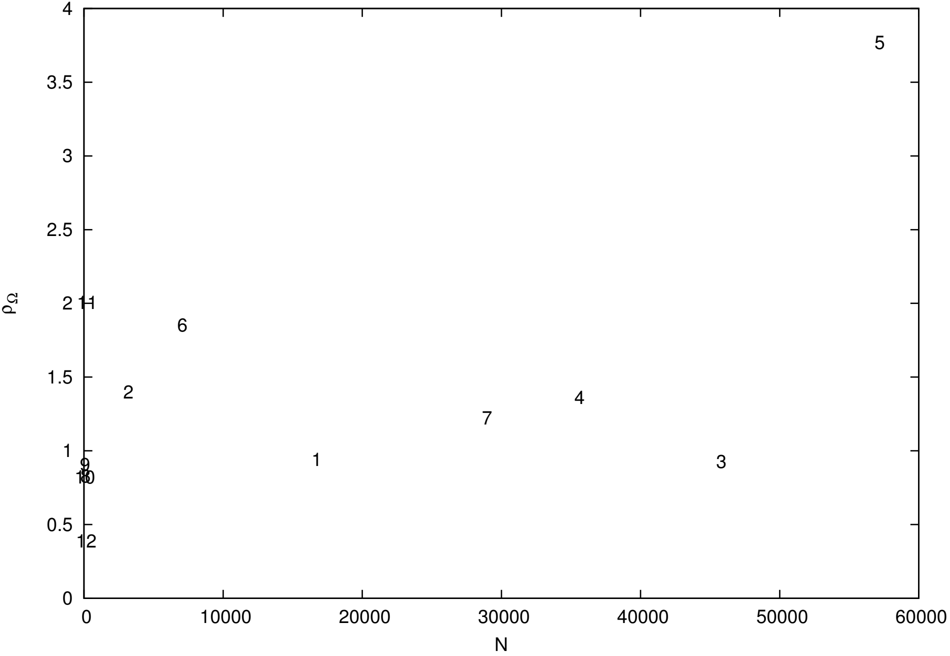

ρ_Ω
N
0
0.5
1
1.5
2
2.5
3
3.5
4
0
10000
20000
30000
40000
50000
60000
1
2
3
4
5
6
7
9
10
11
12

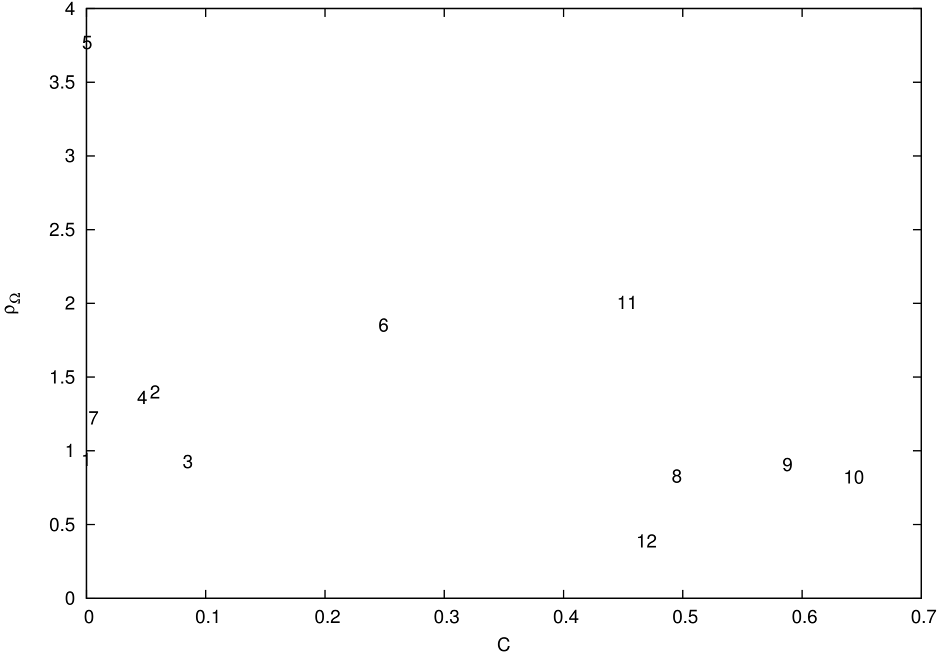

4
3.5
3
2.5
2
1.5
1
0.5
0
0
0.1
0.2
0.3
0.4
0.5
0.6
0.7
ρ_Ω
C
5
11
6
4
2
7
1
3
9
8
10
12

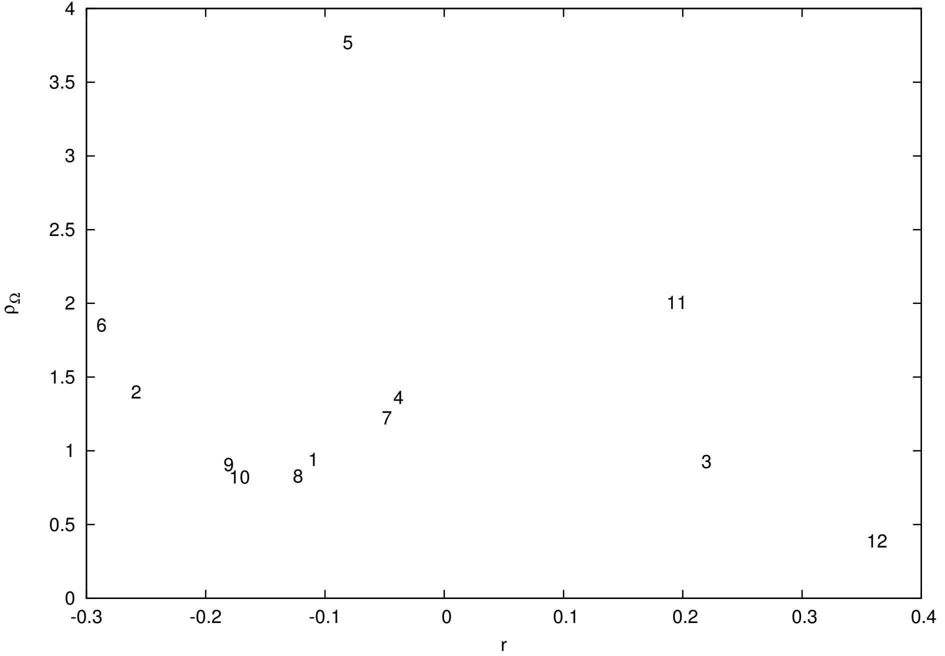

4
3.5
3
2.5
2
1.5
1
0.5
0
-0.3
-0.2
-0.1
0
0.1
0.2
0.3
0.4
r
ρ_Ω
5
11
6
2
4
7
1
9
10
8
3
12

ΔΩ

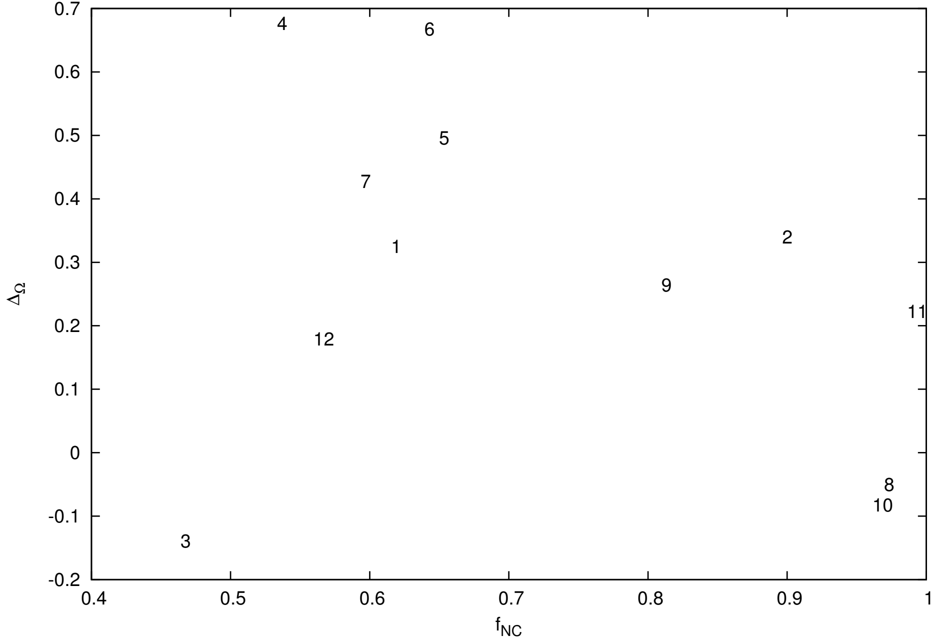

0.7
0.6
0.5
0.4
0.3
0.2
0.1
0
-0.1
-0.2
0.4
0.5
0.6
0.7
0.8
0.9
1
Δ_Ω
f_{NC}
4
6
5
7
1
2
9
11
12
8
10
3

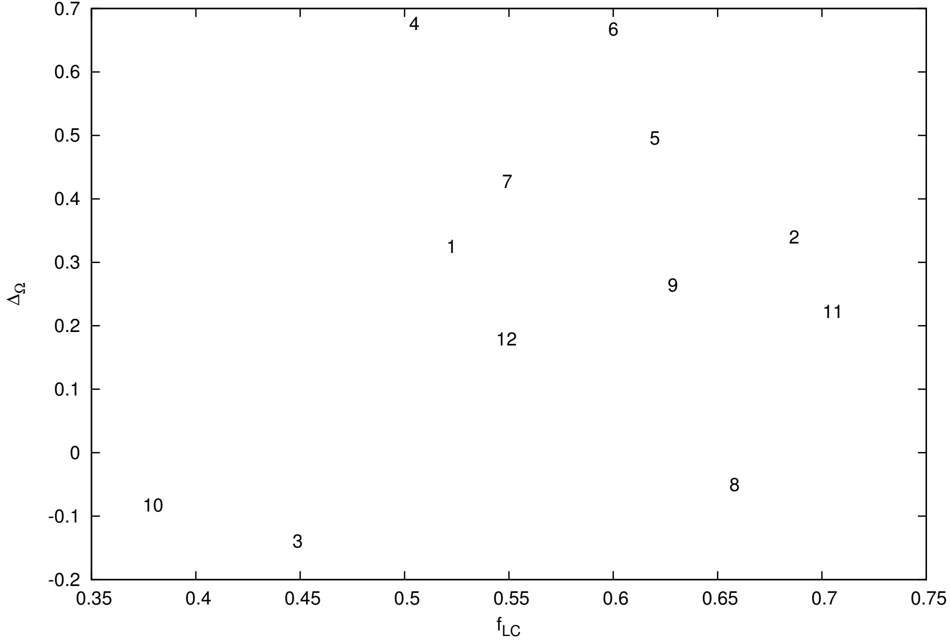

Δ_Ω
f_{LC}
0.7
0.6
0.5
0.4
0.3
0.2
0.1
0
-0.1
-0.2
0.35
0.4
0.45
0.5
0.55
0.6
0.65
0.7
0.75
1
2
3
4
5
6
7
8
9
10
11
12

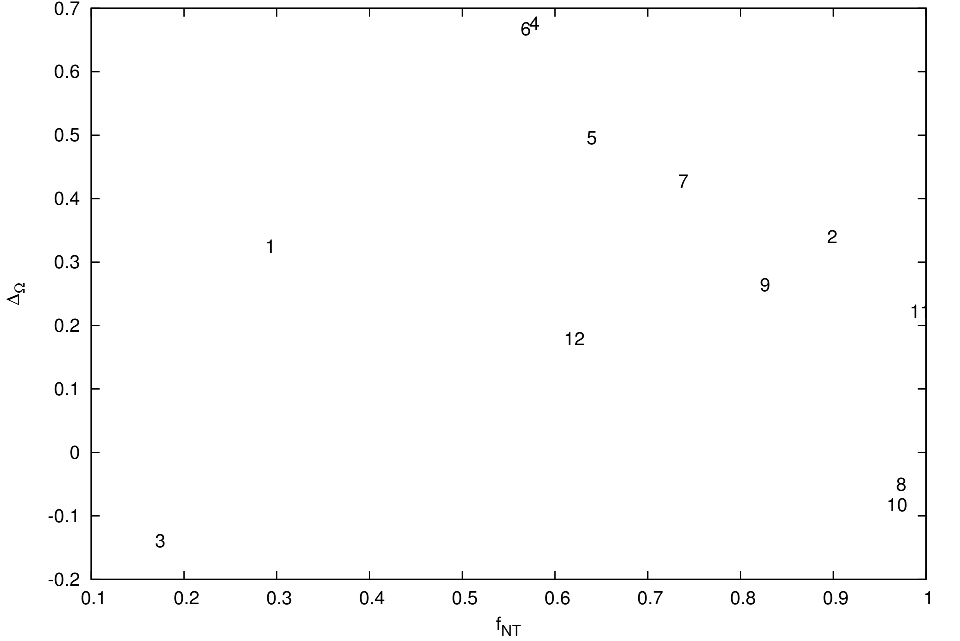

0.7
0.6
0.5
0.4
0.3
0.2
0.1
0
-0.1
-0.2
Δ_Ω
0.1
0.2
0.3
0.4
0.5
0.6
0.7
0.8
0.9
1
f_{NT}
1
2
3
4
5
6
7
8
9
10
11
12

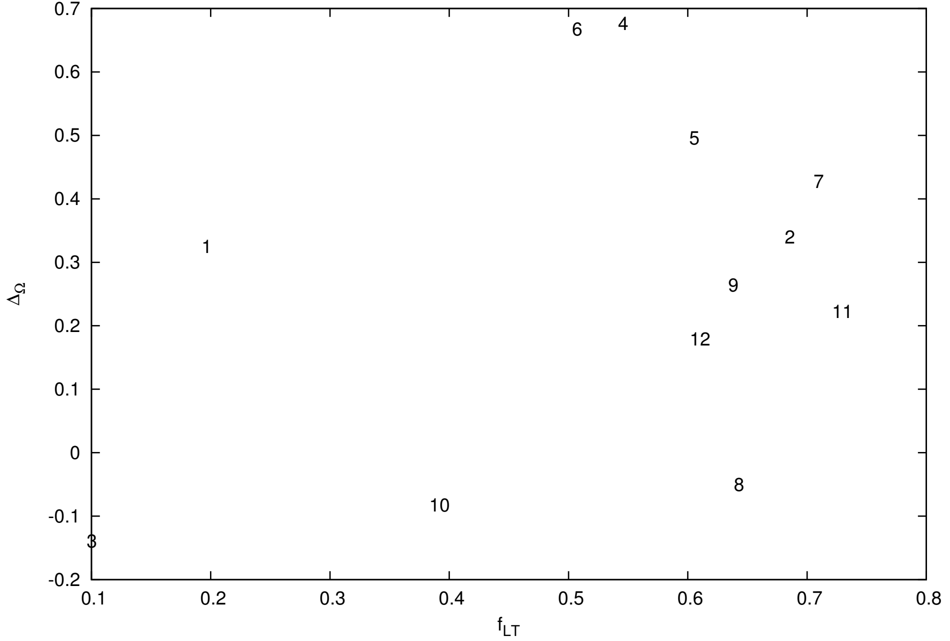
0.7
0.6
0.5
0.4
0.3
0.2
0.1
0
-0.1
-0.2
Δ_Ω
0.1
0.2
0.3
0.4
0.5
0.6
0.7
0.8
f_{LT}
1
2
3
4
5
6
7
8
9
10
11
12

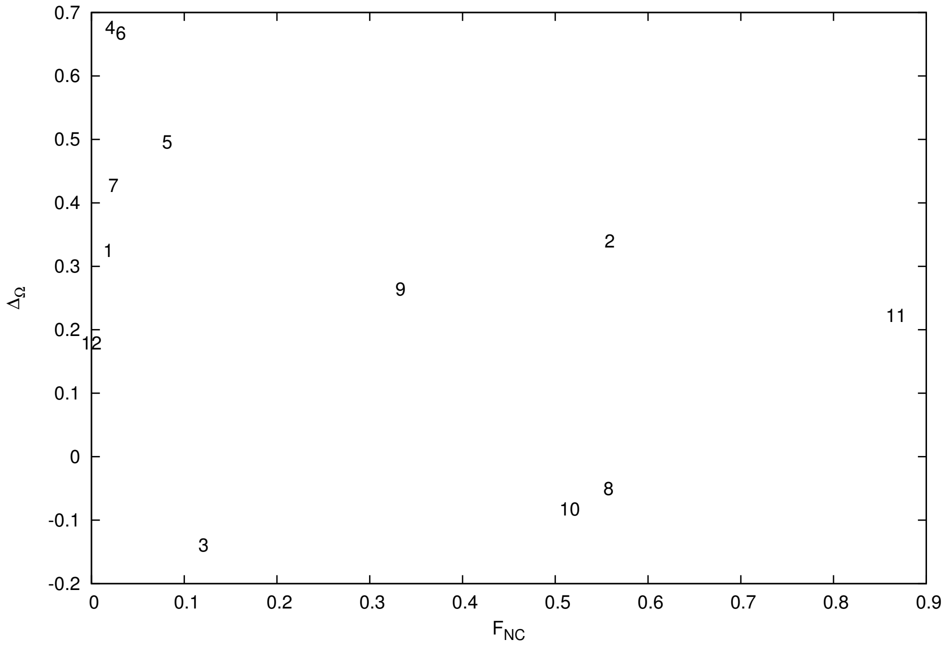

0.7
0.6
0.5
0.4
0.3
0.2
0.1
0
-0.1
-0.2
0
0.1
0.2
0.3
0.4
0.5
0.6
0.7
0.8
0.9
Δ_Ω
F_{NC}
4
6
5
7
1
2
9
11
12
8
10
3

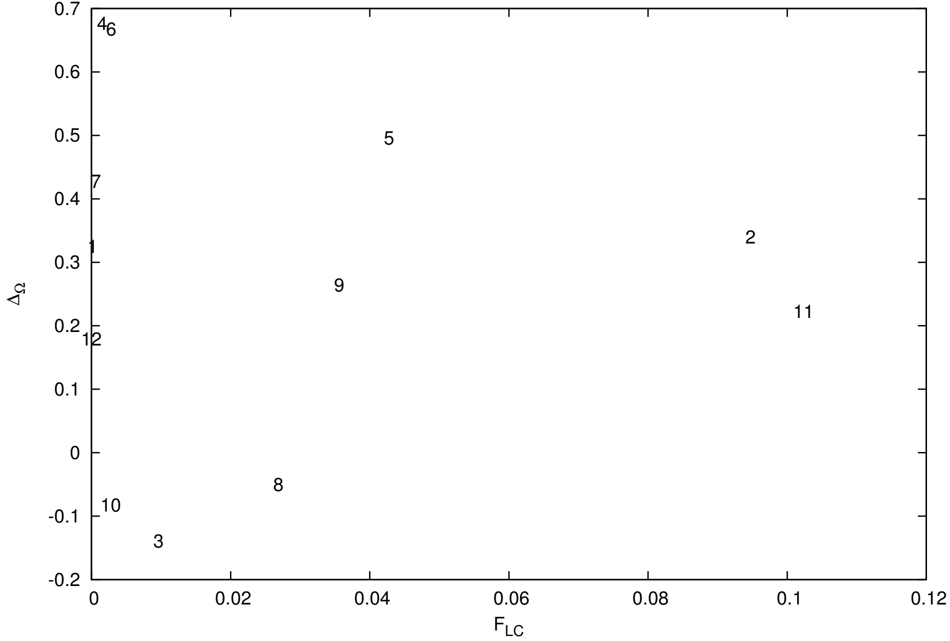

0.7
0.6
0.5
0.4
0.3
0.2
0.1
0
-0.1
-0.2
Δ_Ω
0
0.02
0.04
0.06
0.08
0.1
0.12
F_{LC}
4
6
5
7
1
2
9
11
12
8
10
3

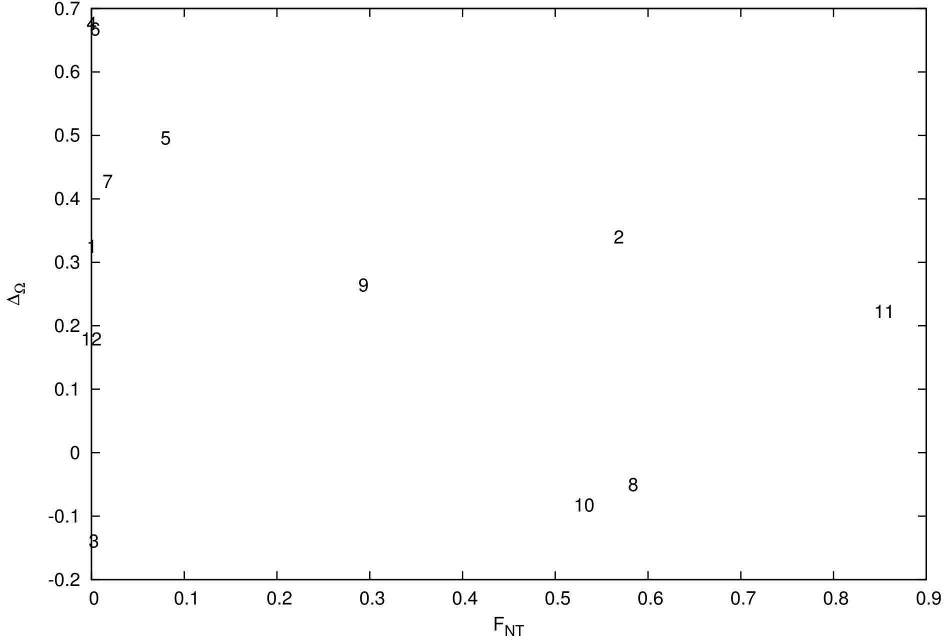

0.7
0.6
0.5
0.4
0.3
0.2
0.1
0
-0.1
-0.2
Δ_Ω
0
0.1
0.2
0.3
0.4
0.5
0.6
0.7
0.8
0.9
F_{NT}
4
6
5
7
1
2
9
11
12
8
10
3

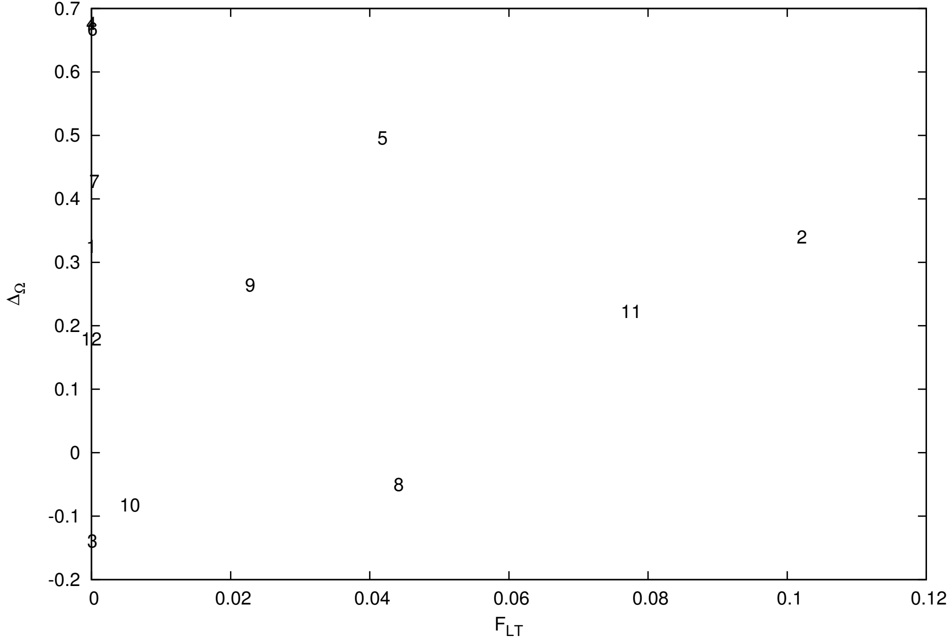

0.7
0.6
0.5
0.4
0.3
0.2
0.1
0
-0.1
-0.2
0
0.02
0.04
0.06
0.08
0.1
0.12
Δ_Ω
F_{LT}
4
6
5
7
1
2
9
11
12
8
10
3

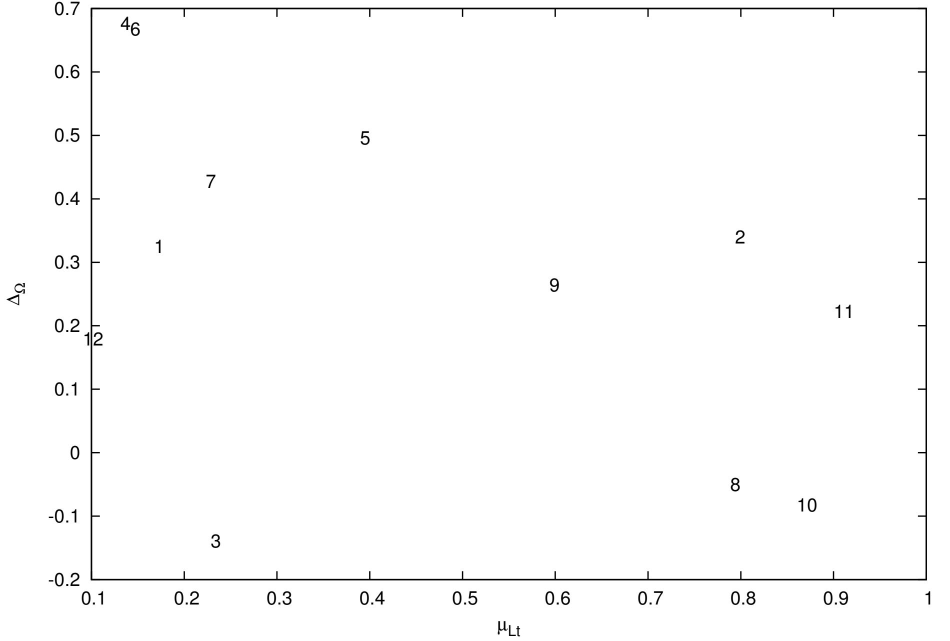

0.7
0.6
0.5
0.4
0.3
0.2
0.1
0
-0.1
-0.2
0.1
0.2
0.3
0.4
0.5
0.6
0.7
0.8
0.9
1
Δ_Ω
μ_{Lt}
4
6
5
7
1
2
9
11
12
8
10
3

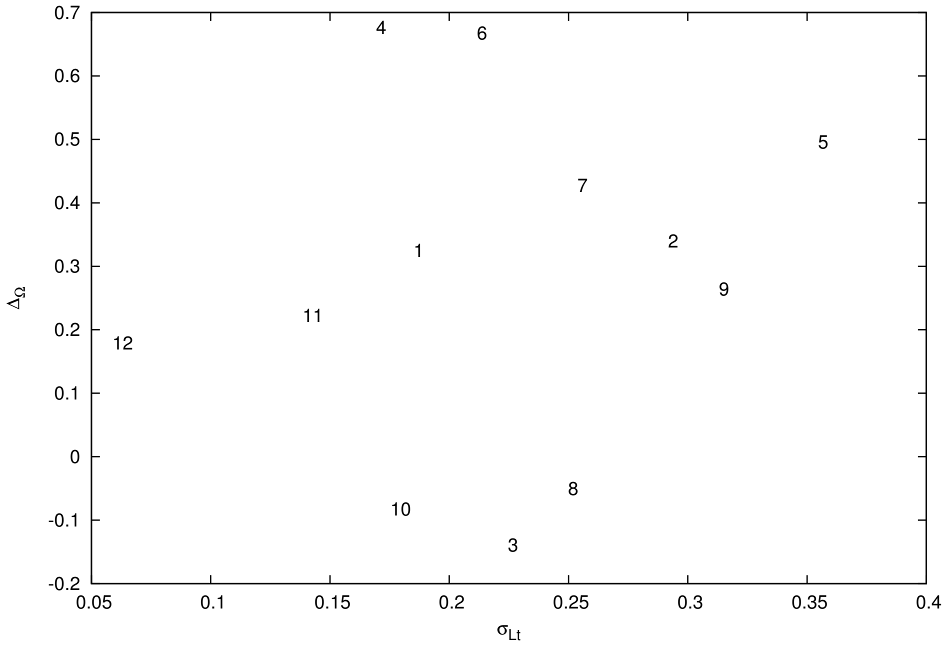

0.7
0.6
0.5
0.4
0.3
0.2
0.1
0
-0.1
-0.2
0.05
0.1
0.15
0.2
0.25
0.3
0.35
0.4
Δ_Ω
σ_{Lt}
1
2
3
4
5
6
7
8
9
10
11
12

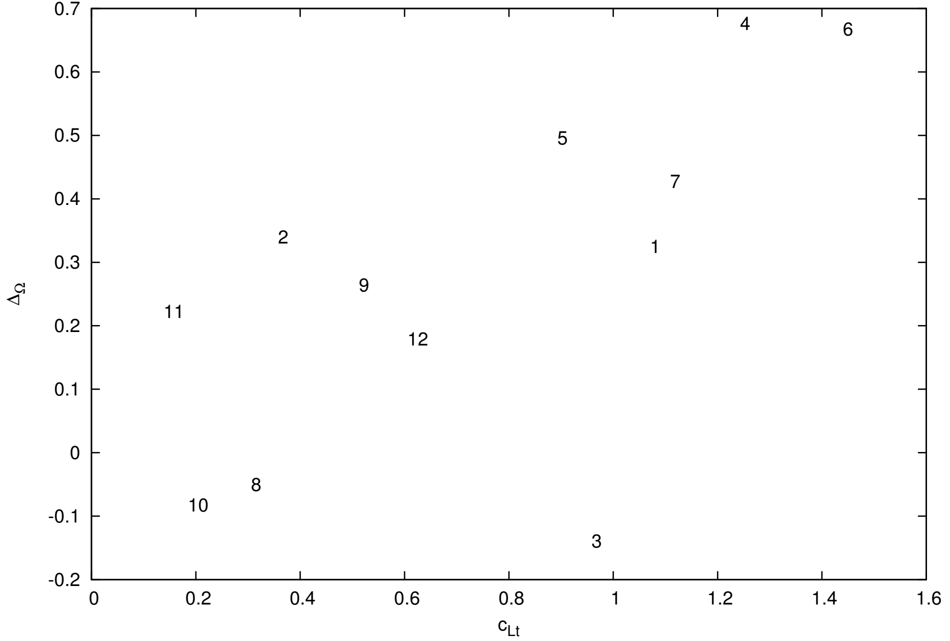

0.7
0.6
0.5
0.4
0.3
0.2
0.1
0
-0.1
-0.2
Δ_Ω
0
0.2
0.4
0.6
0.8
1
1.2
1.4
1.6
c_{Lt}
4
6
5
7
2
1
9
11
12
8
10
3

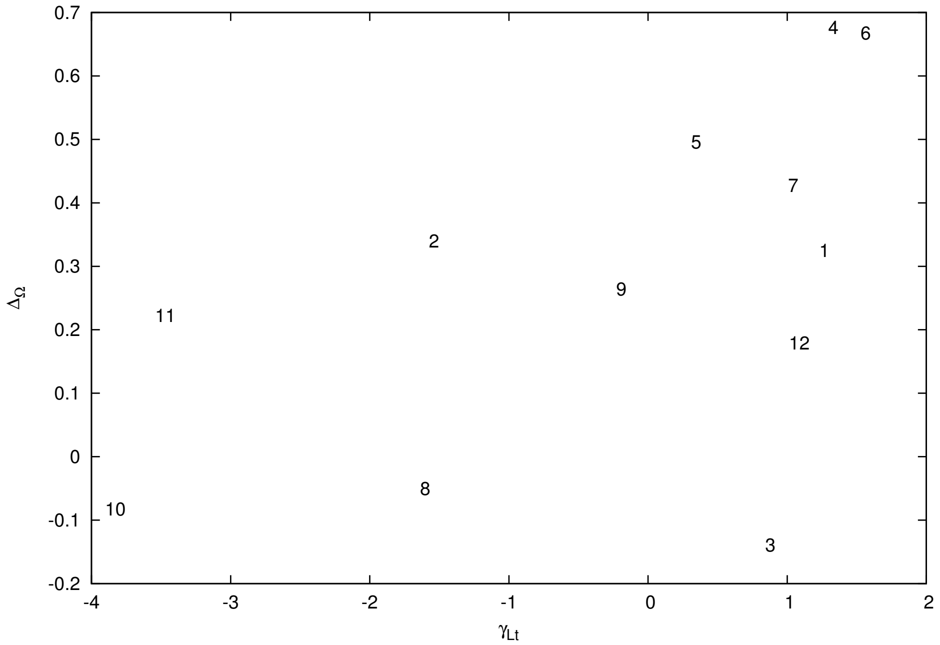

0.7
0.6
0.5
0.4
0.3
0.2
0.1
0
-0.1
-0.2
-4
-3
-2
-1
0
1
2
Δ_Ω
γ_{Lt}
1
2
3
4
5
6
7
8
9
10
11
12

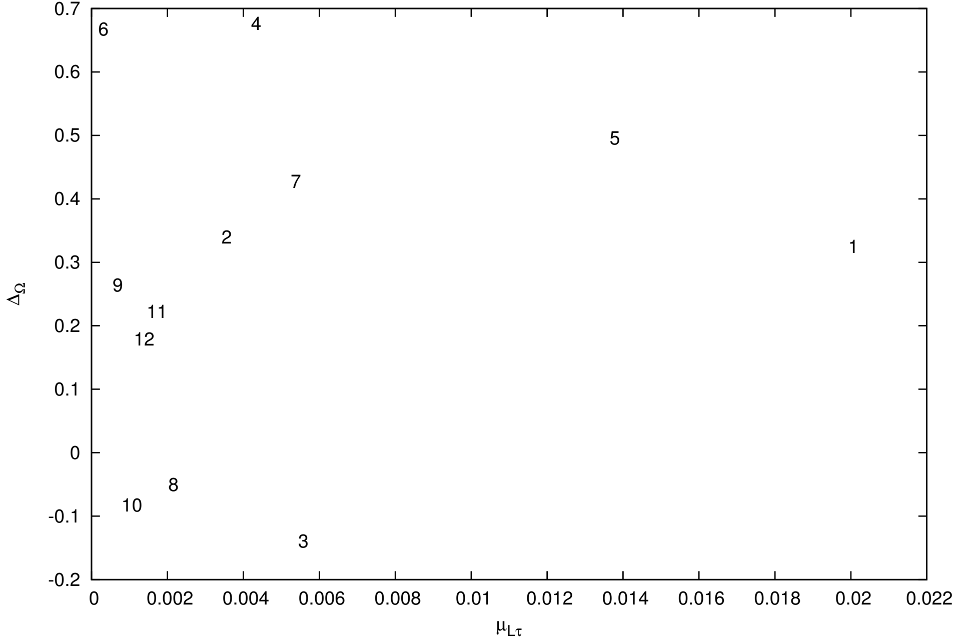

0.7
0.6
0.5
0.4
0.3
0.2
0.1
0
-0.1
-0.2
Δ_Ω
0
0.002
0.004
0.006
0.008
0.01
0.012
0.014
0.016
0.018
0.02
0.022
$\mu_{L\tau}$
6
4
5
7
2
1
9
11
12
8
10
3

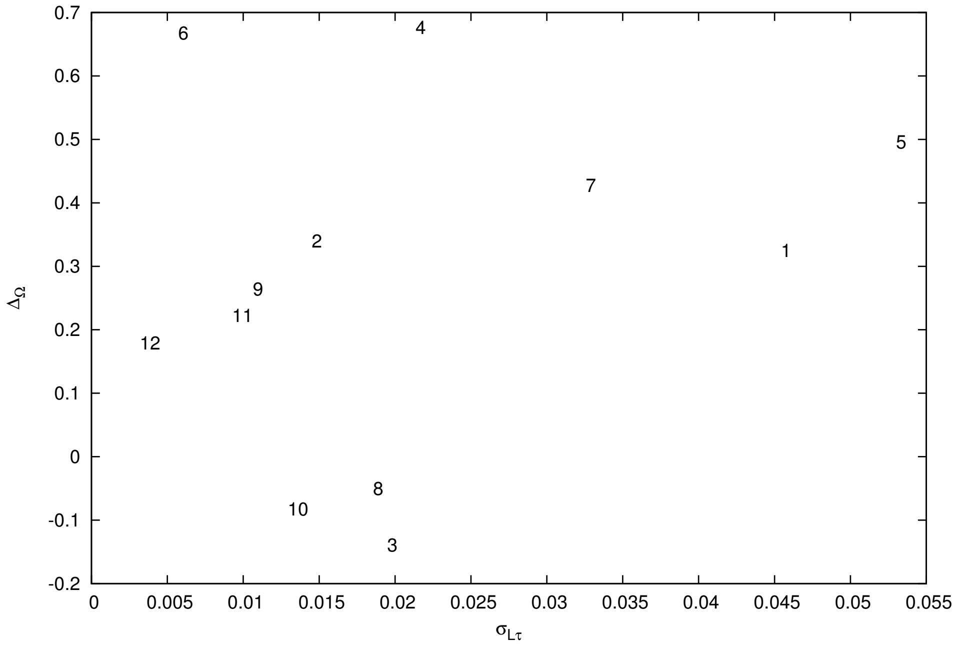

0.7
0.6
0.5
0.4
0.3
0.2
0.1
0
-0.1
-0.2
Δ_Ω
0
0.005
0.01
0.015
0.02
0.025
0.03
0.035
0.04
0.045
0.05
0.055
$\sigma_{L\tau}$
6
4
5
7
2
1
9
11
12
8
10
3

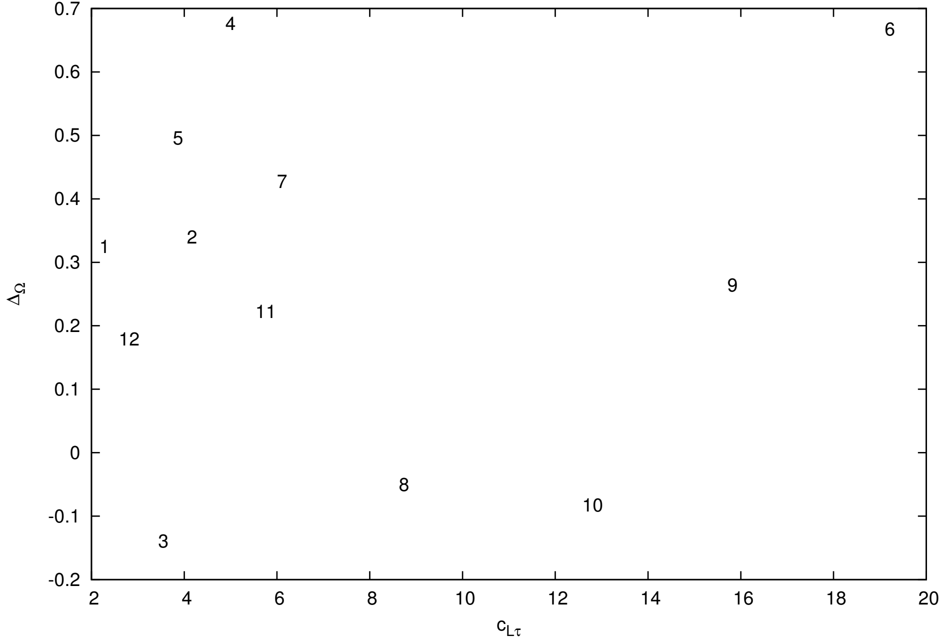

0.7
0.6
0.5
0.4
0.3
0.2
0.1
0
-0.1
-0.2
Δ_Ω
2
4
6
8
10
12
14
16
18
20
$c_{L\tau}$
1
2
3
4
5
6
7
8
9
10
11
12

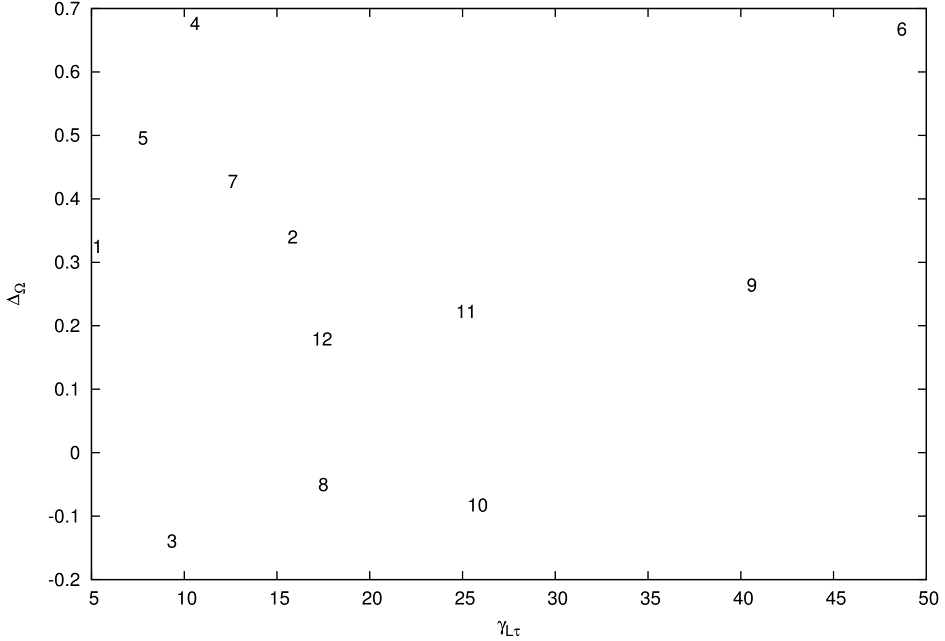

0.7
0.6
0.5
0.4
0.3
0.2
0.1
0
-0.1
-0.2
5
10
15
20
25
30
35
40
45
50
Δ_Ω
$\gamma_{L\tau}$
1
2
3
4
5
6
7
8
9
10
11
12

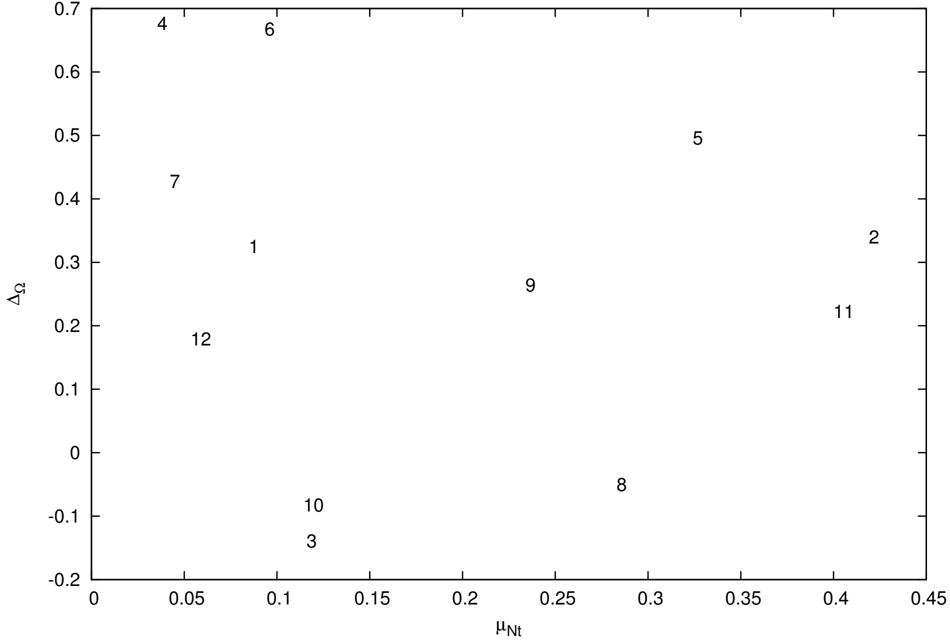
0.7
0.6
0.5
0.4
0.3
0.2
0.1
0
-0.1
-0.2
Δ_Ω
0
0.05
0.1
0.15
0.2
0.25
0.3
0.35
0.4
0.45
μ_Nt
4
6
5
7
1
2
9
11
12
8
10
3

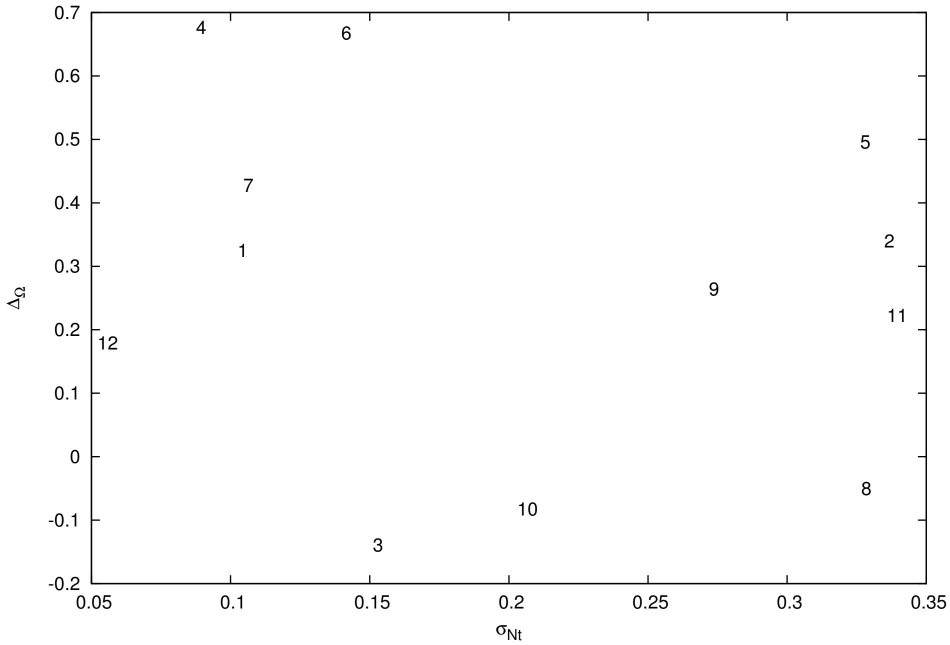

0.7
0.6
0.5
0.4
0.3
0.2
0.1
0
-0.1
-0.2
Δ_Ω
0.05
0.1
0.15
0.2
0.25
0.3
0.35
σ_{Nt}
4
6
5
7
1
2
9
11
12
8
10
3

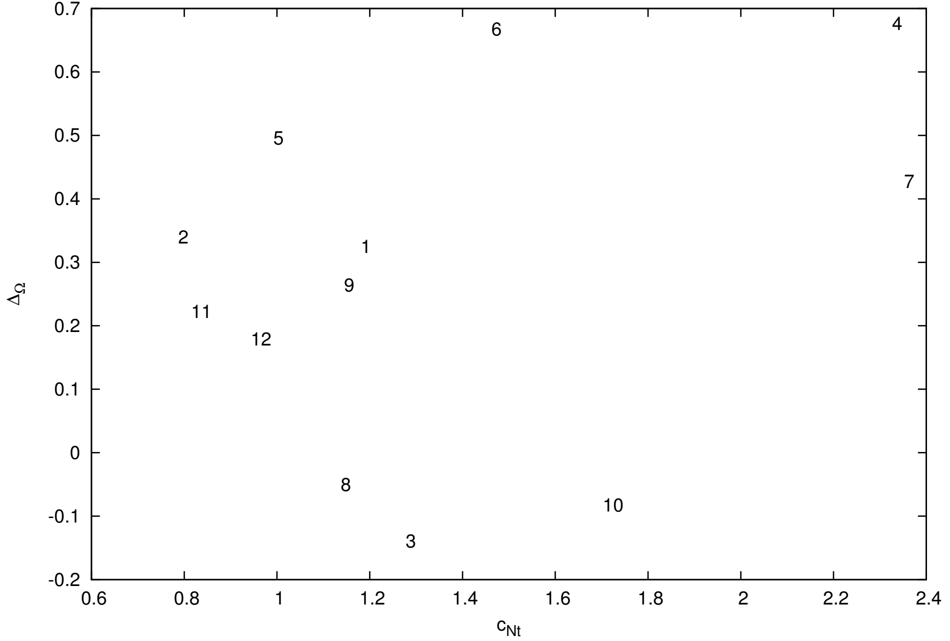

0.7
0.6
0.5
0.4
0.3
0.2
0.1
0
-0.1
-0.2
Δ_Ω
0.6
0.8
1
1.2
1.4
1.6
1.8
2
2.2
2.4
c_{Nt}
1
2
3
4
5
6
7
8
9
10
11
12

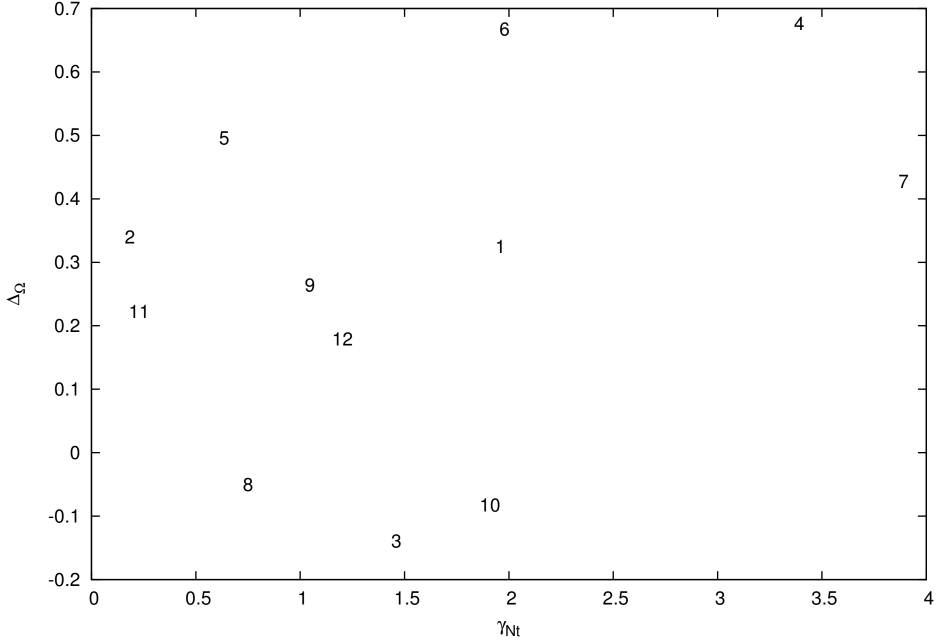

0.7
0.6
0.5
0.4
0.3
0.2
0.1
0
-0.1
-0.2
Δ_Ω
0
0.5
1
1.5
2
2.5
3
3.5
4
γ_{Nt}
1
2
3
4
5
6
7
8
9
10
11
12

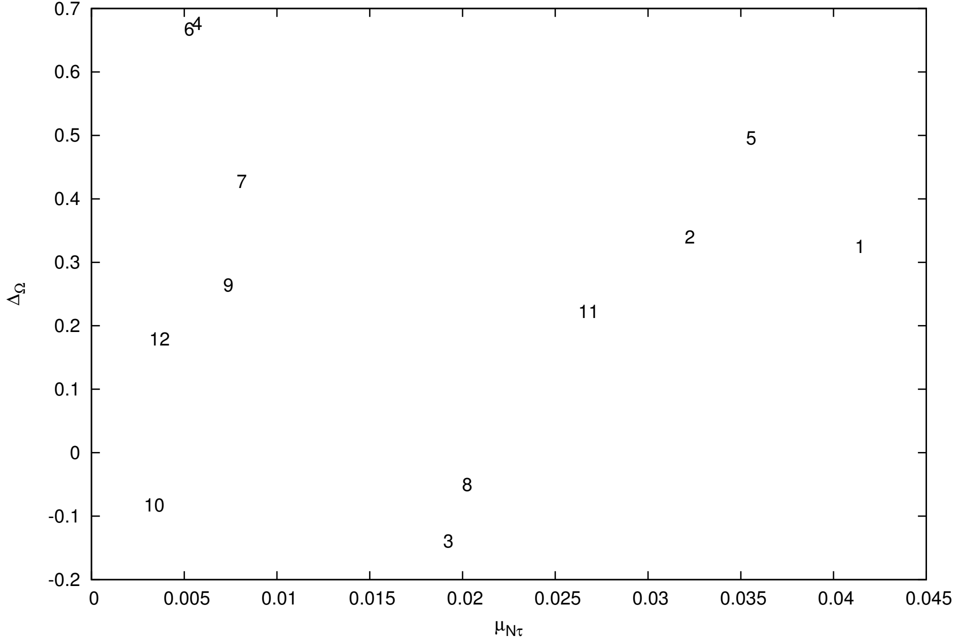

0.7
0.6
0.5
0.4
0.3
0.2
0.1
0
-0.1
-0.2
Δ_Ω
0
0.005
0.01
0.015
0.02
0.025
0.03
0.035
0.04
0.045
$\mu_{N\tau}$
6
4
5
7
2
1
9
11
12
8
10
3

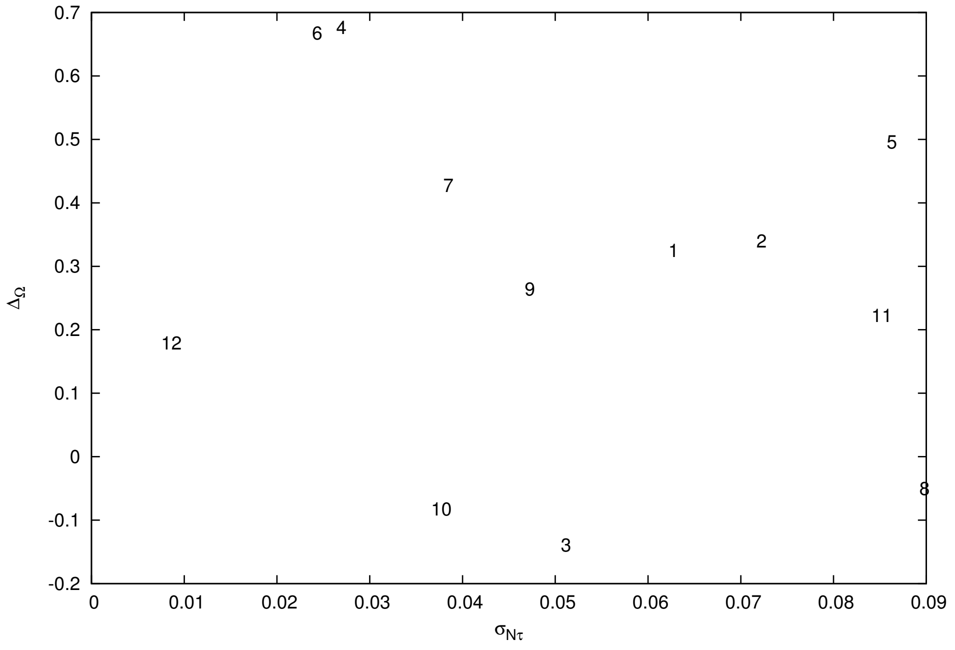

0.7
0.6
0.5
0.4
0.3
0.2
0.1
0
-0.1
-0.2
0
0.01
0.02
0.03
0.04
0.05
0.06
0.07
0.08
0.09
Δ_Ω
$\sigma_{N\tau}$
1
2
3
4
5
6
7
8
9
10
11
12

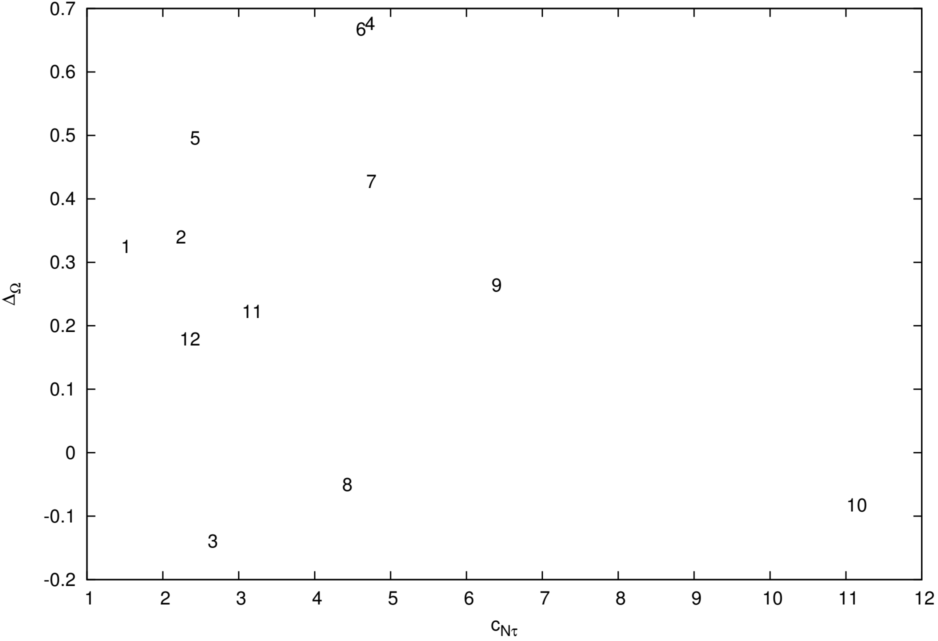

0.7
0.6
0.5
0.4
0.3
0.2
0.1
0
-0.1
-0.2
1
2
3
4
5
6
7
8
9
10
11
12
Δ_Ω
$c_{N\tau}$
6
4
5
7
2
1
9
11
12
8
10
3

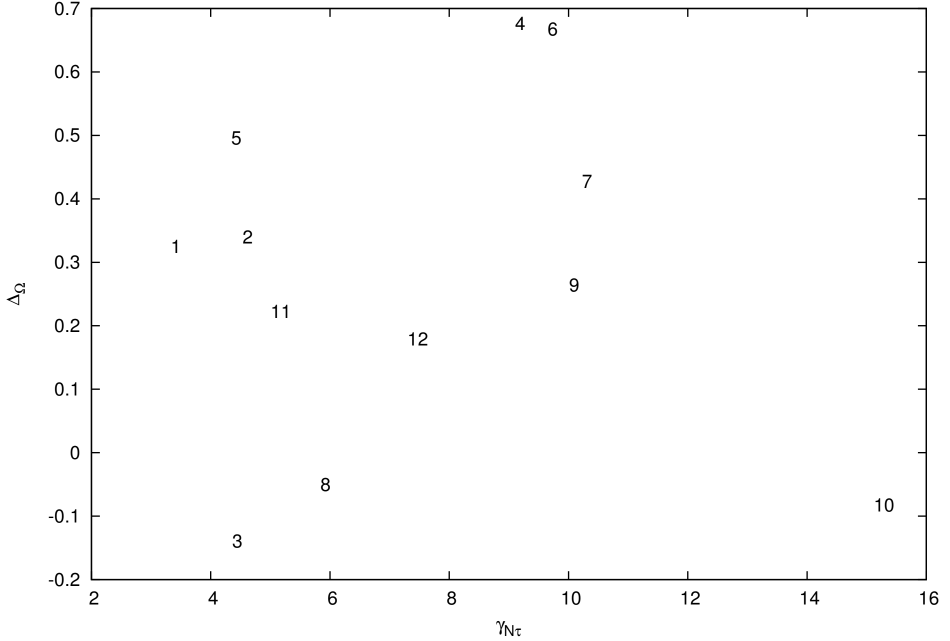

0.7
0.6
0.5
0.4
0.3
0.2
0.1
0
-0.1
-0.2
2
4
6
8
10
12
14
16
Δ_Ω
$\gamma_{N\tau}$
1
2
3
4
5
6
7
8
9
10
11
12

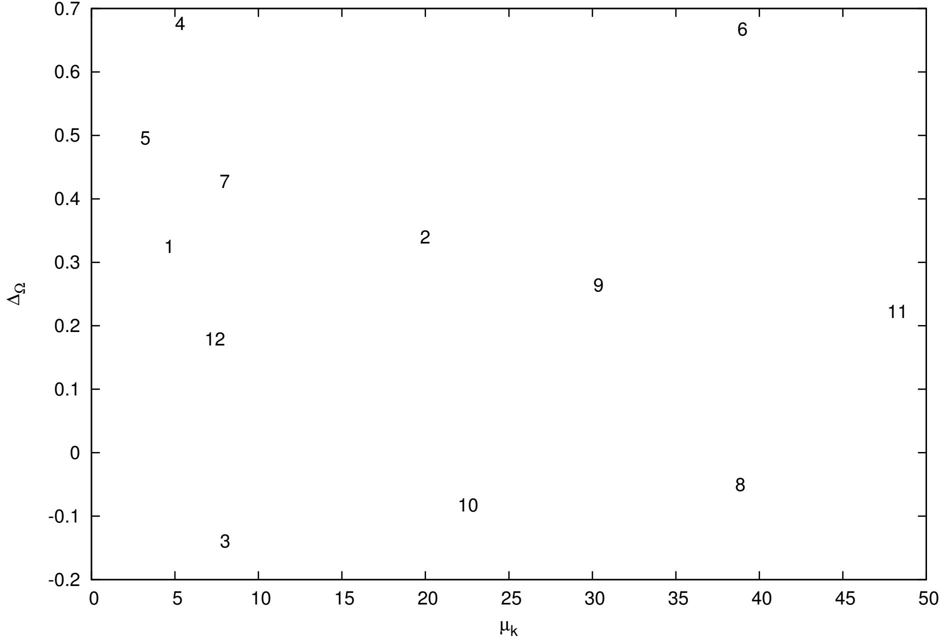

0.7
0.6
0.5
0.4
0.3
0.2
0.1
0
-0.1
-0.2
Δ_Ω
0
5
10
15
20
25
30
35
40
45
50
μ_k
4
6
5
7
2
1
9
11
12
8
10
3

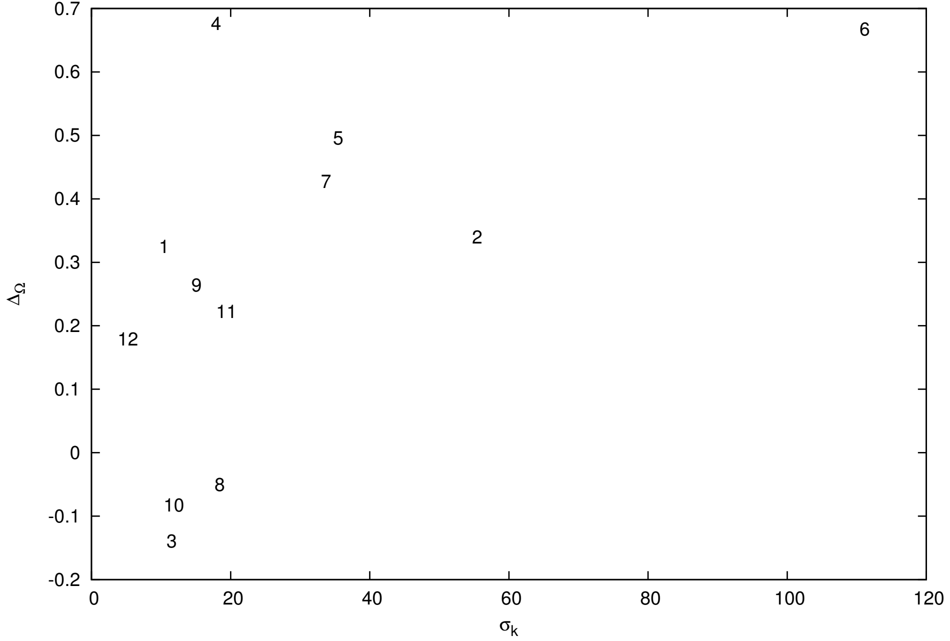

0.7
0.6
0.5
0.4
0.3
0.2
0.1
0
-0.1
-0.2
0
20
40
60
80
100
120
Δ_Ω
σ_k
4
6
5
7
2
1
9
11
12
8
10
3

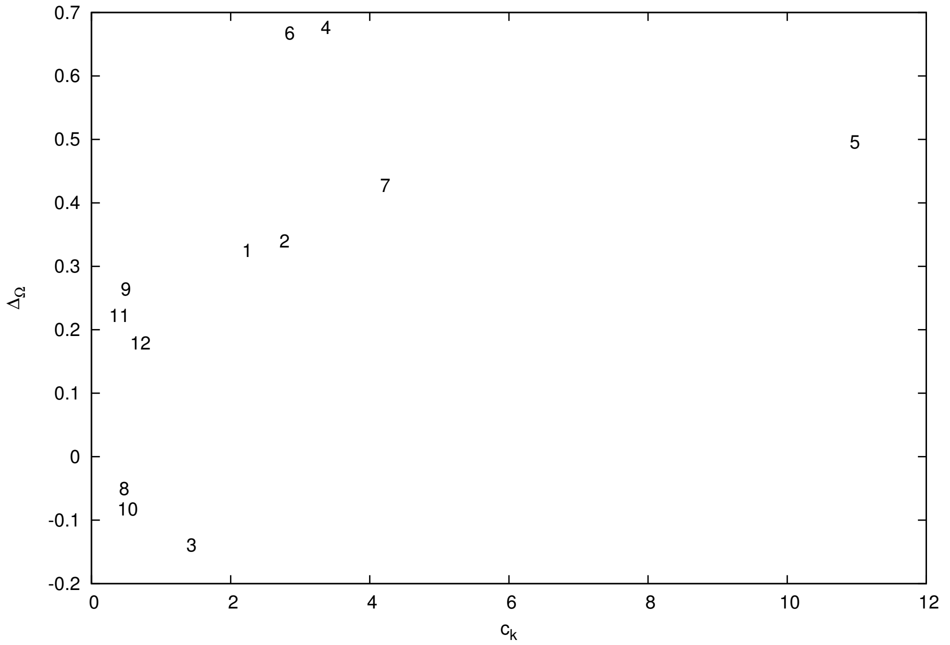

0.7
0.6
0.5
0.4
0.3
0.2
0.1
0
-0.1
-0.2
0
2
4
6
8
10
12
Δ_Ω
c_k
1
2
3
4
5
6
7
8
9
10
11
12

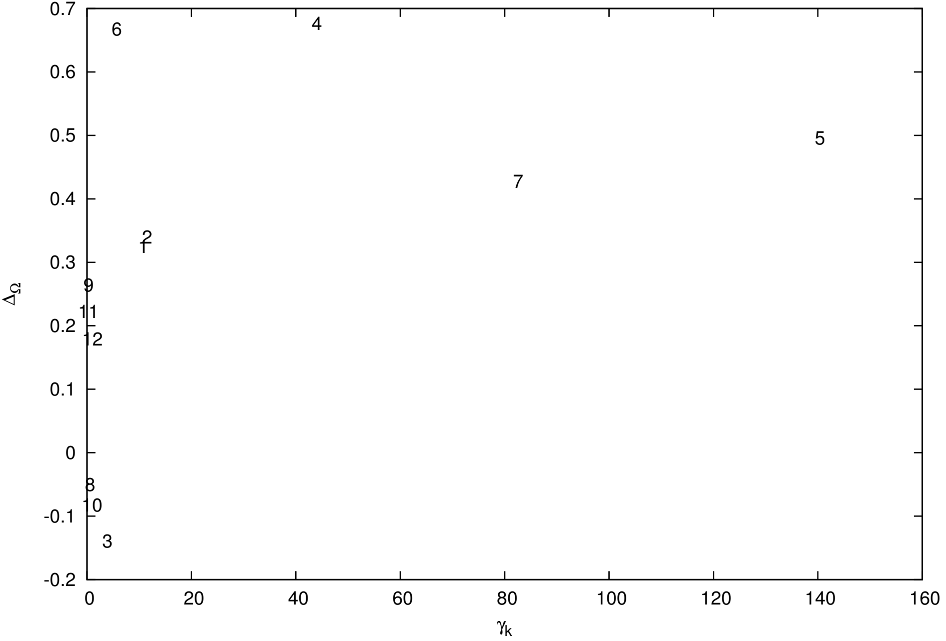

0.7
0.6
0.5
0.4
0.3
0.2
0.1
0
-0.1
-0.2
0
20
40
60
80
100
120
140
160
Δ_Ω
γ_k
1
2
3
4
5
6
7
8
9
10
11
12

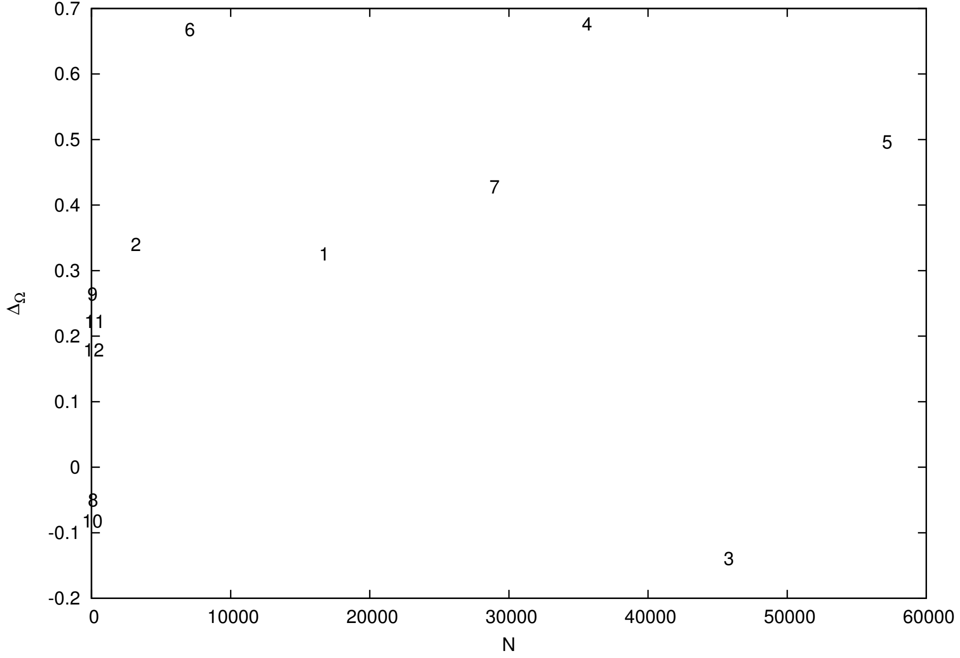

Δ_Ω
N
0.7
0.6
0.5
0.4
0.3
0.2
0.1
0
-0.1
-0.2
0
10000
20000
30000
40000
50000
60000
1
2
3
4
5
6
7
8
9
10
11
12

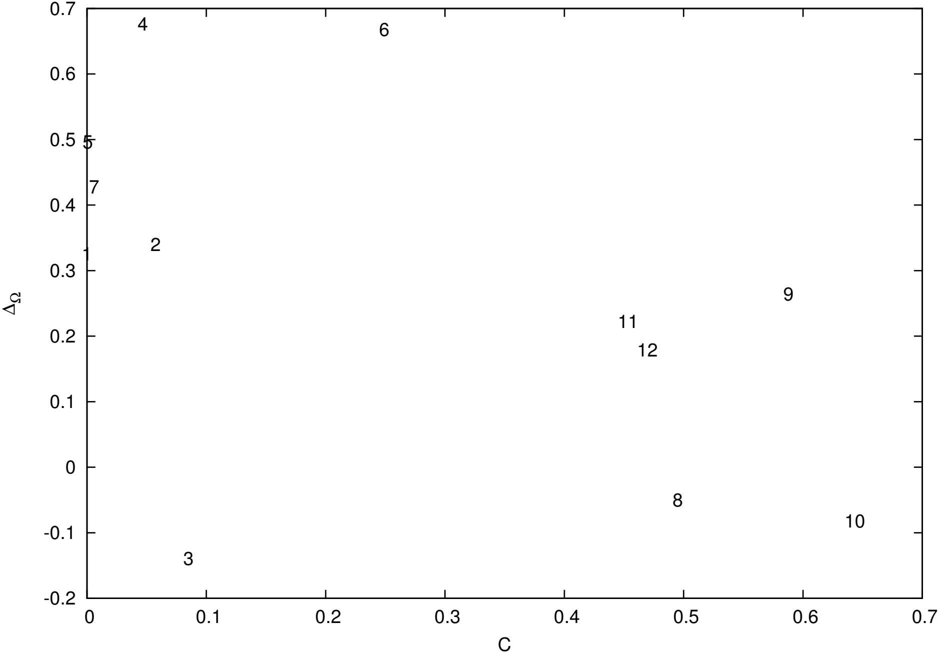

0.7
0.6
0.5
0.4
0.3
0.2
0.1
0
-0.1
-0.2
Δ_Ω
0
0.1
0.2
0.3
0.4
0.5
0.6
0.7
C
1
2
3
4
5
6
7
8
9
10
11
12

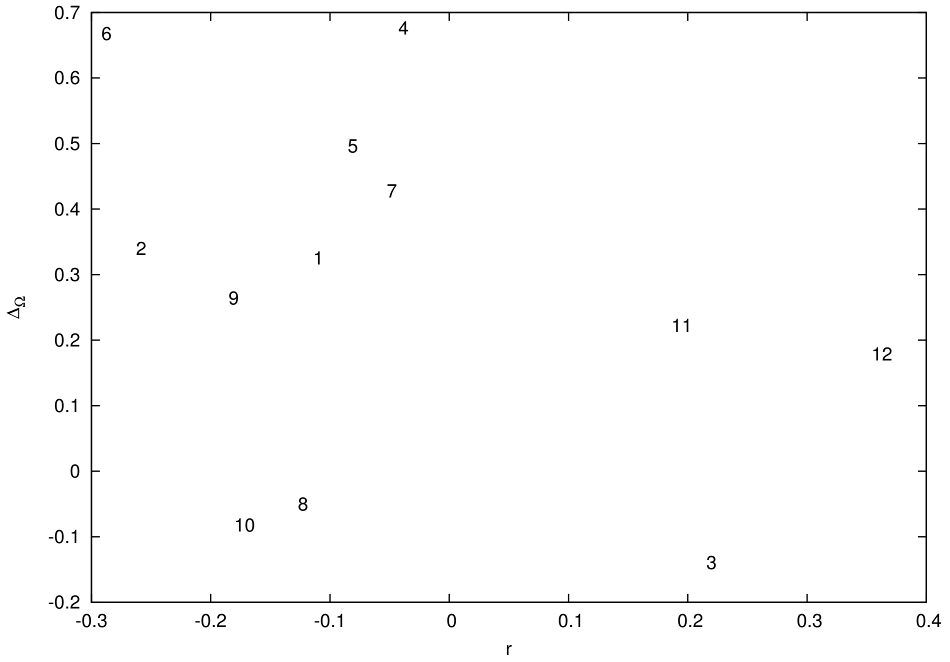

0.7
0.6
0.5
0.4
0.3
0.2
0.1
0
-0.1
-0.2
-0.3
-0.2
-0.1
0
0.1
0.2
0.3
0.4
r
Δ_Ω
6
4
5
7
2
1
9
11
12
8
10
3
